# Global, regional, and national burden of low back pain in postmenopausal women from 1990 to 2021: a comprehensive analysis using data from the Global Burden of Disease Study 2021

**DOI:** 10.3389/fendo.2025.1683183

**Published:** 2025-09-26

**Authors:** Jiaxian Xu, Mingming Lei, Dandan Xu

**Affiliations:** ^1^ College of Sports Medicine and Health, Chengdu Sport University, Chengdu, China; ^2^ Department of Sports Injury, Affiliated Sports Hospital of Chengdu Sport University, Chengdu Sport University, Chengdu, China; ^3^ Department of Intensive Care Unit, The Affiliated Hospital of Xuzhou Medical University, Xuzhou, Jiangsu, China

**Keywords:** low back pain, postmenopausal women, global burden, risk factors, sociodemographic index, public health

## Abstract

**Background:**

Low back pain (LBP) is a leading cause of disability worldwide, with its burden increasing due to population growth and ageing. Postmenopausal women are disproportionately affected, largely owing to estrogen decline, which accelerates osteoporosis and intervertebral disc degeneration. This study aimed to quantify the global, regional, and national burden of LBP among postmenopausal women from 1990 to 2021, using data from the Global Burden of Disease (GBD) Study 2021.

**Methods:**

We analyzed prevalence, incidence, and disability-adjusted life years (DALYs) of LBP in women aged 55 years and older across 204 countries and territories from 1990 to 2021. Temporal trends were assessed using age-standardized rates (ASRs) and estimated annual percentage changes (EAPCs). Contributions of three modifiable risk factors—high body-mass index (BMI), smoking, and occupational or environmental exposures—were examined by Socio-demographic Index (SDI) quintiles.

**Findings:**

Between 1990 and 2021, incident cases nearly doubled (from 35.2 million to 70.3 million), prevalent cases rose from 89.9 million to 176.8 million, and DALYs increased from 9.8 million to 19.1 million. Despite these increases, age-standardized incidence, prevalence, and DALY rates declined modestly. In 2021, the burden in postmenopausal women was 1.78 times higher for incidence, 1.86 times higher for prevalence, and 1.84 times higher for DALYs than in age-matched men. High BMI was the leading modifiable risk factor globally, contributing to 14.2% of DALYs, followed by occupational or environmental risks (12.6%) and smoking (7.3%). Regional variation was marked: high and high-middle SDI regions showed declining ASRs, whereas middle and low-middle SDI regions experienced increases.

**Interpretation:**

The global burden of LBP among postmenopausal women has risen substantially, despite declines in age-standardized rates. High BMI, occupational exposures, and smoking are key modifiable drivers, with varying impacts by development level. Public health strategies should prioritize weight management, workplace interventions, and smoking cessation, with particular focus on high-risk age groups and low-resource regions.

## Introduction

Low back pain (LBP) is a common musculoskeletal disorder (1) and the leading cause of disability worldwide. Its burden continues to rise with population growth and ageing (2, 3), and the Global Burden of Disease (GBD) Study projects that more than 800 million people will be affected by 2050 (4). Postmenopausal women experience a disproportionate burden of severe LBP compared with men (5), primarily due to the sharp decline in estrogen levels after menopause, which accelerates osteoporosis and intervertebral disc degeneration (6, 7).

Beyond its health impact, LBP imposes a significant economic burden. It is among the leading drivers of healthcare expenditure, owing to frequent consultations, diagnostic imaging, and pharmacological treatment, and it contributes substantially to productivity loss through absenteeism and presenteeism; these direct and indirect costs make LBP one of the most expensive musculoskeletal disorders worldwide, underscoring the urgency of addressing it as both a public health and socioeconomic challenge, particularly among the growing population of postmenopausal women (2, 4).

The etiology of LBP is complex (8). In postmenopausal women, estrogen depletion contributes to intervertebral disc degeneration (5). Additional contributors include central obesity (9, 10), sarcopenia (9, 11), sleep disorders (12, 13), psychological factors (14, 15), and vitamin D deficiency (5). In terms of treatment, non-pharmacological approaches (e.g., heat application, manual therapy, and exercise) and pharmacological interventions (e.g., NSAIDs and muscle relaxants) can relieve pain and disability (16). However, prolonged use of medication carries risks of adverse effects (17). Opioid overuse is a major concern, and autonomic dysfunction in postmenopausal women further complicates the selection of pharmacological treatments, warranting further investigation (12, 18). Wang et al. demonstrated that minodronic acid alleviates LBP symptoms in patients with osteoporosis (6). Nevertheless, a systematic and comprehensive treatment protocol for postmenopausal women has yet to be established, and adherence to multidisciplinary clinical pathways and guidelines requires strengthening.

By analyzing global, regional, and national trends in LBP among postmenopausal women, this study supports health decision-makers in allocating resources and prioritizing interventions in high-burden areas. It also elucidates key risk factors to inform targeted prevention and health education strategies, while providing insights for clinical practice to optimize management and improve quality of life.

## Methods

### Data sources

We used data from the GBD 2021, which provides epidemiological estimates for 371 diseases and injuries across 204 countries and territories from 1990 to 2021. The dataset includes prevalence, incidence, severity, and DALYs for LBP but excludes mortality, as LBP rarely causes death (4). Accordingly, we focused on incident cases, prevalent cases, and DALYs to provide a comprehensive assessment of the burden of LBP. Data sources included surveys, disease registries, hospital records, and administrative datasets. Further details on data sources, methods, and statistical models are provided in previous GBD publications and on the Global Health Data Exchange (GHDx) website.

### Case definition

Within the GBD framework, cases of LBP were defined by radiological confirmation or clinical diagnosis of symptomatic LBP. In this study, postmenopausal women were defined as those aged 55 years or older, consistent with previous epidemiological studies using comparable data sources (19, 20). This approach approximates menopausal status in the absence of direct data in the GBD dataset, although it may underestimate women who experience early menopause. In the GBD 2021 study, the main data sources for the LBP model were global cross-sectional, population-based surveys and insurance claims data from US states. The claims used International Classification of Diseases (ICD)-10 codes M54.5 (low back pain) and M54.1 (cervicobrachial syndrome, if related to a radiating pain pattern). GBD 2021 also included data from systematic reviews and meta-analyses that identified LBP cases using ICD-10 codes. The inclusion criteria for LBP were pain in the lumbar region, with or without radiating pain to the lower limbs, while excluding other specific spinal diseases (ICD-10 codes M50–M54, except M54.5).

### Global/regional sociodemographic characteristics

We used the Sociodemographic Index (SDI) and GBD 2021 regional classifications to analyze variations in the burden of LBP. The SDI provides a standardized framework for comparing health outcomes by stratifying countries and regions into five quintiles—high, high-middle, middle, low-middle, and low SDI—based on composite measures of development (19). In addition, the 204 countries and territories were grouped into 21 regions according to geography and cultural similarity. Together, SDI and regional classification enable a detailed analysis of the global distribution of LBP burden.

### Socio-demographic index

Given its methodological importance, the SDI is described in more detail here. Developed by the Institute for Health Metrics and Evaluation (IHME; https://vizhub.healthdata.org/gbd-compare/), the SDI is a composite indicator of development that integrates three dimensions: lag-distributed income per capita (reflecting economic status), average years of education among individuals aged 15 years or older (reflecting educational attainment), and the total fertility rate among women younger than 25 years (reflecting demographic structure). These three components are combined into a single index ranging from 0 to 1, with higher values indicating higher levels of development. According to 2021 estimates, 204 countries and territories were categorized into five SDI quintiles: low (<0.466), low-middle (0.466–0.619), middle (0.619–0.720), high-middle (0.720–0.810), and high (≥0.810) (21).

### Risk factors for LBP

We examined three modifiable risk factors for LBP: high body mass index (BMI), smoking, and environmental/occupational exposures. In the GBD 2021 study, occupational ergonomic factors, elevated body mass index (BMI), and smoking were identified as risk factors for which there was substantial evidence supporting risk–outcome associations; this evidence was characterized by the inclusion of multiple study types, at least two cohort studies, an absence of significant and unexplained heterogeneity, a low risk of confounding and selection bias, and biologically plausible dose–response gradients (22). Based on GBD 2021, high BMI was defined as BMI ≥25 kg/m² for adults (≥20 years), aligned with GBD’s theoretical minimum risk exposure level (TMREL) protocols (23). The inclusion of high BMI as a risk factor was based on a rigorous systematic review of longitudinal cohort studies and the application of strict causal criteria to establish its association with LBP (24). Environmental/occupational risks included factors such as ergonomic stress and occupational hazards, identified through systematic reviews and expert consensus (4, 22). Smoking was identified as a risk factor based on its association with chronic pain and musculoskeletal diseases (25, 26).

### Statistical analysis

Each disability-adjusted life year (DALY) represents one year of healthy life lost and is calculated by combining years lived with disability (YLDs) derived from the GBD dataset. As there are no mortality data for LBP in the GBD dataset, there are no years of life lost due to premature death (YLLs). This study assessed the disease burden of LBP using the number of incident cases, prevalent cases, and DALYs. The age-standardized rates (ASRs) and corresponding 95% uncertainty intervals (UIs) for the incidence, prevalence, and DALYs of LBP in postmenopausal women were calculated using the following formula:


$$\text{ASR}=\frac{\sum_{k=1\;}^{n}T_{k}}{\sum_{k}^{n}I_{k}T_{k}}$$

Where Ik is the proportion of the population in a specific age group, and Tk is the number or weight of the standard population in the same age group. This method ensures comparability across regions and over time, minimizing biases due to varying age structures. The dynamic trends in the prevalence, incidence, and DALYs of LBP from 1990 to 2021 were assessed using the estimated annual percentage change (EAPC) and its 95% confidence interval (CI), calculated as follows:


y=α+βx+ϵ

EAPC=100×(exp(β)−1)

Where y is the natural logarithm of the ASR, and x is the calendar year. In GBD methodology, all estimates are accompanied by 95% uncertainty intervals (UIs). These UIs are derived from 1,000 draws from the posterior distribution of each estimate, incorporating uncertainty from multiple sources, including input data, model specifications, and parameter estimation. The 2.5th and 97.5th percentiles of the ordered draws are presented as the lower and upper bounds of the 95% UI. This approach accounts for study variability and data heterogeneity, providing a more conservative and transparent estimate than standard confidence intervals.

An EAPC greater than 0 indicates an increasing trend in ASRs, whereas an EAPC less than 0 indicates a decreasing trend. If the 95% CI of the EAPC includes 0, it indicates no significant change. We performed stratified analyses to assess the interaction between BMI and SDI on the risk of LBP, enabling us to explore differences in the relationship between BMI and the burden of LBP across different regions and income levels. We also categorized postmenopausal women into specific age groups (55-59, 60-64, 65-69, 70-74, 75-79, 80-84, 85-89, 90-94, and 95 years and older) to better understand the age-related variations in the burden of LBP within this population. All ASRs in this study were expressed per 100,000 population. Data cleaning, calculations, and graphical representations were completed using R programming (v4.5.0).

## Results

### Global burden of LBP in postmenopausal women from 1990 to 2021: temporal trends and gender disparities

#### Temporal trends

**Figure 1:**
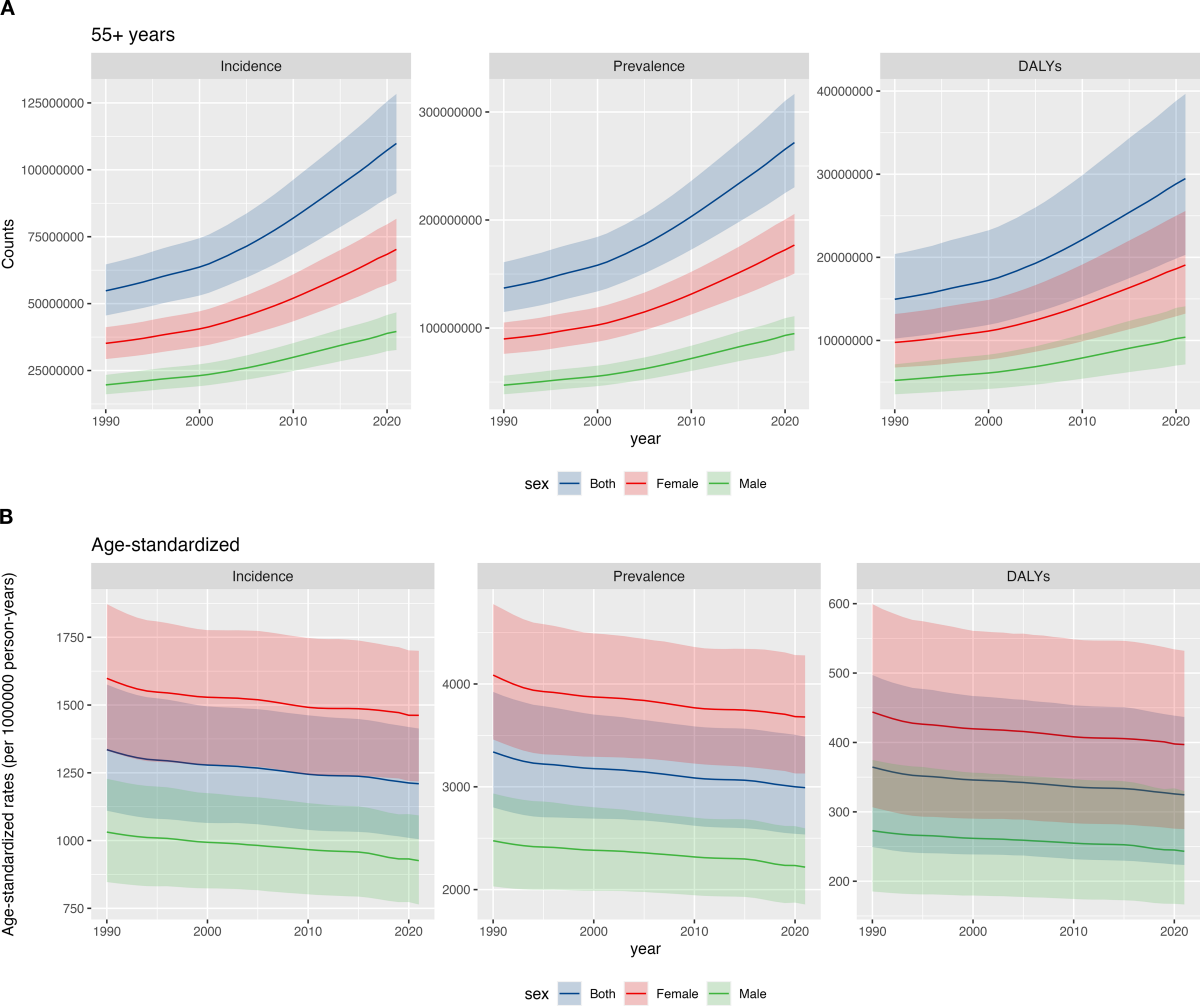
Global burden of LBP in postmenopausal women from 1990 to 2021: temporal trends and gender disparities: **(A)** counts; **(B)** age-standardized rates.

Between 1990 and 2021, the global incidence of LBP in postmenopausal women increased markedly. New cases rose from 35.2 million in 1990 (95% UI 29.3–41.2 million) to 70.3 million in 2021 (58.6–81.7 million). During this period, prevalence also increased substantially, with prevalent cases increasing from 89.9 million in 1990 (95% UI 76.1–105.1 million) to 176.8 million in 2021 (150.4–205.6 million). Similarly, DALYs increased from 9.8 million (95% UI 6.7–13.2 million) to 19.1 million (13.2–25.6 million) (**Figure 1A**, appendix pp 1-9). However, the age-standardized incidence rate (ASIR) declined from 1,598.5 per 100,000 in 1990 (95% UI 1,331.7–1,872.5) to 1,462.1 per 100,000 in 2021 (1,218.2–1,700.2). The age-standardized prevalence rate (ASPR) also declined, from 4,086.5 per 100,000 in 1990 (3,460.2–4,777.1) to 3,679.1 per 100,000 in 2021 (3,129.3–4,277.9). Similarly, the age-standardized DALY rate (ASDR) fell from 443.8 per 100,000 in 1990 (306.6–599.4) to 396.9 per 100,000 in 2021 (275.2–532.1) (**Figure 1B**, appendix pp 9-18).

#### Gender differences

Between 1990 and 2021, the burden of LBP in postmenopausal women remained substantially higher than in age-matched men. In 2021, the number of new cases in women was 1.78 times that in men (70.3 million [95% UI 58.6–81.7 million] vs 39.6 million [32.7–46.7 million]). Prevalent cases were 1.86 times higher in women (176.8 million [150.4–205.6 million] vs 94.8 million [79.3–111.0 million]), and DALYs were 1.84 times higher (19.1 million [13.2–25.6 million] vs 10.4 million [7.1–14.1 million]) (**Figure 1A**, appendix pp 19-41). During this period, the ASIR, ASPR, and ASDR of LBP in postmenopausal women and age-matched men both decreased, mirroring the overall population trend (**Figure 1**). In 2021, the ASIR in postmenopausal women was 1.58 times higher than in men [1,462.1 (95% UI 1,218.2–1,700.2) per 100,000 vs 925.9 (764.3–1,092.8) per 100,000]. The ASPR was 1.66 times higher in postmenopausal women than in men [3,679.1 (95% UI 3,129.3–4,277.9) per 100,000 vs 2,218.7 (1,855.6–2,595.7) per 100,000]. The ASDR was 1.63 times higher in postmenopausal women than in men [396.9 (95% UI 275.2–532.1) per 100,000 vs 243.1 (166.6–329.9) per 100,000] (**Figure 1B**, appendix pp 41-68).

### Regional analysis of the burden of LBP in postmenopausal women from 1990 to 2021

**Figure 2:**
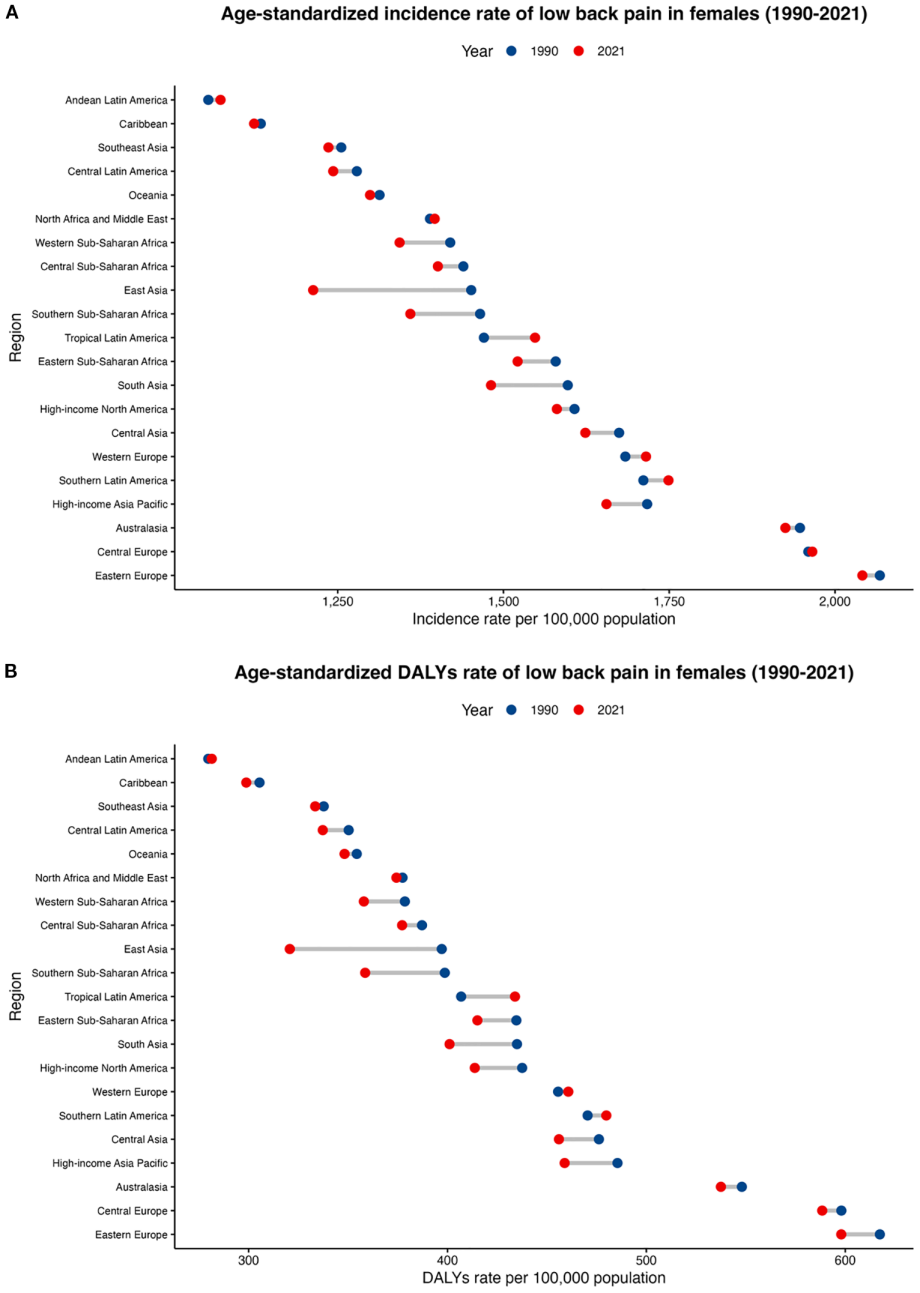
Regional age-standardized incidence rate and age-standardized DALY rate of LBP in postmenopausal women from 1990 to 2021. **(A)** age-standardized incidence rate; **(B)** age-standardized DALY rate.

Stratified by SDI quintiles, the incidence and DALY rate of LBP in postmenopausal women decreased across all global regions. The most pronounced reductions were observed in the High-middle SDI region, with an EAPC of ASIR at -0.39 (-0.42 to -0.37) and an EAPC of ASDR at -0.47 (-0.49 to -0.44). Specifically, the ASIR declined from 1,724.4 per 100,000 in 1990 (95% UI 1,442.1–2,009) to 1,507.5 per 100,000 in 2021 (95% UI 1,249.1–1,754.4), and the ASDR decreased from 488.5 per 100,000 in 1990 (338.1–661.8) to 413.9 per 100,000 in 2021 (287.4–555.5). The regions with the least decline in ASIR and DALY rate were the High SDI countries, with an EAPC of ASIR at -0.02 (-0.04 to 0) and an EAPC of ASDR at -0.08 (-0.1 to -0.06). In 2021, regions with Middle and High SDI had higher incidence and DALYs. At the regional level, from 1990 to 2021, the ASIR of LBP in postmenopausal women decreased in most regions globally, with a few experiencing an increase. The largest decline was in East Asia (EAPC -0.39), followed by South Asia (EAPC -0.25) and Southern Sub-Saharan Africa (EAPC -0.20). The smallest decline was in the Caribbean (EAPC -0.01), while the largest increase was in Tropical Latin America (EAPC 0.13). The smallest increase was in North Africa and the Middle East (EAPC 0.01). Similarly, the ASDR decreased in most regions, with the largest declines in East Asia (EAPC -0.46), Southern Sub-Saharan Africa (EAPC -0.28), and South Asia (EAPC -0.25). The smallest declines were in Southeast Asia (EAPC -0.02) and North Africa and the Middle East (EAPC -0.02). The burden in postmenopausal women remained higher than that in men of the same age group across all regions (**Figure 2**, **Supplementary Table 1**).

### Trends in LBP in postmenopausal women across nations from 1990 to 2021

**Figure 3:**
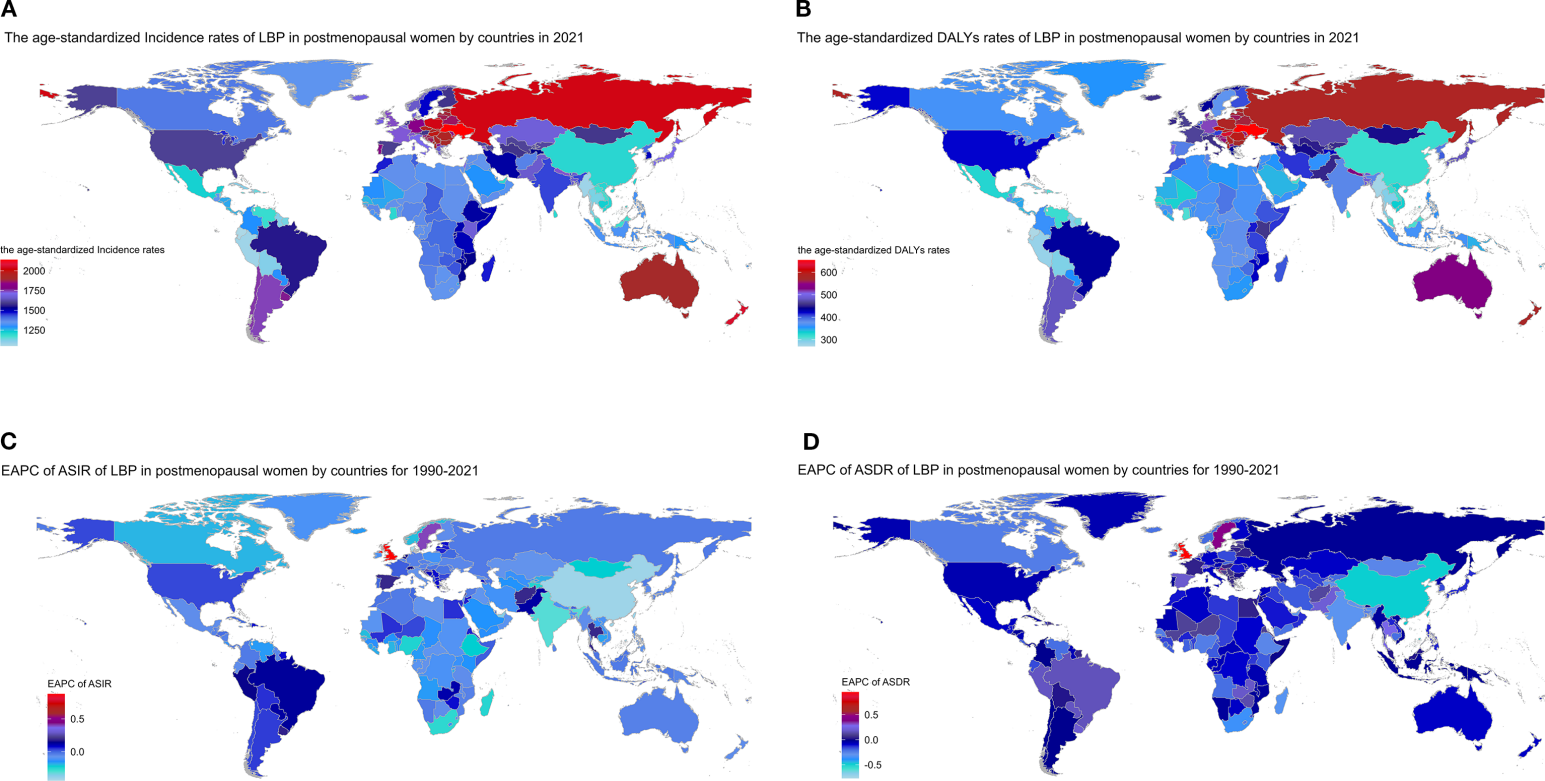
Trends in LBP in postmenopausal women across nations from 1990 to 2021: **(A)** age-standardized incidence rates in 2021; **(B)** age-standardized DALY rates in 2021; **(C)** EAPC of ASIR; **(D)** EAPC of ASDR.

In 2021, China, India, and the USA had the highest numbers of new LBP cases among postmenopausal women, reporting 14.4 million (95% UI 11.9–16.9), 9.1 million (7.5–10.9), and 5.3 million (4.6–5.9), respectively. These countries also had the greatest DALY counts, with China at 3.8 million (2.6–5.1), India at 2.4 million (1.7–3.3), and the USA at 1.4 million (1.0–1.8) (appendix pp 68–140). The highest age-standardized incidence rates were observed in Ukraine (2140.1 per 100,000; 1777.7–2514.2), New Zealand, and Poland, whereas Ecuador (1050.1 per 100,000; 870.4–1235.3), Peru, and Myanmar had the lowest (**Figure 3A**). For age-standardized DALY rates, Ukraine (654.7 per 100,000; 462.6–904.6), Hungary, and Czechia ranked highest, while Ecuador (270.4 per 100,000; 188.4–359.1), Myanmar, and Peru ranked lowest (**Figure 3B**; appendix pp 140–159). From 1990 to 2021, ASIR trends varied across 198 countries and territories. The largest increases were in the UK (EAPC 0.88), Taiwan (Province of China) (0.42), and Sweden (0.40), whereas Denmark (-0.44) and China (-0.41) showed the steepest declines (**Figure 3C**; appendix pp 160–169). ASDR trends were similarly heterogeneous: the UK had the largest increase (0.95), while Denmark had the greatest decrease (-0.77). Other notable changes included increases in Taiwan (0.71), and Sweden (0.45) and decreases in China (-0.49) and South Africa (-0.36) (**Figure 3D**; appendix pp 169–175). These findings highlight substantial cross-national variation in the burden of LBP among postmenopausal women, underscoring the need for context-specific public health interventions.

### Analysis of risk factors for LBP in postmenopausal women across global regions (2021): correlation with SDI

**Figure 4:**
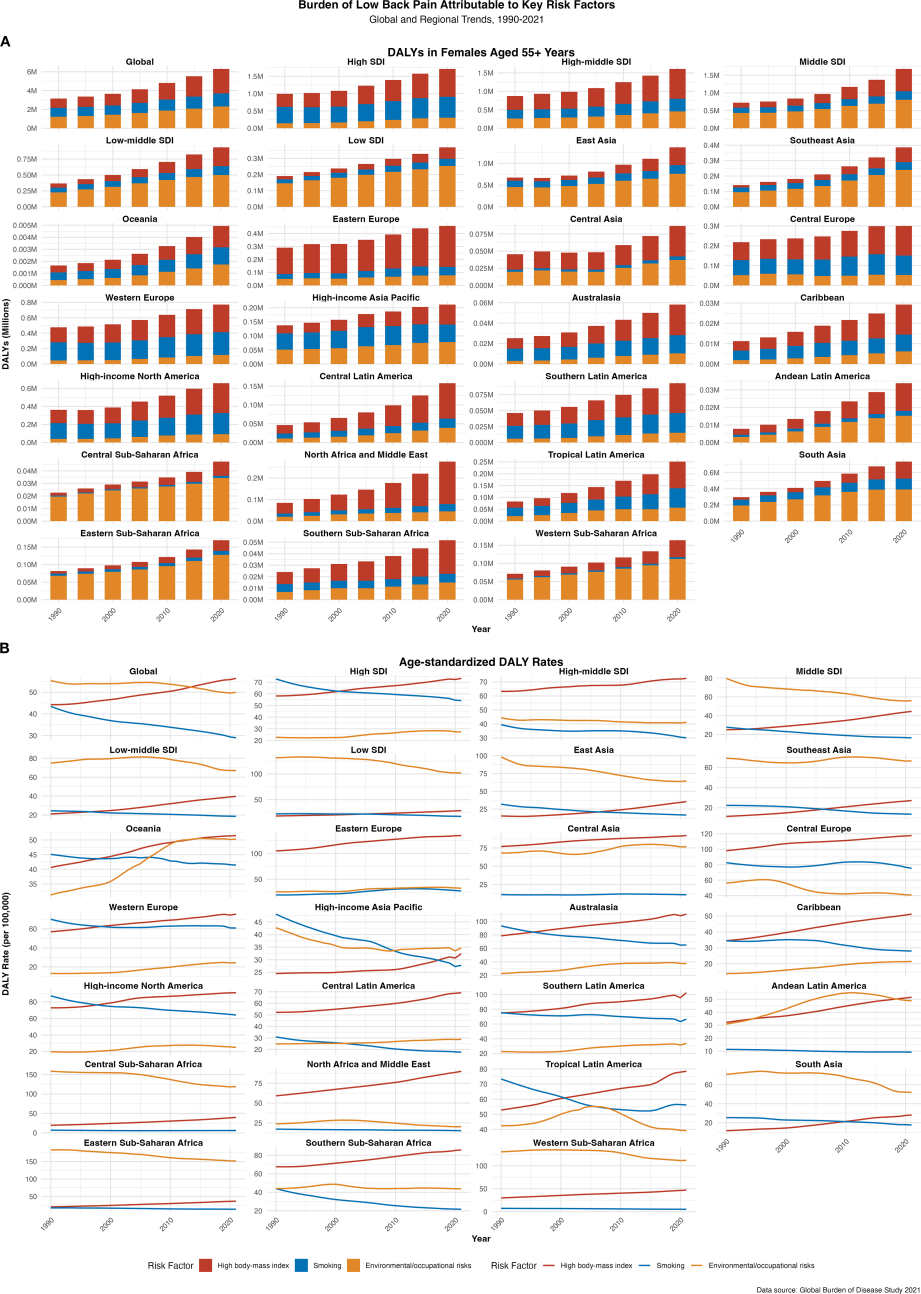
Analysis of risk factors for LBP in postmenopausal women across global regions from 1990 to 2021: correlation with the socio-demographic index (SDI) **(A)** DALYs; **(B)** the age-standardized DALY rate.

In 2021, the global total DALYs attributable to LBP in postmenopausal women were 19.078 million (95% UI 13.229–25.575 million). Of these, 34.1% (95% UI 14.0–51.9) were attributable to three major modifiable GBD risk factors: high BMI (14.2%, 2.709 million DALYs), environmental/occupational risks (12.6%, 2.401 million DALYs), and smoking (7.3%, 1.393 million DALYs). High BMI has become the leading modifiable driver of LBP burden globally. In high SDI regions, the total DALYs attributable to LBP were 5.021 million (95% UI 3.571–6.639 million). The three major modifiable risk factors accounted for 34.7% of the attributable burden: high BMI (16.4%, 0.826 million DALYs), smoking (12.2%, 0.612 million DALYs), and environmental/occupational risks (6.2%, 0.309 million DALYs). High BMI contributed more than smoking and occupational risks, highlighting the need for metabolic interventions. In high-middle SDI regions, the total DALYs were 4.753 million (95% UI 3.301–6.379 million). The three major modifiable risk factors accounted for 32.5% of the attributable burden: high BMI (17.5%, 0.833 million DALYs), environmental/occupational risks (9.9%, 0.473 million DALYs), and smoking (7.3%, 0.346 million DALYs). High BMI emerged as the leading driver, suggesting a focus on weight management and metabolic risk control. In middle SDI regions, the total DALYs were 5.230 million (95% UI 3.566–7.107 million). The three major modifiable risk factors accounted for 35.9% of the attributable burden: environmental/occupational risks (16.1%, 0.842 million DALYs), high BMI (12.8%, 0.669 million DALYs), and smoking (4.7%, 0.246 million DALYs). Occupational and environmental factors exceeded high BMI as contributors, indicating a need for labor environment improvements and occupational health interventions. In low-middle SDI regions, the total DALYs were 3.037 million (95% UI 2.074–4.157 million). The three major modifiable risk factors accounted for 31.8% of the attributable burden: environmental/occupational risks (16.9%, 0.515 million DALYs), high BMI (10.0%, 0.305 million DALYs), and smoking (4.7%, 0.142 million DALYs). Occupational and environmental factors remained the leading drivers, emphasizing the need for labor protection and community-level interventions. In low SDI regions, the total DALYs were 1.018 million (95% UI 0.699–1.402 million). The three major modifiable risk factors accounted for 36.9% of the attributable burden: environmental/occupational risks (25.6%, 0.261 million DALYs), high BMI (7.2%, 0.073 million DALYs), and smoking (4.4%, 0.045 million DALYs). Occupational and environmental factors accounted for over a quarter of the burden, highlighting the urgent need for labor protection and occupational health services (**Figures 4A,B**, appendix pp 175-179). These findings indicate that high BMI is a significant driver of LBP burden in high and high-middle SDI regions, while occupational and environmental factors are more prominent in middle and low-middle SDI regions. Tailored interventions focusing on metabolic health and occupational health are needed to address the burden of LBP in postmenopausal women.

### Analysis of the global burden of LBP in postmenopausal women across different age groups and regions from 1990 to 2021

**Figure 5:**
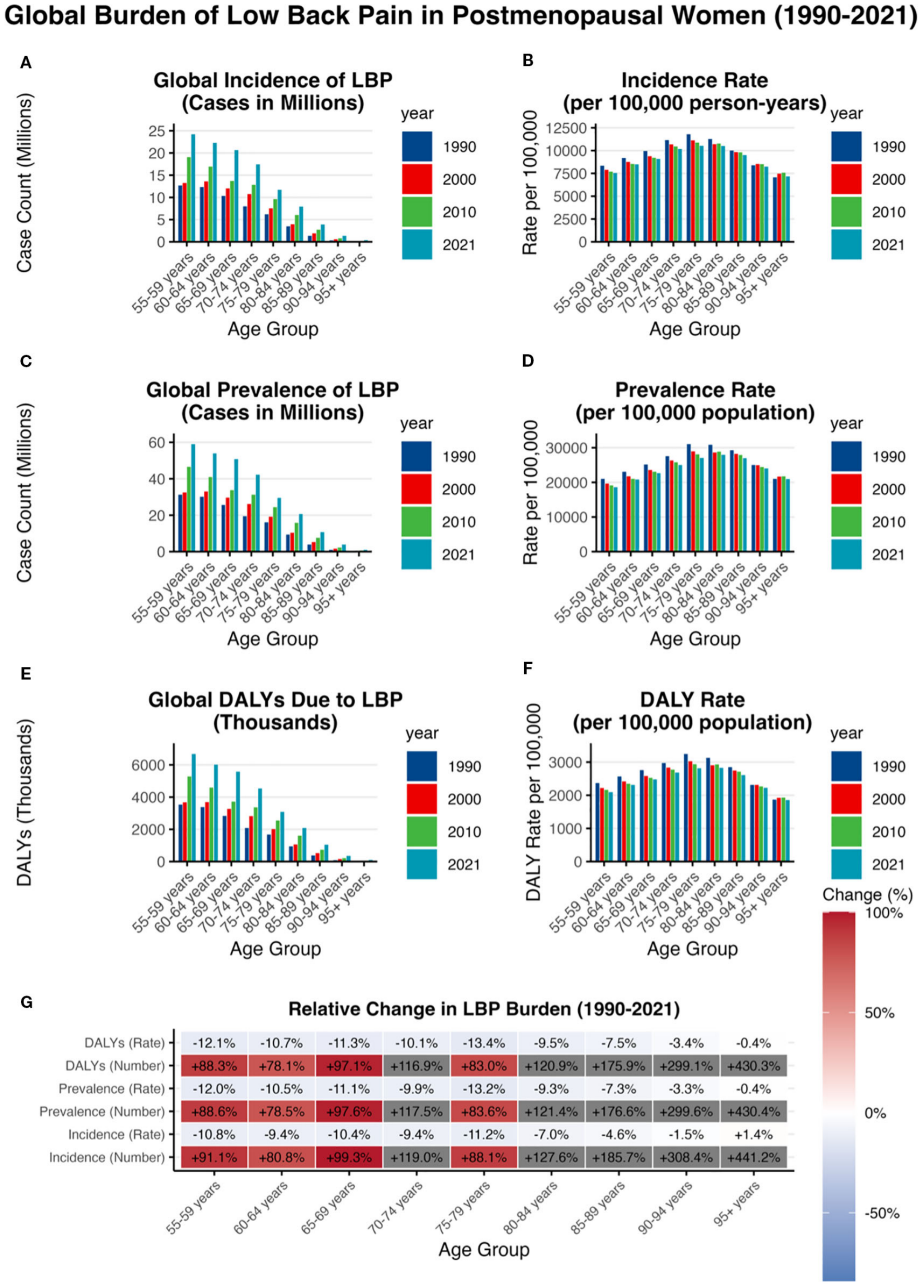
Analysis of the global burden of LBP in postmenopausal women across different age groups and regions from 1990 to 2021 **(A)** incidence case; **(B)** incidence rate; **(C)** prevalence case; **(D)** prevalence rate; **(E)** DALYs case; **(F)** DALY rate; **(G)** relative change.

In 2021, the greatest absolute burden was observed in women aged 55–59 years, with 15.2 million new cases (95% UI 10.8–20.3), 37.4 million prevalent cases (27.5–49.7), and 4.2 million DALYs (2.6–6.2) (**Figures 5A, C, E**). By contrast, the highest incidence rate occurred in women aged 75–79 years (10,533.8 per 100,000 person-years; 95% UI 7,861.9–13,713.6) (**Figure 5B**), while the highest age-standardized prevalence was in those aged 80–84 years (27,970.7 per 100,000; 21,366.2–35,617.5) (**Figure 5D**). The DALY rate also peaked at 80–84 years (2,825.9 per 100,000; 1,865.7–4,051.9) (**Figure 5F**). From 1990 to 2021, age-standardized incidence, prevalence, and DALY rates declined across all age groups, but absolute counts increased steadily with population growth and ageing (**Figure 5G**; appendix pp 179–257). These findings highlight a dual peak in disease burden—early postmenopause (55–59 years) and advanced age (80–84 years)—underscoring the need for targeted prevention and tailored management strategies in these high-risk groups.

## Discussion

LBP is a leading cause of disability globally, with a particularly pronounced burden among postmenopausal women. Postmenopausal women experience a decline in estrogen levels (27, 28), which leads to osteoporosis and intervertebral disc degeneration. They also face multiple risk factors, including central obesity, sarcopenia, sleep disorders, and psychological issues (29, 30). These factors not only increase the incidence of LBP but also worsen its severity. This study used data from the GBD 2021 to conduct a comprehensive analysis of the burden of LBP in postmenopausal women at the global, regional, and national levels from 1990 to 2021, offering important insights into the epidemiological trends and associated risk factors of this common musculoskeletal disorder.

### Global trends and their impacts

From 1990 to 2021, the global incidence, prevalence, and DALYs of LBP in postmenopausal women rose substantially, largely driven by population growth and ageing. New cases increased from 35·2 million (95% UI 29.3–41.2) to 70.3 million (58.6–81.7), prevalent cases from 89·9 million (76.1–105.1) to 176.8 million (150.4–205.6), and DALYs from 9.8 million (6.7–13.2) to 19.1 million (13.2–25.6). Despite these absolute increases, age-standardized incidence, prevalence, and DALY rates declined modestly, suggesting partial gains in disease management (20).These trends highlight the combined impact of demographic change and persistent exposure to risk factors, posing ongoing challenges to health systems, particularly in resource-limited settings.

### Gender differences and their impact

The burden of LBP in postmenopausal women remains significantly higher than in age-matched men, primarily due to the decline in estrogen levels after menopause. This hormonal change reduces bone mineral density, increases the risk of osteoporosis and intervertebral disc degeneration, and consequently elevates the incidence of spinal fractures (31, 32). Postmenopausal women also face multiple risk factors, including central obesity, sarcopenia, sleep disorders, and psychological problems, which further aggravate LBP. For instance, redistribution of body fat toward central or visceral depots increases lumbar loading and predisposes women to pain (33). Sarcopenia, characterized by the loss of muscle mass, not only compromises spinal stability but also accelerates osteoporosis progression (34, 35). Hormonal changes also impair sleep quality (36), thereby increasing susceptibility to chronic pain (37). Moreover, psychological distress is strongly associated with the occurrence and persistence of LBP (38). Therefore, interventions for postmenopausal women must comprehensively address these gender-specific biological and psychosocial factors. Importantly, among these contributors, hormonal decline plays a central mechanistic role. This biological perspective provides the foundation for a deeper exploration of hormonal mechanisms.

### Hormonal mechanisms of LBP in postmenopausal women

A critical biological driver of LBP in postmenopausal women is the decline in estrogen levels. Estrogen is essential for maintaining homeostasis in both bone and intervertebral discs. Following menopause, estrogen deficiency accelerates trabecular bone loss, promotes osteocyte apoptosis, and predisposes women to osteoporosis and vertebral fractures (39, 40). Estrogen receptors (ERα/β) are expressed in disc tissues and regulate genes such as CCN5, which protect disc cells against degeneration (41). Estrogen deficiency alters bone microarchitecture by reducing trabecular thickness and connectivity while increasing marrow fat infiltration. Together, these changes weaken skeletal strength (42, 43).

Estrogen also protects intervertebral discs by maintaining proteoglycan synthesis and collagen turnover. Its deficiency leads to reduced hydration and disc height, accelerating degeneration. Experimental studies confirm that estrogen replacement can attenuate extracellular matrix degradation by downregulating metalloproteinases (MMP-3, MMP-13) and enhancing the expression of proteoglycans and Col2α1 (44). These findings strongly support the role of estrogen deficiency in promoting disc degeneration (45).

Beyond structural effects, estrogen regulates systemic inflammation and immune responses. Postmenopausal estrogen depletion upregulates pro-inflammatory cytokines (e.g., IL-1β, IL-6, TNF-α), contributing to spinal inflammation and heightened nociception (46, 47). Estrogen receptor signaling exerts protective effects: ERα inhibits TLR4 pathways, while ERβ downregulates NF-κB activity, both suppressing inflammatory responses (48, 49). Estrogen deficiency also disrupts immune balance, increasing NK, Th1, and Th17 cells while reducing Treg proportions, thereby promoting chronic inflammation (46). Moreover, estrogen loss accelerates senescence of mesenchymal stem cells and elevates pro-inflammatory cytokine secretion, which further exacerbates osteoporosis (50, 51).

Recognition of these mechanisms underscores the importance of preventive strategies, including early hormonal and metabolic assessment, osteoporosis screening, and lifestyle interventions. Timely identification and management of hormonal changes can reduce the risk of severe LBP and improve long-term quality of life in postmenopausal women (52).

### Regional differences

High and high-middle SDI regions, with better medical infrastructure, have seen a downward trend in ASIR and DALY rates, likely due to better healthcare access and quality (53). In contrast, middle and low-middle SDI regions, undergoing rapid industrialization and urbanization, have seen increases in incidence and DALY rates, possibly due to lifestyle changes and occupational hazards (54, 55). Low SDI regions, with limited access to modern healthcare and heavy physical labor, face significant challenges in managing LBP (56). In many low- and middle-income countries (LMICs), occupational exposures remain major contributors to the burden of LBP. A systematic review and meta-analysis reported high prevalence of LBP among agricultural workers—who make up much of the workforce in LMICs—driven by repetitive movements, heavy lifting, and other high-risk tasks (57). Evidence from Iran shows that agricultural and rice industry work are significant risk factors for LBP in working populations, particularly among older workers (defined as those over 35 years of age) (58). Findings from India further support this pattern: a cross-sectional survey among rice farmers in West Bengal identified high LBP prevalence linked to ergonomic and psychosocial stressors such as prolonged stooping and manual load handling (59), while research among tea-plantation pluckers in Tamil Nadu reported a high burden of musculoskeletal disorders, with lower back symptoms strongly associated with repetitive plucking and limited job rotation (60). Acknowledging these sector-specific exposures enriches interpretation and underscores the need for targeted occupational health interventions in resource-limited settings. Targeted public health interventions are needed in regions with increasing disease burden. These findings underscore the need for tailored interventions addressing the specific needs of postmenopausal women, particularly in regions with limited resources and high disease burden.

### The burden of LBP in postmenopausal women at the national level

In 2021, China, India, and the United States had the highest number of new cases and DALYs of LBP in postmenopausal women. High incidence rates in these countries may be due to large populations, accelerated aging, and lifestyle factors. Occupational factors and high BMI are prominent in China (61), while high BMI, sedentary lifestyles, and occupational hazards are notable in the United States (23, 62). LBP is a leading cause of disability in the United States and imposes a significant economic burden on the healthcare system (63). Age-standardized incidence rates (ASIR) and DALY rates (ASDR) in 2021 were highest in Ukraine, New Zealand, and Poland, and lowest in Ecuador, Peru, and Myanmar. From 1990 to 2021, ASIR and ASDR showed varied trends globally, with increases in the United Kingdom and decreases in Denmark. These differences may relate to population structure, lifestyle, occupational environment, and public health policies. Countries should develop targeted public health strategies to reduce the disease burden, prioritizing improvements in working conditions, occupational health screening, and healthy lifestyle promotion. Given high BMI as a significant risk factor, weight management and health education are crucial (23, 61–63).

### Risk factor analysis

This study highlights three key modifiable GBD risk factors for LBP in postmenopausal women: high BMI, smoking, and environmental/occupational risks. High BMI is the most significant, accounting for 14.2% of global DALYs in 2021, emphasizing the need for weight management initiatives. Environmental and occupational risks, including ergonomic stress and occupational hazards, account for 12.6% of DALYs, particularly in middle and low-middle SDI regions undergoing industrialization and urbanization. Interventions targeting workplace ergonomics and occupational health policies are crucial in these regions. Smoking, although contributing less (7.3% of DALYs), remains an important modifiable risk factor, especially in high SDI regions with higher smoking rates.

### Major risk factors of LBP in postmenopausal women in different SDI regions

#### 1)High SDI and high-middle SDI regions

- High BMI: The primary risk factor, accounting for 16.4% of DALYs in high SDI regions and 17.5% in high-middle SDI regions. Obesity significantly increases the incidence and severity of LBP through increased physical burden and altered biomechanics (64, 65). It also affects treatment outcomes, with obese patients experiencing inferior postoperative and conservative treatment results (66, 67). Psychological factors such as Kinesio phobia and pain catastrophizing further exacerbate symptoms (68). Weight management and lifestyle improvements are essential for prevention and management, and future research should explore the complex relationship between obesity and LBP (23).

- Environmental/Occupational Risks: Account for 6.2% of DALYs in high SDI regions and 9.9% in high-middle SDI regions.

- Smoking: Accounts for 12.2% of DALYs in high SDI regions and 7.3% in high-middle SDI regions.

##### Representative region analysis

- High-Income North America (e.g., United States, Canada): High BMI is the leading risk factor, with metabolic interventions such as weight management and nutritional policies being a priority.

- East Asia (e.g., China, Japan): Environmental/occupational risks account for 20.1% of DALYs (95% UI 12.4–29.8). According to a study on the burden of LBP in China, occupational ergonomic factors are one of the main risk factors for LBP, accounting for 42.2% of all disability-adjusted life years (DALYs) due to LBP (69). Labor protection, occupational health screening, and community-level interventions should be prioritized, while monitoring the increasing burden related to high BMI.

#### 2)Middle SDI, low-middle SDI, and low SDI regions

- Environmental/Occupational Risks: These factors account for 16.1% of DALYs in middle SDI regions, 16.9% in low-middle SDI regions, and 25.6% in low SDI regions. Physical exposures such as prolonged standing, long working hours, and insufficient rest are major risk factors (70–72). Occupational biomechanical factors are significant contributors to LBP in these regions (73, 74).

- High BMI: Accounts for 12.8% of DALYs in middle SDI regions, 10.0% in low-middle SDI regions, and 7.2% in low SDI regions.

- Smoking: Accounts for 4.7% of DALYs in middle SDI regions, 4.7% in low-middle SDI regions, and 4.4% in low SDI regions.

##### Representative region analysis

- Southeast Asia (e.g., Thailand, Indonesia) and South Asia (e.g., India, Bangladesh): Labor protection policies, occupational health screening, and community-level interventions should be prioritized, while monitoring the potential increase in high BMI-related burden.

- Sub-Saharan Africa (e.g., Kenya, Ethiopia): Occupational and environmental factors account for nearly 40% of the total burden, highlighting the need for occupational health interventions and ergonomic improvements in agricultural and manual labor settings (75).

##### Age patterns and high-risk groups of LBP in postmenopausal women

This study identified two key age groups with the highest burden of LBP: the 55–59 years and 80–84 years groups. The 55–59 age group had the highest number of new cases, prevalence, and DALYs, while the 80–84 age group had the highest incidence, prevalence, and DALY rates.

###### 1)55–59 age group

The high burden in the 55–59 age group is attributed to early menopause and declining estrogen levels, which accelerate bone loss and intervertebral disc degeneration (76, 77). Estrogen deficiency leads to reduced trabecular bone mass and increased osteocyte apoptosis, exacerbating bone loss. Postmenopausal women show accelerated disc degeneration, narrower disc spaces, higher incidence of spondylolisthesis and arthritis, and a higher rate of osteoporosis-related spinal fractures (31). Women in this age group often face multiple social and family roles, such as work stress and family care, which can lead to fatigue and anxiety, further exacerbating the burden of LBP (78–80). Multidisciplinary interventions, including psychological support and health education, can help improve overall health and quality of life (81).

###### 2)80–84 age group

The high incidence, prevalence, and DALY rates in the 80–84 age group are likely due to the cumulative effects of aging and multiple chronic diseases, including osteoporosis, sarcopenia, and chronic degenerative changes in the spine (82–84). These age-related changes exacerbate mechanical stress on the lumbar spine, leading to persistent pain and disability. Comorbidities such as obesity, diabetes, and cardiovascular diseases further complicate the management of LBP (85–87). The coexistence of chronic pain with multiple chronic diseases increases the complexity of LBP (88–90). Comprehensive health assessment and multidisciplinary treatment are crucial for managing LBP in this population (91–93).

These findings highlight the need for targeted interventions and comprehensive health assessments to manage LBP in postmenopausal women, particularly in high-risk age groups.

### Strengths and limitations

The strength of this study lies in the use of comprehensive 2021 GBD data, providing a detailed view of the global burden of LBP in postmenopausal women. By analyzing data from different SDI regions and age groups, this study is able to provide targeted public health strategy recommendations. Additionally, this study assessed the contribution of major risk factors to the burden of LBP, providing a scientific basis for future interventions. However, this study also has limitations. The quality and availability of GBD data vary across regions, especially in low- and middle-income countries. Moreover, this study relies on aggregated data, which may not fully reflect changes at the individual level. Importantly, because the GBD dataset does not include direct information on menopausal status, we approximated postmenopausal women as those aged 55 years or older. While this operational definition has been applied in prior large-scale epidemiological studies, it may underestimate women who experience early menopause. Future studies should focus on collecting high-quality individual data, including detailed reproductive histories, to improve the accuracy of burden estimates and provide stronger support for targeted interventions.

## Conclusion

The global burden of LBP in postmenopausal women significantly increased between 1990 and 2021, although age-standardized rates declined. There are significant differences in risk factors across different SDI regions, indicating the need for targeted public health strategies tailored to regional characteristics. Future work should focus on improving data quality, expanding the evidence base for the effectiveness of prevention and treatment strategies, and prioritizing interventions for high-risk age groups and regions. In particular, there is a need to focus on early postmenopausal and elderly women to reduce the impact of LBP on their health and quality of life. Moreover, as the population ages, LBP in elderly women may become more prominent, requiring more research and interventions to address this challenge.

### Clinical and public health significance

The findings of this study are significant for clinical practice and public health policy. From a clinical perspective, the high burden of LBP in postmenopausal women highlights the necessity of using a multidisciplinary approach for management, including non-pharmacological interventions such as physical therapy, weight management, and smoking cessation programs. Although pharmacological interventions are effective in managing acute pain, they should be used cautiously to avoid long-term adverse effects and dependence. From a public health perspective, this study emphasizes the need for targeted interventions in regions with increasing disease burden. Middle SDI and low-middle SDI regions particularly need to strengthen medical infrastructure and public health campaigns to address modifiable risk factors related to LBP. Moreover, the high burden in specific age groups (55–59 and 80–84 years) indicates the need for age-targeted prevention strategies, including early screening for osteoporosis, ergonomic interventions, and lifestyle changes.

## Data Availability

The datasets presented in this study can be found in online repositories. The names of the repository/repositories and accession number(s) can be found in the article/**Supplementary Material**.

## Glossary

ASIR: Age-standardized incidence rate (ASIR) is a measure of the number of new cases of low back pain (LBP) per 100,000 individuals, adjusted for age distribution to allow for accurate comparisons across different regions and time periods
ASPR: Age-standardized prevalence rate (ASPR) is a measure of the number of existing cases of LBP per 100,000 individuals, adjusted for age distribution to facilitate comparisons between regions and over time
ASDR: Age-standardized disability-adjusted life years (DALY) rate (ASDR) is a measure of the average number of DALYs per 100,000 individuals, adjusted for age distribution
DALY: Disability-adjusted life years (DALYs) are a composite measure of overall disease burden, expressed as the sum of years lived with disability (YLDs) and years of life lost due to premature mortality (YLLs)
EAPC: Estimated annual percentage change (EAPC) is a measure used to describe the average annual rate of change in a given metric over a specified period, calculated using the formula EAPC = (exp(β) - 1) ×100%, where β
GBD: The Global Burden of Disease (GBD) Study is a comprehensive effort to quantify the health loss attributable to diseases, injuries, and risk factors globally
HAQ: Healthcare access and quality (HAQ) indices are composite measures that reflect the performance of healthcare systems in terms of access to care and the quality of care provided
ICD-10: International Classification of Diseases, 10th Revision (ICD-10) is a system of codes used to classify diseases and related health conditions
LBP: Low back pain (LBP) is a common musculoskeletal disorder characterized by pain in the lumbar region, with or without radiating pain to the lower limbs
M54.5: ICD-10 code for low back pain
PAR: Population-attributable risk (PAR) is a measure used to estimate the proportion of disease burden in a population that can be attributed to a specific risk factor
SDI: Socio-demographic Index (SDI) is a composite measure that reflects the level of development of a region based on factors such as per capita income, educational attainment, and fertility rates
UI: Uncertainty intervals (UI) are ranges that provide an estimate of the precision of a given metric, typically representing the 95% confidence intervals around the point estimate
YLD: Years lived with disability (YLDs) are a measure of the number of years lost due to disability from a specific disease or condition
YLL: Years of life lost (YLLs) are a measure of the number of years lost due to premature mortality from a specific disease or condition
High BMI: High body mass index (High BMI) is defined as a BMI ≥25 kg/m^2^for adults aged 20 and above, which is identified as a significant risk factor for various health conditions, including LBP
