# Supplementary material for: Global, regional, and national burden of low back pain in postmenopausal women from 1990 to 2021: a comprehensive analysis using data from the Global Burden of Disease Study 2021
Source: Front Endocrinol (Lausanne). 2025 Sep 26;16:1683183. doi: 10.3389/fendo.2025.1683183 (PMC12510828; doi:10.3389/fendo.2025.1683183)
Supplement: Supplementary file 1 [file DataSheet1.docx]

**Appendix 1: The Incidence, Prevalence, and DALYs of low back pain among postmenopausal women globally from 1990 to 2021**

| measure | location | sex | age | cause | metric | year | val | upper | lower |
| --- | --- | --- | --- | --- | --- | --- | --- | --- | --- |
| DALYs (Disability-Adjusted Life Years) | Global | Female | 55+ years | Low back pain | Number | 1990 | 9763112.14 | 13185106.8 | 6744495.33 |
| Prevalence | Global | Female | 55+ years | Low back pain | Number | 1990 | 89891793.4 | 105083987 | 76116025.7 |
| Incidence | Global | Female | 55+ years | Low back pain | Number | 1990 | 35161994.9 | 41189119.9 | 29292850.2 |
| DALYs (Disability-Adjusted Life Years) | Global | Female | 55+ years | Low back pain | Number | 1991 | 9860497.67 | 13303158.7 | 6810687.5 |
| Prevalence | Global | Female | 55+ years | Low back pain | Number | 1991 | 90786148.7 | 106186669 | 76865670.2 |
| Incidence | Global | Female | 55+ years | Low back pain | Number | 1991 | 35583435.3 | 41642834.7 | 29657469 |
| DALYs (Disability-Adjusted Life Years) | Global | Female | 55+ years | Low back pain | Number | 1992 | 9964201.39 | 13453079.1 | 6875757.51 |
| Prevalence | Global | Female | 55+ years | Low back pain | Number | 1992 | 91742949.3 | 107236594 | 77646388.8 |
| Incidence | Global | Female | 55+ years | Low back pain | Number | 1992 | 36030150.3 | 42135606.2 | 30029290.5 |
| DALYs (Disability-Adjusted Life Years) | Global | Female | 55+ years | Low back pain | Number | 1993 | 10078027.1 | 13599643 | 6943960.71 |
| Prevalence | Global | Female | 55+ years | Low back pain | Number | 1993 | 92793734.1 | 108384689 | 78478833.2 |
| Incidence | Global | Female | 55+ years | Low back pain | Number | 1993 | 36507219.1 | 42664805.6 | 30422967.9 |
| DALYs (Disability-Adjusted Life Years) | Global | Female | 55+ years | Low back pain | Number | 1994 | 10206213.2 | 13756366.2 | 7022969.54 |
| Prevalence | Global | Female | 55+ years | Low back pain | Number | 1994 | 93989782.3 | 109690162 | 79435821.7 |
| Incidence | Global | Female | 55+ years | Low back pain | Number | 1994 | 37024927.5 | 43255726.3 | 30856621.1 |
| DALYs (Disability-Adjusted Life Years) | Global | Female | 55+ years | Low back pain | Number | 1995 | 10365657.7 | 13974746.7 | 7125991.66 |
| Prevalence | Global | Female | 55+ years | Low back pain | Number | 1995 | 95468929.9 | 111413401 | 80630418.6 |
| Incidence | Global | Female | 55+ years | Low back pain | Number | 1995 | 37633036.6 | 43976671 | 31374865.9 |
| DALYs (Disability-Adjusted Life Years) | Global | Female | 55+ years | Low back pain | Number | 1996 | 10532608.8 | 14166893.9 | 7252356.06 |
| Prevalence | Global | Female | 55+ years | Low back pain | Number | 1996 | 97037209.2 | 113084887 | 82028862.5 |
| Incidence | Global | Female | 55+ years | Low back pain | Number | 1996 | 38262685.6 | 44643092.9 | 31926274.5 |
| DALYs (Disability-Adjusted Life Years) | Global | Female | 55+ years | Low back pain | Number | 1997 | 10685992.5 | 14354717.5 | 7362186.91 |
| Prevalence | Global | Female | 55+ years | Low back pain | Number | 1997 | 98484904.5 | 114604420 | 83372617.4 |
| Incidence | Global | Female | 55+ years | Low back pain | Number | 1997 | 38844480.2 | 45262936.6 | 32437063.9 |
| DALYs (Disability-Adjusted Life Years) | Global | Female | 55+ years | Low back pain | Number | 1998 | 10827662.2 | 14534216.7 | 7467918.94 |
| Prevalence | Global | Female | 55+ years | Low back pain | Number | 1998 | 99836846.1 | 115988879 | 84645535.1 |
| Incidence | Global | Female | 55+ years | Low back pain | Number | 1998 | 39387085.2 | 45837527.9 | 32918413.3 |
| DALYs (Disability-Adjusted Life Years) | Global | Female | 55+ years | Low back pain | Number | 1999 | 10972830.7 | 14700718.5 | 7580233.91 |
| Prevalence | Global | Female | 55+ years | Low back pain | Number | 1999 | 101231651 | 117421259 | 85925932.9 |
| Incidence | Global | Female | 55+ years | Low back pain | Number | 1999 | 39945059.9 | 46447013 | 33412292.4 |
| DALYs (Disability-Adjusted Life Years) | Global | Female | 55+ years | Low back pain | Number | 2000 | 11133613 | 14878895.2 | 7699111.4 |
| Prevalence | Global | Female | 55+ years | Low back pain | Number | 2000 | 102757164 | 119168475 | 87338759.9 |
| Incidence | Global | Female | 55+ years | Low back pain | Number | 2000 | 40556678.2 | 47127053.7 | 33959091.1 |
| DALYs (Disability-Adjusted Life Years) | Global | Female | 55+ years | Low back pain | Number | 2001 | 11333215.9 | 15144678.8 | 7842318.39 |
| Prevalence | Global | Female | 55+ years | Low back pain | Number | 2001 | 104640002 | 121308026 | 88967539.5 |
| Incidence | Global | Female | 55+ years | Low back pain | Number | 2001 | 41317594.2 | 48036217.4 | 34601040 |
| DALYs (Disability-Adjusted Life Years) | Global | Female | 55+ years | Low back pain | Number | 2002 | 11592177.5 | 15483569.4 | 8031434.97 |
| Prevalence | Global | Female | 55+ years | Low back pain | Number | 2002 | 107038342 | 124000053 | 91074744.9 |
| Incidence | Global | Female | 55+ years | Low back pain | Number | 2002 | 42291193.7 | 49195046.4 | 35419481.8 |
| DALYs (Disability-Adjusted Life Years) | Global | Female | 55+ years | Low back pain | Number | 2003 | 11855542.1 | 15843751.9 | 8226025.93 |
| Prevalence | Global | Female | 55+ years | Low back pain | Number | 2003 | 109479390 | 126733567 | 93201516.1 |
| Incidence | Global | Female | 55+ years | Low back pain | Number | 2003 | 43284869.6 | 50398844 | 36252717.8 |
| DALYs (Disability-Adjusted Life Years) | Global | Female | 55+ years | Low back pain | Number | 2004 | 12154844.2 | 16238676 | 8430905.96 |
| Prevalence | Global | Female | 55+ years | Low back pain | Number | 2004 | 112241238 | 129836609 | 95604332 |
| Incidence | Global | Female | 55+ years | Low back pain | Number | 2004 | 44404520.3 | 51773928.1 | 37160938 |
| DALYs (Disability-Adjusted Life Years) | Global | Female | 55+ years | Low back pain | Number | 2005 | 12440126.1 | 16659258.2 | 8643274.98 |
| Prevalence | Global | Female | 55+ years | Low back pain | Number | 2005 | 114881211 | 132791524 | 97969298.6 |
| Incidence | Global | Female | 55+ years | Low back pain | Number | 2005 | 45463320.2 | 53062581 | 38077601.2 |
| DALYs (Disability-Adjusted Life Years) | Global | Female | 55+ years | Low back pain | Number | 2006 | 12770518.1 | 17101859.2 | 8859052.95 |
| Prevalence | Global | Female | 55+ years | Low back pain | Number | 2006 | 117927748 | 136329183 | 100681286 |
| Incidence | Global | Female | 55+ years | Low back pain | Number | 2006 | 46673410 | 54512485.1 | 39079038.5 |
| DALYs (Disability-Adjusted Life Years) | Global | Female | 55+ years | Low back pain | Number | 2007 | 13113432.3 | 17593849.7 | 9091847.43 |
| Prevalence | Global | Female | 55+ years | Low back pain | Number | 2007 | 121089861 | 140150720 | 103458525 |
| Incidence | Global | Female | 55+ years | Low back pain | Number | 2007 | 47918854.2 | 56004141.8 | 40086957.9 |
| DALYs (Disability-Adjusted Life Years) | Global | Female | 55+ years | Low back pain | Number | 2008 | 13468338.1 | 18077788.6 | 9356506.49 |
| Prevalence | Global | Female | 55+ years | Low back pain | Number | 2008 | 124359867 | 143933204 | 106331577 |
| Incidence | Global | Female | 55+ years | Low back pain | Number | 2008 | 49206576.3 | 57550050.7 | 41127071.3 |
| DALYs (Disability-Adjusted Life Years) | Global | Female | 55+ years | Low back pain | Number | 2009 | 13842480.1 | 18605809.8 | 9617859.92 |
| Prevalence | Global | Female | 55+ years | Low back pain | Number | 2009 | 127813287 | 147908853 | 109342207 |
| Incidence | Global | Female | 55+ years | Low back pain | Number | 2009 | 50574902.4 | 59208960.3 | 42225657.6 |
| DALYs (Disability-Adjusted Life Years) | Global | Female | 55+ years | Low back pain | Number | 2010 | 14234818.6 | 19133380.5 | 9893008.94 |
| Prevalence | Global | Female | 55+ years | Low back pain | Number | 2010 | 131433663 | 152072600 | 112521575 |
| Incidence | Global | Female | 55+ years | Low back pain | Number | 2010 | 52024648.5 | 60948144 | 43402607.8 |
| DALYs (Disability-Adjusted Life Years) | Global | Female | 55+ years | Low back pain | Number | 2011 | 14643339.8 | 19686931.8 | 10178013.3 |
| Prevalence | Global | Female | 55+ years | Low back pain | Number | 2011 | 135201887 | 156353926 | 115722384 |
| Incidence | Global | Female | 55+ years | Low back pain | Number | 2011 | 53545451.6 | 62742964.4 | 44673535.8 |
| DALYs (Disability-Adjusted Life Years) | Global | Female | 55+ years | Low back pain | Number | 2012 | 15062583.4 | 20261361.4 | 10474006 |
| Prevalence | Global | Female | 55+ years | Low back pain | Number | 2012 | 139074792 | 160858657 | 118958447 |
| Incidence | Global | Female | 55+ years | Low back pain | Number | 2012 | 55109172.5 | 64582787.7 | 45981853 |
| DALYs (Disability-Adjusted Life Years) | Global | Female | 55+ years | Low back pain | Number | 2013 | 15483102.4 | 20839687.7 | 10762193.8 |
| Prevalence | Global | Female | 55+ years | Low back pain | Number | 2013 | 142972552 | 165422042 | 122192730 |
| Incidence | Global | Female | 55+ years | Low back pain | Number | 2013 | 56683600.8 | 66445684.7 | 47275596.7 |
| DALYs (Disability-Adjusted Life Years) | Global | Female | 55+ years | Low back pain | Number | 2014 | 15909328.4 | 21423226 | 11057370.1 |
| Prevalence | Global | Female | 55+ years | Low back pain | Number | 2014 | 146928417 | 170158717 | 125440178 |
| Incidence | Global | Female | 55+ years | Low back pain | Number | 2014 | 58277263.3 | 68229879.5 | 48595556.8 |
| DALYs (Disability-Adjusted Life Years) | Global | Female | 55+ years | Low back pain | Number | 2015 | 16340940.9 | 22017512.7 | 11350353.9 |
| Prevalence | Global | Female | 55+ years | Low back pain | Number | 2015 | 150935440 | 174931735 | 128743904 |
| Incidence | Global | Female | 55+ years | Low back pain | Number | 2015 | 59891762.6 | 70035478.2 | 49935930.7 |
| DALYs (Disability-Adjusted Life Years) | Global | Female | 55+ years | Low back pain | Number | 2016 | 16779787.7 | 22593477 | 11643737 |
| Prevalence | Global | Female | 55+ years | Low back pain | Number | 2016 | 155017714 | 179908976 | 132088032 |
| Incidence | Global | Female | 55+ years | Low back pain | Number | 2016 | 61529928.4 | 71810614.4 | 51308724.4 |
| DALYs (Disability-Adjusted Life Years) | Global | Female | 55+ years | Low back pain | Number | 2017 | 17232948.6 | 23175360.4 | 11937353.6 |
| Prevalence | Global | Female | 55+ years | Low back pain | Number | 2017 | 159235513 | 185076451 | 135598068 |
| Incidence | Global | Female | 55+ years | Low back pain | Number | 2017 | 63227104.5 | 73695607.3 | 52700086.7 |
| DALYs (Disability-Adjusted Life Years) | Global | Female | 55+ years | Low back pain | Number | 2018 | 17708723.1 | 23787434.9 | 12271487 |
| Prevalence | Global | Female | 55+ years | Low back pain | Number | 2018 | 163661305 | 190267025 | 139324735 |
| Incidence | Global | Female | 55+ years | Low back pain | Number | 2018 | 65007529.7 | 75688563.7 | 54225299.9 |
| DALYs (Disability-Adjusted Life Years) | Global | Female | 55+ years | Low back pain | Number | 2019 | 18206875.7 | 24408880.4 | 12598984.1 |
| Prevalence | Global | Female | 55+ years | Low back pain | Number | 2019 | 168299658 | 195622507 | 143230465 |
| Incidence | Global | Female | 55+ years | Low back pain | Number | 2019 | 66870302.6 | 77834605.2 | 55829486 |
| DALYs (Disability-Adjusted Life Years) | Global | Female | 55+ years | Low back pain | Number | 2020 | 18616524.1 | 24994716.2 | 12903541.8 |
| Prevalence | Global | Female | 55+ years | Low back pain | Number | 2020 | 172320139 | 200341228 | 146447045 |
| Incidence | Global | Female | 55+ years | Low back pain | Number | 2020 | 68422705.6 | 79623057.6 | 57079532.2 |
| DALYs (Disability-Adjusted Life Years) | Global | Female | 55+ years | Low back pain | Number | 2021 | 19078108.2 | 25575222.4 | 13229420.8 |
| Prevalence | Global | Female | 55+ years | Low back pain | Number | 2021 | 176831236 | 205614417 | 150407719 |
| Incidence | Global | Female | 55+ years | Low back pain | Number | 2021 | 70273888.3 | 81718501.4 | 58553412.9 |

**Appendix 2: The age-standardized incidence, prevalence, and DALY rates of low back pain among postmenopausal women globally from 1990 to 2021**

| **measure** | **location** | **sex** | **age** | **cause** | **metric** | **year** | **val** | **upper** | **lower** |
| --- | --- | --- | --- | --- | --- | --- | --- | --- | --- |
| **DALYs (Disability-Adjusted Life Years)** | **Global** | **Female** | **Age-standardized** | **Low back pain** | **Rate** | **1990** | **443.831859** | **599.396009** | **306.605297** |
| **Prevalence** | **Global** | **Female** | **Age-standardized** | **Low back pain** | **Rate** | **1990** | **4086.48812** | **4777.12644** | **3460.24062** |
| **Incidence** | **Global** | **Female** | **Age-standardized** | **Low back pain** | **Rate** | **1990** | **1598.4671** | **1872.46068** | **1331.65531** |
| **DALYs (Disability-Adjusted Life Years)** | **Global** | **Female** | **Age-standardized** | **Low back pain** | **Rate** | **1991** | **438.915653** | **592.15719** | **303.1609** |
| **Prevalence** | **Global** | **Female** | **Age-standardized** | **Low back pain** | **Rate** | **1991** | **4041.12075** | **4726.63681** | **3421.4851** |
| **Incidence** | **Global** | **Female** | **Age-standardized** | **Low back pain** | **Rate** | **1991** | **1583.90857** | **1853.62774** | **1320.12884** |
| **DALYs (Disability-Adjusted Life Years)** | **Global** | **Female** | **Age-standardized** | **Low back pain** | **Rate** | **1992** | **434.354835** | **586.440371** | **299.724825** |
| **Prevalence** | **Global** | **Female** | **Age-standardized** | **Low back pain** | **Rate** | **1992** | **3999.21599** | **4674.60776** | **3384.72528** |
| **Incidence** | **Global** | **Female** | **Age-standardized** | **Low back pain** | **Rate** | **1992** | **1570.60956** | **1836.75576** | **1309.02287** |
| **DALYs (Disability-Adjusted Life Years)** | **Global** | **Female** | **Age-standardized** | **Low back pain** | **Rate** | **1993** | **430.521831** | **580.96125** | **296.638088** |
| **Prevalence** | **Global** | **Female** | **Age-standardized** | **Low back pain** | **Rate** | **1993** | **3964.04257** | **4630.07037** | **3352.52631** |
| **Incidence** | **Global** | **Female** | **Age-standardized** | **Low back pain** | **Rate** | **1993** | **1559.54679** | **1822.59187** | **1299.63452** |
| **DALYs (Disability-Adjusted Life Years)** | **Global** | **Female** | **Age-standardized** | **Low back pain** | **Rate** | **1994** | **427.743103** | **576.530257** | **294.333139** |
| **Prevalence** | **Global** | **Female** | **Age-standardized** | **Low back pain** | **Rate** | **1994** | **3939.11827** | **4597.12225** | **3329.16078** |
| **Incidence** | **Global** | **Female** | **Age-standardized** | **Low back pain** | **Rate** | **1994** | **1551.71727** | **1812.85048** | **1293.20312** |
| **DALYs (Disability-Adjusted Life Years)** | **Global** | **Female** | **Age-standardized** | **Low back pain** | **Rate** | **1995** | **426.2936** | **574.719448** | **293.060482** |
| **Prevalence** | **Global** | **Female** | **Age-standardized** | **Low back pain** | **Rate** | **1995** | **3926.21433** | **4581.93982** | **3315.97207** |
| **Incidence** | **Global** | **Female** | **Age-standardized** | **Low back pain** | **Rate** | **1995** | **1547.68015** | **1808.56574** | **1290.3093** |
| **DALYs (Disability-Adjusted Life Years)** | **Global** | **Female** | **Age-standardized** | **Low back pain** | **Rate** | **1996** | **425.260684** | **571.997225** | **292.818423** |
| **Prevalence** | **Global** | **Female** | **Age-standardized** | **Low back pain** | **Rate** | **1996** | **3917.93816** | **4565.8732** | **3311.96676** |
| **Incidence** | **Global** | **Female** | **Age-standardized** | **Low back pain** | **Rate** | **1996** | **1544.87992** | **1802.49287** | **1289.04335** |
| **DALYs (Disability-Adjusted Life Years)** | **Global** | **Female** | **Age-standardized** | **Low back pain** | **Rate** | **1997** | **423.825323** | **569.333434** | **291.997328** |
| **Prevalence** | **Global** | **Female** | **Age-standardized** | **Low back pain** | **Rate** | **1997** | **3906.08515** | **4545.41357** | **3306.70517** |
| **Incidence** | **Global** | **Female** | **Age-standardized** | **Low back pain** | **Rate** | **1997** | **1540.64065** | **1795.20796** | **1286.51121** |
| **DALYs (Disability-Adjusted Life Years)** | **Global** | **Female** | **Age-standardized** | **Low back pain** | **Rate** | **1998** | **422.168869** | **566.686854** | **291.173001** |
| **Prevalence** | **Global** | **Female** | **Age-standardized** | **Low back pain** | **Rate** | **1998** | **3892.62314** | **4522.38838** | **3300.31629** |
| **Incidence** | **Global** | **Female** | **Age-standardized** | **Low back pain** | **Rate** | **1998** | **1535.69634** | **1787.1981** | **1283.48383** |
| **DALYs (Disability-Adjusted Life Years)** | **Global** | **Female** | **Age-standardized** | **Low back pain** | **Rate** | **1999** | **420.641357** | **563.549217** | **290.586809** |
| **Prevalence** | **Global** | **Female** | **Age-standardized** | **Low back pain** | **Rate** | **1999** | **3880.69588** | **4501.32137** | **3293.95411** |
| **Incidence** | **Global** | **Female** | **Age-standardized** | **Low back pain** | **Rate** | **1999** | **1531.28619** | **1780.53731** | **1280.8538** |
| **DALYs (Disability-Adjusted Life Years)** | **Global** | **Female** | **Age-standardized** | **Low back pain** | **Rate** | **2000** | **419.6679** | **560.841724** | **290.20857** |
| **Prevalence** | **Global** | **Female** | **Age-standardized** | **Low back pain** | **Rate** | **2000** | **3873.30539** | **4491.90964** | **3292.12753** |
| **Incidence** | **Global** | **Female** | **Age-standardized** | **Low back pain** | **Rate** | **2000** | **1528.73429** | **1776.39654** | **1280.04633** |
| **DALYs (Disability-Adjusted Life Years)** | **Global** | **Female** | **Age-standardized** | **Low back pain** | **Rate** | **2001** | **419.029399** | **559.952774** | **289.958473** |
| **Prevalence** | **Global** | **Female** | **Age-standardized** | **Low back pain** | **Rate** | **2001** | **3868.91398** | **4485.19024** | **3289.44715** |
| **Incidence** | **Global** | **Female** | **Age-standardized** | **Low back pain** | **Rate** | **2001** | **1527.65878** | **1776.07023** | **1279.32382** |
| **DALYs (Disability-Adjusted Life Years)** | **Global** | **Female** | **Age-standardized** | **Low back pain** | **Rate** | **2002** | **418.401635** | **558.855379** | **289.882165** |
| **Prevalence** | **Global** | **Female** | **Age-standardized** | **Low back pain** | **Rate** | **2002** | **3863.38263** | **4475.58924** | **3287.2014** |
| **Incidence** | **Global** | **Female** | **Age-standardized** | **Low back pain** | **Rate** | **2002** | **1526.43492** | **1775.61876** | **1278.41116** |
| **DALYs (Disability-Adjusted Life Years)** | **Global** | **Female** | **Age-standardized** | **Low back pain** | **Rate** | **2003** | **417.749521** | **558.280654** | **289.857551** |
| **Prevalence** | **Global** | **Female** | **Age-standardized** | **Low back pain** | **Rate** | **2003** | **3857.68633** | **4465.66564** | **3284.10869** |
| **Incidence** | **Global** | **Female** | **Age-standardized** | **Low back pain** | **Rate** | **2003** | **1525.21356** | **1775.88615** | **1277.42413** |
| **DALYs (Disability-Adjusted Life Years)** | **Global** | **Female** | **Age-standardized** | **Low back pain** | **Rate** | **2004** | **416.674823** | **556.670848** | **289.016147** |
| **Prevalence** | **Global** | **Female** | **Age-standardized** | **Low back pain** | **Rate** | **2004** | **3847.69211** | **4450.87121** | **3277.3697** |
| **Incidence** | **Global** | **Female** | **Age-standardized** | **Low back pain** | **Rate** | **2004** | **1522.21166** | **1774.83906** | **1273.89763** |
| **DALYs (Disability-Adjusted Life Years)** | **Global** | **Female** | **Age-standardized** | **Low back pain** | **Rate** | **2005** | **415.80409** | **556.826165** | **288.896517** |
| **Prevalence** | **Global** | **Female** | **Age-standardized** | **Low back pain** | **Rate** | **2005** | **3839.8387** | **4438.48065** | **3274.56771** |
| **Incidence** | **Global** | **Female** | **Age-standardized** | **Low back pain** | **Rate** | **2005** | **1519.58544** | **1773.58638** | **1272.72202** |
| **DALYs (Disability-Adjusted Life Years)** | **Global** | **Female** | **Age-standardized** | **Low back pain** | **Rate** | **2006** | **414.409223** | **554.963245** | **287.480368** |
| **Prevalence** | **Global** | **Female** | **Age-standardized** | **Low back pain** | **Rate** | **2006** | **3826.80999** | **4423.94507** | **3267.15432** |
| **Incidence** | **Global** | **Female** | **Age-standardized** | **Low back pain** | **Rate** | **2006** | **1514.57376** | **1768.95537** | **1268.13289** |
| **DALYs (Disability-Adjusted Life Years)** | **Global** | **Female** | **Age-standardized** | **Low back pain** | **Rate** | **2007** | **412.94944** | **554.04033** | **286.307445** |
| **Prevalence** | **Global** | **Female** | **Age-standardized** | **Low back pain** | **Rate** | **2007** | **3813.18859** | **4413.42587** | **3257.96777** |
| **Incidence** | **Global** | **Female** | **Age-standardized** | **Low back pain** | **Rate** | **2007** | **1508.99197** | **1763.60227** | **1262.3611** |
| **DALYs (Disability-Adjusted Life Years)** | **Global** | **Female** | **Age-standardized** | **Low back pain** | **Rate** | **2008** | **411.306591** | **552.073578** | **285.736277** |
| **Prevalence** | **Global** | **Female** | **Age-standardized** | **Low back pain** | **Rate** | **2008** | **3797.7984** | **4395.54419** | **3247.23643** |
| **Incidence** | **Global** | **Female** | **Age-standardized** | **Low back pain** | **Rate** | **2008** | **1502.70872** | **1757.50824** | **1255.97051** |
| **DALYs (Disability-Adjusted Life Years)** | **Global** | **Female** | **Age-standardized** | **Low back pain** | **Rate** | **2009** | **409.681207** | **550.656427** | **284.649603** |
| **Prevalence** | **Global** | **Female** | **Age-standardized** | **Low back pain** | **Rate** | **2009** | **3782.75434** | **4377.50151** | **3236.08537** |
| **Incidence** | **Global** | **Female** | **Age-standardized** | **Low back pain** | **Rate** | **2009** | **1496.81177** | **1752.34482** | **1249.70802** |
| **DALYs (Disability-Adjusted Life Years)** | **Global** | **Female** | **Age-standardized** | **Low back pain** | **Rate** | **2010** | **408.115238** | **548.558035** | **283.634643** |
| **Prevalence** | **Global** | **Female** | **Age-standardized** | **Low back pain** | **Rate** | **2010** | **3768.2307** | **4359.95335** | **3226.01719** |
| **Incidence** | **Global** | **Female** | **Age-standardized** | **Low back pain** | **Rate** | **2010** | **1491.55759** | **1747.39608** | **1244.36188** |
| **DALYs (Disability-Adjusted Life Years)** | **Global** | **Female** | **Age-standardized** | **Low back pain** | **Rate** | **2011** | **407.092695** | **547.307257** | **282.954226** |
| **Prevalence** | **Global** | **Female** | **Age-standardized** | **Low back pain** | **Rate** | **2011** | **3758.68493** | **4346.72295** | **3217.14429** |
| **Incidence** | **Global** | **Female** | **Age-standardized** | **Low back pain** | **Rate** | **2011** | **1488.59225** | **1744.28804** | **1241.94824** |
| **DALYs (Disability-Adjusted Life Years)** | **Global** | **Female** | **Age-standardized** | **Low back pain** | **Rate** | **2012** | **406.556809** | **546.877929** | **282.705718** |
| **Prevalence** | **Global** | **Female** | **Age-standardized** | **Low back pain** | **Rate** | **2012** | **3753.7919** | **4341.76398** | **3210.82813** |
| **Incidence** | **Global** | **Female** | **Age-standardized** | **Low back pain** | **Rate** | **2012** | **1487.46126** | **1743.16526** | **1241.10419** |
| **DALYs (Disability-Adjusted Life Years)** | **Global** | **Female** | **Age-standardized** | **Low back pain** | **Rate** | **2013** | **406.241788** | **546.78654** | **282.375763** |
| **Prevalence** | **Global** | **Female** | **Age-standardized** | **Low back pain** | **Rate** | **2013** | **3751.27825** | **4340.30239** | **3206.06245** |
| **Incidence** | **Global** | **Female** | **Age-standardized** | **Low back pain** | **Rate** | **2013** | **1487.25022** | **1743.38534** | **1240.40535** |
| **DALYs (Disability-Adjusted Life Years)** | **Global** | **Female** | **Age-standardized** | **Low back pain** | **Rate** | **2014** | **406.023179** | **546.743776** | **282.195982** |
| **Prevalence** | **Global** | **Female** | **Age-standardized** | **Low back pain** | **Rate** | **2014** | **3749.77129** | **4342.63353** | **3201.36832** |
| **Incidence** | **Global** | **Female** | **Age-standardized** | **Low back pain** | **Rate** | **2014** | **1487.29846** | **1741.29994** | **1240.21089** |
| **DALYs (Disability-Adjusted Life Years)** | **Global** | **Female** | **Age-standardized** | **Low back pain** | **Rate** | **2015** | **405.546713** | **546.426915** | **281.691169** |
| **Prevalence** | **Global** | **Female** | **Age-standardized** | **Low back pain** | **Rate** | **2015** | **3745.89027** | **4341.42627** | **3195.14448** |
| **Incidence** | **Global** | **Female** | **Age-standardized** | **Low back pain** | **Rate** | **2015** | **1486.38366** | **1738.12868** | **1239.3015** |
| **DALYs (Disability-Adjusted Life Years)** | **Global** | **Female** | **Age-standardized** | **Low back pain** | **Rate** | **2016** | **404.730841** | **544.957844** | **280.848576** |
| **Prevalence** | **Global** | **Female** | **Age-standardized** | **Low back pain** | **Rate** | **2016** | **3739.04907** | **4339.42981** | **3185.98192** |
| **Incidence** | **Global** | **Female** | **Age-standardized** | **Low back pain** | **Rate** | **2016** | **1484.11053** | **1732.08212** | **1237.57365** |
| **DALYs (Disability-Adjusted Life Years)** | **Global** | **Female** | **Age-standardized** | **Low back pain** | **Rate** | **2017** | **403.600726** | **542.773757** | **279.576334** |
| **Prevalence** | **Global** | **Female** | **Age-standardized** | **Low back pain** | **Rate** | **2017** | **3729.34255** | **4334.54493** | **3175.74664** |
| **Incidence** | **Global** | **Female** | **Age-standardized** | **Low back pain** | **Rate** | **2017** | **1480.79739** | **1725.9728** | **1234.25153** |
| **DALYs (Disability-Adjusted Life Years)** | **Global** | **Female** | **Age-standardized** | **Low back pain** | **Rate** | **2018** | **402.173083** | **540.223368** | **278.691** |
| **Prevalence** | **Global** | **Female** | **Age-standardized** | **Low back pain** | **Rate** | **2018** | **3716.82199** | **4321.0499** | **3164.12754** |
| **Incidence** | **Global** | **Female** | **Age-standardized** | **Low back pain** | **Rate** | **2018** | **1476.3503** | **1718.9214** | **1231.481** |
| **DALYs (Disability-Adjusted Life Years)** | **Global** | **Female** | **Age-standardized** | **Low back pain** | **Rate** | **2019** | **400.834117** | **537.374572** | **277.373382** |
| **Prevalence** | **Global** | **Female** | **Age-standardized** | **Low back pain** | **Rate** | **2019** | **3705.2071** | **4306.73424** | **3153.29539** |
| **Incidence** | **Global** | **Female** | **Age-standardized** | **Low back pain** | **Rate** | **2019** | **1472.18552** | **1713.57051** | **1229.11603** |
| **DALYs (Disability-Adjusted Life Years)** | **Global** | **Female** | **Age-standardized** | **Low back pain** | **Rate** | **2020** | **397.868321** | **534.181662** | **275.771701** |
| **Prevalence** | **Global** | **Female** | **Age-standardized** | **Low back pain** | **Rate** | **2020** | **3682.78869** | **4281.64933** | **3129.83452** |
| **Incidence** | **Global** | **Female** | **Age-standardized** | **Low back pain** | **Rate** | **2020** | **1462.31524** | **1701.68674** | **1219.8914** |
| **DALYs (Disability-Adjusted Life Years)** | **Global** | **Female** | **Age-standardized** | **Low back pain** | **Rate** | **2021** | **396.932837** | **532.109655** | **275.246973** |
| **Prevalence** | **Global** | **Female** | **Age-standardized** | **Low back pain** | **Rate** | **2021** | **3679.09246** | **4277.94585** | **3129.33348** |
| **Incidence** | **Global** | **Female** | **Age-standardized** | **Low back pain** | **Rate** | **2021** | **1462.09538** | **1700.20823** | **1218.24303** |

**Appendix 3: The incidence, prevalence, and DALYs of low back pain among postmenopausal women and age-matched men globally from 1990 to 2021**

| **measure** | **location** | **sex** | **age** | **cause** | **metric** | **year** | **val** | **upper** | **lower** |
| --- | --- | --- | --- | --- | --- | --- | --- | --- | --- |
| **DALYs (Disability-Adjusted Life Years)** | **Global** | **Male** | **55+ years** | **Low back pain** | **Number** | **1990** | **5193579.23** | **7133546.25** | **3526648.83** |
| **DALYs (Disability-Adjusted Life Years)** | **Global** | **Female** | **55+ years** | **Low back pain** | **Number** | **1990** | **9763112.14** | **13185106.8** | **6744495.33** |
| **DALYs (Disability-Adjusted Life Years)** | **Global** | **Both** | **55+ years** | **Low back pain** | **Number** | **1990** | **14956691.4** | **20401083.9** | **10228044.8** |
| **Prevalence** | **Global** | **Female** | **55+ years** | **Low back pain** | **Number** | **1990** | **89891793.4** | **105083987** | **76116025.7** |
| **Incidence** | **Global** | **Female** | **55+ years** | **Low back pain** | **Number** | **1990** | **35161994.9** | **41189119.9** | **29292850.2** |
| **Prevalence** | **Global** | **Male** | **55+ years** | **Low back pain** | **Number** | **1990** | **47114636.1** | **55863686.4** | **38668400.2** |
| **Prevalence** | **Global** | **Both** | **55+ years** | **Low back pain** | **Number** | **1990** | **137006429** | **160963564** | **114867792** |
| **Incidence** | **Global** | **Male** | **55+ years** | **Low back pain** | **Number** | **1990** | **19631913** | **23366912.6** | **16122483.9** |
| **Incidence** | **Global** | **Both** | **55+ years** | **Low back pain** | **Number** | **1990** | **54793907.9** | **64649618.5** | **45557317** |
| **DALYs (Disability-Adjusted Life Years)** | **Global** | **Female** | **55+ years** | **Low back pain** | **Number** | **1991** | **9860497.67** | **13303158.7** | **6810687.5** |
| **Prevalence** | **Global** | **Female** | **55+ years** | **Low back pain** | **Number** | **1991** | **90786148.7** | **106186669** | **76865670.2** |
| **Incidence** | **Global** | **Female** | **55+ years** | **Low back pain** | **Number** | **1991** | **35583435.3** | **41642834.7** | **29657469** |
| **DALYs (Disability-Adjusted Life Years)** | **Global** | **Male** | **55+ years** | **Low back pain** | **Number** | **1991** | **5279846.29** | **7249853.47** | **3588907.99** |
| **DALYs (Disability-Adjusted Life Years)** | **Global** | **Both** | **55+ years** | **Low back pain** | **Number** | **1991** | **15140344** | **20610885.1** | **10363782.3** |
| **Prevalence** | **Global** | **Male** | **55+ years** | **Low back pain** | **Number** | **1991** | **47898092.5** | **56749528.1** | **39368466.7** |
| **Prevalence** | **Global** | **Both** | **55+ years** | **Low back pain** | **Number** | **1991** | **138684241** | **162993881** | **116318595** |
| **Incidence** | **Global** | **Male** | **55+ years** | **Low back pain** | **Number** | **1991** | **19983585.1** | **23786776.9** | **16422935.2** |
| **Incidence** | **Global** | **Both** | **55+ years** | **Low back pain** | **Number** | **1991** | **55567020.4** | **65500262.7** | **46264787.6** |
| **DALYs (Disability-Adjusted Life Years)** | **Global** | **Male** | **55+ years** | **Low back pain** | **Number** | **1992** | **5368070.2** | **7366342.83** | **3649817.05** |
| **DALYs (Disability-Adjusted Life Years)** | **Global** | **Both** | **55+ years** | **Low back pain** | **Number** | **1992** | **15332271.6** | **20856559.3** | **10482237.4** |
| **DALYs (Disability-Adjusted Life Years)** | **Global** | **Female** | **55+ years** | **Low back pain** | **Number** | **1992** | **9964201.39** | **13453079.1** | **6875757.51** |
| **Prevalence** | **Global** | **Female** | **55+ years** | **Low back pain** | **Number** | **1992** | **91742949.3** | **107236594** | **77646388.8** |
| **Incidence** | **Global** | **Female** | **55+ years** | **Low back pain** | **Number** | **1992** | **36030150.3** | **42135606.2** | **30029290.5** |
| **Prevalence** | **Global** | **Male** | **55+ years** | **Low back pain** | **Number** | **1992** | **48696676** | **57659386** | **40089994.1** |
| **Prevalence** | **Global** | **Both** | **55+ years** | **Low back pain** | **Number** | **1992** | **140439625** | **165199234** | **117831416** |
| **Incidence** | **Global** | **Male** | **55+ years** | **Low back pain** | **Number** | **1992** | **20339746.6** | **24187926.5** | **16726899.2** |
| **Incidence** | **Global** | **Both** | **55+ years** | **Low back pain** | **Number** | **1992** | **56369896.9** | **66390345.3** | **46961228.7** |
| **DALYs (Disability-Adjusted Life Years)** | **Global** | **Male** | **55+ years** | **Low back pain** | **Number** | **1993** | **5457220.68** | **7487358.52** | **3712580.61** |
| **DALYs (Disability-Adjusted Life Years)** | **Global** | **Both** | **55+ years** | **Low back pain** | **Number** | **1993** | **15535247.8** | **21123563.8** | **10622728.4** |
| **DALYs (Disability-Adjusted Life Years)** | **Global** | **Female** | **55+ years** | **Low back pain** | **Number** | **1993** | **10078027.1** | **13599643** | **6943960.71** |
| **Prevalence** | **Global** | **Female** | **55+ years** | **Low back pain** | **Number** | **1993** | **92793734.1** | **108384689** | **78478833.2** |
| **Incidence** | **Global** | **Female** | **55+ years** | **Low back pain** | **Number** | **1993** | **36507219.1** | **42664805.6** | **30422967.9** |
| **Prevalence** | **Global** | **Male** | **55+ years** | **Low back pain** | **Number** | **1993** | **49509121.9** | **58596864.6** | **40851728.7** |
| **Prevalence** | **Global** | **Both** | **55+ years** | **Low back pain** | **Number** | **1993** | **142302856** | **167490060** | **119427023** |
| **Incidence** | **Global** | **Male** | **55+ years** | **Low back pain** | **Number** | **1993** | **20697432.1** | **24595525.4** | **17024255.4** |
| **Incidence** | **Global** | **Both** | **55+ years** | **Low back pain** | **Number** | **1993** | **57204651.3** | **67319533.7** | **47677118.5** |
| **DALYs (Disability-Adjusted Life Years)** | **Global** | **Male** | **55+ years** | **Low back pain** | **Number** | **1994** | **5548252.15** | **7610055.21** | **3777065.97** |
| **DALYs (Disability-Adjusted Life Years)** | **Global** | **Both** | **55+ years** | **Low back pain** | **Number** | **1994** | **15754465.4** | **21383939.4** | **10782729.7** |
| **DALYs (Disability-Adjusted Life Years)** | **Global** | **Female** | **55+ years** | **Low back pain** | **Number** | **1994** | **10206213.2** | **13756366.2** | **7022969.54** |
| **Prevalence** | **Global** | **Female** | **55+ years** | **Low back pain** | **Number** | **1994** | **93989782.3** | **109690162** | **79435821.7** |
| **Incidence** | **Global** | **Female** | **55+ years** | **Low back pain** | **Number** | **1994** | **37024927.5** | **43255726.3** | **30856621.1** |
| **Prevalence** | **Global** | **Male** | **55+ years** | **Low back pain** | **Number** | **1994** | **50345011.5** | **59563209.2** | **41625814.6** |
| **Prevalence** | **Global** | **Both** | **55+ years** | **Low back pain** | **Number** | **1994** | **144334794** | **169683016** | **121158423** |
| **Incidence** | **Global** | **Male** | **55+ years** | **Low back pain** | **Number** | **1994** | **21057966.2** | **25016334.7** | **17321571.9** |
| **Incidence** | **Global** | **Both** | **55+ years** | **Low back pain** | **Number** | **1994** | **58082893.7** | **68311655.6** | **48400862.8** |
| **DALYs (Disability-Adjusted Life Years)** | **Global** | **Male** | **55+ years** | **Low back pain** | **Number** | **1995** | **5648435.85** | **7745625** | **3844802.83** |
| **DALYs (Disability-Adjusted Life Years)** | **Global** | **Both** | **55+ years** | **Low back pain** | **Number** | **1995** | **16014093.6** | **21731653.9** | **10959134.2** |
| **DALYs (Disability-Adjusted Life Years)** | **Global** | **Female** | **55+ years** | **Low back pain** | **Number** | **1995** | **10365657.7** | **13974746.7** | **7125991.66** |
| **Prevalence** | **Global** | **Female** | **55+ years** | **Low back pain** | **Number** | **1995** | **95468929.9** | **111413401** | **80630418.6** |
| **Incidence** | **Global** | **Female** | **55+ years** | **Low back pain** | **Number** | **1995** | **37633036.6** | **43976671** | **31374865.9** |
| **Prevalence** | **Global** | **Male** | **55+ years** | **Low back pain** | **Number** | **1995** | **51267509.9** | **60673148.5** | **42452727.7** |
| **Prevalence** | **Global** | **Both** | **55+ years** | **Low back pain** | **Number** | **1995** | **146736440** | **172399953** | **123179961** |
| **Incidence** | **Global** | **Male** | **55+ years** | **Low back pain** | **Number** | **1995** | **21446093.6** | **25468600.5** | **17655589.7** |
| **Incidence** | **Global** | **Both** | **55+ years** | **Low back pain** | **Number** | **1995** | **59079130.2** | **69464860.9** | **49217082.1** |
| **DALYs (Disability-Adjusted Life Years)** | **Global** | **Male** | **55+ years** | **Low back pain** | **Number** | **1996** | **5747857.62** | **7852731.18** | **3920631.33** |
| **DALYs (Disability-Adjusted Life Years)** | **Global** | **Both** | **55+ years** | **Low back pain** | **Number** | **1996** | **16280466.4** | **22073191.2** | **11155366.8** |
| **DALYs (Disability-Adjusted Life Years)** | **Global** | **Female** | **55+ years** | **Low back pain** | **Number** | **1996** | **10532608.8** | **14166893.9** | **7252356.06** |
| **Prevalence** | **Global** | **Female** | **55+ years** | **Low back pain** | **Number** | **1996** | **97037209.2** | **113084887** | **82028862.5** |
| **Incidence** | **Global** | **Female** | **55+ years** | **Low back pain** | **Number** | **1996** | **38262685.6** | **44643092.9** | **31926274.5** |
| **Prevalence** | **Global** | **Male** | **55+ years** | **Low back pain** | **Number** | **1996** | **52187884.3** | **61652937.1** | **43290144.2** |
| **Prevalence** | **Global** | **Both** | **55+ years** | **Low back pain** | **Number** | **1996** | **149225093** | **175006741** | **125439990** |
| **Incidence** | **Global** | **Male** | **55+ years** | **Low back pain** | **Number** | **1996** | **21824907.6** | **25905208.3** | **18005097.4** |
| **Incidence** | **Global** | **Both** | **55+ years** | **Low back pain** | **Number** | **1996** | **60087593.2** | **70560966.8** | **50075834.2** |
| **DALYs (Disability-Adjusted Life Years)** | **Global** | **Male** | **55+ years** | **Low back pain** | **Number** | **1997** | **5834798.01** | **7963904.19** | **3982397** |
| **DALYs (Disability-Adjusted Life Years)** | **Global** | **Both** | **55+ years** | **Low back pain** | **Number** | **1997** | **16520790.5** | **22352198.7** | **11345641.6** |
| **DALYs (Disability-Adjusted Life Years)** | **Global** | **Female** | **55+ years** | **Low back pain** | **Number** | **1997** | **10685992.5** | **14354717.5** | **7362186.91** |
| **Prevalence** | **Global** | **Female** | **55+ years** | **Low back pain** | **Number** | **1997** | **98484904.5** | **114604420** | **83372617.4** |
| **Incidence** | **Global** | **Female** | **55+ years** | **Low back pain** | **Number** | **1997** | **38844480.2** | **45262936.6** | **32437063.9** |
| **Prevalence** | **Global** | **Male** | **55+ years** | **Low back pain** | **Number** | **1997** | **52999179.1** | **62503210.3** | **44028419.8** |
| **Prevalence** | **Global** | **Both** | **55+ years** | **Low back pain** | **Number** | **1997** | **151484084** | **177396752** | **127605124** |
| **Incidence** | **Global** | **Male** | **55+ years** | **Low back pain** | **Number** | **1997** | **22151350.7** | **26264535.9** | **18286006.1** |
| **Incidence** | **Global** | **Both** | **55+ years** | **Low back pain** | **Number** | **1997** | **60995830.9** | **71548991.5** | **50847435** |
| **DALYs (Disability-Adjusted Life Years)** | **Global** | **Male** | **55+ years** | **Low back pain** | **Number** | **1998** | **5915660.85** | **8070246.97** | **4043792.29** |
| **DALYs (Disability-Adjusted Life Years)** | **Global** | **Both** | **55+ years** | **Low back pain** | **Number** | **1998** | **16743323** | **22640995.1** | **11512083.2** |
| **DALYs (Disability-Adjusted Life Years)** | **Global** | **Female** | **55+ years** | **Low back pain** | **Number** | **1998** | **10827662.2** | **14534216.7** | **7467918.94** |
| **Prevalence** | **Global** | **Female** | **55+ years** | **Low back pain** | **Number** | **1998** | **99836846.1** | **115988879** | **84645535.1** |
| **Incidence** | **Global** | **Female** | **55+ years** | **Low back pain** | **Number** | **1998** | **39387085.2** | **45837527.9** | **32918413.3** |
| **Prevalence** | **Global** | **Male** | **55+ years** | **Low back pain** | **Number** | **1998** | **53768576.7** | **63313492.7** | **44734857.3** |
| **Prevalence** | **Global** | **Both** | **55+ years** | **Low back pain** | **Number** | **1998** | **153605423** | **179632109** | **129678581** |
| **Incidence** | **Global** | **Male** | **55+ years** | **Low back pain** | **Number** | **1998** | **22454532.8** | **26588000.7** | **18556103.1** |
| **Incidence** | **Global** | **Both** | **55+ years** | **Low back pain** | **Number** | **1998** | **61841618** | **72462546.4** | **51531468.2** |
| **DALYs (Disability-Adjusted Life Years)** | **Global** | **Male** | **55+ years** | **Low back pain** | **Number** | **1999** | **5998290** | **8171781.04** | **4100594.26** |
| **DALYs (Disability-Adjusted Life Years)** | **Global** | **Both** | **55+ years** | **Low back pain** | **Number** | **1999** | **16971120.7** | **22939729.8** | **11690656.8** |
| **DALYs (Disability-Adjusted Life Years)** | **Global** | **Female** | **55+ years** | **Low back pain** | **Number** | **1999** | **10972830.7** | **14700718.5** | **7580233.91** |
| **Prevalence** | **Global** | **Female** | **55+ years** | **Low back pain** | **Number** | **1999** | **101231651** | **117421259** | **85925932.9** |
| **Incidence** | **Global** | **Female** | **55+ years** | **Low back pain** | **Number** | **1999** | **39945059.9** | **46447013** | **33412292.4** |
| **Prevalence** | **Global** | **Male** | **55+ years** | **Low back pain** | **Number** | **1999** | **54553011** | **64223641.4** | **45450375.9** |
| **Prevalence** | **Global** | **Both** | **55+ years** | **Low back pain** | **Number** | **1999** | **155784662** | **181927378** | **131806608** |
| **Incidence** | **Global** | **Male** | **55+ years** | **Low back pain** | **Number** | **1999** | **22763019.1** | **26916076** | **18840627.2** |
| **Incidence** | **Global** | **Both** | **55+ years** | **Low back pain** | **Number** | **1999** | **62708079** | **73405192.4** | **52238083** |
| **DALYs (Disability-Adjusted Life Years)** | **Global** | **Male** | **55+ years** | **Low back pain** | **Number** | **2000** | **6090404.57** | **8291193.14** | **4174523.86** |
| **DALYs (Disability-Adjusted Life Years)** | **Global** | **Both** | **55+ years** | **Low back pain** | **Number** | **2000** | **17224017.6** | **23248434.3** | **11882099.7** |
| **DALYs (Disability-Adjusted Life Years)** | **Global** | **Female** | **55+ years** | **Low back pain** | **Number** | **2000** | **11133613** | **14878895.2** | **7699111.4** |
| **Prevalence** | **Global** | **Female** | **55+ years** | **Low back pain** | **Number** | **2000** | **102757164** | **119168475** | **87338759.9** |
| **Incidence** | **Global** | **Female** | **55+ years** | **Low back pain** | **Number** | **2000** | **40556678.2** | **47127053.7** | **33959091.1** |
| **Prevalence** | **Global** | **Male** | **55+ years** | **Low back pain** | **Number** | **2000** | **55421663.9** | **65157295.3** | **46242041.2** |
| **Prevalence** | **Global** | **Both** | **55+ years** | **Low back pain** | **Number** | **2000** | **158178828** | **184381054** | **133952729** |
| **Incidence** | **Global** | **Male** | **55+ years** | **Low back pain** | **Number** | **2000** | **23110777.9** | **27330306.9** | **19159839.8** |
| **Incidence** | **Global** | **Both** | **55+ years** | **Low back pain** | **Number** | **2000** | **63667456.1** | **74447435.6** | **53029290.9** |
| **DALYs (Disability-Adjusted Life Years)** | **Global** | **Male** | **55+ years** | **Low back pain** | **Number** | **2001** | **6204593.5** | **8448205.6** | **4250800.58** |
| **DALYs (Disability-Adjusted Life Years)** | **Global** | **Both** | **55+ years** | **Low back pain** | **Number** | **2001** | **17537809.4** | **23666951** | **12112446.3** |
| **DALYs (Disability-Adjusted Life Years)** | **Global** | **Female** | **55+ years** | **Low back pain** | **Number** | **2001** | **11333215.9** | **15144678.8** | **7842318.39** |
| **Prevalence** | **Global** | **Female** | **55+ years** | **Low back pain** | **Number** | **2001** | **104640002** | **121308026** | **88967539.5** |
| **Incidence** | **Global** | **Female** | **55+ years** | **Low back pain** | **Number** | **2001** | **41317594.2** | **48036217.4** | **34601040** |
| **Prevalence** | **Global** | **Male** | **55+ years** | **Low back pain** | **Number** | **2001** | **56480632** | **66280423.1** | **47181674.7** |
| **Prevalence** | **Global** | **Both** | **55+ years** | **Low back pain** | **Number** | **2001** | **161120634** | **187558337** | **136534346** |
| **Incidence** | **Global** | **Male** | **55+ years** | **Low back pain** | **Number** | **2001** | **23543227.2** | **27823916.8** | **19543610.1** |
| **Incidence** | **Global** | **Both** | **55+ years** | **Low back pain** | **Number** | **2001** | **64860821.5** | **75800202.7** | **54050878** |
| **DALYs (Disability-Adjusted Life Years)** | **Global** | **Male** | **55+ years** | **Low back pain** | **Number** | **2002** | **6355361.47** | **8634376.72** | **4349439.82** |
| **DALYs (Disability-Adjusted Life Years)** | **Global** | **Both** | **55+ years** | **Low back pain** | **Number** | **2002** | **17947539** | **24202274.4** | **12405692.8** |
| **DALYs (Disability-Adjusted Life Years)** | **Global** | **Female** | **55+ years** | **Low back pain** | **Number** | **2002** | **11592177.5** | **15483569.4** | **8031434.97** |
| **Prevalence** | **Global** | **Female** | **55+ years** | **Low back pain** | **Number** | **2002** | **107038342** | **124000053** | **91074744.9** |
| **Incidence** | **Global** | **Female** | **55+ years** | **Low back pain** | **Number** | **2002** | **42291193.7** | **49195046.4** | **35419481.8** |
| **Prevalence** | **Global** | **Male** | **55+ years** | **Low back pain** | **Number** | **2002** | **57853712.2** | **67838243.1** | **48384312.4** |
| **Prevalence** | **Global** | **Both** | **55+ years** | **Low back pain** | **Number** | **2002** | **164892054** | **191839184** | **139877933** |
| **Incidence** | **Global** | **Male** | **55+ years** | **Low back pain** | **Number** | **2002** | **24107262.8** | **28444261.3** | **20023351.9** |
| **Incidence** | **Global** | **Both** | **55+ years** | **Low back pain** | **Number** | **2002** | **66398456.5** | **77603206.4** | **55412973.7** |
| **DALYs (Disability-Adjusted Life Years)** | **Global** | **Male** | **55+ years** | **Low back pain** | **Number** | **2003** | **6509706.01** | **8849833.4** | **4458563.64** |
| **DALYs (Disability-Adjusted Life Years)** | **Global** | **Both** | **55+ years** | **Low back pain** | **Number** | **2003** | **18365248.1** | **24745194.5** | **12703888.5** |
| **DALYs (Disability-Adjusted Life Years)** | **Global** | **Female** | **55+ years** | **Low back pain** | **Number** | **2003** | **11855542.1** | **15843751.9** | **8226025.93** |
| **Prevalence** | **Global** | **Female** | **55+ years** | **Low back pain** | **Number** | **2003** | **109479390** | **126733567** | **93201516.1** |
| **Incidence** | **Global** | **Female** | **55+ years** | **Low back pain** | **Number** | **2003** | **43284869.6** | **50398844** | **36252717.8** |
| **Prevalence** | **Global** | **Male** | **55+ years** | **Low back pain** | **Number** | **2003** | **59251878** | **69414976.3** | **49603642.1** |
| **Prevalence** | **Global** | **Both** | **55+ years** | **Low back pain** | **Number** | **2003** | **168731268** | **196130639** | **143219717** |
| **Incidence** | **Global** | **Male** | **55+ years** | **Low back pain** | **Number** | **2003** | **24683568.8** | **29086148.8** | **20516097.9** |
| **Incidence** | **Global** | **Both** | **55+ years** | **Low back pain** | **Number** | **2003** | **67968438.4** | **79471533.3** | **56827892.3** |
| **DALYs (Disability-Adjusted Life Years)** | **Global** | **Male** | **55+ years** | **Low back pain** | **Number** | **2004** | **6682390.28** | **9073643.88** | **4575993.23** |
| **DALYs (Disability-Adjusted Life Years)** | **Global** | **Both** | **55+ years** | **Low back pain** | **Number** | **2004** | **18837234.5** | **25381439.9** | **13031784.8** |
| **DALYs (Disability-Adjusted Life Years)** | **Global** | **Female** | **55+ years** | **Low back pain** | **Number** | **2004** | **12154844.2** | **16238676** | **8430905.96** |
| **Prevalence** | **Global** | **Female** | **55+ years** | **Low back pain** | **Number** | **2004** | **112241238** | **129836609** | **95604332** |
| **Incidence** | **Global** | **Female** | **55+ years** | **Low back pain** | **Number** | **2004** | **44404520.3** | **51773928.1** | **37160938** |
| **Prevalence** | **Global** | **Male** | **55+ years** | **Low back pain** | **Number** | **2004** | **60817034.6** | **71123575.7** | **50949222.4** |
| **Prevalence** | **Global** | **Both** | **55+ years** | **Low back pain** | **Number** | **2004** | **173058273** | **200944286** | **146911302** |
| **Incidence** | **Global** | **Male** | **55+ years** | **Low back pain** | **Number** | **2004** | **25334170.7** | **29889044.7** | **21070294.2** |
| **Incidence** | **Global** | **Both** | **55+ years** | **Low back pain** | **Number** | **2004** | **69738691.1** | **81628495.1** | **58332376.1** |
| **DALYs (Disability-Adjusted Life Years)** | **Global** | **Male** | **55+ years** | **Low back pain** | **Number** | **2005** | **6844902.36** | **9287010.65** | **4685073.85** |
| **DALYs (Disability-Adjusted Life Years)** | **Global** | **Both** | **55+ years** | **Low back pain** | **Number** | **2005** | **19285028.4** | **25996058.8** | **13358316.1** |
| **DALYs (Disability-Adjusted Life Years)** | **Global** | **Female** | **55+ years** | **Low back pain** | **Number** | **2005** | **12440126.1** | **16659258.2** | **8643274.98** |
| **Prevalence** | **Global** | **Female** | **55+ years** | **Low back pain** | **Number** | **2005** | **114881211** | **132791524** | **97969298.6** |
| **Prevalence** | **Global** | **Male** | **55+ years** | **Low back pain** | **Number** | **2005** | **62291702.8** | **72738770.8** | **52224809.8** |
| **Incidence** | **Global** | **Female** | **55+ years** | **Low back pain** | **Number** | **2005** | **45463320.2** | **53062581** | **38077601.2** |
| **Prevalence** | **Global** | **Both** | **55+ years** | **Low back pain** | **Number** | **2005** | **177172914** | **205628661** | **150563794** |
| **Incidence** | **Global** | **Male** | **55+ years** | **Low back pain** | **Number** | **2005** | **25945467.3** | **30633066.7** | **21582808** |
| **Incidence** | **Global** | **Both** | **55+ years** | **Low back pain** | **Number** | **2005** | **71408787.5** | **83644856** | **59720781.6** |
| **DALYs (Disability-Adjusted Life Years)** | **Global** | **Male** | **55+ years** | **Low back pain** | **Number** | **2006** | **7035072.26** | **9562435.73** | **4814868.02** |
| **DALYs (Disability-Adjusted Life Years)** | **Global** | **Both** | **55+ years** | **Low back pain** | **Number** | **2006** | **19805590.4** | **26679374** | **13697117.9** |
| **Prevalence** | **Global** | **Male** | **55+ years** | **Low back pain** | **Number** | **2006** | **64013670.8** | **74661825.8** | **53686275.4** |
| **DALYs (Disability-Adjusted Life Years)** | **Global** | **Female** | **55+ years** | **Low back pain** | **Number** | **2006** | **12770518.1** | **17101859.2** | **8859052.95** |
| **Prevalence** | **Global** | **Both** | **55+ years** | **Low back pain** | **Number** | **2006** | **181941418** | **211153756** | **154695420** |
| **Prevalence** | **Global** | **Female** | **55+ years** | **Low back pain** | **Number** | **2006** | **117927748** | **136329183** | **100681286** |
| **Incidence** | **Global** | **Female** | **55+ years** | **Low back pain** | **Number** | **2006** | **46673410** | **54512485.1** | **39079038.5** |
| **Incidence** | **Global** | **Male** | **55+ years** | **Low back pain** | **Number** | **2006** | **26664953.2** | **31482829.7** | **22144084** |
| **Incidence** | **Global** | **Both** | **55+ years** | **Low back pain** | **Number** | **2006** | **73338363.2** | **85989522** | **61312071.9** |
| **DALYs (Disability-Adjusted Life Years)** | **Global** | **Male** | **55+ years** | **Low back pain** | **Number** | **2007** | **7238618.9** | **9843056.92** | **4960433.76** |
| **DALYs (Disability-Adjusted Life Years)** | **Global** | **Both** | **55+ years** | **Low back pain** | **Number** | **2007** | **20352051.2** | **27433080.2** | **14062705** |
| **Prevalence** | **Global** | **Male** | **55+ years** | **Low back pain** | **Number** | **2007** | **65851321.1** | **76755686.9** | **55245876.6** |
| **Prevalence** | **Global** | **Both** | **55+ years** | **Low back pain** | **Number** | **2007** | **186941182** | **216988212** | **158941052** |
| **DALYs (Disability-Adjusted Life Years)** | **Global** | **Female** | **55+ years** | **Low back pain** | **Number** | **2007** | **13113432.3** | **17593849.7** | **9091847.43** |
| **Prevalence** | **Global** | **Female** | **55+ years** | **Low back pain** | **Number** | **2007** | **121089861** | **140150720** | **103458525** |
| **Incidence** | **Global** | **Female** | **55+ years** | **Low back pain** | **Number** | **2007** | **47918854.2** | **56004141.8** | **40086957.9** |
| **Incidence** | **Global** | **Male** | **55+ years** | **Low back pain** | **Number** | **2007** | **27437452.8** | **32375908.4** | **22771281.1** |
| **Incidence** | **Global** | **Both** | **55+ years** | **Low back pain** | **Number** | **2007** | **75356307** | **88353226.1** | **62979277.7** |
| **DALYs (Disability-Adjusted Life Years)** | **Global** | **Male** | **55+ years** | **Low back pain** | **Number** | **2008** | **7448808.63** | **10139040.6** | **5099615.07** |
| **DALYs (Disability-Adjusted Life Years)** | **Global** | **Both** | **55+ years** | **Low back pain** | **Number** | **2008** | **20917146.8** | **28236159.5** | **14449650.2** |
| **Prevalence** | **Global** | **Male** | **55+ years** | **Low back pain** | **Number** | **2008** | **67756489.5** | **78953503.1** | **56859295.4** |
| **Prevalence** | **Global** | **Both** | **55+ years** | **Low back pain** | **Number** | **2008** | **192116356** | **223049053** | **163311768** |
| **DALYs (Disability-Adjusted Life Years)** | **Global** | **Female** | **55+ years** | **Low back pain** | **Number** | **2008** | **13468338.1** | **18077788.6** | **9356506.49** |
| **Prevalence** | **Global** | **Female** | **55+ years** | **Low back pain** | **Number** | **2008** | **124359867** | **143933204** | **106331577** |
| **Incidence** | **Global** | **Female** | **55+ years** | **Low back pain** | **Number** | **2008** | **49206576.3** | **57550050.7** | **41127071.3** |
| **Incidence** | **Global** | **Male** | **55+ years** | **Low back pain** | **Number** | **2008** | **28240698.5** | **33302640.5** | **23419603.3** |
| **Incidence** | **Global** | **Both** | **55+ years** | **Low back pain** | **Number** | **2008** | **77447274.7** | **90849027.9** | **64706237.2** |
| **DALYs (Disability-Adjusted Life Years)** | **Global** | **Male** | **55+ years** | **Low back pain** | **Number** | **2009** | **7670140.46** | **10434143.4** | **5250969.74** |
| **DALYs (Disability-Adjusted Life Years)** | **Global** | **Both** | **55+ years** | **Low back pain** | **Number** | **2009** | **21512620.5** | **29039175.5** | **14856063** |
| **Prevalence** | **Global** | **Male** | **55+ years** | **Low back pain** | **Number** | **2009** | **69753860.5** | **81288318.5** | **58536286.9** |
| **Prevalence** | **Global** | **Both** | **55+ years** | **Low back pain** | **Number** | **2009** | **197567147** | **229548129** | **167879930** |
| **DALYs (Disability-Adjusted Life Years)** | **Global** | **Female** | **55+ years** | **Low back pain** | **Number** | **2009** | **13842480.1** | **18605809.8** | **9617859.92** |
| **Prevalence** | **Global** | **Female** | **55+ years** | **Low back pain** | **Number** | **2009** | **127813287** | **147908853** | **109342207** |
| **Incidence** | **Global** | **Female** | **55+ years** | **Low back pain** | **Number** | **2009** | **50574902.4** | **59208960.3** | **42225657.6** |
| **Incidence** | **Global** | **Male** | **55+ years** | **Low back pain** | **Number** | **2009** | **29084674.9** | **34265555.8** | **24118936.2** |
| **Incidence** | **Global** | **Both** | **55+ years** | **Low back pain** | **Number** | **2009** | **79659577.3** | **93524814.9** | **66539932.3** |
| **DALYs (Disability-Adjusted Life Years)** | **Global** | **Male** | **55+ years** | **Low back pain** | **Number** | **2010** | **7895720.85** | **10754257.9** | **5403917.36** |
| **DALYs (Disability-Adjusted Life Years)** | **Global** | **Both** | **55+ years** | **Low back pain** | **Number** | **2010** | **22130539.5** | **29870508.4** | **15261151.2** |
| **Prevalence** | **Global** | **Male** | **55+ years** | **Low back pain** | **Number** | **2010** | **71796429.7** | **83642053.2** | **60255939.4** |
| **Prevalence** | **Global** | **Both** | **55+ years** | **Low back pain** | **Number** | **2010** | **203230093** | **236253402** | **172634390** |
| **DALYs (Disability-Adjusted Life Years)** | **Global** | **Female** | **55+ years** | **Low back pain** | **Number** | **2010** | **14234818.6** | **19133380.5** | **9893008.94** |
| **Prevalence** | **Global** | **Female** | **55+ years** | **Low back pain** | **Number** | **2010** | **131433663** | **152072600** | **112521575** |
| **Incidence** | **Global** | **Female** | **55+ years** | **Low back pain** | **Number** | **2010** | **52024648.5** | **60948144** | **43402607.8** |
| **Incidence** | **Global** | **Male** | **55+ years** | **Low back pain** | **Number** | **2010** | **29945787.3** | **35257566.7** | **24837238** |
| **Incidence** | **Global** | **Both** | **55+ years** | **Low back pain** | **Number** | **2010** | **81970435.8** | **96291606.7** | **68455843.8** |
| **DALYs (Disability-Adjusted Life Years)** | **Global** | **Male** | **55+ years** | **Low back pain** | **Number** | **2011** | **8124613.12** | **11064887.9** | **5557910.62** |
| **DALYs (Disability-Adjusted Life Years)** | **Global** | **Both** | **55+ years** | **Low back pain** | **Number** | **2011** | **22767952.9** | **30760665.3** | **15705256.7** |
| **Prevalence** | **Global** | **Male** | **55+ years** | **Low back pain** | **Number** | **2011** | **73870804.5** | **86008098.3** | **61991730.6** |
| **Prevalence** | **Global** | **Both** | **55+ years** | **Low back pain** | **Number** | **2011** | **209072692** | **243120313** | **177462829** |
| **DALYs (Disability-Adjusted Life Years)** | **Global** | **Female** | **55+ years** | **Low back pain** | **Number** | **2011** | **14643339.8** | **19686931.8** | **10178013.3** |
| **Prevalence** | **Global** | **Female** | **55+ years** | **Low back pain** | **Number** | **2011** | **135201887** | **156353926** | **115722384** |
| **Incidence** | **Global** | **Female** | **55+ years** | **Low back pain** | **Number** | **2011** | **53545451.6** | **62742964.4** | **44673535.8** |
| **Incidence** | **Global** | **Male** | **55+ years** | **Low back pain** | **Number** | **2011** | **30814714.6** | **36282669** | **25552753.2** |
| **Incidence** | **Global** | **Both** | **55+ years** | **Low back pain** | **Number** | **2011** | **84360166.2** | **99096958.9** | **70423681** |
| **DALYs (Disability-Adjusted Life Years)** | **Global** | **Male** | **55+ years** | **Low back pain** | **Number** | **2012** | **8357333.98** | **11381249.1** | **5719145.68** |
| **DALYs (Disability-Adjusted Life Years)** | **Global** | **Both** | **55+ years** | **Low back pain** | **Number** | **2012** | **23419917.4** | **31642571.9** | **16151441.5** |
| **Prevalence** | **Global** | **Male** | **55+ years** | **Low back pain** | **Number** | **2012** | **76000491.2** | **88516019.7** | **63789715.1** |
| **Prevalence** | **Global** | **Both** | **55+ years** | **Low back pain** | **Number** | **2012** | **215075283** | **250152742** | **182483156** |
| **DALYs (Disability-Adjusted Life Years)** | **Global** | **Female** | **55+ years** | **Low back pain** | **Number** | **2012** | **15062583.4** | **20261361.4** | **10474006** |
| **Prevalence** | **Global** | **Female** | **55+ years** | **Low back pain** | **Number** | **2012** | **139074792** | **160858657** | **118958447** |
| **Incidence** | **Global** | **Female** | **55+ years** | **Low back pain** | **Number** | **2012** | **55109172.5** | **64582787.7** | **45981853** |
| **Incidence** | **Global** | **Male** | **55+ years** | **Low back pain** | **Number** | **2012** | **31696733.2** | **37280150.6** | **26285480** |
| **Incidence** | **Global** | **Both** | **55+ years** | **Low back pain** | **Number** | **2012** | **86805905.7** | **101847828** | **72447768.4** |
| **DALYs (Disability-Adjusted Life Years)** | **Global** | **Male** | **55+ years** | **Low back pain** | **Number** | **2013** | **8591304.79** | **11704986.5** | **5887979.55** |
| **DALYs (Disability-Adjusted Life Years)** | **Global** | **Both** | **55+ years** | **Low back pain** | **Number** | **2013** | **24074407.2** | **32529436** | **16596909.2** |
| **Prevalence** | **Global** | **Male** | **55+ years** | **Low back pain** | **Number** | **2013** | **78136371** | **91058085.4** | **65585313.7** |
| **Prevalence** | **Global** | **Both** | **55+ years** | **Low back pain** | **Number** | **2013** | **221108923** | **257165591** | **187520117** |
| **DALYs (Disability-Adjusted Life Years)** | **Global** | **Female** | **55+ years** | **Low back pain** | **Number** | **2013** | **15483102.4** | **20839687.7** | **10762193.8** |
| **Prevalence** | **Global** | **Female** | **55+ years** | **Low back pain** | **Number** | **2013** | **142972552** | **165422042** | **122192730** |
| **Incidence** | **Global** | **Female** | **55+ years** | **Low back pain** | **Number** | **2013** | **56683600.8** | **66445684.7** | **47275596.7** |
| **Incidence** | **Global** | **Male** | **55+ years** | **Low back pain** | **Number** | **2013** | **32578299.5** | **38295656.3** | **27003758.2** |
| **Incidence** | **Global** | **Both** | **55+ years** | **Low back pain** | **Number** | **2013** | **89261900.3** | **104626515** | **74482611.5** |
| **DALYs (Disability-Adjusted Life Years)** | **Global** | **Male** | **55+ years** | **Low back pain** | **Number** | **2014** | **8825846.45** | **12032471.5** | **6034183.13** |
| **DALYs (Disability-Adjusted Life Years)** | **Global** | **Both** | **55+ years** | **Low back pain** | **Number** | **2014** | **24735174.9** | **33413849.4** | **17039110.9** |
| **Prevalence** | **Global** | **Male** | **55+ years** | **Low back pain** | **Number** | **2014** | **80281611.2** | **93675448.2** | **67393070** |
| **Prevalence** | **Global** | **Both** | **55+ years** | **Low back pain** | **Number** | **2014** | **227210028** | **264340235** | **192666064** |
| **DALYs (Disability-Adjusted Life Years)** | **Global** | **Female** | **55+ years** | **Low back pain** | **Number** | **2014** | **15909328.4** | **21423226** | **11057370.1** |
| **Prevalence** | **Global** | **Female** | **55+ years** | **Low back pain** | **Number** | **2014** | **146928417** | **170158717** | **125440178** |
| **Incidence** | **Global** | **Female** | **55+ years** | **Low back pain** | **Number** | **2014** | **58277263.3** | **68229879.5** | **48595556.8** |
| **Incidence** | **Global** | **Male** | **55+ years** | **Low back pain** | **Number** | **2014** | **33462858.9** | **39342514.2** | **27712755.8** |
| **Incidence** | **Global** | **Both** | **55+ years** | **Low back pain** | **Number** | **2014** | **91740122.2** | **107444150** | **76535055.3** |
| **DALYs (Disability-Adjusted Life Years)** | **Global** | **Male** | **55+ years** | **Low back pain** | **Number** | **2015** | **9063032.31** | **12344840.5** | **6193114.69** |
| **DALYs (Disability-Adjusted Life Years)** | **Global** | **Both** | **55+ years** | **Low back pain** | **Number** | **2015** | **25403973.2** | **34334803** | **17492243** |
| **Prevalence** | **Global** | **Male** | **55+ years** | **Low back pain** | **Number** | **2015** | **82444685.9** | **96285002.7** | **69209901.5** |
| **Prevalence** | **Global** | **Both** | **55+ years** | **Low back pain** | **Number** | **2015** | **233380126** | **271572652** | **197822233** |
| **DALYs (Disability-Adjusted Life Years)** | **Global** | **Female** | **55+ years** | **Low back pain** | **Number** | **2015** | **16340940.9** | **22017512.7** | **11350353.9** |
| **Prevalence** | **Global** | **Female** | **55+ years** | **Low back pain** | **Number** | **2015** | **150935440** | **174931735** | **128743904** |
| **Incidence** | **Global** | **Female** | **55+ years** | **Low back pain** | **Number** | **2015** | **59891762.6** | **70035478.2** | **49935930.7** |
| **Incidence** | **Global** | **Male** | **55+ years** | **Low back pain** | **Number** | **2015** | **34362811.1** | **40405310.6** | **28468152.3** |
| **Incidence** | **Global** | **Both** | **55+ years** | **Low back pain** | **Number** | **2015** | **94254573.6** | **110325567** | **78617452.1** |
| **DALYs (Disability-Adjusted Life Years)** | **Global** | **Male** | **55+ years** | **Low back pain** | **Number** | **2016** | **9281611.87** | **12637530.1** | **6349957.23** |
| **DALYs (Disability-Adjusted Life Years)** | **Global** | **Both** | **55+ years** | **Low back pain** | **Number** | **2016** | **26061399.6** | **35198807.9** | **17948117.1** |
| **Prevalence** | **Global** | **Male** | **55+ years** | **Low back pain** | **Number** | **2016** | **84452699.5** | **98711709.9** | **70854957.1** |
| **Prevalence** | **Global** | **Both** | **55+ years** | **Low back pain** | **Number** | **2016** | **239470413** | **278833766** | **203034857** |
| **DALYs (Disability-Adjusted Life Years)** | **Global** | **Female** | **55+ years** | **Low back pain** | **Number** | **2016** | **16779787.7** | **22593477** | **11643737** |
| **Prevalence** | **Global** | **Female** | **55+ years** | **Low back pain** | **Number** | **2016** | **155017714** | **179908976** | **132088032** |
| **Incidence** | **Global** | **Female** | **55+ years** | **Low back pain** | **Number** | **2016** | **61529928.4** | **71810614.4** | **51308724.4** |
| **Incidence** | **Global** | **Male** | **55+ years** | **Low back pain** | **Number** | **2016** | **35201886.4** | **41386124.5** | **29149281.2** |
| **Incidence** | **Global** | **Both** | **55+ years** | **Low back pain** | **Number** | **2016** | **96731814.9** | **113186890** | **80722935.9** |
| **DALYs (Disability-Adjusted Life Years)** | **Global** | **Male** | **55+ years** | **Low back pain** | **Number** | **2017** | **9481158.79** | **12899683.9** | **6487317.46** |
| **DALYs (Disability-Adjusted Life Years)** | **Global** | **Both** | **55+ years** | **Low back pain** | **Number** | **2017** | **26714107.4** | **36026124.1** | **18393989.7** |
| **Prevalence** | **Global** | **Male** | **55+ years** | **Low back pain** | **Number** | **2017** | **86304051.7** | **100938866** | **72335911.3** |
| **Prevalence** | **Global** | **Both** | **55+ years** | **Low back pain** | **Number** | **2017** | **245539564** | **286177552** | **208247248** |
| **DALYs (Disability-Adjusted Life Years)** | **Global** | **Female** | **55+ years** | **Low back pain** | **Number** | **2017** | **17232948.6** | **23175360.4** | **11937353.6** |
| **Prevalence** | **Global** | **Female** | **55+ years** | **Low back pain** | **Number** | **2017** | **159235513** | **185076451** | **135598068** |
| **Incidence** | **Global** | **Female** | **55+ years** | **Low back pain** | **Number** | **2017** | **63227104.5** | **73695607.3** | **52700086.7** |
| **Incidence** | **Global** | **Male** | **55+ years** | **Low back pain** | **Number** | **2017** | **35980345.9** | **42313658.4** | **29818670.4** |
| **Incidence** | **Global** | **Both** | **55+ years** | **Low back pain** | **Number** | **2017** | **99207450.4** | **116107089** | **82790591.4** |
| **DALYs (Disability-Adjusted Life Years)** | **Global** | **Male** | **55+ years** | **Low back pain** | **Number** | **2018** | **9692044.91** | **13157464.9** | **6619728.36** |
| **DALYs (Disability-Adjusted Life Years)** | **Global** | **Both** | **55+ years** | **Low back pain** | **Number** | **2018** | **27400768** | **36927754.7** | **18875126.2** |
| **Prevalence** | **Global** | **Male** | **55+ years** | **Low back pain** | **Number** | **2018** | **88259984.4** | **103322119** | **73895632.5** |
| **Prevalence** | **Global** | **Both** | **55+ years** | **Low back pain** | **Number** | **2018** | **251921289** | **293961112** | **213535026** |
| **DALYs (Disability-Adjusted Life Years)** | **Global** | **Female** | **55+ years** | **Low back pain** | **Number** | **2018** | **17708723.1** | **23787434.9** | **12271487** |
| **Prevalence** | **Global** | **Female** | **55+ years** | **Low back pain** | **Number** | **2018** | **163661305** | **190267025** | **139324735** |
| **Incidence** | **Global** | **Female** | **55+ years** | **Low back pain** | **Number** | **2018** | **65007529.7** | **75688563.7** | **54225299.9** |
| **Incidence** | **Global** | **Male** | **55+ years** | **Low back pain** | **Number** | **2018** | **36803830** | **43294450.9** | **30523935.8** |
| **Incidence** | **Global** | **Both** | **55+ years** | **Low back pain** | **Number** | **2018** | **101811360** | **119147538** | **84945577.8** |
| **DALYs (Disability-Adjusted Life Years)** | **Global** | **Male** | **55+ years** | **Low back pain** | **Number** | **2019** | **9939434.61** | **13475682.6** | **6791673.46** |
| **DALYs (Disability-Adjusted Life Years)** | **Global** | **Both** | **55+ years** | **Low back pain** | **Number** | **2019** | **28146310.3** | **37881039.7** | **19383578.1** |
| **Prevalence** | **Global** | **Male** | **55+ years** | **Low back pain** | **Number** | **2019** | **90546710.4** | **106095656** | **75713550.1** |
| **Prevalence** | **Global** | **Both** | **55+ years** | **Low back pain** | **Number** | **2019** | **258846369** | **302337888** | **219211849** |
| **DALYs (Disability-Adjusted Life Years)** | **Global** | **Female** | **55+ years** | **Low back pain** | **Number** | **2019** | **18206875.7** | **24408880.4** | **12598984.1** |
| **Prevalence** | **Global** | **Female** | **55+ years** | **Low back pain** | **Number** | **2019** | **168299658** | **195622507** | **143230465** |
| **Incidence** | **Global** | **Female** | **55+ years** | **Low back pain** | **Number** | **2019** | **66870302.6** | **77834605.2** | **55829486** |
| **Incidence** | **Global** | **Male** | **55+ years** | **Low back pain** | **Number** | **2019** | **37766887.3** | **44426033.6** | **31309007** |
| **Incidence** | **Global** | **Both** | **55+ years** | **Low back pain** | **Number** | **2019** | **104637190** | **122441234** | **87269041** |
| **DALYs (Disability-Adjusted Life Years)** | **Global** | **Male** | **55+ years** | **Low back pain** | **Number** | **2020** | **10218140.7** | **13924227.3** | **6984110.21** |
| **DALYs (Disability-Adjusted Life Years)** | **Global** | **Both** | **55+ years** | **Low back pain** | **Number** | **2020** | **28834664.8** | **38816195.6** | **19840699.4** |
| **Prevalence** | **Global** | **Male** | **55+ years** | **Low back pain** | **Number** | **2020** | **93174147.7** | **109149742** | **78134203.8** |
| **Prevalence** | **Global** | **Both** | **55+ years** | **Low back pain** | **Number** | **2020** | **265494287** | **310273138** | **225010551** |
| **DALYs (Disability-Adjusted Life Years)** | **Global** | **Female** | **55+ years** | **Low back pain** | **Number** | **2020** | **18616524.1** | **24994716.2** | **12903541.8** |
| **Prevalence** | **Global** | **Female** | **55+ years** | **Low back pain** | **Number** | **2020** | **172320139** | **200341228** | **146447045** |
| **Incidence** | **Global** | **Female** | **55+ years** | **Low back pain** | **Number** | **2020** | **68422705.6** | **79623057.6** | **57079532.2** |
| **Incidence** | **Global** | **Male** | **55+ years** | **Low back pain** | **Number** | **2020** | **38876028.9** | **45771402.5** | **32240564.7** |
| **Incidence** | **Global** | **Both** | **55+ years** | **Low back pain** | **Number** | **2020** | **107298734** | **125567632** | **89378769** |
| **DALYs (Disability-Adjusted Life Years)** | **Global** | **Male** | **55+ years** | **Low back pain** | **Number** | **2021** | **10392850.6** | **14102954.8** | **7120333.63** |
| **DALYs (Disability-Adjusted Life Years)** | **Global** | **Both** | **55+ years** | **Low back pain** | **Number** | **2021** | **29470958.8** | **39656202.3** | **20299620.1** |
| **Prevalence** | **Global** | **Male** | **55+ years** | **Low back pain** | **Number** | **2021** | **94848821.1** | **110964562** | **79326775.8** |
| **Prevalence** | **Global** | **Both** | **55+ years** | **Low back pain** | **Number** | **2021** | **271680057** | **316720242** | **230309073** |
| **DALYs (Disability-Adjusted Life Years)** | **Global** | **Female** | **55+ years** | **Low back pain** | **Number** | **2021** | **19078108.2** | **25575222.4** | **13229420.8** |
| **Prevalence** | **Global** | **Female** | **55+ years** | **Low back pain** | **Number** | **2021** | **176831236** | **205614417** | **150407719** |
| **Incidence** | **Global** | **Male** | **55+ years** | **Low back pain** | **Number** | **2021** | **39581955.3** | **46718109** | **32674724.6** |
| **Incidence** | **Global** | **Female** | **55+ years** | **Low back pain** | **Number** | **2021** | **70273888.3** | **81718501.4** | **58553412.9** |
| **Incidence** | **Global** | **Both** | **55+ years** | **Low back pain** | **Number** | **2021** | **109855844** | **128356824** | **91292145.1** |

**Appendix 4:** The age-standardized incidence, prevalence, and DALY rates of low back pain among postmenopausal women and age-matched men globally from 1990 to 2021

| **measure** | **location** | **sex** | **age** | **cause** | **metric** | **year** | **val** | **upper** | **lower** |
| --- | --- | --- | --- | --- | --- | --- | --- | --- | --- |
| **DALYs (Disability-Adjusted Life Years)** | **Global** | **Male** | **Age-standardized** | **Low back pain** | **Rate** | **1990** | **272.831696** | **374.743012** | **185.263676** |
| **DALYs (Disability-Adjusted Life Years)** | **Global** | **Female** | **Age-standardized** | **Low back pain** | **Rate** | **1990** | **443.831859** | **599.396009** | **306.605297** |
| **DALYs (Disability-Adjusted Life Years)** | **Global** | **Both** | **Age-standardized** | **Low back pain** | **Rate** | **1990** | **364.502576** | **497.185335** | **249.262928** |
| **Prevalence** | **Global** | **Female** | **Age-standardized** | **Low back pain** | **Rate** | **1990** | **4086.48812** | **4777.12644** | **3460.24062** |
| **Incidence** | **Global** | **Female** | **Age-standardized** | **Low back pain** | **Rate** | **1990** | **1598.4671** | **1872.46068** | **1331.65531** |
| **Prevalence** | **Global** | **Male** | **Age-standardized** | **Low back pain** | **Rate** | **1990** | **2475.04958** | **2934.65906** | **2031.3477** |
| **Prevalence** | **Global** | **Both** | **Age-standardized** | **Low back pain** | **Rate** | **1990** | **3338.92003** | **3922.76822** | **2799.38959** |
| **Incidence** | **Global** | **Male** | **Age-standardized** | **Low back pain** | **Rate** | **1990** | **1031.31345** | **1227.52231** | **846.954371** |
| **Incidence** | **Global** | **Both** | **Age-standardized** | **Low back pain** | **Rate** | **1990** | **1335.35687** | **1575.54581** | **1110.2562** |
| **DALYs (Disability-Adjusted Life Years)** | **Global** | **Female** | **Age-standardized** | **Low back pain** | **Rate** | **1991** | **438.915653** | **592.15719** | **303.1609** |
| **Prevalence** | **Global** | **Female** | **Age-standardized** | **Low back pain** | **Rate** | **1991** | **4041.12075** | **4726.63681** | **3421.4851** |
| **Incidence** | **Global** | **Female** | **Age-standardized** | **Low back pain** | **Rate** | **1991** | **1583.90857** | **1853.62774** | **1320.12884** |
| **DALYs (Disability-Adjusted Life Years)** | **Global** | **Male** | **Age-standardized** | **Low back pain** | **Rate** | **1991** | **270.814761** | **371.8607** | **184.08287** |
| **DALYs (Disability-Adjusted Life Years)** | **Global** | **Both** | **Age-standardized** | **Low back pain** | **Rate** | **1991** | **360.813057** | **491.182796** | **246.981706** |
| **Prevalence** | **Global** | **Male** | **Age-standardized** | **Low back pain** | **Rate** | **1991** | **2456.79699** | **2910.80631** | **2019.29399** |
| **Prevalence** | **Global** | **Both** | **Age-standardized** | **Low back pain** | **Rate** | **1991** | **3305.0164** | **3884.34508** | **2772.01549** |
| **Incidence** | **Global** | **Male** | **Age-standardized** | **Low back pain** | **Rate** | **1991** | **1025.0014** | **1220.07535** | **842.367947** |
| **Incidence** | **Global** | **Both** | **Age-standardized** | **Low back pain** | **Rate** | **1991** | **1324.23058** | **1560.95199** | **1102.54691** |
| **DALYs (Disability-Adjusted Life Years)** | **Global** | **Male** | **Age-standardized** | **Low back pain** | **Rate** | **1992** | **269.030604** | **369.177672** | **182.917221** |
| **DALYs (Disability-Adjusted Life Years)** | **Global** | **Both** | **Age-standardized** | **Low back pain** | **Rate** | **1992** | **357.44881** | **486.239255** | **244.37757** |
| **DALYs (Disability-Adjusted Life Years)** | **Global** | **Female** | **Age-standardized** | **Low back pain** | **Rate** | **1992** | **434.354835** | **586.440371** | **299.724825** |
| **Prevalence** | **Global** | **Female** | **Age-standardized** | **Low back pain** | **Rate** | **1992** | **3999.21599** | **4674.60776** | **3384.72528** |
| **Incidence** | **Global** | **Female** | **Age-standardized** | **Low back pain** | **Rate** | **1992** | **1570.60956** | **1836.75576** | **1309.02287** |
| **Prevalence** | **Global** | **Male** | **Age-standardized** | **Low back pain** | **Rate** | **1992** | **2440.52251** | **2889.70502** | **2009.18299** |
| **Prevalence** | **Global** | **Both** | **Age-standardized** | **Low back pain** | **Rate** | **1992** | **3274.13825** | **3851.37122** | **2747.06191** |
| **Incidence** | **Global** | **Male** | **Age-standardized** | **Low back pain** | **Rate** | **1992** | **1019.3634** | **1212.22194** | **838.298984** |
| **Incidence** | **Global** | **Both** | **Age-standardized** | **Low back pain** | **Rate** | **1992** | **1314.17921** | **1547.79087** | **1094.83029** |
| **DALYs (Disability-Adjusted Life Years)** | **Global** | **Male** | **Age-standardized** | **Low back pain** | **Rate** | **1993** | **267.556393** | **367.089908** | **182.020251** |
| **DALYs (Disability-Adjusted Life Years)** | **Global** | **Both** | **Age-standardized** | **Low back pain** | **Rate** | **1993** | **354.642385** | **482.213814** | **242.498207** |
| **DALYs (Disability-Adjusted Life Years)** | **Global** | **Female** | **Age-standardized** | **Low back pain** | **Rate** | **1993** | **430.521831** | **580.96125** | **296.638088** |
| **Prevalence** | **Global** | **Female** | **Age-standardized** | **Low back pain** | **Rate** | **1993** | **3964.04257** | **4630.07037** | **3352.52631** |
| **Incidence** | **Global** | **Female** | **Age-standardized** | **Low back pain** | **Rate** | **1993** | **1559.54679** | **1822.59187** | **1299.63452** |
| **Prevalence** | **Global** | **Male** | **Age-standardized** | **Low back pain** | **Rate** | **1993** | **2427.33121** | **2872.88469** | **2002.87689** |
| **Prevalence** | **Global** | **Both** | **Age-standardized** | **Low back pain** | **Rate** | **1993** | **3248.52395** | **3823.50351** | **2726.3089** |
| **Incidence** | **Global** | **Male** | **Age-standardized** | **Low back pain** | **Rate** | **1993** | **1014.75286** | **1205.86842** | **834.664499** |
| **Incidence** | **Global** | **Both** | **Age-standardized** | **Low back pain** | **Rate** | **1993** | **1305.88159** | **1536.78656** | **1088.38477** |
| **DALYs (Disability-Adjusted Life Years)** | **Global** | **Male** | **Age-standardized** | **Low back pain** | **Rate** | **1994** | **266.457235** | **365.476228** | **181.395244** |
| **DALYs (Disability-Adjusted Life Years)** | **Global** | **Both** | **Age-standardized** | **Low back pain** | **Rate** | **1994** | **352.583667** | **478.570842** | **241.316622** |
| **DALYs (Disability-Adjusted Life Years)** | **Global** | **Female** | **Age-standardized** | **Low back pain** | **Rate** | **1994** | **427.743103** | **576.530257** | **294.333139** |
| **Prevalence** | **Global** | **Female** | **Age-standardized** | **Low back pain** | **Rate** | **1994** | **3939.11827** | **4597.12225** | **3329.16078** |
| **Incidence** | **Global** | **Female** | **Age-standardized** | **Low back pain** | **Rate** | **1994** | **1551.71727** | **1812.85048** | **1293.20312** |
| **Prevalence** | **Global** | **Male** | **Age-standardized** | **Low back pain** | **Rate** | **1994** | **2417.84118** | **2860.54916** | **1999.09794** |
| **Prevalence** | **Global** | **Both** | **Age-standardized** | **Low back pain** | **Rate** | **1994** | **3230.20107** | **3797.49223** | **2711.51575** |
| **Incidence** | **Global** | **Male** | **Age-standardized** | **Low back pain** | **Rate** | **1994** | **1011.31803** | **1201.42041** | **831.876062** |
| **Incidence** | **Global** | **Both** | **Age-standardized** | **Low back pain** | **Rate** | **1994** | **1299.89049** | **1528.80935** | **1083.20741** |
| **DALYs (Disability-Adjusted Life Years)** | **Global** | **Male** | **Age-standardized** | **Low back pain** | **Rate** | **1995** | **265.944058** | **364.685551** | **181.024002** |
| **DALYs (Disability-Adjusted Life Years)** | **Global** | **Both** | **Age-standardized** | **Low back pain** | **Rate** | **1995** | **351.533483** | **477.042549** | **240.56951** |
| **DALYs (Disability-Adjusted Life Years)** | **Global** | **Female** | **Age-standardized** | **Low back pain** | **Rate** | **1995** | **426.2936** | **574.719448** | **293.060482** |
| **Prevalence** | **Global** | **Female** | **Age-standardized** | **Low back pain** | **Rate** | **1995** | **3926.21433** | **4581.93982** | **3315.97207** |
| **Incidence** | **Global** | **Female** | **Age-standardized** | **Low back pain** | **Rate** | **1995** | **1547.68015** | **1808.56574** | **1290.3093** |
| **Prevalence** | **Global** | **Male** | **Age-standardized** | **Low back pain** | **Rate** | **1995** | **2413.81685** | **2856.66045** | **1998.7924** |
| **Prevalence** | **Global** | **Both** | **Age-standardized** | **Low back pain** | **Rate** | **1995** | **3221.08596** | **3784.43874** | **2703.98575** |
| **Incidence** | **Global** | **Male** | **Age-standardized** | **Low back pain** | **Rate** | **1995** | **1009.74169** | **1199.1325** | **831.274231** |
| **Incidence** | **Global** | **Both** | **Age-standardized** | **Low back pain** | **Rate** | **1995** | **1296.87593** | **1524.85837** | **1080.38911** |
| **DALYs (Disability-Adjusted Life Years)** | **Global** | **Male** | **Age-standardized** | **Low back pain** | **Rate** | **1996** | **265.459023** | **362.670491** | **181.070414** |
| **DALYs (Disability-Adjusted Life Years)** | **Global** | **Both** | **Age-standardized** | **Low back pain** | **Rate** | **1996** | **350.721399** | **475.510977** | **240.314113** |
| **DALYs (Disability-Adjusted Life Years)** | **Global** | **Female** | **Age-standardized** | **Low back pain** | **Rate** | **1996** | **425.260684** | **571.997225** | **292.818423** |
| **Prevalence** | **Global** | **Female** | **Age-standardized** | **Low back pain** | **Rate** | **1996** | **3917.93816** | **4565.8732** | **3311.96676** |
| **Incidence** | **Global** | **Female** | **Age-standardized** | **Low back pain** | **Rate** | **1996** | **1544.87992** | **1802.49287** | **1289.04335** |
| **Prevalence** | **Global** | **Male** | **Age-standardized** | **Low back pain** | **Rate** | **1996** | **2410.24494** | **2847.37889** | **1999.31177** |
| **Prevalence** | **Global** | **Both** | **Age-standardized** | **Low back pain** | **Rate** | **1996** | **3214.67654** | **3770.07682** | **2702.28675** |
| **Incidence** | **Global** | **Male** | **Age-standardized** | **Low back pain** | **Rate** | **1996** | **1007.9614** | **1196.40599** | **831.547314** |
| **Incidence** | **Global** | **Both** | **Age-standardized** | **Low back pain** | **Rate** | **1996** | **1294.43495** | **1520.05725** | **1078.75697** |
| **DALYs (Disability-Adjusted Life Years)** | **Global** | **Male** | **Age-standardized** | **Low back pain** | **Rate** | **1997** | **264.55669** | **361.092899** | **180.566622** |
| **DALYs (Disability-Adjusted Life Years)** | **Global** | **Both** | **Age-standardized** | **Low back pain** | **Rate** | **1997** | **349.511729** | **472.880255** | **240.026942** |
| **DALYs (Disability-Adjusted Life Years)** | **Global** | **Female** | **Age-standardized** | **Low back pain** | **Rate** | **1997** | **423.825323** | **569.333434** | **291.997328** |
| **Prevalence** | **Global** | **Female** | **Age-standardized** | **Low back pain** | **Rate** | **1997** | **3906.08515** | **4545.41357** | **3306.70517** |
| **Incidence** | **Global** | **Female** | **Age-standardized** | **Low back pain** | **Rate** | **1997** | **1540.64065** | **1795.20796** | **1286.51121** |
| **Prevalence** | **Global** | **Male** | **Age-standardized** | **Low back pain** | **Rate** | **1997** | **2403.04589** | **2833.96998** | **1996.30098** |
| **Prevalence** | **Global** | **Both** | **Age-standardized** | **Low back pain** | **Rate** | **1997** | **3204.77789** | **3752.98298** | **2699.59768** |
| **Incidence** | **Global** | **Male** | **Age-standardized** | **Low back pain** | **Rate** | **1997** | **1004.36862** | **1190.86533** | **829.109292** |
| **Incidence** | **Global** | **Both** | **Age-standardized** | **Low back pain** | **Rate** | **1997** | **1290.41999** | **1513.68131** | **1075.72183** |
| **DALYs (Disability-Adjusted Life Years)** | **Global** | **Male** | **Age-standardized** | **Low back pain** | **Rate** | **1998** | **263.439767** | **359.389091** | **180.08059** |
| **DALYs (Disability-Adjusted Life Years)** | **Global** | **Both** | **Age-standardized** | **Low back pain** | **Rate** | **1998** | **348.071146** | **470.675809** | **239.320712** |
| **DALYs (Disability-Adjusted Life Years)** | **Global** | **Female** | **Age-standardized** | **Low back pain** | **Rate** | **1998** | **422.168869** | **566.686854** | **291.173001** |
| **Prevalence** | **Global** | **Female** | **Age-standardized** | **Low back pain** | **Rate** | **1998** | **3892.62314** | **4522.38838** | **3300.31629** |
| **Incidence** | **Global** | **Female** | **Age-standardized** | **Low back pain** | **Rate** | **1998** | **1535.69634** | **1787.1981** | **1283.48383** |
| **Prevalence** | **Global** | **Male** | **Age-standardized** | **Low back pain** | **Rate** | **1998** | **2394.45459** | **2819.51453** | **1992.15957** |
| **Prevalence** | **Global** | **Both** | **Age-standardized** | **Low back pain** | **Rate** | **1998** | **3193.24996** | **3734.30974** | **2695.84312** |
| **Incidence** | **Global** | **Male** | **Age-standardized** | **Low back pain** | **Rate** | **1998** | **999.958759** | **1184.03284** | **826.351541** |
| **Incidence** | **Global** | **Both** | **Age-standardized** | **Low back pain** | **Rate** | **1998** | **1285.60399** | **1506.39879** | **1071.26985** |
| **DALYs (Disability-Adjusted Life Years)** | **Global** | **Male** | **Age-standardized** | **Low back pain** | **Rate** | **1999** | **262.444479** | **357.541702** | **179.414187** |
| **DALYs (Disability-Adjusted Life Years)** | **Global** | **Both** | **Age-standardized** | **Low back pain** | **Rate** | **1999** | **346.763992** | **468.718148** | **238.870425** |
| **DALYs (Disability-Adjusted Life Years)** | **Global** | **Female** | **Age-standardized** | **Low back pain** | **Rate** | **1999** | **420.641357** | **563.549217** | **290.586809** |
| **Prevalence** | **Global** | **Female** | **Age-standardized** | **Low back pain** | **Rate** | **1999** | **3880.69588** | **4501.32137** | **3293.95411** |
| **Incidence** | **Global** | **Female** | **Age-standardized** | **Low back pain** | **Rate** | **1999** | **1531.28619** | **1780.53731** | **1280.8538** |
| **Prevalence** | **Global** | **Male** | **Age-standardized** | **Low back pain** | **Rate** | **1999** | **2386.86968** | **2809.99086** | **1988.60012** |
| **Prevalence** | **Global** | **Both** | **Age-standardized** | **Low back pain** | **Rate** | **1999** | **3183.08449** | **3717.24796** | **2693.1507** |
| **Incidence** | **Global** | **Male** | **Age-standardized** | **Low back pain** | **Rate** | **1999** | **995.955293** | **1177.66489** | **824.338035** |
| **Incidence** | **Global** | **Both** | **Age-standardized** | **Low back pain** | **Rate** | **1999** | **1281.28861** | **1499.85837** | **1067.35945** |
| **DALYs (Disability-Adjusted Life Years)** | **Global** | **Male** | **Age-standardized** | **Low back pain** | **Rate** | **2000** | **261.816863** | **356.425284** | **179.456181** |
| **DALYs (Disability-Adjusted Life Years)** | **Global** | **Both** | **Age-standardized** | **Low back pain** | **Rate** | **2000** | **345.921747** | **466.914235** | **238.636349** |
| **DALYs (Disability-Adjusted Life Years)** | **Global** | **Female** | **Age-standardized** | **Low back pain** | **Rate** | **2000** | **419.6679** | **560.841724** | **290.20857** |
| **Prevalence** | **Global** | **Female** | **Age-standardized** | **Low back pain** | **Rate** | **2000** | **3873.30539** | **4491.90964** | **3292.12753** |
| **Incidence** | **Global** | **Female** | **Age-standardized** | **Low back pain** | **Rate** | **2000** | **1528.73429** | **1776.39654** | **1280.04633** |
| **Prevalence** | **Global** | **Male** | **Age-standardized** | **Low back pain** | **Rate** | **2000** | **2382.48971** | **2801.00911** | **1987.87224** |
| **Prevalence** | **Global** | **Both** | **Age-standardized** | **Low back pain** | **Rate** | **2000** | **3176.81379** | **3703.05104** | **2690.26444** |
| **Incidence** | **Global** | **Male** | **Age-standardized** | **Low back pain** | **Rate** | **2000** | **993.495804** | **1174.88668** | **823.651221** |
| **Incidence** | **Global** | **Both** | **Age-standardized** | **Low back pain** | **Rate** | **2000** | **1278.67714** | **1495.17886** | **1065.02358** |
| **DALYs (Disability-Adjusted Life Years)** | **Global** | **Male** | **Age-standardized** | **Low back pain** | **Rate** | **2001** | **261.405809** | **355.931459** | **179.090534** |
| **DALYs (Disability-Adjusted Life Years)** | **Global** | **Both** | **Age-standardized** | **Low back pain** | **Rate** | **2001** | **345.355963** | **466.051516** | **238.519273** |
| **DALYs (Disability-Adjusted Life Years)** | **Global** | **Female** | **Age-standardized** | **Low back pain** | **Rate** | **2001** | **419.029399** | **559.952774** | **289.958473** |
| **Prevalence** | **Global** | **Female** | **Age-standardized** | **Low back pain** | **Rate** | **2001** | **3868.91398** | **4485.19024** | **3289.44715** |
| **Incidence** | **Global** | **Female** | **Age-standardized** | **Low back pain** | **Rate** | **2001** | **1527.65878** | **1776.07023** | **1279.32382** |
| **Prevalence** | **Global** | **Male** | **Age-standardized** | **Low back pain** | **Rate** | **2001** | **2379.58624** | **2792.46136** | **1987.81175** |
| **Prevalence** | **Global** | **Both** | **Age-standardized** | **Low back pain** | **Rate** | **2001** | **3172.80058** | **3693.41396** | **2688.6454** |
| **Incidence** | **Global** | **Male** | **Age-standardized** | **Low back pain** | **Rate** | **2001** | **991.900012** | **1172.2498** | **823.39209** |
| **Incidence** | **Global** | **Both** | **Age-standardized** | **Low back pain** | **Rate** | **2001** | **1277.24455** | **1492.66373** | **1064.37427** |
| **DALYs (Disability-Adjusted Life Years)** | **Global** | **Male** | **Age-standardized** | **Low back pain** | **Rate** | **2002** | **261.000189** | **354.594143** | **178.621566** |
| **DALYs (Disability-Adjusted Life Years)** | **Global** | **Both** | **Age-standardized** | **Low back pain** | **Rate** | **2002** | **344.774433** | **464.92867** | **238.314884** |
| **DALYs (Disability-Adjusted Life Years)** | **Global** | **Female** | **Age-standardized** | **Low back pain** | **Rate** | **2002** | **418.401635** | **558.855379** | **289.882165** |
| **Prevalence** | **Global** | **Female** | **Age-standardized** | **Low back pain** | **Rate** | **2002** | **3863.38263** | **4475.58924** | **3287.2014** |
| **Incidence** | **Global** | **Female** | **Age-standardized** | **Low back pain** | **Rate** | **2002** | **1526.43492** | **1775.61876** | **1278.41116** |
| **Prevalence** | **Global** | **Male** | **Age-standardized** | **Low back pain** | **Rate** | **2002** | **2375.91991** | **2785.9618** | **1987.03327** |
| **Prevalence** | **Global** | **Both** | **Age-standardized** | **Low back pain** | **Rate** | **2002** | **3167.59665** | **3685.25433** | **2687.0723** |
| **Incidence** | **Global** | **Male** | **Age-standardized** | **Low back pain** | **Rate** | **2002** | **990.030257** | **1168.14088** | **822.313359** |
| **Incidence** | **Global** | **Both** | **Age-standardized** | **Low back pain** | **Rate** | **2002** | **1275.52252** | **1490.76714** | **1064.49004** |
| **DALYs (Disability-Adjusted Life Years)** | **Global** | **Male** | **Age-standardized** | **Low back pain** | **Rate** | **2003** | **260.546439** | **354.208404** | **178.450897** |
| **DALYs (Disability-Adjusted Life Years)** | **Global** | **Both** | **Age-standardized** | **Low back pain** | **Rate** | **2003** | **344.148146** | **463.702575** | **238.059385** |
| **DALYs (Disability-Adjusted Life Years)** | **Global** | **Female** | **Age-standardized** | **Low back pain** | **Rate** | **2003** | **417.749521** | **558.280654** | **289.857551** |
| **Prevalence** | **Global** | **Female** | **Age-standardized** | **Low back pain** | **Rate** | **2003** | **3857.68633** | **4465.66564** | **3284.10869** |
| **Incidence** | **Global** | **Female** | **Age-standardized** | **Low back pain** | **Rate** | **2003** | **1525.21356** | **1775.88615** | **1277.42413** |
| **Prevalence** | **Global** | **Male** | **Age-standardized** | **Low back pain** | **Rate** | **2003** | **2371.51506** | **2778.28597** | **1985.35115** |
| **Prevalence** | **Global** | **Both** | **Age-standardized** | **Low back pain** | **Rate** | **2003** | **3161.87142** | **3675.31086** | **2683.80803** |
| **Incidence** | **Global** | **Male** | **Age-standardized** | **Low back pain** | **Rate** | **2003** | **987.942612** | **1164.1528** | **821.142498** |
| **Incidence** | **Global** | **Both** | **Age-standardized** | **Low back pain** | **Rate** | **2003** | **1273.66709** | **1489.22469** | **1064.90333** |
| **DALYs (Disability-Adjusted Life Years)** | **Global** | **Male** | **Age-standardized** | **Low back pain** | **Rate** | **2004** | **259.666267** | **352.586295** | **177.815277** |
| **DALYs (Disability-Adjusted Life Years)** | **Global** | **Both** | **Age-standardized** | **Low back pain** | **Rate** | **2004** | **343.0841** | **462.27425** | **237.348968** |
| **DALYs (Disability-Adjusted Life Years)** | **Global** | **Female** | **Age-standardized** | **Low back pain** | **Rate** | **2004** | **416.674823** | **556.670848** | **289.016147** |
| **Prevalence** | **Global** | **Female** | **Age-standardized** | **Low back pain** | **Rate** | **2004** | **3847.69211** | **4450.87121** | **3277.3697** |
| **Incidence** | **Global** | **Female** | **Age-standardized** | **Low back pain** | **Rate** | **2004** | **1522.21166** | **1774.83906** | **1273.89763** |
| **Prevalence** | **Global** | **Male** | **Age-standardized** | **Low back pain** | **Rate** | **2004** | **2363.24603** | **2763.74061** | **1979.79972** |
| **Prevalence** | **Global** | **Both** | **Age-standardized** | **Low back pain** | **Rate** | **2004** | **3151.92455** | **3659.81478** | **2675.70762** |
| **Incidence** | **Global** | **Male** | **Age-standardized** | **Low back pain** | **Rate** | **2004** | **984.442581** | **1161.4372** | **818.755624** |
| **Incidence** | **Global** | **Both** | **Age-standardized** | **Low back pain** | **Rate** | **2004** | **1270.15651** | **1486.70649** | **1062.41236** |
| **DALYs (Disability-Adjusted Life Years)** | **Global** | **Male** | **Age-standardized** | **Low back pain** | **Rate** | **2005** | **259.094805** | **351.534045** | **177.340484** |
| **DALYs (Disability-Adjusted Life Years)** | **Global** | **Both** | **Age-standardized** | **Low back pain** | **Rate** | **2005** | **342.316942** | **461.440407** | **237.115437** |
| **DALYs (Disability-Adjusted Life Years)** | **Global** | **Female** | **Age-standardized** | **Low back pain** | **Rate** | **2005** | **415.80409** | **556.826165** | **288.896517** |
| **Prevalence** | **Global** | **Female** | **Age-standardized** | **Low back pain** | **Rate** | **2005** | **3839.8387** | **4438.48065** | **3274.56771** |
| **Prevalence** | **Global** | **Male** | **Age-standardized** | **Low back pain** | **Rate** | **2005** | **2357.87974** | **2753.32454** | **1976.82541** |
| **Incidence** | **Global** | **Female** | **Age-standardized** | **Low back pain** | **Rate** | **2005** | **1519.58544** | **1773.58638** | **1272.72202** |
| **Prevalence** | **Global** | **Both** | **Age-standardized** | **Low back pain** | **Rate** | **2005** | **3144.88985** | **3649.99071** | **2672.56737** |
| **Incidence** | **Global** | **Male** | **Age-standardized** | **Low back pain** | **Rate** | **2005** | **982.093747** | **1159.52983** | **816.957372** |
| **Incidence** | **Global** | **Both** | **Age-standardized** | **Low back pain** | **Rate** | **2005** | **1267.53445** | **1484.72954** | **1060.06768** |
| **DALYs (Disability-Adjusted Life Years)** | **Global** | **Male** | **Age-standardized** | **Low back pain** | **Rate** | **2006** | **258.202738** | **350.962578** | **176.716324** |
| **DALYs (Disability-Adjusted Life Years)** | **Global** | **Both** | **Age-standardized** | **Low back pain** | **Rate** | **2006** | **341.108046** | **459.49396** | **235.902947** |
| **Prevalence** | **Global** | **Male** | **Age-standardized** | **Low back pain** | **Rate** | **2006** | **2349.44354** | **2740.25443** | **1970.40525** |
| **DALYs (Disability-Adjusted Life Years)** | **Global** | **Female** | **Age-standardized** | **Low back pain** | **Rate** | **2006** | **414.409223** | **554.963245** | **287.480368** |
| **Prevalence** | **Global** | **Both** | **Age-standardized** | **Low back pain** | **Rate** | **2006** | **3133.54364** | **3636.66237** | **2664.29082** |
| **Prevalence** | **Global** | **Female** | **Age-standardized** | **Low back pain** | **Rate** | **2006** | **3826.80999** | **4423.94507** | **3267.15432** |
| **Incidence** | **Global** | **Female** | **Age-standardized** | **Low back pain** | **Rate** | **2006** | **1514.57376** | **1768.95537** | **1268.13289** |
| **Incidence** | **Global** | **Male** | **Age-standardized** | **Low back pain** | **Rate** | **2006** | **978.66286** | **1155.4896** | **812.736946** |
| **Incidence** | **Global** | **Both** | **Age-standardized** | **Low back pain** | **Rate** | **2006** | **1263.09316** | **1480.98175** | **1055.96656** |
| **DALYs (Disability-Adjusted Life Years)** | **Global** | **Male** | **Age-standardized** | **Low back pain** | **Rate** | **2007** | **257.489309** | **350.133355** | **176.450601** |
| **DALYs (Disability-Adjusted Life Years)** | **Global** | **Both** | **Age-standardized** | **Low back pain** | **Rate** | **2007** | **339.949602** | **458.227262** | **234.895782** |
| **Prevalence** | **Global** | **Male** | **Age-standardized** | **Low back pain** | **Rate** | **2007** | **2342.43733** | **2730.32315** | **1965.18463** |
| **Prevalence** | **Global** | **Both** | **Age-standardized** | **Low back pain** | **Rate** | **2007** | **3122.56391** | **3624.45317** | **2654.86495** |
| **DALYs (Disability-Adjusted Life Years)** | **Global** | **Female** | **Age-standardized** | **Low back pain** | **Rate** | **2007** | **412.94944** | **554.04033** | **286.307445** |
| **Prevalence** | **Global** | **Female** | **Age-standardized** | **Low back pain** | **Rate** | **2007** | **3813.18859** | **4413.42587** | **3257.96777** |
| **Incidence** | **Global** | **Female** | **Age-standardized** | **Low back pain** | **Rate** | **2007** | **1508.99197** | **1763.60227** | **1262.3611** |
| **Incidence** | **Global** | **Male** | **Age-standardized** | **Low back pain** | **Rate** | **2007** | **975.994295** | **1151.6631** | **810.011068** |
| **Incidence** | **Global** | **Both** | **Age-standardized** | **Low back pain** | **Rate** | **2007** | **1258.7108** | **1475.80427** | **1051.97163** |
| **DALYs (Disability-Adjusted Life Years)** | **Global** | **Male** | **Age-standardized** | **Low back pain** | **Rate** | **2008** | **256.655814** | **349.350326** | **175.712107** |
| **DALYs (Disability-Adjusted Life Years)** | **Global** | **Both** | **Age-standardized** | **Low back pain** | **Rate** | **2008** | **338.641537** | **457.13388** | **233.934954** |
| **Prevalence** | **Global** | **Male** | **Age-standardized** | **Low back pain** | **Rate** | **2008** | **2334.61455** | **2720.41834** | **1959.14132** |
| **Prevalence** | **Global** | **Both** | **Age-standardized** | **Low back pain** | **Rate** | **2008** | **3110.29889** | **3611.08879** | **2643.96233** |
| **DALYs (Disability-Adjusted Life Years)** | **Global** | **Female** | **Age-standardized** | **Low back pain** | **Rate** | **2008** | **411.306591** | **552.073578** | **285.736277** |
| **Prevalence** | **Global** | **Female** | **Age-standardized** | **Low back pain** | **Rate** | **2008** | **3797.7984** | **4395.54419** | **3247.23643** |
| **Incidence** | **Global** | **Female** | **Age-standardized** | **Low back pain** | **Rate** | **2008** | **1502.70872** | **1757.50824** | **1255.97051** |
| **Incidence** | **Global** | **Male** | **Age-standardized** | **Low back pain** | **Rate** | **2008** | **973.060232** | **1147.47428** | **806.944796** |
| **Incidence** | **Global** | **Both** | **Age-standardized** | **Low back pain** | **Rate** | **2008** | **1253.84521** | **1470.81506** | **1047.57211** |
| **DALYs (Disability-Adjusted Life Years)** | **Global** | **Male** | **Age-standardized** | **Low back pain** | **Rate** | **2009** | **255.833742** | **348.025693** | **175.143499** |
| **DALYs (Disability-Adjusted Life Years)** | **Global** | **Both** | **Age-standardized** | **Low back pain** | **Rate** | **2009** | **337.35034** | **455.378074** | **232.965477** |
| **Prevalence** | **Global** | **Male** | **Age-standardized** | **Low back pain** | **Rate** | **2009** | **2326.60553** | **2711.33167** | **1952.44891** |
| **Prevalence** | **Global** | **Both** | **Age-standardized** | **Low back pain** | **Rate** | **2009** | **3098.15088** | **3599.66092** | **2632.61054** |
| **DALYs (Disability-Adjusted Life Years)** | **Global** | **Female** | **Age-standardized** | **Low back pain** | **Rate** | **2009** | **409.681207** | **550.656427** | **284.649603** |
| **Prevalence** | **Global** | **Female** | **Age-standardized** | **Low back pain** | **Rate** | **2009** | **3782.75434** | **4377.50151** | **3236.08537** |
| **Incidence** | **Global** | **Female** | **Age-standardized** | **Low back pain** | **Rate** | **2009** | **1496.81177** | **1752.34482** | **1249.70802** |
| **Incidence** | **Global** | **Male** | **Age-standardized** | **Low back pain** | **Rate** | **2009** | **970.104951** | **1142.91067** | **804.475192** |
| **Incidence** | **Global** | **Both** | **Age-standardized** | **Low back pain** | **Rate** | **2009** | **1249.18233** | **1466.61017** | **1043.44651** |
| **DALYs (Disability-Adjusted Life Years)** | **Global** | **Male** | **Age-standardized** | **Low back pain** | **Rate** | **2010** | **254.879253** | **347.154778** | **174.442137** |
| **DALYs (Disability-Adjusted Life Years)** | **Global** | **Both** | **Age-standardized** | **Low back pain** | **Rate** | **2010** | **336.03577** | **453.561436** | **231.729222** |
| **Prevalence** | **Global** | **Male** | **Age-standardized** | **Low back pain** | **Rate** | **2010** | **2317.6377** | **2700.02251** | **1945.10281** |
| **Prevalence** | **Global** | **Both** | **Age-standardized** | **Low back pain** | **Rate** | **2010** | **3085.89769** | **3587.33207** | **2621.32472** |
| **DALYs (Disability-Adjusted Life Years)** | **Global** | **Female** | **Age-standardized** | **Low back pain** | **Rate** | **2010** | **408.115238** | **548.558035** | **283.634643** |
| **Prevalence** | **Global** | **Female** | **Age-standardized** | **Low back pain** | **Rate** | **2010** | **3768.2307** | **4359.95335** | **3226.01719** |
| **Incidence** | **Global** | **Female** | **Age-standardized** | **Low back pain** | **Rate** | **2010** | **1491.55759** | **1747.39608** | **1244.36188** |
| **Incidence** | **Global** | **Male** | **Age-standardized** | **Low back pain** | **Rate** | **2010** | **966.670431** | **1138.13829** | **801.762978** |
| **Incidence** | **Global** | **Both** | **Age-standardized** | **Low back pain** | **Rate** | **2010** | **1244.66006** | **1462.11638** | **1039.45103** |
| **DALYs (Disability-Adjusted Life Years)** | **Global** | **Male** | **Age-standardized** | **Low back pain** | **Rate** | **2011** | **254.1159** | **346.079733** | **173.836395** |
| **DALYs (Disability-Adjusted Life Years)** | **Global** | **Both** | **Age-standardized** | **Low back pain** | **Rate** | **2011** | **335.105673** | **452.744851** | **231.154757** |
| **Prevalence** | **Global** | **Male** | **Age-standardized** | **Low back pain** | **Rate** | **2011** | **2310.47875** | **2690.10043** | **1938.93348** |
| **Prevalence** | **Global** | **Both** | **Age-standardized** | **Low back pain** | **Rate** | **2011** | **3077.19563** | **3578.3189** | **2611.95202** |
| **DALYs (Disability-Adjusted Life Years)** | **Global** | **Female** | **Age-standardized** | **Low back pain** | **Rate** | **2011** | **407.092695** | **547.307257** | **282.954226** |
| **Prevalence** | **Global** | **Female** | **Age-standardized** | **Low back pain** | **Rate** | **2011** | **3758.68493** | **4346.72295** | **3217.14429** |
| **Incidence** | **Global** | **Female** | **Age-standardized** | **Low back pain** | **Rate** | **2011** | **1488.59225** | **1744.28804** | **1241.94824** |
| **Incidence** | **Global** | **Male** | **Age-standardized** | **Low back pain** | **Rate** | **2011** | **963.800837** | **1134.82365** | **799.220932** |
| **Incidence** | **Global** | **Both** | **Age-standardized** | **Low back pain** | **Rate** | **2011** | **1241.63865** | **1458.53926** | **1036.51721** |
| **DALYs (Disability-Adjusted Life Years)** | **Global** | **Male** | **Age-standardized** | **Low back pain** | **Rate** | **2012** | **253.593272** | **345.350347** | **173.540614** |
| **DALYs (Disability-Adjusted Life Years)** | **Global** | **Both** | **Age-standardized** | **Low back pain** | **Rate** | **2012** | **334.547263** | **452.005686** | **230.719027** |
| **Prevalence** | **Global** | **Male** | **Age-standardized** | **Low back pain** | **Rate** | **2012** | **2306.14371** | **2685.91241** | **1935.62237** |
| **Prevalence** | **Global** | **Both** | **Age-standardized** | **Low back pain** | **Rate** | **2012** | **3072.29296** | **3573.36509** | **2606.72313** |
| **DALYs (Disability-Adjusted Life Years)** | **Global** | **Female** | **Age-standardized** | **Low back pain** | **Rate** | **2012** | **406.556809** | **546.877929** | **282.705718** |
| **Prevalence** | **Global** | **Female** | **Age-standardized** | **Low back pain** | **Rate** | **2012** | **3753.7919** | **4341.76398** | **3210.82813** |
| **Incidence** | **Global** | **Female** | **Age-standardized** | **Low back pain** | **Rate** | **2012** | **1487.46126** | **1743.16526** | **1241.10419** |
| **Incidence** | **Global** | **Male** | **Age-standardized** | **Low back pain** | **Rate** | **2012** | **961.799337** | **1131.22144** | **797.601351** |
| **Incidence** | **Global** | **Both** | **Age-standardized** | **Low back pain** | **Rate** | **2012** | **1239.99917** | **1454.86901** | **1034.89702** |
| **DALYs (Disability-Adjusted Life Years)** | **Global** | **Male** | **Age-standardized** | **Low back pain** | **Rate** | **2013** | **253.26857** | **345.058786** | **173.575516** |
| **DALYs (Disability-Adjusted Life Years)** | **Global** | **Both** | **Age-standardized** | **Low back pain** | **Rate** | **2013** | **334.205514** | **451.579837** | **230.401461** |
| **Prevalence** | **Global** | **Male** | **Age-standardized** | **Low back pain** | **Rate** | **2013** | **2303.43207** | **2684.35955** | **1933.43142** |
| **Prevalence** | **Global** | **Both** | **Age-standardized** | **Low back pain** | **Rate** | **2013** | **3069.47626** | **3570.02181** | **2603.19004** |
| **DALYs (Disability-Adjusted Life Years)** | **Global** | **Female** | **Age-standardized** | **Low back pain** | **Rate** | **2013** | **406.241788** | **546.78654** | **282.375763** |
| **Prevalence** | **Global** | **Female** | **Age-standardized** | **Low back pain** | **Rate** | **2013** | **3751.27825** | **4340.30239** | **3206.06245** |
| **Incidence** | **Global** | **Female** | **Age-standardized** | **Low back pain** | **Rate** | **2013** | **1487.25022** | **1743.38534** | **1240.40535** |
| **Incidence** | **Global** | **Male** | **Age-standardized** | **Low back pain** | **Rate** | **2013** | **960.396532** | **1128.94215** | **796.061064** |
| **Incidence** | **Global** | **Both** | **Age-standardized** | **Low back pain** | **Rate** | **2013** | **1239.15073** | **1452.44525** | **1033.98183** |
| **DALYs (Disability-Adjusted Life Years)** | **Global** | **Male** | **Age-standardized** | **Low back pain** | **Rate** | **2014** | **253.00415** | **344.926148** | **172.977559** |
| **DALYs (Disability-Adjusted Life Years)** | **Global** | **Both** | **Age-standardized** | **Low back pain** | **Rate** | **2014** | **333.954512** | **451.127021** | **230.048423** |
| **Prevalence** | **Global** | **Male** | **Age-standardized** | **Low back pain** | **Rate** | **2014** | **2301.37483** | **2685.32625** | **1931.90835** |
| **Prevalence** | **Global** | **Both** | **Age-standardized** | **Low back pain** | **Rate** | **2014** | **3067.60774** | **3568.91004** | **2601.22282** |
| **DALYs (Disability-Adjusted Life Years)** | **Global** | **Female** | **Age-standardized** | **Low back pain** | **Rate** | **2014** | **406.023179** | **546.743776** | **282.195982** |
| **Prevalence** | **Global** | **Female** | **Age-standardized** | **Low back pain** | **Rate** | **2014** | **3749.77129** | **4342.63353** | **3201.36832** |
| **Incidence** | **Global** | **Female** | **Age-standardized** | **Low back pain** | **Rate** | **2014** | **1487.29846** | **1741.29994** | **1240.21089** |
| **Incidence** | **Global** | **Male** | **Age-standardized** | **Low back pain** | **Rate** | **2014** | **959.255549** | **1127.80337** | **794.421505** |
| **Incidence** | **Global** | **Both** | **Age-standardized** | **Low back pain** | **Rate** | **2014** | **1238.60162** | **1450.62482** | **1033.31499** |
| **DALYs (Disability-Adjusted Life Years)** | **Global** | **Male** | **Age-standardized** | **Low back pain** | **Rate** | **2015** | **252.593203** | **344.059549** | **172.606543** |
| **DALYs (Disability-Adjusted Life Years)** | **Global** | **Both** | **Age-standardized** | **Low back pain** | **Rate** | **2015** | **333.501182** | **450.744351** | **229.636667** |
| **Prevalence** | **Global** | **Male** | **Age-standardized** | **Low back pain** | **Rate** | **2015** | **2297.79246** | **2683.532** | **1928.92954** |
| **Prevalence** | **Global** | **Both** | **Age-standardized** | **Low back pain** | **Rate** | **2015** | **3063.79428** | **3565.1825** | **2596.99332** |
| **DALYs (Disability-Adjusted Life Years)** | **Global** | **Female** | **Age-standardized** | **Low back pain** | **Rate** | **2015** | **405.546713** | **546.426915** | **281.691169** |
| **Prevalence** | **Global** | **Female** | **Age-standardized** | **Low back pain** | **Rate** | **2015** | **3745.89027** | **4341.42627** | **3195.14448** |
| **Incidence** | **Global** | **Female** | **Age-standardized** | **Low back pain** | **Rate** | **2015** | **1486.38366** | **1738.12868** | **1239.3015** |
| **Incidence** | **Global** | **Male** | **Age-standardized** | **Low back pain** | **Rate** | **2015** | **957.716161** | **1126.12495** | **793.427799** |
| **Incidence** | **Global** | **Both** | **Age-standardized** | **Low back pain** | **Rate** | **2015** | **1237.36596** | **1448.34459** | **1032.08317** |
| **DALYs (Disability-Adjusted Life Years)** | **Global** | **Male** | **Age-standardized** | **Low back pain** | **Rate** | **2016** | **251.357737** | **342.240229** | **171.964838** |
| **DALYs (Disability-Adjusted Life Years)** | **Global** | **Both** | **Age-standardized** | **Low back pain** | **Rate** | **2016** | **332.479285** | **449.050114** | **228.973778** |
| **Prevalence** | **Global** | **Male** | **Age-standardized** | **Low back pain** | **Rate** | **2016** | **2287.08545** | **2673.23741** | **1918.84146** |
| **Prevalence** | **Global** | **Both** | **Age-standardized** | **Low back pain** | **Rate** | **2016** | **3055.0528** | **3557.23224** | **2590.22482** |
| **DALYs (Disability-Adjusted Life Years)** | **Global** | **Female** | **Age-standardized** | **Low back pain** | **Rate** | **2016** | **404.730841** | **544.957844** | **280.848576** |
| **Prevalence** | **Global** | **Female** | **Age-standardized** | **Low back pain** | **Rate** | **2016** | **3739.04907** | **4339.42981** | **3185.98192** |
| **Incidence** | **Global** | **Female** | **Age-standardized** | **Low back pain** | **Rate** | **2016** | **1484.11053** | **1732.08212** | **1237.57365** |
| **Incidence** | **Global** | **Male** | **Age-standardized** | **Low back pain** | **Rate** | **2016** | **953.311413** | **1120.78837** | **789.399242** |
| **Incidence** | **Global** | **Both** | **Age-standardized** | **Low back pain** | **Rate** | **2016** | **1234.05976** | **1443.98601** | **1029.82589** |
| **DALYs (Disability-Adjusted Life Years)** | **Global** | **Male** | **Age-standardized** | **Low back pain** | **Rate** | **2017** | **249.221875** | **339.081274** | **170.525719** |
| **DALYs (Disability-Adjusted Life Years)** | **Global** | **Both** | **Age-standardized** | **Low back pain** | **Rate** | **2017** | **330.861506** | **446.193373** | **227.814579** |
| **Prevalence** | **Global** | **Male** | **Age-standardized** | **Low back pain** | **Rate** | **2017** | **2268.58954** | **2653.28046** | **1901.4228** |
| **Prevalence** | **Global** | **Both** | **Age-standardized** | **Low back pain** | **Rate** | **2017** | **3041.07447** | **3544.38703** | **2579.19896** |
| **DALYs (Disability-Adjusted Life Years)** | **Global** | **Female** | **Age-standardized** | **Low back pain** | **Rate** | **2017** | **403.600726** | **542.773757** | **279.576334** |
| **Prevalence** | **Global** | **Female** | **Age-standardized** | **Low back pain** | **Rate** | **2017** | **3729.34255** | **4334.54493** | **3175.74664** |
| **Incidence** | **Global** | **Female** | **Age-standardized** | **Low back pain** | **Rate** | **2017** | **1480.79739** | **1725.9728** | **1234.25153** |
| **Incidence** | **Global** | **Male** | **Age-standardized** | **Low back pain** | **Rate** | **2017** | **945.779887** | **1112.25743** | **783.813997** |
| **Incidence** | **Global** | **Both** | **Age-standardized** | **Low back pain** | **Rate** | **2017** | **1228.71133** | **1438.01796** | **1025.38405** |
| **DALYs (Disability-Adjusted Life Years)** | **Global** | **Male** | **Age-standardized** | **Low back pain** | **Rate** | **2018** | **246.935113** | **335.227509** | **168.658254** |
| **DALYs (Disability-Adjusted Life Years)** | **Global** | **Both** | **Age-standardized** | **Low back pain** | **Rate** | **2018** | **329.012083** | **443.406458** | **226.64126** |
| **Prevalence** | **Global** | **Male** | **Age-standardized** | **Low back pain** | **Rate** | **2018** | **2248.69875** | **2632.45366** | **1882.72202** |
| **Prevalence** | **Global** | **Both** | **Age-standardized** | **Low back pain** | **Rate** | **2018** | **3024.92061** | **3529.70973** | **2564.00125** |
| **DALYs (Disability-Adjusted Life Years)** | **Global** | **Female** | **Age-standardized** | **Low back pain** | **Rate** | **2018** | **402.173083** | **540.223368** | **278.691** |
| **Prevalence** | **Global** | **Female** | **Age-standardized** | **Low back pain** | **Rate** | **2018** | **3716.82199** | **4321.0499** | **3164.12754** |
| **Incidence** | **Global** | **Female** | **Age-standardized** | **Low back pain** | **Rate** | **2018** | **1476.3503** | **1718.9214** | **1231.481** |
| **Incidence** | **Global** | **Male** | **Age-standardized** | **Low back pain** | **Rate** | **2018** | **937.692511** | **1103.06135** | **777.692593** |
| **Incidence** | **Global** | **Both** | **Age-standardized** | **Low back pain** | **Rate** | **2018** | **1222.4901** | **1430.65259** | **1019.97584** |
| **DALYs (Disability-Adjusted Life Years)** | **Global** | **Male** | **Age-standardized** | **Low back pain** | **Rate** | **2019** | **245.389881** | **332.694592** | **167.676332** |
| **DALYs (Disability-Adjusted Life Years)** | **Global** | **Both** | **Age-standardized** | **Low back pain** | **Rate** | **2019** | **327.560214** | **440.850731** | **225.581574** |
| **Prevalence** | **Global** | **Male** | **Age-standardized** | **Low back pain** | **Rate** | **2019** | **2235.46382** | **2619.3442** | **1869.25512** |
| **Prevalence** | **Global** | **Both** | **Age-standardized** | **Low back pain** | **Rate** | **2019** | **3012.39384** | **3518.53803** | **2551.13652** |
| **DALYs (Disability-Adjusted Life Years)** | **Global** | **Female** | **Age-standardized** | **Low back pain** | **Rate** | **2019** | **400.834117** | **537.374572** | **277.373382** |
| **Prevalence** | **Global** | **Female** | **Age-standardized** | **Low back pain** | **Rate** | **2019** | **3705.2071** | **4306.73424** | **3153.29539** |
| **Incidence** | **Global** | **Female** | **Age-standardized** | **Low back pain** | **Rate** | **2019** | **1472.18552** | **1713.57051** | **1229.11603** |
| **Incidence** | **Global** | **Male** | **Age-standardized** | **Low back pain** | **Rate** | **2019** | **932.408365** | **1096.8128** | **772.97289** |
| **Incidence** | **Global** | **Both** | **Age-standardized** | **Low back pain** | **Rate** | **2019** | **1217.74328** | **1424.94261** | **1015.6168** |
| **DALYs (Disability-Adjusted Life Years)** | **Global** | **Male** | **Age-standardized** | **Low back pain** | **Rate** | **2020** | **245.02275** | **333.891708** | **167.473314** |
| **DALYs (Disability-Adjusted Life Years)** | **Global** | **Both** | **Age-standardized** | **Low back pain** | **Rate** | **2020** | **325.839385** | **438.633339** | **224.205182** |
| **Prevalence** | **Global** | **Male** | **Age-standardized** | **Low back pain** | **Rate** | **2020** | **2234.2407** | **2617.32253** | **1873.59501** |
| **Prevalence** | **Global** | **Both** | **Age-standardized** | **Low back pain** | **Rate** | **2020** | **3000.15608** | **3506.16902** | **2542.67909** |
| **DALYs (Disability-Adjusted Life Years)** | **Global** | **Female** | **Age-standardized** | **Low back pain** | **Rate** | **2020** | **397.868321** | **534.181662** | **275.771701** |
| **Prevalence** | **Global** | **Female** | **Age-standardized** | **Low back pain** | **Rate** | **2020** | **3682.78869** | **4281.64933** | **3129.83452** |
| **Incidence** | **Global** | **Female** | **Age-standardized** | **Low back pain** | **Rate** | **2020** | **1462.31524** | **1701.68674** | **1219.8914** |
| **Incidence** | **Global** | **Male** | **Age-standardized** | **Low back pain** | **Rate** | **2020** | **932.215729** | **1097.56121** | **773.102664** |
| **Incidence** | **Global** | **Both** | **Age-standardized** | **Low back pain** | **Rate** | **2020** | **1212.50425** | **1418.94766** | **1010.00387** |
| **DALYs (Disability-Adjusted Life Years)** | **Global** | **Male** | **Age-standardized** | **Low back pain** | **Rate** | **2021** | **243.107779** | **329.8939** | **166.557623** |
| **DALYs (Disability-Adjusted Life Years)** | **Global** | **Both** | **Age-standardized** | **Low back pain** | **Rate** | **2021** | **324.520742** | **436.675992** | **223.530147** |
| **Prevalence** | **Global** | **Male** | **Age-standardized** | **Low back pain** | **Rate** | **2021** | **2218.68735** | **2595.664** | **1855.59833** |
| **Prevalence** | **Global** | **Both** | **Age-standardized** | **Low back pain** | **Rate** | **2021** | **2991.61674** | **3487.57868** | **2536.05835** |
| **DALYs (Disability-Adjusted Life Years)** | **Global** | **Female** | **Age-standardized** | **Low back pain** | **Rate** | **2021** | **396.932837** | **532.109655** | **275.246973** |
| **Prevalence** | **Global** | **Female** | **Age-standardized** | **Low back pain** | **Rate** | **2021** | **3679.09246** | **4277.94585** | **3129.33348** |
| **Incidence** | **Global** | **Male** | **Age-standardized** | **Low back pain** | **Rate** | **2021** | **925.894308** | **1092.82199** | **764.321553** |
| **Incidence** | **Global** | **Female** | **Age-standardized** | **Low back pain** | **Rate** | **2021** | **1462.09538** | **1700.20823** | **1218.24303** |
| **Incidence** | **Global** | **Both** | **Age-standardized** | **Low back pain** | **Rate** | **2021** | **1209.68239** | **1413.40673** | **1005.26742** |

Appendix 5: National analysis of incidence, prevalence, and DALYs of low back pain in postmenopausal women in 2021

| **measure** | **location** | **sex** | **age** | **cause** | **metric** | **year** | **val** | **upper** | **lower** |
| --- | --- | --- | --- | --- | --- | --- | --- | --- | --- |
| **DALYs (Disability-Adjusted Life Years)** | **Afghanistan** | **Female** | **55+ years** | **Low back pain** | **Number** | **2021** | **15483.8614** | **21138.4965** | **10756.3712** |
| **Prevalence** | **Afghanistan** | **Female** | **55+ years** | **Low back pain** | **Number** | **2021** | **147119.54** | **176534.554** | **120764.499** |
| **Incidence** | **Afghanistan** | **Female** | **55+ years** | **Low back pain** | **Number** | **2021** | **59547.2446** | **71146.6032** | **48595.3867** |
| **DALYs (Disability-Adjusted Life Years)** | **Albania** | **Female** | **55+ years** | **Low back pain** | **Number** | **2021** | **14761.6988** | **20386.6585** | **10154.3689** |
| **Prevalence** | **Albania** | **Female** | **55+ years** | **Low back pain** | **Number** | **2021** | **134977.174** | **156792.989** | **113951.031** |
| **Incidence** | **Albania** | **Female** | **55+ years** | **Low back pain** | **Number** | **2021** | **47571.7646** | **56109.5886** | **39320.9336** |
| **DALYs (Disability-Adjusted Life Years)** | **Algeria** | **Female** | **55+ years** | **Low back pain** | **Number** | **2021** | **65791.0474** | **90254.0231** | **44598.7815** |
| **Prevalence** | **Algeria** | **Female** | **55+ years** | **Low back pain** | **Number** | **2021** | **610181.112** | **729275.203** | **496561.55** |
| **Incidence** | **Algeria** | **Female** | **55+ years** | **Low back pain** | **Number** | **2021** | **247796.431** | **297313.017** | **202197.309** |
| **DALYs (Disability-Adjusted Life Years)** | **American Samoa** | **Female** | **55+ years** | **Low back pain** | **Number** | **2021** | **90.2734069** | **124.090661** | **62.0156208** |
| **Prevalence** | **American Samoa** | **Female** | **55+ years** | **Low back pain** | **Number** | **2021** | **841.899269** | **1009.04888** | **689.90339** |
| **Incidence** | **American Samoa** | **Female** | **55+ years** | **Low back pain** | **Number** | **2021** | **340.06362** | **410.270788** | **279.221846** |
| **DALYs (Disability-Adjusted Life Years)** | **Andorra** | **Female** | **55+ years** | **Low back pain** | **Number** | **2021** | **343.114968** | **468.560486** | **235.059323** |
| **Prevalence** | **Andorra** | **Female** | **55+ years** | **Low back pain** | **Number** | **2021** | **3163.78439** | **3694.74304** | **2641.26611** |
| **Incidence** | **Andorra** | **Female** | **55+ years** | **Low back pain** | **Number** | **2021** | **1292.34702** | **1521.78591** | **1091.95259** |
| **DALYs (Disability-Adjusted Life Years)** | **Angola** | **Female** | **55+ years** | **Low back pain** | **Number** | **2021** | **24492.0404** | **33771.6815** | **16867.6888** |
| **Prevalence** | **Angola** | **Female** | **55+ years** | **Low back pain** | **Number** | **2021** | **224293.376** | **268983.269** | **182938.298** |
| **Incidence** | **Angola** | **Female** | **55+ years** | **Low back pain** | **Number** | **2021** | **90829.4429** | **108814.663** | **73644.8809** |
| **DALYs (Disability-Adjusted Life Years)** | **Antigua and Barbuda** | **Female** | **55+ years** | **Low back pain** | **Number** | **2021** | **177.786792** | **243.043748** | **122.2131** |
| **Prevalence** | **Antigua and Barbuda** | **Female** | **55+ years** | **Low back pain** | **Number** | **2021** | **1631.28743** | **1948.79263** | **1326.06425** |
| **Incidence** | **Antigua and Barbuda** | **Female** | **55+ years** | **Low back pain** | **Number** | **2021** | **670.345214** | **807.135554** | **549.297871** |
| **DALYs (Disability-Adjusted Life Years)** | **Argentina** | **Female** | **55+ years** | **Low back pain** | **Number** | **2021** | **151730.687** | **205789.846** | **104762.051** |
| **Prevalence** | **Argentina** | **Female** | **55+ years** | **Low back pain** | **Number** | **2021** | **1398466.72** | **1621788.26** | **1174055.17** |
| **Incidence** | **Argentina** | **Female** | **55+ years** | **Low back pain** | **Number** | **2021** | **551861.793** | **652352.108** | **457580.661** |
| **DALYs (Disability-Adjusted Life Years)** | **Armenia** | **Female** | **55+ years** | **Low back pain** | **Number** | **2021** | **13709.5966** | **18829.4405** | **9524.16834** |
| **Prevalence** | **Armenia** | **Female** | **55+ years** | **Low back pain** | **Number** | **2021** | **126385.092** | **147294.936** | **106805.145** |
| **Incidence** | **Armenia** | **Female** | **55+ years** | **Low back pain** | **Number** | **2021** | **47454.8834** | **56380.1658** | **39008.1846** |
| **DALYs (Disability-Adjusted Life Years)** | **Australia** | **Female** | **55+ years** | **Low back pain** | **Number** | **2021** | **125915.444** | **168145.76** | **86293.1121** |
| **Prevalence** | **Australia** | **Female** | **55+ years** | **Low back pain** | **Number** | **2021** | **1163558.62** | **1345777.22** | **981875.105** |
| **Incidence** | **Australia** | **Female** | **55+ years** | **Low back pain** | **Number** | **2021** | **450806.114** | **526204.807** | **374190.091** |
| **DALYs (Disability-Adjusted Life Years)** | **Austria** | **Female** | **55+ years** | **Low back pain** | **Number** | **2021** | **40109.7183** | **54297.8845** | **27864.096** |
| **Prevalence** | **Austria** | **Female** | **55+ years** | **Low back pain** | **Number** | **2021** | **371149.696** | **428010.934** | **315401.703** |
| **Incidence** | **Austria** | **Female** | **55+ years** | **Low back pain** | **Number** | **2021** | **155094.175** | **182056.804** | **129544.986** |
| **DALYs (Disability-Adjusted Life Years)** | **Azerbaijan** | **Female** | **55+ years** | **Low back pain** | **Number** | **2021** | **29334.7283** | **41438.2299** | **20129.6653** |
| **Prevalence** | **Azerbaijan** | **Female** | **55+ years** | **Low back pain** | **Number** | **2021** | **266992.097** | **320303.159** | **220698.1** |
| **Incidence** | **Azerbaijan** | **Female** | **55+ years** | **Low back pain** | **Number** | **2021** | **103713.165** | **123592.362** | **84972.5146** |
| **DALYs (Disability-Adjusted Life Years)** | **Bahamas** | **Female** | **55+ years** | **Low back pain** | **Number** | **2021** | **707.033308** | **981.244075** | **478.622601** |
| **Prevalence** | **Bahamas** | **Female** | **55+ years** | **Low back pain** | **Number** | **2021** | **6447.7251** | **7783.12755** | **5253.73056** |
| **Incidence** | **Bahamas** | **Female** | **55+ years** | **Low back pain** | **Number** | **2021** | **2651.73583** | **3209.1928** | **2169.42417** |
| **DALYs (Disability-Adjusted Life Years)** | **Bahrain** | **Female** | **55+ years** | **Low back pain** | **Number** | **2021** | **1363.28266** | **1863.91598** | **918.71782** |
| **Prevalence** | **Bahrain** | **Female** | **55+ years** | **Low back pain** | **Number** | **2021** | **12759.2342** | **15419.7437** | **10262.7545** |
| **Incidence** | **Bahrain** | **Female** | **55+ years** | **Low back pain** | **Number** | **2021** | **5246.3077** | **6338.46987** | **4216.90818** |
| **DALYs (Disability-Adjusted Life Years)** | **Bangladesh** | **Female** | **55+ years** | **Low back pain** | **Number** | **2021** | **338315.242** | **470134.535** | **235019.497** |
| **Prevalence** | **Bangladesh** | **Female** | **55+ years** | **Low back pain** | **Number** | **2021** | **3138774.4** | **3655650.3** | **2613380.15** |
| **Incidence** | **Bangladesh** | **Female** | **55+ years** | **Low back pain** | **Number** | **2021** | **1145745** | **1381412.43** | **930920.62** |
| **DALYs (Disability-Adjusted Life Years)** | **Barbados** | **Female** | **55+ years** | **Low back pain** | **Number** | **2021** | **913.002306** | **1233.12095** | **619.712527** |
| **Prevalence** | **Barbados** | **Female** | **55+ years** | **Low back pain** | **Number** | **2021** | **8395.30906** | **9867.0814** | **6922.84467** |
| **Incidence** | **Barbados** | **Female** | **55+ years** | **Low back pain** | **Number** | **2021** | **3427.7257** | **4064.06912** | **2828.91129** |
| **DALYs (Disability-Adjusted Life Years)** | **Belarus** | **Female** | **55+ years** | **Low back pain** | **Number** | **2021** | **61079.3319** | **81639.9696** | **43379.1493** |
| **Prevalence** | **Belarus** | **Female** | **55+ years** | **Low back pain** | **Number** | **2021** | **566379.374** | **652953.266** | **483989.061** |
| **Incidence** | **Belarus** | **Female** | **55+ years** | **Low back pain** | **Number** | **2021** | **201306.822** | **235755.935** | **164828.774** |
| **DALYs (Disability-Adjusted Life Years)** | **Belgium** | **Female** | **55+ years** | **Low back pain** | **Number** | **2021** | **55715.405** | **74893.0352** | **39011.7867** |
| **Prevalence** | **Belgium** | **Female** | **55+ years** | **Low back pain** | **Number** | **2021** | **519839.845** | **603507.786** | **434571.62** |
| **Incidence** | **Belgium** | **Female** | **55+ years** | **Low back pain** | **Number** | **2021** | **209151.786** | **242758.381** | **176741.908** |
| **DALYs (Disability-Adjusted Life Years)** | **Belize** | **Female** | **55+ years** | **Low back pain** | **Number** | **2021** | **457.454451** | **630.467994** | **305.601343** |
| **Prevalence** | **Belize** | **Female** | **55+ years** | **Low back pain** | **Number** | **2021** | **4169.08921** | **5048.13958** | **3384.83781** |
| **Incidence** | **Belize** | **Female** | **55+ years** | **Low back pain** | **Number** | **2021** | **1696.28623** | **2037.63757** | **1377.85137** |
| **DALYs (Disability-Adjusted Life Years)** | **Benin** | **Female** | **55+ years** | **Low back pain** | **Number** | **2021** | **9631.50047** | **13189.8987** | **6545.63999** |
| **Prevalence** | **Benin** | **Female** | **55+ years** | **Low back pain** | **Number** | **2021** | **88790.667** | **106334.685** | **73435.7485** |
| **Incidence** | **Benin** | **Female** | **55+ years** | **Low back pain** | **Number** | **2021** | **35840.3827** | **43671.7315** | **29211.6152** |
| **DALYs (Disability-Adjusted Life Years)** | **Bermuda** | **Female** | **55+ years** | **Low back pain** | **Number** | **2021** | **241.338185** | **327.511878** | **165.684773** |
| **Prevalence** | **Bermuda** | **Female** | **55+ years** | **Low back pain** | **Number** | **2021** | **2210.36195** | **2599.12398** | **1849.95114** |
| **Incidence** | **Bermuda** | **Female** | **55+ years** | **Low back pain** | **Number** | **2021** | **895.872998** | **1064.22422** | **739.707942** |
| **DALYs (Disability-Adjusted Life Years)** | **Bhutan** | **Female** | **55+ years** | **Low back pain** | **Number** | **2021** | **1459.31848** | **1988.54919** | **1008.75565** |
| **Prevalence** | **Bhutan** | **Female** | **55+ years** | **Low back pain** | **Number** | **2021** | **13484.0899** | **15924.6126** | **11283.4854** |
| **Incidence** | **Bhutan** | **Female** | **55+ years** | **Low back pain** | **Number** | **2021** | **4967.44312** | **5963.9155** | **4063.2339** |
| **DALYs (Disability-Adjusted Life Years)** | **Bolivia (Plurinational State of)** | **Female** | **55+ years** | **Low back pain** | **Number** | **2021** | **14687.4588** | **19791.6869** | **10074.6872** |
| **Prevalence** | **Bolivia (Plurinational State of)** | **Female** | **55+ years** | **Low back pain** | **Number** | **2021** | **135538.149** | **161586.184** | **110720.166** |
| **Incidence** | **Bolivia (Plurinational State of)** | **Female** | **55+ years** | **Low back pain** | **Number** | **2021** | **55510.9536** | **66322.0686** | **45643.8515** |
| **DALYs (Disability-Adjusted Life Years)** | **Bosnia and Herzegovina** | **Female** | **55+ years** | **Low back pain** | **Number** | **2021** | **20371.2599** | **27690.9005** | **14055.1513** |
| **Prevalence** | **Bosnia and Herzegovina** | **Female** | **55+ years** | **Low back pain** | **Number** | **2021** | **189853.102** | **221083.329** | **161182.32** |
| **Incidence** | **Bosnia and Herzegovina** | **Female** | **55+ years** | **Low back pain** | **Number** | **2021** | **69090.9605** | **81186.1144** | **57382.8984** |
| **DALYs (Disability-Adjusted Life Years)** | **Botswana** | **Female** | **55+ years** | **Low back pain** | **Number** | **2021** | **3038.45453** | **4172.13904** | **2069.82888** |
| **Prevalence** | **Botswana** | **Female** | **55+ years** | **Low back pain** | **Number** | **2021** | **28115.9078** | **33506.4188** | **23096.2711** |
| **Incidence** | **Botswana** | **Female** | **55+ years** | **Low back pain** | **Number** | **2021** | **11365.6178** | **13667.0158** | **9188.70273** |
| **DALYs (Disability-Adjusted Life Years)** | **Brazil** | **Female** | **55+ years** | **Low back pain** | **Number** | **2021** | **633966.266** | **861703.099** | **443826.724** |
| **Prevalence** | **Brazil** | **Female** | **55+ years** | **Low back pain** | **Number** | **2021** | **5919489.51** | **6973892.97** | **4959440.11** |
| **Incidence** | **Brazil** | **Female** | **55+ years** | **Low back pain** | **Number** | **2021** | **2260143.23** | **2648210.95** | **1864846.71** |
| **DALYs (Disability-Adjusted Life Years)** | **Brunei Darussalam** | **Female** | **55+ years** | **Low back pain** | **Number** | **2021** | **704.951865** | **969.726083** | **484.460365** |
| **Prevalence** | **Brunei Darussalam** | **Female** | **55+ years** | **Low back pain** | **Number** | **2021** | **6399.51757** | **7697.78873** | **5229.82353** |
| **Incidence** | **Brunei Darussalam** | **Female** | **55+ years** | **Low back pain** | **Number** | **2021** | **2652.46153** | **3183.70626** | **2139.98551** |
| **DALYs (Disability-Adjusted Life Years)** | **Bulgaria** | **Female** | **55+ years** | **Low back pain** | **Number** | **2021** | **47154.8013** | **63957.0968** | **32666.3458** |
| **Prevalence** | **Bulgaria** | **Female** | **55+ years** | **Low back pain** | **Number** | **2021** | **438821.519** | **508191.231** | **375800.343** |
| **Incidence** | **Bulgaria** | **Female** | **55+ years** | **Low back pain** | **Number** | **2021** | **158344.704** | **187881.069** | **131405.409** |
| **DALYs (Disability-Adjusted Life Years)** | **Burkina Faso** | **Female** | **55+ years** | **Low back pain** | **Number** | **2021** | **17829.4523** | **24931.9701** | **12275.2914** |
| **Prevalence** | **Burkina Faso** | **Female** | **55+ years** | **Low back pain** | **Number** | **2021** | **162646.783** | **192516.335** | **132607.846** |
| **Incidence** | **Burkina Faso** | **Female** | **55+ years** | **Low back pain** | **Number** | **2021** | **65655.5452** | **78556.2191** | **52763.0825** |
| **DALYs (Disability-Adjusted Life Years)** | **Burundi** | **Female** | **55+ years** | **Low back pain** | **Number** | **2021** | **9546.28767** | **13220.6904** | **6513.20273** |
| **Prevalence** | **Burundi** | **Female** | **55+ years** | **Low back pain** | **Number** | **2021** | **86820.3001** | **104430.892** | **71726.277** |
| **Incidence** | **Burundi** | **Female** | **55+ years** | **Low back pain** | **Number** | **2021** | **34479.3071** | **41640.3462** | **28291.3378** |
| **DALYs (Disability-Adjusted Life Years)** | **Cabo Verde** | **Female** | **55+ years** | **Low back pain** | **Number** | **2021** | **892.5429** | **1207.21404** | **613.104445** |
| **Prevalence** | **Cabo Verde** | **Female** | **55+ years** | **Low back pain** | **Number** | **2021** | **8207.33295** | **9797.10391** | **6687.26086** |
| **Incidence** | **Cabo Verde** | **Female** | **55+ years** | **Low back pain** | **Number** | **2021** | **3411.27699** | **4083.55488** | **2775.16214** |
| **DALYs (Disability-Adjusted Life Years)** | **Cambodia** | **Female** | **55+ years** | **Low back pain** | **Number** | **2021** | **25718.1381** | **35769.9025** | **17509.5058** |
| **Prevalence** | **Cambodia** | **Female** | **55+ years** | **Low back pain** | **Number** | **2021** | **236167.655** | **281758.375** | **193953.962** |
| **Incidence** | **Cambodia** | **Female** | **55+ years** | **Low back pain** | **Number** | **2021** | **94479.5184** | **113358.316** | **76600.044** |
| **DALYs (Disability-Adjusted Life Years)** | **Cameroon** | **Female** | **55+ years** | **Low back pain** | **Number** | **2021** | **23645.9515** | **32584.9013** | **15842.163** |
| **Prevalence** | **Cameroon** | **Female** | **55+ years** | **Low back pain** | **Number** | **2021** | **217112.526** | **257601.138** | **176105.698** |
| **Incidence** | **Cameroon** | **Female** | **55+ years** | **Low back pain** | **Number** | **2021** | **87319.5526** | **105261.98** | **71324.3775** |
| **DALYs (Disability-Adjusted Life Years)** | **Canada** | **Female** | **55+ years** | **Low back pain** | **Number** | **2021** | **144080.724** | **196162.542** | **98524.9592** |
| **Prevalence** | **Canada** | **Female** | **55+ years** | **Low back pain** | **Number** | **2021** | **1326485.22** | **1551688.83** | **1124274.75** |
| **Incidence** | **Canada** | **Female** | **55+ years** | **Low back pain** | **Number** | **2021** | **549338.953** | **652224.498** | **458089.924** |
| **DALYs (Disability-Adjusted Life Years)** | **Central African Republic** | **Female** | **55+ years** | **Low back pain** | **Number** | **2021** | **4496.58756** | **6322.75992** | **3046.81056** |
| **Prevalence** | **Central African Republic** | **Female** | **55+ years** | **Low back pain** | **Number** | **2021** | **41477.1532** | **50339.3872** | **33451.7029** |
| **Incidence** | **Central African Republic** | **Female** | **55+ years** | **Low back pain** | **Number** | **2021** | **16780.5102** | **20457.3567** | **13420.819** |
| **DALYs (Disability-Adjusted Life Years)** | **Chad** | **Female** | **55+ years** | **Low back pain** | **Number** | **2021** | **9954.9872** | **14044.1551** | **6747.1028** |
| **Prevalence** | **Chad** | **Female** | **55+ years** | **Low back pain** | **Number** | **2021** | **91629.4105** | **110391.068** | **74100.5784** |
| **Incidence** | **Chad** | **Female** | **55+ years** | **Low back pain** | **Number** | **2021** | **36064.1016** | **43205.9367** | **29522.794** |
| **DALYs (Disability-Adjusted Life Years)** | **Chile** | **Female** | **55+ years** | **Low back pain** | **Number** | **2021** | **71646.6493** | **96759.0313** | **49423.3453** |
| **Prevalence** | **Chile** | **Female** | **55+ years** | **Low back pain** | **Number** | **2021** | **663648.782** | **780573.832** | **560625.595** |
| **Incidence** | **Chile** | **Female** | **55+ years** | **Low back pain** | **Number** | **2021** | **262192.348** | **308856.616** | **217055.987** |
| **DALYs (Disability-Adjusted Life Years)** | **China** | **Female** | **55+ years** | **Low back pain** | **Number** | **2021** | **3781185.72** | **5125842.56** | **2558951.35** |
| **Prevalence** | **China** | **Female** | **55+ years** | **Low back pain** | **Number** | **2021** | **34596953.3** | **40586981** | **28898107.8** |
| **Incidence** | **China** | **Female** | **55+ years** | **Low back pain** | **Number** | **2021** | **14351285.2** | **16928417.8** | **11877513.7** |
| **DALYs (Disability-Adjusted Life Years)** | **Colombia** | **Female** | **55+ years** | **Low back pain** | **Number** | **2021** | **115992.683** | **159225.699** | **78811.7868** |
| **Prevalence** | **Colombia** | **Female** | **55+ years** | **Low back pain** | **Number** | **2021** | **1062053.56** | **1263101.16** | **891580.95** |
| **Incidence** | **Colombia** | **Female** | **55+ years** | **Low back pain** | **Number** | **2021** | **415528.592** | **488612.053** | **344617.076** |
| **DALYs (Disability-Adjusted Life Years)** | **Comoros** | **Female** | **55+ years** | **Low back pain** | **Number** | **2021** | **1072.49517** | **1477.61524** | **733.403127** |
| **Prevalence** | **Comoros** | **Female** | **55+ years** | **Low back pain** | **Number** | **2021** | **9844.62712** | **11718.449** | **8181.67337** |
| **Incidence** | **Comoros** | **Female** | **55+ years** | **Low back pain** | **Number** | **2021** | **3955.36839** | **4791.4956** | **3227.39648** |
| **DALYs (Disability-Adjusted Life Years)** | **Congo** | **Female** | **55+ years** | **Low back pain** | **Number** | **2021** | **4896.19349** | **6773.77337** | **3292.7651** |
| **Prevalence** | **Congo** | **Female** | **55+ years** | **Low back pain** | **Number** | **2021** | **45055.3106** | **53857.6819** | **36313.6949** |
| **Incidence** | **Congo** | **Female** | **55+ years** | **Low back pain** | **Number** | **2021** | **18477.3936** | **22319.7739** | **15200.0227** |
| **DALYs (Disability-Adjusted Life Years)** | **Cook Islands** | **Female** | **55+ years** | **Low back pain** | **Number** | **2021** | **52.9676584** | **71.3946539** | **36.4513757** |
| **Prevalence** | **Cook Islands** | **Female** | **55+ years** | **Low back pain** | **Number** | **2021** | **495.091422** | **578.353006** | **411.849766** |
| **Incidence** | **Cook Islands** | **Female** | **55+ years** | **Low back pain** | **Number** | **2021** | **197.697076** | **235.133282** | **162.84122** |
| **DALYs (Disability-Adjusted Life Years)** | **Costa Rica** | **Female** | **55+ years** | **Low back pain** | **Number** | **2021** | **10785.6305** | **14762.0029** | **7431.82454** |
| **Prevalence** | **Costa Rica** | **Female** | **55+ years** | **Low back pain** | **Number** | **2021** | **99341.715** | **117635.386** | **82825.5931** |
| **Incidence** | **Costa Rica** | **Female** | **55+ years** | **Low back pain** | **Number** | **2021** | **39635.0318** | **47321.8825** | **32648.2383** |
| **DALYs (Disability-Adjusted Life Years)** | **Croatia** | **Female** | **55+ years** | **Low back pain** | **Number** | **2021** | **28101.1566** | **37789.6556** | **19753.1833** |
| **Prevalence** | **Croatia** | **Female** | **55+ years** | **Low back pain** | **Number** | **2021** | **262600.411** | **304552.788** | **221045.358** |
| **Incidence** | **Croatia** | **Female** | **55+ years** | **Low back pain** | **Number** | **2021** | **95820.8232** | **112302.197** | **78694.2055** |
| **DALYs (Disability-Adjusted Life Years)** | **Cuba** | **Female** | **55+ years** | **Low back pain** | **Number** | **2021** | **32677.8281** | **43264.2101** | **23135.9965** |
| **Prevalence** | **Cuba** | **Female** | **55+ years** | **Low back pain** | **Number** | **2021** | **300331.905** | **340216.704** | **263266.734** |
| **Incidence** | **Cuba** | **Female** | **55+ years** | **Low back pain** | **Number** | **2021** | **123563.65** | **141591.274** | **104736.049** |
| **DALYs (Disability-Adjusted Life Years)** | **Cyprus** | **Female** | **55+ years** | **Low back pain** | **Number** | **2021** | **4959.34493** | **6652.9646** | **3368.06689** |
| **Prevalence** | **Cyprus** | **Female** | **55+ years** | **Low back pain** | **Number** | **2021** | **45479.1062** | **53283.9419** | **38247.0412** |
| **Incidence** | **Cyprus** | **Female** | **55+ years** | **Low back pain** | **Number** | **2021** | **18579.3965** | **21897.6439** | **15465.3534** |
| **DALYs (Disability-Adjusted Life Years)** | **Czechia** | **Female** | **55+ years** | **Low back pain** | **Number** | **2021** | **71208.056** | **97077.823** | **49645.7107** |
| **Prevalence** | **Czechia** | **Female** | **55+ years** | **Low back pain** | **Number** | **2021** | **668458.314** | **771395.459** | **568536.695** |
| **Incidence** | **Czechia** | **Female** | **55+ years** | **Low back pain** | **Number** | **2021** | **230572.893** | **269954.304** | **191524.522** |
| **DALYs (Disability-Adjusted Life Years)** | **C么te d'Ivoire** | **Female** | **55+ years** | **Low back pain** | **Number** | **2021** | **19222.1941** | **26935.28** | **13249.3175** |
| **Prevalence** | **C么te d'Ivoire** | **Female** | **55+ years** | **Low back pain** | **Number** | **2021** | **176305.762** | **211699.688** | **143354.428** |
| **Incidence** | **C么te d'Ivoire** | **Female** | **55+ years** | **Low back pain** | **Number** | **2021** | **70955.8259** | **84482.0552** | **56508.7929** |
| **DALYs (Disability-Adjusted Life Years)** | **Democratic People's Republic of Korea** | **Female** | **55+ years** | **Low back pain** | **Number** | **2021** | **77989.1145** | **106075.304** | **53570.7716** |
| **Prevalence** | **Democratic People's Republic of Korea** | **Female** | **55+ years** | **Low back pain** | **Number** | **2021** | **710630.265** | **848052.902** | **592797.3** |
| **Incidence** | **Democratic People's Republic of Korea** | **Female** | **55+ years** | **Low back pain** | **Number** | **2021** | **281001.921** | **330353.989** | **231795.935** |
| **DALYs (Disability-Adjusted Life Years)** | **Democratic Republic of the Congo** | **Female** | **55+ years** | **Low back pain** | **Number** | **2021** | **76068.8734** | **104887.134** | **50964.1251** |
| **Prevalence** | **Democratic Republic of the Congo** | **Female** | **55+ years** | **Low back pain** | **Number** | **2021** | **699622.991** | **837608.863** | **569592.457** |
| **Incidence** | **Democratic Republic of the Congo** | **Female** | **55+ years** | **Low back pain** | **Number** | **2021** | **282118.447** | **338640.117** | **230157.572** |
| **DALYs (Disability-Adjusted Life Years)** | **Denmark** | **Female** | **55+ years** | **Low back pain** | **Number** | **2021** | **32088.2389** | **44657.5128** | **21853.7272** |
| **Prevalence** | **Denmark** | **Female** | **55+ years** | **Low back pain** | **Number** | **2021** | **294660.093** | **346072.005** | **240766.727** |
| **Incidence** | **Denmark** | **Female** | **55+ years** | **Low back pain** | **Number** | **2021** | **104005.546** | **129050.56** | **79941.5145** |
| **DALYs (Disability-Adjusted Life Years)** | **Djibouti** | **Female** | **55+ years** | **Low back pain** | **Number** | **2021** | **1056.73034** | **1475.19064** | **714.23002** |
| **Prevalence** | **Djibouti** | **Female** | **55+ years** | **Low back pain** | **Number** | **2021** | **9599.2845** | **11480.722** | **7765.64798** |
| **Incidence** | **Djibouti** | **Female** | **55+ years** | **Low back pain** | **Number** | **2021** | **3950.68654** | **4764.10156** | **3148.88382** |
| **DALYs (Disability-Adjusted Life Years)** | **Dominica** | **Female** | **55+ years** | **Low back pain** | **Number** | **2021** | **141.55884** | **194.347848** | **97.0322937** |
| **Prevalence** | **Dominica** | **Female** | **55+ years** | **Low back pain** | **Number** | **2021** | **1303.31844** | **1538.76821** | **1070.43018** |
| **Incidence** | **Dominica** | **Female** | **55+ years** | **Low back pain** | **Number** | **2021** | **530.73825** | **636.214243** | **440.213639** |
| **DALYs (Disability-Adjusted Life Years)** | **Dominican Republic** | **Female** | **55+ years** | **Low back pain** | **Number** | **2021** | **16306.0065** | **22126.4523** | **11189.3076** |
| **Prevalence** | **Dominican Republic** | **Female** | **55+ years** | **Low back pain** | **Number** | **2021** | **149719.524** | **177033.702** | **124701.436** |
| **Incidence** | **Dominican Republic** | **Female** | **55+ years** | **Low back pain** | **Number** | **2021** | **60481.6409** | **71788.8451** | **49167.3443** |
| **DALYs (Disability-Adjusted Life Years)** | **Ecuador** | **Female** | **55+ years** | **Low back pain** | **Number** | **2021** | **24001.632** | **31869.9433** | **16715.7242** |
| **Prevalence** | **Ecuador** | **Female** | **55+ years** | **Low back pain** | **Number** | **2021** | **221302.393** | **256970.721** | **186175.348** |
| **Incidence** | **Ecuador** | **Female** | **55+ years** | **Low back pain** | **Number** | **2021** | **93198.7202** | **109632.022** | **77243.6633** |
| **DALYs (Disability-Adjusted Life Years)** | **Egypt** | **Female** | **55+ years** | **Low back pain** | **Number** | **2021** | **114659.781** | **161443.529** | **77128.3052** |
| **Prevalence** | **Egypt** | **Female** | **55+ years** | **Low back pain** | **Number** | **2021** | **1054993.59** | **1278623.61** | **859339.82** |
| **Incidence** | **Egypt** | **Female** | **55+ years** | **Low back pain** | **Number** | **2021** | **427789.879** | **517038.183** | **339927.535** |
| **DALYs (Disability-Adjusted Life Years)** | **El Salvador** | **Female** | **55+ years** | **Low back pain** | **Number** | **2021** | **12600.4452** | **16975.0386** | **8615.25596** |
| **Prevalence** | **El Salvador** | **Female** | **55+ years** | **Low back pain** | **Number** | **2021** | **116146.97** | **137217.002** | **97051.4101** |
| **Incidence** | **El Salvador** | **Female** | **55+ years** | **Low back pain** | **Number** | **2021** | **46167.2835** | **54548.3852** | **38464.053** |
| **DALYs (Disability-Adjusted Life Years)** | **Equatorial Guinea** | **Female** | **55+ years** | **Low back pain** | **Number** | **2021** | **1018.55361** | **1403.60654** | **695.328598** |
| **Prevalence** | **Equatorial Guinea** | **Female** | **55+ years** | **Low back pain** | **Number** | **2021** | **9414.0189** | **11308.651** | **7691.86658** |
| **Incidence** | **Equatorial Guinea** | **Female** | **55+ years** | **Low back pain** | **Number** | **2021** | **3842.15498** | **4621.10667** | **3094.3332** |
| **DALYs (Disability-Adjusted Life Years)** | **Eritrea** | **Female** | **55+ years** | **Low back pain** | **Number** | **2021** | **5856.55648** | **8023.3576** | **3948.86183** |
| **Prevalence** | **Eritrea** | **Female** | **55+ years** | **Low back pain** | **Number** | **2021** | **53634.7464** | **64492.4787** | **43484.4862** |
| **Incidence** | **Eritrea** | **Female** | **55+ years** | **Low back pain** | **Number** | **2021** | **22135.0922** | **26770.8** | **17726.7314** |
| **DALYs (Disability-Adjusted Life Years)** | **Estonia** | **Female** | **55+ years** | **Low back pain** | **Number** | **2021** | **9385.98153** | **12487.3344** | **6557.65782** |
| **Prevalence** | **Estonia** | **Female** | **55+ years** | **Low back pain** | **Number** | **2021** | **87692.4655** | **100955.519** | **75127.0645** |
| **Incidence** | **Estonia** | **Female** | **55+ years** | **Low back pain** | **Number** | **2021** | **30913.9871** | **36037.0225** | **25636.7363** |
| **DALYs (Disability-Adjusted Life Years)** | **Eswatini** | **Female** | **55+ years** | **Low back pain** | **Number** | **2021** | **1117.55912** | **1543.15469** | **759.019531** |
| **Prevalence** | **Eswatini** | **Female** | **55+ years** | **Low back pain** | **Number** | **2021** | **10375.3672** | **12436.7988** | **8494.4766** |
| **Incidence** | **Eswatini** | **Female** | **55+ years** | **Low back pain** | **Number** | **2021** | **4315.70167** | **5125.6809** | **3523.0476** |
| **DALYs (Disability-Adjusted Life Years)** | **Ethiopia** | **Female** | **55+ years** | **Low back pain** | **Number** | **2021** | **83059.1216** | **113114.773** | **57331.1798** |
| **Prevalence** | **Ethiopia** | **Female** | **55+ years** | **Low back pain** | **Number** | **2021** | **767659.821** | **906953.27** | **639867.645** |
| **Incidence** | **Ethiopia** | **Female** | **55+ years** | **Low back pain** | **Number** | **2021** | **311174.645** | **367629.3** | **256313.166** |
| **DALYs (Disability-Adjusted Life Years)** | **Fiji** | **Female** | **55+ years** | **Low back pain** | **Number** | **2021** | **1505.8326** | **2085.35461** | **1022.16475** |
| **Prevalence** | **Fiji** | **Female** | **55+ years** | **Low back pain** | **Number** | **2021** | **13972.0872** | **16841.7237** | **11369.4239** |
| **Incidence** | **Fiji** | **Female** | **55+ years** | **Low back pain** | **Number** | **2021** | **5706.1409** | **6867.06913** | **4625.43291** |
| **DALYs (Disability-Adjusted Life Years)** | **Finland** | **Female** | **55+ years** | **Low back pain** | **Number** | **2021** | **26903.3381** | **36394.4838** | **18527.408** |
| **Prevalence** | **Finland** | **Female** | **55+ years** | **Low back pain** | **Number** | **2021** | **250156.952** | **291803.33** | **209875.342** |
| **Incidence** | **Finland** | **Female** | **55+ years** | **Low back pain** | **Number** | **2021** | **104930.92** | **125569.749** | **87970.7986** |
| **DALYs (Disability-Adjusted Life Years)** | **France** | **Female** | **55+ years** | **Low back pain** | **Number** | **2021** | **345954.654** | **460240.953** | **241813.18** |
| **Prevalence** | **France** | **Female** | **55+ years** | **Low back pain** | **Number** | **2021** | **3220934.45** | **3712012.12** | **2746751.53** |
| **Incidence** | **France** | **Female** | **55+ years** | **Low back pain** | **Number** | **2021** | **1279955.12** | **1493598** | **1079776.23** |
| **DALYs (Disability-Adjusted Life Years)** | **Gabon** | **Female** | **55+ years** | **Low back pain** | **Number** | **2021** | **2003.09919** | **2752.03971** | **1370.89759** |
| **Prevalence** | **Gabon** | **Female** | **55+ years** | **Low back pain** | **Number** | **2021** | **18515.0388** | **22021.4062** | **15461.7753** |
| **Incidence** | **Gabon** | **Female** | **55+ years** | **Low back pain** | **Number** | **2021** | **7604.64628** | **9156.86789** | **6249.6636** |
| **DALYs (Disability-Adjusted Life Years)** | **Gambia** | **Female** | **55+ years** | **Low back pain** | **Number** | **2021** | **1616.00581** | **2232.49812** | **1110.6488** |
| **Prevalence** | **Gambia** | **Female** | **55+ years** | **Low back pain** | **Number** | **2021** | **15032.1199** | **17996.8249** | **12405.0128** |
| **Incidence** | **Gambia** | **Female** | **55+ years** | **Low back pain** | **Number** | **2021** | **6205.82826** | **7459.94021** | **5046.78194** |
| **DALYs (Disability-Adjusted Life Years)** | **Georgia** | **Female** | **55+ years** | **Low back pain** | **Number** | **2021** | **16701.2718** | **22381.7476** | **11595.5505** |
| **Prevalence** | **Georgia** | **Female** | **55+ years** | **Low back pain** | **Number** | **2021** | **155256.327** | **180946.372** | **130714.508** |
| **Incidence** | **Georgia** | **Female** | **55+ years** | **Low back pain** | **Number** | **2021** | **61346.5719** | **72360.4139** | **51592.6637** |
| **DALYs (Disability-Adjusted Life Years)** | **Germany** | **Female** | **55+ years** | **Low back pain** | **Number** | **2021** | **530959.361** | **712249.566** | **371853.207** |
| **Prevalence** | **Germany** | **Female** | **55+ years** | **Low back pain** | **Number** | **2021** | **4946645.68** | **5722666.76** | **4242553.24** |
| **Incidence** | **Germany** | **Female** | **55+ years** | **Low back pain** | **Number** | **2021** | **1878091.29** | **2179257.77** | **1585170.76** |
| **DALYs (Disability-Adjusted Life Years)** | **Ghana** | **Female** | **55+ years** | **Low back pain** | **Number** | **2021** | **29147.6624** | **38804.175** | **20492.235** |
| **Prevalence** | **Ghana** | **Female** | **55+ years** | **Low back pain** | **Number** | **2021** | **265697.176** | **313128.939** | **223586.761** |
| **Incidence** | **Ghana** | **Female** | **55+ years** | **Low back pain** | **Number** | **2021** | **112910.277** | **133216.439** | **94162.4297** |
| **DALYs (Disability-Adjusted Life Years)** | **Greece** | **Female** | **55+ years** | **Low back pain** | **Number** | **2021** | **53165.3508** | **71099.2415** | **37281.8367** |
| **Prevalence** | **Greece** | **Female** | **55+ years** | **Low back pain** | **Number** | **2021** | **492834.993** | **569674.367** | **416857.127** |
| **Incidence** | **Greece** | **Female** | **55+ years** | **Low back pain** | **Number** | **2021** | **201099.551** | **233270.097** | **168853.53** |
| **DALYs (Disability-Adjusted Life Years)** | **Greenland** | **Female** | **55+ years** | **Low back pain** | **Number** | **2021** | **134.978393** | **187.828504** | **90.0774992** |
| **Prevalence** | **Greenland** | **Female** | **55+ years** | **Low back pain** | **Number** | **2021** | **1225.38197** | **1471.61532** | **988.183128** |
| **Incidence** | **Greenland** | **Female** | **55+ years** | **Low back pain** | **Number** | **2021** | **511.80817** | **628.46166** | **410.405459** |
| **DALYs (Disability-Adjusted Life Years)** | **Grenada** | **Female** | **55+ years** | **Low back pain** | **Number** | **2021** | **190.140721** | **259.356324** | **129.517019** |
| **Prevalence** | **Grenada** | **Female** | **55+ years** | **Low back pain** | **Number** | **2021** | **1750.71767** | **2081.63259** | **1442.04286** |
| **Incidence** | **Grenada** | **Female** | **55+ years** | **Low back pain** | **Number** | **2021** | **713.748901** | **852.838994** | **588.299299** |
| **DALYs (Disability-Adjusted Life Years)** | **Guam** | **Female** | **55+ years** | **Low back pain** | **Number** | **2021** | **429.417192** | **581.938652** | **294.733381** |
| **Prevalence** | **Guam** | **Female** | **55+ years** | **Low back pain** | **Number** | **2021** | **3940.78715** | **4640.91972** | **3291.34771** |
| **Incidence** | **Guam** | **Female** | **55+ years** | **Low back pain** | **Number** | **2021** | **1592.65897** | **1895.25253** | **1315.59359** |
| **DALYs (Disability-Adjusted Life Years)** | **Guatemala** | **Female** | **55+ years** | **Low back pain** | **Number** | **2021** | **22584.2676** | **30915.1214** | **15513.562** |
| **Prevalence** | **Guatemala** | **Female** | **55+ years** | **Low back pain** | **Number** | **2021** | **209426.54** | **248173.203** | **175399.746** |
| **Incidence** | **Guatemala** | **Female** | **55+ years** | **Low back pain** | **Number** | **2021** | **80565.1887** | **95720.759** | **65687.4874** |
| **DALYs (Disability-Adjusted Life Years)** | **Guinea** | **Female** | **55+ years** | **Low back pain** | **Number** | **2021** | **9946.50612** | **13823.7896** | **6790.25196** |
| **Prevalence** | **Guinea** | **Female** | **55+ years** | **Low back pain** | **Number** | **2021** | **91361.3617** | **109958.866** | **74847.0302** |
| **Incidence** | **Guinea** | **Female** | **55+ years** | **Low back pain** | **Number** | **2021** | **36926.9454** | **43934.7596** | **30418.0333** |
| **DALYs (Disability-Adjusted Life Years)** | **Guinea-Bissau** | **Female** | **55+ years** | **Low back pain** | **Number** | **2021** | **1314.13827** | **1858.14102** | **891.880695** |
| **Prevalence** | **Guinea-Bissau** | **Female** | **55+ years** | **Low back pain** | **Number** | **2021** | **12043.4535** | **14643.9544** | **9823.72581** |
| **Incidence** | **Guinea-Bissau** | **Female** | **55+ years** | **Low back pain** | **Number** | **2021** | **4967.26182** | **5967.74845** | **4004.62548** |
| **DALYs (Disability-Adjusted Life Years)** | **Guyana** | **Female** | **55+ years** | **Low back pain** | **Number** | **2021** | **1067.24039** | **1489.8213** | **739.227697** |
| **Prevalence** | **Guyana** | **Female** | **55+ years** | **Low back pain** | **Number** | **2021** | **9933.04255** | **11963.9115** | **8039.17695** |
| **Incidence** | **Guyana** | **Female** | **55+ years** | **Low back pain** | **Number** | **2021** | **4082.37485** | **4923.73613** | **3347.06105** |
| **DALYs (Disability-Adjusted Life Years)** | **Haiti** | **Female** | **55+ years** | **Low back pain** | **Number** | **2021** | **11092.7692** | **15303.5387** | **7556.95122** |
| **Prevalence** | **Haiti** | **Female** | **55+ years** | **Low back pain** | **Number** | **2021** | **102520.398** | **124059.427** | **83292.7072** |
| **Incidence** | **Haiti** | **Female** | **55+ years** | **Low back pain** | **Number** | **2021** | **42133.3528** | **51037.8736** | **33766.7629** |
| **DALYs (Disability-Adjusted Life Years)** | **Honduras** | **Female** | **55+ years** | **Low back pain** | **Number** | **2021** | **11867.223** | **16065.5262** | **8228.2216** |
| **Prevalence** | **Honduras** | **Female** | **55+ years** | **Low back pain** | **Number** | **2021** | **109257.453** | **129613.501** | **89483.7922** |
| **Incidence** | **Honduras** | **Female** | **55+ years** | **Low back pain** | **Number** | **2021** | **43514.6067** | **52837.9705** | **35529.7749** |
| **DALYs (Disability-Adjusted Life Years)** | **Hungary** | **Female** | **55+ years** | **Low back pain** | **Number** | **2021** | **71442.157** | **97132.0909** | **49625.1357** |
| **Prevalence** | **Hungary** | **Female** | **55+ years** | **Low back pain** | **Number** | **2021** | **668031.278** | **765774.948** | **572307.918** |
| **Incidence** | **Hungary** | **Female** | **55+ years** | **Low back pain** | **Number** | **2021** | **225741.375** | **263458.156** | **188270.012** |
| **DALYs (Disability-Adjusted Life Years)** | **Iceland** | **Female** | **55+ years** | **Low back pain** | **Number** | **2021** | **1400.11988** | **1894.98315** | **969.715261** |
| **Prevalence** | **Iceland** | **Female** | **55+ years** | **Low back pain** | **Number** | **2021** | **12819.6871** | **14925.8764** | **10798.7078** |
| **Incidence** | **Iceland** | **Female** | **55+ years** | **Low back pain** | **Number** | **2021** | **5184.88723** | **6113.12152** | **4319.51567** |
| **DALYs (Disability-Adjusted Life Years)** | **India** | **Female** | **55+ years** | **Low back pain** | **Number** | **2021** | **2438030.12** | **3330974.92** | **1655024.95** |
| **Prevalence** | **India** | **Female** | **55+ years** | **Low back pain** | **Number** | **2021** | **22921933.9** | **27286428.4** | **18939725.6** |
| **Incidence** | **India** | **Female** | **55+ years** | **Low back pain** | **Number** | **2021** | **9147098.01** | **10861467.1** | **7516931.84** |
| **DALYs (Disability-Adjusted Life Years)** | **Indonesia** | **Female** | **55+ years** | **Low back pain** | **Number** | **2021** | **477479.949** | **660542.328** | **322167.464** |
| **Prevalence** | **Indonesia** | **Female** | **55+ years** | **Low back pain** | **Number** | **2021** | **4353263.17** | **5192391.92** | **3580414.94** |
| **Incidence** | **Indonesia** | **Female** | **55+ years** | **Low back pain** | **Number** | **2021** | **1736514.67** | **2066922.5** | **1411876.36** |
| **DALYs (Disability-Adjusted Life Years)** | **Iran (Islamic Republic of)** | **Female** | **55+ years** | **Low back pain** | **Number** | **2021** | **164929.978** | **225458.081** | **113234.826** |
| **Prevalence** | **Iran (Islamic Republic of)** | **Female** | **55+ years** | **Low back pain** | **Number** | **2021** | **1537664.2** | **1819196.91** | **1279485.16** |
| **Incidence** | **Iran (Islamic Republic of)** | **Female** | **55+ years** | **Low back pain** | **Number** | **2021** | **605578.581** | **711446.535** | **498874.075** |
| **DALYs (Disability-Adjusted Life Years)** | **Iraq** | **Female** | **55+ years** | **Low back pain** | **Number** | **2021** | **43018.1535** | **58964.9513** | **29403.0069** |
| **Prevalence** | **Iraq** | **Female** | **55+ years** | **Low back pain** | **Number** | **2021** | **402756.737** | **478460.443** | **333696.089** |
| **Incidence** | **Iraq** | **Female** | **55+ years** | **Low back pain** | **Number** | **2021** | **163652.636** | **194494.911** | **132968.325** |
| **DALYs (Disability-Adjusted Life Years)** | **Ireland** | **Female** | **55+ years** | **Low back pain** | **Number** | **2021** | **18947.8231** | **25650.3501** | **13298.0617** |
| **Prevalence** | **Ireland** | **Female** | **55+ years** | **Low back pain** | **Number** | **2021** | **174109.713** | **200178.152** | **147481.463** |
| **Incidence** | **Ireland** | **Female** | **55+ years** | **Low back pain** | **Number** | **2021** | **70698.711** | **83524.2728** | **58799.9729** |
| **DALYs (Disability-Adjusted Life Years)** | **Israel** | **Female** | **55+ years** | **Low back pain** | **Number** | **2021** | **30405.4135** | **40888.829** | **21069.2275** |
| **Prevalence** | **Israel** | **Female** | **55+ years** | **Low back pain** | **Number** | **2021** | **279876.969** | **327576.117** | **235794.376** |
| **Incidence** | **Israel** | **Female** | **55+ years** | **Low back pain** | **Number** | **2021** | **112572.674** | **131680.803** | **93302.1906** |
| **DALYs (Disability-Adjusted Life Years)** | **Italy** | **Female** | **55+ years** | **Low back pain** | **Number** | **2021** | **346283.847** | **459379.189** | **242861.793** |
| **Prevalence** | **Italy** | **Female** | **55+ years** | **Low back pain** | **Number** | **2021** | **3221200.85** | **3702352.14** | **2722254.73** |
| **Incidence** | **Italy** | **Female** | **55+ years** | **Low back pain** | **Number** | **2021** | **1297329.77** | **1502273.48** | **1084923.37** |
| **DALYs (Disability-Adjusted Life Years)** | **Jamaica** | **Female** | **55+ years** | **Low back pain** | **Number** | **2021** | **5182.93079** | **7088.38859** | **3529.07295** |
| **Prevalence** | **Jamaica** | **Female** | **55+ years** | **Low back pain** | **Number** | **2021** | **47584.0721** | **56861.4315** | **39406.9714** |
| **Incidence** | **Jamaica** | **Female** | **55+ years** | **Low back pain** | **Number** | **2021** | **19152.7702** | **22643.5438** | **15778.2881** |
| **DALYs (Disability-Adjusted Life Years)** | **Japan** | **Female** | **55+ years** | **Low back pain** | **Number** | **2021** | **834789.765** | **1111884.8** | **584249.273** |
| **Prevalence** | **Japan** | **Female** | **55+ years** | **Low back pain** | **Number** | **2021** | **7704924.9** | **8807761.07** | **6583030.72** |
| **Incidence** | **Japan** | **Female** | **55+ years** | **Low back pain** | **Number** | **2021** | **2984127.48** | **3471738.78** | **2512396.5** |
| **DALYs (Disability-Adjusted Life Years)** | **Jordan** | **Female** | **55+ years** | **Low back pain** | **Number** | **2021** | **13082.1276** | **17906.1625** | **8906.09942** |
| **Prevalence** | **Jordan** | **Female** | **55+ years** | **Low back pain** | **Number** | **2021** | **122100.369** | **146447.194** | **98583.5349** |
| **Incidence** | **Jordan** | **Female** | **55+ years** | **Low back pain** | **Number** | **2021** | **49555.9364** | **60070.6233** | **39692.252** |
| **DALYs (Disability-Adjusted Life Years)** | **Kazakhstan** | **Female** | **55+ years** | **Low back pain** | **Number** | **2021** | **54158.244** | **75794.5715** | **37104.7565** |
| **Prevalence** | **Kazakhstan** | **Female** | **55+ years** | **Low back pain** | **Number** | **2021** | **499721.613** | **589461.586** | **414853.793** |
| **Incidence** | **Kazakhstan** | **Female** | **55+ years** | **Low back pain** | **Number** | **2021** | **191206.292** | **225897.267** | **156976.372** |
| **DALYs (Disability-Adjusted Life Years)** | **Kenya** | **Female** | **55+ years** | **Low back pain** | **Number** | **2021** | **55921.8942** | **76631.1112** | **38444.4904** |
| **Prevalence** | **Kenya** | **Female** | **55+ years** | **Low back pain** | **Number** | **2021** | **515822.654** | **611149.478** | **427143.992** |
| **Incidence** | **Kenya** | **Female** | **55+ years** | **Low back pain** | **Number** | **2021** | **203542.453** | **241781.884** | **168234.36** |
| **DALYs (Disability-Adjusted Life Years)** | **Kiribati** | **Female** | **55+ years** | **Low back pain** | **Number** | **2021** | **155.298272** | **214.82587** | **103.893806** |
| **Prevalence** | **Kiribati** | **Female** | **55+ years** | **Low back pain** | **Number** | **2021** | **1434.20951** | **1730.91179** | **1157.23381** |
| **Incidence** | **Kiribati** | **Female** | **55+ years** | **Low back pain** | **Number** | **2021** | **577.130505** | **687.123237** | **465.03456** |
| **DALYs (Disability-Adjusted Life Years)** | **Kuwait** | **Female** | **55+ years** | **Low back pain** | **Number** | **2021** | **4136.41765** | **5741.76814** | **2761.82442** |
| **Prevalence** | **Kuwait** | **Female** | **55+ years** | **Low back pain** | **Number** | **2021** | **38383.944** | **46393.7225** | **31184.3318** |
| **Incidence** | **Kuwait** | **Female** | **55+ years** | **Low back pain** | **Number** | **2021** | **15515.9275** | **18813.4698** | **12582.6102** |
| **DALYs (Disability-Adjusted Life Years)** | **Kyrgyzstan** | **Female** | **55+ years** | **Low back pain** | **Number** | **2021** | **13348.4571** | **18462.5817** | **9093.12568** |
| **Prevalence** | **Kyrgyzstan** | **Female** | **55+ years** | **Low back pain** | **Number** | **2021** | **121594.935** | **143610.915** | **100146.675** |
| **Incidence** | **Kyrgyzstan** | **Female** | **55+ years** | **Low back pain** | **Number** | **2021** | **47405.2793** | **56310.3024** | **38620.219** |
| **DALYs (Disability-Adjusted Life Years)** | **Lao People's Democratic Republic** | **Female** | **55+ years** | **Low back pain** | **Number** | **2021** | **7699.73987** | **10530.042** | **5289.05547** |
| **Prevalence** | **Lao People's Democratic Republic** | **Female** | **55+ years** | **Low back pain** | **Number** | **2021** | **70179.2587** | **83598.1976** | **57553.3167** |
| **Incidence** | **Lao People's Democratic Republic** | **Female** | **55+ years** | **Low back pain** | **Number** | **2021** | **28662.9469** | **34308.1804** | **23107.8938** |
| **DALYs (Disability-Adjusted Life Years)** | **Latvia** | **Female** | **55+ years** | **Low back pain** | **Number** | **2021** | **14154.798** | **19173.2023** | **9971.91801** |
| **Prevalence** | **Latvia** | **Female** | **55+ years** | **Low back pain** | **Number** | **2021** | **132543.598** | **152080.485** | **113562.254** |
| **Incidence** | **Latvia** | **Female** | **55+ years** | **Low back pain** | **Number** | **2021** | **47138.182** | **55677.8595** | **39350.1488** |
| **DALYs (Disability-Adjusted Life Years)** | **Lebanon** | **Female** | **55+ years** | **Low back pain** | **Number** | **2021** | **11978.0677** | **16131.7618** | **8362.05853** |
| **Prevalence** | **Lebanon** | **Female** | **55+ years** | **Low back pain** | **Number** | **2021** | **113166.908** | **134025.241** | **95694.0419** |
| **Incidence** | **Lebanon** | **Female** | **55+ years** | **Low back pain** | **Number** | **2021** | **45321.2529** | **53341.2648** | **37983.3261** |
| **DALYs (Disability-Adjusted Life Years)** | **Lesotho** | **Female** | **55+ years** | **Low back pain** | **Number** | **2021** | **2406.03601** | **3314.73203** | **1650.75199** |
| **Prevalence** | **Lesotho** | **Female** | **55+ years** | **Low back pain** | **Number** | **2021** | **22472.3225** | **26791.3199** | **18554.9631** |
| **Incidence** | **Lesotho** | **Female** | **55+ years** | **Low back pain** | **Number** | **2021** | **9225.0534** | **11022.349** | **7459.2203** |
| **DALYs (Disability-Adjusted Life Years)** | **Liberia** | **Female** | **55+ years** | **Low back pain** | **Number** | **2021** | **3340.17514** | **4610.94053** | **2284.70711** |
| **Prevalence** | **Liberia** | **Female** | **55+ years** | **Low back pain** | **Number** | **2021** | **31310.3843** | **37741.2998** | **25846.7577** |
| **Incidence** | **Liberia** | **Female** | **55+ years** | **Low back pain** | **Number** | **2021** | **12808.8793** | **15371.0694** | **10409.1611** |
| **DALYs (Disability-Adjusted Life Years)** | **Libya** | **Female** | **55+ years** | **Low back pain** | **Number** | **2021** | **8985.02389** | **12388.6989** | **6170.47529** |
| **Prevalence** | **Libya** | **Female** | **55+ years** | **Low back pain** | **Number** | **2021** | **83671.4646** | **99634.2763** | **69157.1814** |
| **Incidence** | **Libya** | **Female** | **55+ years** | **Low back pain** | **Number** | **2021** | **34038.9256** | **41061.2532** | **27530.0343** |
| **DALYs (Disability-Adjusted Life Years)** | **Lithuania** | **Female** | **55+ years** | **Low back pain** | **Number** | **2021** | **20304.9618** | **27369.0128** | **14101.1016** |
| **Prevalence** | **Lithuania** | **Female** | **55+ years** | **Low back pain** | **Number** | **2021** | **190168.443** | **221613.424** | **161726.379** |
| **Incidence** | **Lithuania** | **Female** | **55+ years** | **Low back pain** | **Number** | **2021** | **68181.1313** | **79023.5657** | **57316.4256** |
| **DALYs (Disability-Adjusted Life Years)** | **Luxembourg** | **Female** | **55+ years** | **Low back pain** | **Number** | **2021** | **2664.38676** | **3562.49905** | **1870.51189** |
| **Prevalence** | **Luxembourg** | **Female** | **55+ years** | **Low back pain** | **Number** | **2021** | **24526.4501** | **28304.1014** | **20958.5766** |
| **Incidence** | **Luxembourg** | **Female** | **55+ years** | **Low back pain** | **Number** | **2021** | **9725.32166** | **11360.2548** | **8176.43725** |
| **DALYs (Disability-Adjusted Life Years)** | **Madagascar** | **Female** | **55+ years** | **Low back pain** | **Number** | **2021** | **23366.9464** | **32708.2049** | **15799.5401** |
| **Prevalence** | **Madagascar** | **Female** | **55+ years** | **Low back pain** | **Number** | **2021** | **212639.046** | **256732.449** | **173720.505** |
| **Incidence** | **Madagascar** | **Female** | **55+ years** | **Low back pain** | **Number** | **2021** | **84906.2119** | **102713.624** | **68047.2035** |
| **DALYs (Disability-Adjusted Life Years)** | **Malawi** | **Female** | **55+ years** | **Low back pain** | **Number** | **2021** | **16196.3405** | **22320.811** | **11049.2106** |
| **Prevalence** | **Malawi** | **Female** | **55+ years** | **Low back pain** | **Number** | **2021** | **148924.61** | **178037.992** | **122565.499** |
| **Incidence** | **Malawi** | **Female** | **55+ years** | **Low back pain** | **Number** | **2021** | **59528.5126** | **71742.7586** | **48839.7941** |
| **DALYs (Disability-Adjusted Life Years)** | **Malaysia** | **Female** | **55+ years** | **Low back pain** | **Number** | **2021** | **47132.8569** | **64339.7035** | **32153.0363** |
| **Prevalence** | **Malaysia** | **Female** | **55+ years** | **Low back pain** | **Number** | **2021** | **434709.545** | **513792.786** | **358359.792** |
| **Incidence** | **Malaysia** | **Female** | **55+ years** | **Low back pain** | **Number** | **2021** | **178342.324** | **212263.831** | **147368.269** |
| **DALYs (Disability-Adjusted Life Years)** | **Maldives** | **Female** | **55+ years** | **Low back pain** | **Number** | **2021** | **451.823443** | **612.381332** | **307.698902** |
| **Prevalence** | **Maldives** | **Female** | **55+ years** | **Low back pain** | **Number** | **2021** | **4133.93637** | **4973.55793** | **3400.41067** |
| **Incidence** | **Maldives** | **Female** | **55+ years** | **Low back pain** | **Number** | **2021** | **1712.74298** | **2059.046** | **1390.16292** |
| **DALYs (Disability-Adjusted Life Years)** | **Mali** | **Female** | **55+ years** | **Low back pain** | **Number** | **2021** | **14085.1267** | **19223.8314** | **9570.02917** |
| **Prevalence** | **Mali** | **Female** | **55+ years** | **Low back pain** | **Number** | **2021** | **129945.135** | **156508.698** | **104499.53** |
| **Incidence** | **Mali** | **Female** | **55+ years** | **Low back pain** | **Number** | **2021** | **54212.0447** | **65505.7613** | **43550.5186** |
| **DALYs (Disability-Adjusted Life Years)** | **Malta** | **Female** | **55+ years** | **Low back pain** | **Number** | **2021** | **2543.98113** | **3381.96676** | **1795.99612** |
| **Prevalence** | **Malta** | **Female** | **55+ years** | **Low back pain** | **Number** | **2021** | **23410.0197** | **26618.3472** | **19894.3845** |
| **Incidence** | **Malta** | **Female** | **55+ years** | **Low back pain** | **Number** | **2021** | **9136.39998** | **10568.5896** | **7743.61445** |
| **DALYs (Disability-Adjusted Life Years)** | **Marshall Islands** | **Female** | **55+ years** | **Low back pain** | **Number** | **2021** | **58.8483675** | **83.4314745** | **40.1374611** |
| **Prevalence** | **Marshall Islands** | **Female** | **55+ years** | **Low back pain** | **Number** | **2021** | **543.200593** | **655.491201** | **439.593152** |
| **Incidence** | **Marshall Islands** | **Female** | **55+ years** | **Low back pain** | **Number** | **2021** | **223.950163** | **272.451303** | **178.03406** |
| **DALYs (Disability-Adjusted Life Years)** | **Mauritania** | **Female** | **55+ years** | **Low back pain** | **Number** | **2021** | **3771.30973** | **5223.90082** | **2562.19569** |
| **Prevalence** | **Mauritania** | **Female** | **55+ years** | **Low back pain** | **Number** | **2021** | **34410.1598** | **41720.7441** | **28085.4287** |
| **Incidence** | **Mauritania** | **Female** | **55+ years** | **Low back pain** | **Number** | **2021** | **14176.5195** | **16948.9016** | **11462.4888** |
| **DALYs (Disability-Adjusted Life Years)** | **Mauritius** | **Female** | **55+ years** | **Low back pain** | **Number** | **2021** | **3554.52931** | **4747.19211** | **2397.78681** |
| **Prevalence** | **Mauritius** | **Female** | **55+ years** | **Low back pain** | **Number** | **2021** | **33137.4368** | **39289.2599** | **27583.5154** |
| **Incidence** | **Mauritius** | **Female** | **55+ years** | **Low back pain** | **Number** | **2021** | **13486.1193** | **16134.3509** | **11045.3112** |
| **DALYs (Disability-Adjusted Life Years)** | **Mexico** | **Female** | **55+ years** | **Low back pain** | **Number** | **2021** | **230370.746** | **316679.744** | **155924.965** |
| **Prevalence** | **Mexico** | **Female** | **55+ years** | **Low back pain** | **Number** | **2021** | **2132007.76** | **2545601.97** | **1746237.48** |
| **Incidence** | **Mexico** | **Female** | **55+ years** | **Low back pain** | **Number** | **2021** | **861561.355** | **1026730.6** | **704515.623** |
| **DALYs (Disability-Adjusted Life Years)** | **Micronesia (Federated States of)** | **Female** | **55+ years** | **Low back pain** | **Number** | **2021** | **149.085835** | **203.904598** | **100.393342** |
| **Prevalence** | **Micronesia (Federated States of)** | **Female** | **55+ years** | **Low back pain** | **Number** | **2021** | **1372.2589** | **1673.37224** | **1118.22758** |
| **Incidence** | **Micronesia (Federated States of)** | **Female** | **55+ years** | **Low back pain** | **Number** | **2021** | **553.719145** | **659.396535** | **444.399689** |
| **DALYs (Disability-Adjusted Life Years)** | **Monaco** | **Female** | **55+ years** | **Low back pain** | **Number** | **2021** | **226.151735** | **305.006386** | **155.767327** |
| **Prevalence** | **Monaco** | **Female** | **55+ years** | **Low back pain** | **Number** | **2021** | **2080.99268** | **2397.85888** | **1761.20016** |
| **Incidence** | **Monaco** | **Female** | **55+ years** | **Low back pain** | **Number** | **2021** | **845.947299** | **989.814195** | **710.801729** |
| **DALYs (Disability-Adjusted Life Years)** | **Mongolia** | **Female** | **55+ years** | **Low back pain** | **Number** | **2021** | **6153.96031** | **8422.79147** | **4197.39093** |
| **Prevalence** | **Mongolia** | **Female** | **55+ years** | **Low back pain** | **Number** | **2021** | **55949.7684** | **67129.7647** | **46089.7159** |
| **Incidence** | **Mongolia** | **Female** | **55+ years** | **Low back pain** | **Number** | **2021** | **22002.0152** | **26325.9986** | **17972.1888** |
| **DALYs (Disability-Adjusted Life Years)** | **Montenegro** | **Female** | **55+ years** | **Low back pain** | **Number** | **2021** | **3339.49607** | **4586.85741** | **2288.43965** |
| **Prevalence** | **Montenegro** | **Female** | **55+ years** | **Low back pain** | **Number** | **2021** | **30856.7594** | **35980.1117** | **25529.3999** |
| **Incidence** | **Montenegro** | **Female** | **55+ years** | **Low back pain** | **Number** | **2021** | **11063.2528** | **12967.1353** | **9237.01265** |
| **DALYs (Disability-Adjusted Life Years)** | **Morocco** | **Female** | **55+ years** | **Low back pain** | **Number** | **2021** | **74378.1859** | **102417.056** | **51688.5128** |
| **Prevalence** | **Morocco** | **Female** | **55+ years** | **Low back pain** | **Number** | **2021** | **698267.067** | **826101.858** | **573479.279** |
| **Incidence** | **Morocco** | **Female** | **55+ years** | **Low back pain** | **Number** | **2021** | **269878.649** | **320973.351** | **220683.579** |
| **DALYs (Disability-Adjusted Life Years)** | **Mozambique** | **Female** | **55+ years** | **Low back pain** | **Number** | **2021** | **25564.7503** | **34945.739** | **17634.7289** |
| **Prevalence** | **Mozambique** | **Female** | **55+ years** | **Low back pain** | **Number** | **2021** | **237828.396** | **284469.873** | **194325.26** |
| **Incidence** | **Mozambique** | **Female** | **55+ years** | **Low back pain** | **Number** | **2021** | **92635.0146** | **111295.766** | **75360.2699** |
| **DALYs (Disability-Adjusted Life Years)** | **Myanmar** | **Female** | **55+ years** | **Low back pain** | **Number** | **2021** | **81136.4312** | **109479.619** | **55543.8125** |
| **Prevalence** | **Myanmar** | **Female** | **55+ years** | **Low back pain** | **Number** | **2021** | **746948.896** | **887872.545** | **615276.254** |
| **Incidence** | **Myanmar** | **Female** | **55+ years** | **Low back pain** | **Number** | **2021** | **315587.596** | **376839.524** | **259140.674** |
| **DALYs (Disability-Adjusted Life Years)** | **Namibia** | **Female** | **55+ years** | **Low back pain** | **Number** | **2021** | **3042.11775** | **4145.91796** | **2089.16336** |
| **Prevalence** | **Namibia** | **Female** | **55+ years** | **Low back pain** | **Number** | **2021** | **27892.8968** | **33294.8213** | **22667.6508** |
| **Incidence** | **Namibia** | **Female** | **55+ years** | **Low back pain** | **Number** | **2021** | **11158.1486** | **13435.1745** | **9090.98432** |
| **DALYs (Disability-Adjusted Life Years)** | **Nauru** | **Female** | **55+ years** | **Low back pain** | **Number** | **2021** | **11.467951** | **15.9038187** | **7.80320425** |
| **Prevalence** | **Nauru** | **Female** | **55+ years** | **Low back pain** | **Number** | **2021** | **106.242501** | **127.62625** | **85.9354733** |
| **Incidence** | **Nauru** | **Female** | **55+ years** | **Low back pain** | **Number** | **2021** | **42.8248252** | **52.2681075** | **34.6109714** |
| **DALYs (Disability-Adjusted Life Years)** | **Nepal** | **Female** | **55+ years** | **Low back pain** | **Number** | **2021** | **67531.2682** | **93715.4041** | **47206.8498** |
| **Prevalence** | **Nepal** | **Female** | **55+ years** | **Low back pain** | **Number** | **2021** | **630579.778** | **747305.33** | **530429.141** |
| **Incidence** | **Nepal** | **Female** | **55+ years** | **Low back pain** | **Number** | **2021** | **219739.638** | **263305.324** | **180179.897** |
| **DALYs (Disability-Adjusted Life Years)** | **Netherlands** | **Female** | **55+ years** | **Low back pain** | **Number** | **2021** | **75470.0691** | **101452.794** | **51307.4567** |
| **Prevalence** | **Netherlands** | **Female** | **55+ years** | **Low back pain** | **Number** | **2021** | **696071.584** | **807337.41** | **590483.299** |
| **Incidence** | **Netherlands** | **Female** | **55+ years** | **Low back pain** | **Number** | **2021** | **292915.94** | **346086.533** | **247360.504** |
| **DALYs (Disability-Adjusted Life Years)** | **New Zealand** | **Female** | **55+ years** | **Low back pain** | **Number** | **2021** | **26262.3221** | **35622.5857** | **18623.7534** |
| **Prevalence** | **New Zealand** | **Female** | **55+ years** | **Low back pain** | **Number** | **2021** | **241947.54** | **277923.848** | **205894.542** |
| **Incidence** | **New Zealand** | **Female** | **55+ years** | **Low back pain** | **Number** | **2021** | **94276.398** | **109928.092** | **78666.2277** |
| **DALYs (Disability-Adjusted Life Years)** | **Nicaragua** | **Female** | **55+ years** | **Low back pain** | **Number** | **2021** | **9523.21454** | **13076.7518** | **6588.68766** |
| **Prevalence** | **Nicaragua** | **Female** | **55+ years** | **Low back pain** | **Number** | **2021** | **87736.625** | **104046.037** | **72755.7323** |
| **Incidence** | **Nicaragua** | **Female** | **55+ years** | **Low back pain** | **Number** | **2021** | **34771.257** | **41625.7571** | **28787.1414** |
| **DALYs (Disability-Adjusted Life Years)** | **Niger** | **Female** | **55+ years** | **Low back pain** | **Number** | **2021** | **15813.1463** | **21866.0755** | **10838.4609** |
| **Prevalence** | **Niger** | **Female** | **55+ years** | **Low back pain** | **Number** | **2021** | **144540.179** | **173373.547** | **117797.755** |
| **Incidence** | **Niger** | **Female** | **55+ years** | **Low back pain** | **Number** | **2021** | **58683.2397** | **70683.1824** | **47208.9712** |
| **DALYs (Disability-Adjusted Life Years)** | **Nigeria** | **Female** | **55+ years** | **Low back pain** | **Number** | **2021** | **186046.131** | **257105.456** | **127023.408** |
| **Prevalence** | **Nigeria** | **Female** | **55+ years** | **Low back pain** | **Number** | **2021** | **1707755.98** | **2045593.92** | **1399605.72** |
| **Incidence** | **Nigeria** | **Female** | **55+ years** | **Low back pain** | **Number** | **2021** | **698004.206** | **839945.993** | **566452.837** |
| **DALYs (Disability-Adjusted Life Years)** | **Niue** | **Female** | **55+ years** | **Low back pain** | **Number** | **2021** | **4.51449676** | **6.07376535** | **3.14891499** |
| **Prevalence** | **Niue** | **Female** | **55+ years** | **Low back pain** | **Number** | **2021** | **42.1731706** | **49.8081194** | **34.9456649** |
| **Incidence** | **Niue** | **Female** | **55+ years** | **Low back pain** | **Number** | **2021** | **16.953035** | **20.2455768** | **13.9172029** |
| **DALYs (Disability-Adjusted Life Years)** | **North Macedonia** | **Female** | **55+ years** | **Low back pain** | **Number** | **2021** | **9906.7607** | **13511.1963** | **6922.30777** |
| **Prevalence** | **North Macedonia** | **Female** | **55+ years** | **Low back pain** | **Number** | **2021** | **91910.8903** | **107094.962** | **77825.8607** |
| **Incidence** | **North Macedonia** | **Female** | **55+ years** | **Low back pain** | **Number** | **2021** | **34450.3096** | **40714.4167** | **28318.4345** |
| **DALYs (Disability-Adjusted Life Years)** | **Northern Mariana Islands** | **Female** | **55+ years** | **Low back pain** | **Number** | **2021** | **104.157741** | **147.632568** | **70.4053493** |
| **Prevalence** | **Northern Mariana Islands** | **Female** | **55+ years** | **Low back pain** | **Number** | **2021** | **943.904474** | **1142.14903** | **772.39038** |
| **Incidence** | **Northern Mariana Islands** | **Female** | **55+ years** | **Low back pain** | **Number** | **2021** | **380.920378** | **462.17722** | **307.635561** |
| **DALYs (Disability-Adjusted Life Years)** | **Norway** | **Female** | **55+ years** | **Low back pain** | **Number** | **2021** | **22374.4204** | **29846.1813** | **15595.9653** |
| **Prevalence** | **Norway** | **Female** | **55+ years** | **Low back pain** | **Number** | **2021** | **206699.45** | **238787.815** | **175344.765** |
| **Incidence** | **Norway** | **Female** | **55+ years** | **Low back pain** | **Number** | **2021** | **84655.6606** | **98918.5687** | **71500.3747** |
| **DALYs (Disability-Adjusted Life Years)** | **Oman** | **Female** | **55+ years** | **Low back pain** | **Number** | **2021** | **2941.93427** | **4062.92274** | **2008.50327** |
| **Prevalence** | **Oman** | **Female** | **55+ years** | **Low back pain** | **Number** | **2021** | **27242.7009** | **32556.9221** | **22296.5865** |
| **Incidence** | **Oman** | **Female** | **55+ years** | **Low back pain** | **Number** | **2021** | **11142.0986** | **13406.6364** | **8934.66282** |
| **DALYs (Disability-Adjusted Life Years)** | **Pakistan** | **Female** | **55+ years** | **Low back pain** | **Number** | **2021** | **257737.937** | **357505.062** | **173797.079** |
| **Prevalence** | **Pakistan** | **Female** | **55+ years** | **Low back pain** | **Number** | **2021** | **2398752.75** | **2875677.6** | **1919360.83** |
| **Incidence** | **Pakistan** | **Female** | **55+ years** | **Low back pain** | **Number** | **2021** | **943262.51** | **1146076.11** | **763302.058** |
| **DALYs (Disability-Adjusted Life Years)** | **Palau** | **Female** | **55+ years** | **Low back pain** | **Number** | **2021** | **43.8222639** | **60.7549611** | **29.3738164** |
| **Prevalence** | **Palau** | **Female** | **55+ years** | **Low back pain** | **Number** | **2021** | **404.324699** | **496.273625** | **325.394533** |
| **Incidence** | **Palau** | **Female** | **55+ years** | **Low back pain** | **Number** | **2021** | **164.650018** | **198.644593** | **132.773177** |
| **DALYs (Disability-Adjusted Life Years)** | **Palestine** | **Female** | **55+ years** | **Low back pain** | **Number** | **2021** | **4706.20304** | **6536.60455** | **3246.74859** |
| **Prevalence** | **Palestine** | **Female** | **55+ years** | **Low back pain** | **Number** | **2021** | **43810.7525** | **51704.5513** | **36253.3056** |
| **Incidence** | **Palestine** | **Female** | **55+ years** | **Low back pain** | **Number** | **2021** | **17830.9159** | **21273.6808** | **14479.8058** |
| **DALYs (Disability-Adjusted Life Years)** | **Panama** | **Female** | **55+ years** | **Low back pain** | **Number** | **2021** | **8058.15208** | **10975.9029** | **5588.82918** |
| **Prevalence** | **Panama** | **Female** | **55+ years** | **Low back pain** | **Number** | **2021** | **74242.8** | **88773.4286** | **61728.619** |
| **Incidence** | **Panama** | **Female** | **55+ years** | **Low back pain** | **Number** | **2021** | **29528.9953** | **35342.4544** | **24016.3456** |
| **DALYs (Disability-Adjusted Life Years)** | **Papua New Guinea** | **Female** | **55+ years** | **Low back pain** | **Number** | **2021** | **7941.23743** | **11019.5632** | **5346.75933** |
| **Prevalence** | **Papua New Guinea** | **Female** | **55+ years** | **Low back pain** | **Number** | **2021** | **72611.1877** | **88181.4186** | **58575.9476** |
| **Incidence** | **Papua New Guinea** | **Female** | **55+ years** | **Low back pain** | **Number** | **2021** | **29620.0459** | **36406.9968** | **23855.7224** |
| **DALYs (Disability-Adjusted Life Years)** | **Paraguay** | **Female** | **55+ years** | **Low back pain** | **Number** | **2021** | **11225.0709** | **15386.9661** | **7767.00583** |
| **Prevalence** | **Paraguay** | **Female** | **55+ years** | **Low back pain** | **Number** | **2021** | **103719.168** | **124269.565** | **85908.5856** |
| **Incidence** | **Paraguay** | **Female** | **55+ years** | **Low back pain** | **Number** | **2021** | **40902.4795** | **49286.8091** | **33738.7119** |
| **DALYs (Disability-Adjusted Life Years)** | **Peru** | **Female** | **55+ years** | **Low back pain** | **Number** | **2021** | **50291.45** | **67975.9076** | **34221.7722** |
| **Prevalence** | **Peru** | **Female** | **55+ years** | **Low back pain** | **Number** | **2021** | **460659.777** | **547107.186** | **381690.302** |
| **Incidence** | **Peru** | **Female** | **55+ years** | **Low back pain** | **Number** | **2021** | **190578.55** | **226421.469** | **157055.195** |
| **DALYs (Disability-Adjusted Life Years)** | **Philippines** | **Female** | **55+ years** | **Low back pain** | **Number** | **2021** | **164454.668** | **225128.371** | **112208.457** |
| **Prevalence** | **Philippines** | **Female** | **55+ years** | **Low back pain** | **Number** | **2021** | **1513360.79** | **1801409.82** | **1262450.97** |
| **Incidence** | **Philippines** | **Female** | **55+ years** | **Low back pain** | **Number** | **2021** | **604865.154** | **718065.417** | **496677.109** |
| **DALYs (Disability-Adjusted Life Years)** | **Poland** | **Female** | **55+ years** | **Low back pain** | **Number** | **2021** | **245702.785** | **331549.848** | **172851.078** |
| **Prevalence** | **Poland** | **Female** | **55+ years** | **Low back pain** | **Number** | **2021** | **2289087.53** | **2646412.52** | **1961333.48** |
| **Incidence** | **Poland** | **Female** | **55+ years** | **Low back pain** | **Number** | **2021** | **854930.448** | **1000797.72** | **709169.898** |
| **DALYs (Disability-Adjusted Life Years)** | **Portugal** | **Female** | **55+ years** | **Low back pain** | **Number** | **2021** | **66327.2388** | **89177.8743** | **46599.5943** |
| **Prevalence** | **Portugal** | **Female** | **55+ years** | **Low back pain** | **Number** | **2021** | **615708.862** | **702492.822** | **533714.462** |
| **Incidence** | **Portugal** | **Female** | **55+ years** | **Low back pain** | **Number** | **2021** | **239185.952** | **278315.661** | **201472.191** |
| **DALYs (Disability-Adjusted Life Years)** | **Puerto Rico** | **Female** | **55+ years** | **Low back pain** | **Number** | **2021** | **11927.7203** | **15990.2726** | **8212.63092** |
| **Prevalence** | **Puerto Rico** | **Female** | **55+ years** | **Low back pain** | **Number** | **2021** | **110712.725** | **131807.17** | **92622.3916** |
| **Incidence** | **Puerto Rico** | **Female** | **55+ years** | **Low back pain** | **Number** | **2021** | **44979.7184** | **53350.3317** | **37956.8626** |
| **DALYs (Disability-Adjusted Life Years)** | **Qatar** | **Female** | **55+ years** | **Low back pain** | **Number** | **2021** | **1003.61104** | **1392.63419** | **686.383712** |
| **Prevalence** | **Qatar** | **Female** | **55+ years** | **Low back pain** | **Number** | **2021** | **9390.59952** | **11469.5975** | **7575.11682** |
| **Incidence** | **Qatar** | **Female** | **55+ years** | **Low back pain** | **Number** | **2021** | **3809.21666** | **4638.14113** | **3043.20482** |
| **DALYs (Disability-Adjusted Life Years)** | **Republic of Korea** | **Female** | **55+ years** | **Low back pain** | **Number** | **2021** | **218361.352** | **295651.745** | **149523.977** |
| **Prevalence** | **Republic of Korea** | **Female** | **55+ years** | **Low back pain** | **Number** | **2021** | **1995318.23** | **2347371.63** | **1668794.28** |
| **Incidence** | **Republic of Korea** | **Female** | **55+ years** | **Low back pain** | **Number** | **2021** | **810858.114** | **956222.994** | **666661.898** |
| **DALYs (Disability-Adjusted Life Years)** | **Republic of Moldova** | **Female** | **55+ years** | **Low back pain** | **Number** | **2021** | **21209.679** | **29245.0288** | **14622.6054** |
| **Prevalence** | **Republic of Moldova** | **Female** | **55+ years** | **Low back pain** | **Number** | **2021** | **196509.196** | **228337.932** | **167478.108** |
| **Incidence** | **Republic of Moldova** | **Female** | **55+ years** | **Low back pain** | **Number** | **2021** | **70845.0809** | **84165.8113** | **59016.3245** |
| **DALYs (Disability-Adjusted Life Years)** | **Romania** | **Female** | **55+ years** | **Low back pain** | **Number** | **2021** | **124876.678** | **169325.149** | **87419.8654** |
| **Prevalence** | **Romania** | **Female** | **55+ years** | **Low back pain** | **Number** | **2021** | **1153057.19** | **1328161.97** | **983806.721** |
| **Incidence** | **Romania** | **Female** | **55+ years** | **Low back pain** | **Number** | **2021** | **406121.897** | **473700.592** | **336370.7** |
| **DALYs (Disability-Adjusted Life Years)** | **Russian Federation** | **Female** | **55+ years** | **Low back pain** | **Number** | **2021** | **933666.627** | **1265931.72** | **655246.632** |
| **Prevalence** | **Russian Federation** | **Female** | **55+ years** | **Low back pain** | **Number** | **2021** | **8727318.57** | **10101316.2** | **7481533.7** |
| **Incidence** | **Russian Federation** | **Female** | **55+ years** | **Low back pain** | **Number** | **2021** | **3247756.64** | **3789466.71** | **2700089.4** |
| **DALYs (Disability-Adjusted Life Years)** | **Rwanda** | **Female** | **55+ years** | **Low back pain** | **Number** | **2021** | **16260.4161** | **22275.478** | **11088.8016** |
| **Prevalence** | **Rwanda** | **Female** | **55+ years** | **Low back pain** | **Number** | **2021** | **148277.754** | **177024.339** | **122462.204** |
| **Incidence** | **Rwanda** | **Female** | **55+ years** | **Low back pain** | **Number** | **2021** | **57295.1072** | **68592.2329** | **46586.8178** |
| **DALYs (Disability-Adjusted Life Years)** | **Saint Kitts and Nevis** | **Female** | **55+ years** | **Low back pain** | **Number** | **2021** | **121.264228** | **166.826635** | **80.1451282** |
| **Prevalence** | **Saint Kitts and Nevis** | **Female** | **55+ years** | **Low back pain** | **Number** | **2021** | **1107.01992** | **1328.65031** | **892.348276** |
| **Incidence** | **Saint Kitts and Nevis** | **Female** | **55+ years** | **Low back pain** | **Number** | **2021** | **456.226342** | **548.179332** | **367.713733** |
| **DALYs (Disability-Adjusted Life Years)** | **Saint Lucia** | **Female** | **55+ years** | **Low back pain** | **Number** | **2021** | **406.814836** | **546.380242** | **281.191026** |
| **Prevalence** | **Saint Lucia** | **Female** | **55+ years** | **Low back pain** | **Number** | **2021** | **3752.22771** | **4487.06581** | **3069.99601** |
| **Incidence** | **Saint Lucia** | **Female** | **55+ years** | **Low back pain** | **Number** | **2021** | **1522.73785** | **1821.98505** | **1250.64407** |
| **DALYs (Disability-Adjusted Life Years)** | **Saint Vincent and the Grenadines** | **Female** | **55+ years** | **Low back pain** | **Number** | **2021** | **224.35114** | **303.498228** | **155.323489** |
| **Prevalence** | **Saint Vincent and the Grenadines** | **Female** | **55+ years** | **Low back pain** | **Number** | **2021** | **2065.23569** | **2480.16504** | **1686.94077** |
| **Incidence** | **Saint Vincent and the Grenadines** | **Female** | **55+ years** | **Low back pain** | **Number** | **2021** | **844.906682** | **1006.04474** | **693.573639** |
| **DALYs (Disability-Adjusted Life Years)** | **Samoa** | **Female** | **55+ years** | **Low back pain** | **Number** | **2021** | **274.874789** | **377.205399** | **188.244715** |
| **Prevalence** | **Samoa** | **Female** | **55+ years** | **Low back pain** | **Number** | **2021** | **2546.9096** | **3013.07229** | **2090.26795** |
| **Incidence** | **Samoa** | **Female** | **55+ years** | **Low back pain** | **Number** | **2021** | **1022.3102** | **1221.07892** | **841.741065** |
| **DALYs (Disability-Adjusted Life Years)** | **San Marino** | **Female** | **55+ years** | **Low back pain** | **Number** | **2021** | **167.042377** | **224.149942** | **115.763179** |
| **Prevalence** | **San Marino** | **Female** | **55+ years** | **Low back pain** | **Number** | **2021** | **1541.97319** | **1786.19531** | **1322.00659** |
| **Incidence** | **San Marino** | **Female** | **55+ years** | **Low back pain** | **Number** | **2021** | **626.341819** | **734.073113** | **529.540763** |
| **DALYs (Disability-Adjusted Life Years)** | **Sao Tome and Principe** | **Female** | **55+ years** | **Low back pain** | **Number** | **2021** | **184.092497** | **255.950642** | **124.679095** |
| **Prevalence** | **Sao Tome and Principe** | **Female** | **55+ years** | **Low back pain** | **Number** | **2021** | **1688.899** | **2031.15898** | **1373.76065** |
| **Incidence** | **Sao Tome and Principe** | **Female** | **55+ years** | **Low back pain** | **Number** | **2021** | **713.28808** | **864.907037** | **583.110994** |
| **DALYs (Disability-Adjusted Life Years)** | **Saudi Arabia** | **Female** | **55+ years** | **Low back pain** | **Number** | **2021** | **25282.2693** | **35222.2248** | **16910.6575** |
| **Prevalence** | **Saudi Arabia** | **Female** | **55+ years** | **Low back pain** | **Number** | **2021** | **233237.925** | **281993.401** | **185347.149** |
| **Incidence** | **Saudi Arabia** | **Female** | **55+ years** | **Low back pain** | **Number** | **2021** | **95055.049** | **117518.92** | **75478.8595** |
| **DALYs (Disability-Adjusted Life Years)** | **Senegal** | **Female** | **55+ years** | **Low back pain** | **Number** | **2021** | **13322.4567** | **18225.5562** | **9036.73767** |
| **Prevalence** | **Senegal** | **Female** | **55+ years** | **Low back pain** | **Number** | **2021** | **123044.656** | **148760.017** | **100596.693** |
| **Incidence** | **Senegal** | **Female** | **55+ years** | **Low back pain** | **Number** | **2021** | **51366.3352** | **61031.6987** | **41988.6681** |
| **DALYs (Disability-Adjusted Life Years)** | **Serbia** | **Female** | **55+ years** | **Low back pain** | **Number** | **2021** | **55146.0692** | **74523.7016** | **38129.7197** |
| **Prevalence** | **Serbia** | **Female** | **55+ years** | **Low back pain** | **Number** | **2021** | **512174.413** | **588904.229** | **437170.743** |
| **Incidence** | **Serbia** | **Female** | **55+ years** | **Low back pain** | **Number** | **2021** | **180177.632** | **213493.742** | **151774.796** |
| **DALYs (Disability-Adjusted Life Years)** | **Seychelles** | **Female** | **55+ years** | **Low back pain** | **Number** | **2021** | **196.601981** | **269.028604** | **133.20274** |
| **Prevalence** | **Seychelles** | **Female** | **55+ years** | **Low back pain** | **Number** | **2021** | **1822.90788** | **2143.20028** | **1511.14533** |
| **Incidence** | **Seychelles** | **Female** | **55+ years** | **Low back pain** | **Number** | **2021** | **751.539544** | **901.817107** | **618.248124** |
| **DALYs (Disability-Adjusted Life Years)** | **Sierra Leone** | **Female** | **55+ years** | **Low back pain** | **Number** | **2021** | **6375.29894** | **8787.67659** | **4393.76126** |
| **Prevalence** | **Sierra Leone** | **Female** | **55+ years** | **Low back pain** | **Number** | **2021** | **58806.9572** | **71532.9741** | **48301.314** |
| **Incidence** | **Sierra Leone** | **Female** | **55+ years** | **Low back pain** | **Number** | **2021** | **23966.0002** | **28537.0882** | **19552.1012** |
| **DALYs (Disability-Adjusted Life Years)** | **Singapore** | **Female** | **55+ years** | **Low back pain** | **Number** | **2021** | **15943.0301** | **21666.2445** | **10748.5199** |
| **Prevalence** | **Singapore** | **Female** | **55+ years** | **Low back pain** | **Number** | **2021** | **143212.467** | **169728.604** | **119278.513** |
| **Incidence** | **Singapore** | **Female** | **55+ years** | **Low back pain** | **Number** | **2021** | **61276.4625** | **71851.6536** | **50806.426** |
| **DALYs (Disability-Adjusted Life Years)** | **Slovakia** | **Female** | **55+ years** | **Low back pain** | **Number** | **2021** | **33549.5243** | **45255.7997** | **23374.5058** |
| **Prevalence** | **Slovakia** | **Female** | **55+ years** | **Low back pain** | **Number** | **2021** | **311390.807** | **364382.722** | **263268.612** |
| **Incidence** | **Slovakia** | **Female** | **55+ years** | **Low back pain** | **Number** | **2021** | **109051.087** | **128289.794** | **90510.1459** |
| **DALYs (Disability-Adjusted Life Years)** | **Slovenia** | **Female** | **55+ years** | **Low back pain** | **Number** | **2021** | **13124.0671** | **17408.9929** | **9111.12547** |
| **Prevalence** | **Slovenia** | **Female** | **55+ years** | **Low back pain** | **Number** | **2021** | **122951.564** | **142099.508** | **105958.154** |
| **Incidence** | **Slovenia** | **Female** | **55+ years** | **Low back pain** | **Number** | **2021** | **44872.7101** | **52691.7201** | **37279.7147** |
| **DALYs (Disability-Adjusted Life Years)** | **Solomon Islands** | **Female** | **55+ years** | **Low back pain** | **Number** | **2021** | **600.378262** | **818.875681** | **410.018056** |
| **Prevalence** | **Solomon Islands** | **Female** | **55+ years** | **Low back pain** | **Number** | **2021** | **5523.59765** | **6653.56673** | **4547.24009** |
| **Incidence** | **Solomon Islands** | **Female** | **55+ years** | **Low back pain** | **Number** | **2021** | **2210.456** | **2646.65043** | **1787.9154** |
| **DALYs (Disability-Adjusted Life Years)** | **Somalia** | **Female** | **55+ years** | **Low back pain** | **Number** | **2021** | **13973.8728** | **19776.4149** | **9308.65568** |
| **Prevalence** | **Somalia** | **Female** | **55+ years** | **Low back pain** | **Number** | **2021** | **127728.132** | **153943.539** | **103433.172** |
| **Incidence** | **Somalia** | **Female** | **55+ years** | **Low back pain** | **Number** | **2021** | **50643.4666** | **61375.8179** | **40689.8816** |
| **DALYs (Disability-Adjusted Life Years)** | **South Africa** | **Female** | **55+ years** | **Low back pain** | **Number** | **2021** | **99239.1565** | **133564.951** | **67553.8599** |
| **Prevalence** | **South Africa** | **Female** | **55+ years** | **Low back pain** | **Number** | **2021** | **928227.973** | **1100491.33** | **762028.947** |
| **Incidence** | **South Africa** | **Female** | **55+ years** | **Low back pain** | **Number** | **2021** | **379534.345** | **451530.104** | **312218.42** |
| **DALYs (Disability-Adjusted Life Years)** | **South Sudan** | **Female** | **55+ years** | **Low back pain** | **Number** | **2021** | **6923.03026** | **9548.09525** | **4619.99096** |
| **Prevalence** | **South Sudan** | **Female** | **55+ years** | **Low back pain** | **Number** | **2021** | **64546.3683** | **78337.6855** | **52644.0338** |
| **Incidence** | **South Sudan** | **Female** | **55+ years** | **Low back pain** | **Number** | **2021** | **25897.3706** | **31212.0997** | **21136.2623** |
| **DALYs (Disability-Adjusted Life Years)** | **Spain** | **Female** | **55+ years** | **Low back pain** | **Number** | **2021** | **199824.44** | **271682.066** | **137980.462** |
| **Prevalence** | **Spain** | **Female** | **55+ years** | **Low back pain** | **Number** | **2021** | **1855209.04** | **2135407.34** | **1550632.74** |
| **Incidence** | **Spain** | **Female** | **55+ years** | **Low back pain** | **Number** | **2021** | **824574.639** | **973292.069** | **671385.567** |
| **DALYs (Disability-Adjusted Life Years)** | **Sri Lanka** | **Female** | **55+ years** | **Low back pain** | **Number** | **2021** | **50735.9956** | **69294.9535** | **34741.1902** |
| **Prevalence** | **Sri Lanka** | **Female** | **55+ years** | **Low back pain** | **Number** | **2021** | **472773.241** | **563598.381** | **395053.055** |
| **Incidence** | **Sri Lanka** | **Female** | **55+ years** | **Low back pain** | **Number** | **2021** | **194973.117** | **232426.649** | **159258.28** |
| **DALYs (Disability-Adjusted Life Years)** | **Sudan** | **Female** | **55+ years** | **Low back pain** | **Number** | **2021** | **31316.1435** | **42807.7095** | **21546.3436** |
| **Prevalence** | **Sudan** | **Female** | **55+ years** | **Low back pain** | **Number** | **2021** | **288743.309** | **339894.158** | **236868.599** |
| **Incidence** | **Sudan** | **Female** | **55+ years** | **Low back pain** | **Number** | **2021** | **116397.459** | **138374.51** | **95793.7646** |
| **DALYs (Disability-Adjusted Life Years)** | **Suriname** | **Female** | **55+ years** | **Low back pain** | **Number** | **2021** | **1103.25403** | **1512.2754** | **748.291919** |
| **Prevalence** | **Suriname** | **Female** | **55+ years** | **Low back pain** | **Number** | **2021** | **10181.1781** | **12236.0688** | **8364.91892** |
| **Incidence** | **Suriname** | **Female** | **55+ years** | **Low back pain** | **Number** | **2021** | **4135.68607** | **4958.03739** | **3384.38678** |
| **DALYs (Disability-Adjusted Life Years)** | **Sweden** | **Female** | **55+ years** | **Low back pain** | **Number** | **2021** | **39977.1163** | **54013.8227** | **27184.6087** |
| **Prevalence** | **Sweden** | **Female** | **55+ years** | **Low back pain** | **Number** | **2021** | **370883.64** | **439138.326** | **307743.071** |
| **Incidence** | **Sweden** | **Female** | **55+ years** | **Low back pain** | **Number** | **2021** | **157792.446** | **185508.153** | **130598.725** |
| **DALYs (Disability-Adjusted Life Years)** | **Switzerland** | **Female** | **55+ years** | **Low back pain** | **Number** | **2021** | **44681.7873** | **60066.4514** | **31306.0982** |
| **Prevalence** | **Switzerland** | **Female** | **55+ years** | **Low back pain** | **Number** | **2021** | **415103.405** | **475857.677** | **350084.483** |
| **Incidence** | **Switzerland** | **Female** | **55+ years** | **Low back pain** | **Number** | **2021** | **163350.357** | **190762.68** | **137672.527** |
| **DALYs (Disability-Adjusted Life Years)** | **Syrian Arab Republic** | **Female** | **55+ years** | **Low back pain** | **Number** | **2021** | **25478.9711** | **35145.7074** | **17563.3798** |
| **Prevalence** | **Syrian Arab Republic** | **Female** | **55+ years** | **Low back pain** | **Number** | **2021** | **235224.868** | **278114.553** | **194406.904** |
| **Incidence** | **Syrian Arab Republic** | **Female** | **55+ years** | **Low back pain** | **Number** | **2021** | **95582.8561** | **115063.925** | **78663.0569** |
| **DALYs (Disability-Adjusted Life Years)** | **Taiwan (Province of China)** | **Female** | **55+ years** | **Low back pain** | **Number** | **2021** | **108362.929** | **140440.894** | **79826.35** |
| **Prevalence** | **Taiwan (Province of China)** | **Female** | **55+ years** | **Low back pain** | **Number** | **2021** | **992650.537** | **1018031.84** | **966685.996** |
| **Incidence** | **Taiwan (Province of China)** | **Female** | **55+ years** | **Low back pain** | **Number** | **2021** | **373549.247** | **432702.237** | **321224.95** |
| **DALYs (Disability-Adjusted Life Years)** | **Tajikistan** | **Female** | **55+ years** | **Low back pain** | **Number** | **2021** | **13715.5671** | **19511.6326** | **9184.78747** |
| **Prevalence** | **Tajikistan** | **Female** | **55+ years** | **Low back pain** | **Number** | **2021** | **124226.015** | **147853.59** | **101261.005** |
| **Incidence** | **Tajikistan** | **Female** | **55+ years** | **Low back pain** | **Number** | **2021** | **49609.0318** | **60357.9097** | **39591.3234** |
| **DALYs (Disability-Adjusted Life Years)** | **Thailand** | **Female** | **55+ years** | **Low back pain** | **Number** | **2021** | **188951.95** | **261168.747** | **128948.839** |
| **Prevalence** | **Thailand** | **Female** | **55+ years** | **Low back pain** | **Number** | **2021** | **1737411.1** | **2075142.2** | **1442257.8** |
| **Incidence** | **Thailand** | **Female** | **55+ years** | **Low back pain** | **Number** | **2021** | **724920.994** | **856555.587** | **595908.444** |
| **DALYs (Disability-Adjusted Life Years)** | **Timor-Leste** | **Female** | **55+ years** | **Low back pain** | **Number** | **2021** | **1332.00779** | **1812.14959** | **916.101891** |
| **Prevalence** | **Timor-Leste** | **Female** | **55+ years** | **Low back pain** | **Number** | **2021** | **12294.7037** | **14372.8151** | **10211.7785** |
| **Incidence** | **Timor-Leste** | **Female** | **55+ years** | **Low back pain** | **Number** | **2021** | **5131.80778** | **6095.57635** | **4217.37672** |
| **DALYs (Disability-Adjusted Life Years)** | **Togo** | **Female** | **55+ years** | **Low back pain** | **Number** | **2021** | **7601.30439** | **10468.1623** | **5219.84683** |
| **Prevalence** | **Togo** | **Female** | **55+ years** | **Low back pain** | **Number** | **2021** | **69438.0136** | **83273.1528** | **56642.8192** |
| **Incidence** | **Togo** | **Female** | **55+ years** | **Low back pain** | **Number** | **2021** | **28436.014** | **34226.8209** | **23111.6681** |
| **DALYs (Disability-Adjusted Life Years)** | **Tokelau** | **Female** | **55+ years** | **Low back pain** | **Number** | **2021** | **2.90104322** | **3.89740065** | **2.02055208** |
| **Prevalence** | **Tokelau** | **Female** | **55+ years** | **Low back pain** | **Number** | **2021** | **27.1204812** | **32.0109879** | **22.5166816** |
| **Incidence** | **Tokelau** | **Female** | **55+ years** | **Low back pain** | **Number** | **2021** | **10.925431** | **13.0184633** | **9.06677647** |
| **DALYs (Disability-Adjusted Life Years)** | **Tonga** | **Female** | **55+ years** | **Low back pain** | **Number** | **2021** | **158.37629** | **215.926002** | **108.010326** |
| **Prevalence** | **Tonga** | **Female** | **55+ years** | **Low back pain** | **Number** | **2021** | **1471.32537** | **1740.84711** | **1216.45072** |
| **Incidence** | **Tonga** | **Female** | **55+ years** | **Low back pain** | **Number** | **2021** | **586.875803** | **688.343001** | **482.830776** |
| **DALYs (Disability-Adjusted Life Years)** | **Trinidad and Tobago** | **Female** | **55+ years** | **Low back pain** | **Number** | **2021** | **3364.72562** | **4572.80294** | **2322.87891** |
| **Prevalence** | **Trinidad and Tobago** | **Female** | **55+ years** | **Low back pain** | **Number** | **2021** | **31107.3643** | **37136.729** | **25715.9043** |
| **Incidence** | **Trinidad and Tobago** | **Female** | **55+ years** | **Low back pain** | **Number** | **2021** | **12656.4605** | **14994.1267** | **10570.6967** |
| **DALYs (Disability-Adjusted Life Years)** | **Tunisia** | **Female** | **55+ years** | **Low back pain** | **Number** | **2021** | **28112.4215** | **37984.6996** | **19757.592** |
| **Prevalence** | **Tunisia** | **Female** | **55+ years** | **Low back pain** | **Number** | **2021** | **262433.015** | **309202.673** | **218363.442** |
| **Incidence** | **Tunisia** | **Female** | **55+ years** | **Low back pain** | **Number** | **2021** | **104021.689** | **123741.216** | **85176.6981** |
| **DALYs (Disability-Adjusted Life Years)** | **Turkmenistan** | **Female** | **55+ years** | **Low back pain** | **Number** | **2021** | **10829.5376** | **14933.4452** | **7453.14941** |
| **Prevalence** | **Turkmenistan** | **Female** | **55+ years** | **Low back pain** | **Number** | **2021** | **98436.0133** | **116681.829** | **81294.4478** |
| **Incidence** | **Turkmenistan** | **Female** | **55+ years** | **Low back pain** | **Number** | **2021** | **38665.459** | **45942.266** | **31632.6077** |
| **DALYs (Disability-Adjusted Life Years)** | **Tuvalu** | **Female** | **55+ years** | **Low back pain** | **Number** | **2021** | **21.6380649** | **29.5021322** | **14.5809364** |
| **Prevalence** | **Tuvalu** | **Female** | **55+ years** | **Low back pain** | **Number** | **2021** | **199.688266** | **239.692316** | **164.532159** |
| **Incidence** | **Tuvalu** | **Female** | **55+ years** | **Low back pain** | **Number** | **2021** | **80.9337021** | **96.7572643** | **66.001077** |
| **DALYs (Disability-Adjusted Life Years)** | **T眉rkiye** | **Female** | **55+ years** | **Low back pain** | **Number** | **2021** | **195149.638** | **265214.004** | **135301.629** |
| **Prevalence** | **T眉rkiye** | **Female** | **55+ years** | **Low back pain** | **Number** | **2021** | **1810490** | **2144065.55** | **1501660.26** |
| **Incidence** | **T眉rkiye** | **Female** | **55+ years** | **Low back pain** | **Number** | **2021** | **733644.983** | **869463.539** | **602766.665** |
| **DALYs (Disability-Adjusted Life Years)** | **Uganda** | **Female** | **55+ years** | **Low back pain** | **Number** | **2021** | **33972.8498** | **47421.6741** | **23126.6306** |
| **Prevalence** | **Uganda** | **Female** | **55+ years** | **Low back pain** | **Number** | **2021** | **311099.092** | **371910.661** | **255252.304** |
| **Incidence** | **Uganda** | **Female** | **55+ years** | **Low back pain** | **Number** | **2021** | **121989.638** | **147754.066** | **99247.9259** |
| **DALYs (Disability-Adjusted Life Years)** | **Ukraine** | **Female** | **55+ years** | **Low back pain** | **Number** | **2021** | **334743.511** | **462519.707** | **236505.696** |
| **Prevalence** | **Ukraine** | **Female** | **55+ years** | **Low back pain** | **Number** | **2021** | **3116075.15** | **3581597.63** | **2681091.29** |
| **Incidence** | **Ukraine** | **Female** | **55+ years** | **Low back pain** | **Number** | **2021** | **1094212.12** | **1285509.68** | **908919.752** |
| **DALYs (Disability-Adjusted Life Years)** | **United Arab Emirates** | **Female** | **55+ years** | **Low back pain** | **Number** | **2021** | **3219.91549** | **4588.58819** | **2152.58102** |
| **Prevalence** | **United Arab Emirates** | **Female** | **55+ years** | **Low back pain** | **Number** | **2021** | **29477.4299** | **37468.3609** | **23192.8752** |
| **Incidence** | **United Arab Emirates** | **Female** | **55+ years** | **Low back pain** | **Number** | **2021** | **12335.0174** | **15307.2314** | **9595.89522** |
| **DALYs (Disability-Adjusted Life Years)** | **United Kingdom** | **Female** | **55+ years** | **Low back pain** | **Number** | **2021** | **314292.126** | **416860.03** | **219137.505** |
| **Prevalence** | **United Kingdom** | **Female** | **55+ years** | **Low back pain** | **Number** | **2021** | **2914970.77** | **3365496.72** | **2475604.01** |
| **Incidence** | **United Kingdom** | **Female** | **55+ years** | **Low back pain** | **Number** | **2021** | **1175560.62** | **1364560.17** | **984229.574** |
| **DALYs (Disability-Adjusted Life Years)** | **United Republic of Tanzania** | **Female** | **55+ years** | **Low back pain** | **Number** | **2021** | **53016.1971** | **72831.6308** | **36160.8678** |
| **Prevalence** | **United Republic of Tanzania** | **Female** | **55+ years** | **Low back pain** | **Number** | **2021** | **487949.851** | **582237.004** | **404493.518** |
| **Incidence** | **United Republic of Tanzania** | **Female** | **55+ years** | **Low back pain** | **Number** | **2021** | **193904.974** | **232344.289** | **158575.666** |
| **DALYs (Disability-Adjusted Life Years)** | **United States of America** | **Female** | **55+ years** | **Low back pain** | **Number** | **2021** | **1378945.56** | **1790647.15** | **985178.625** |
| **Prevalence** | **United States of America** | **Female** | **55+ years** | **Low back pain** | **Number** | **2021** | **13030896.6** | **14242257.1** | **11913172.5** |
| **Incidence** | **United States of America** | **Female** | **55+ years** | **Low back pain** | **Number** | **2021** | **5269099.75** | **5907877.4** | **4634837.15** |
| **DALYs (Disability-Adjusted Life Years)** | **United States Virgin Islands** | **Female** | **55+ years** | **Low back pain** | **Number** | **2021** | **321.523932** | **434.021755** | **220.685921** |
| **Prevalence** | **United States Virgin Islands** | **Female** | **55+ years** | **Low back pain** | **Number** | **2021** | **2976.86331** | **3511.1915** | **2478.12105** |
| **Incidence** | **United States Virgin Islands** | **Female** | **55+ years** | **Low back pain** | **Number** | **2021** | **1207.90625** | **1442.18592** | **997.067277** |
| **DALYs (Disability-Adjusted Life Years)** | **Uruguay** | **Female** | **55+ years** | **Low back pain** | **Number** | **2021** | **15248.0083** | **20529.4524** | **10546.6891** |
| **Prevalence** | **Uruguay** | **Female** | **55+ years** | **Low back pain** | **Number** | **2021** | **141338.355** | **165059.156** | **120637.041** |
| **Incidence** | **Uruguay** | **Female** | **55+ years** | **Low back pain** | **Number** | **2021** | **55632.5812** | **65311.196** | **46770.2651** |
| **DALYs (Disability-Adjusted Life Years)** | **Uzbekistan** | **Female** | **55+ years** | **Low back pain** | **Number** | **2021** | **69546.3032** | **97243.3857** | **47555.4662** |
| **Prevalence** | **Uzbekistan** | **Female** | **55+ years** | **Low back pain** | **Number** | **2021** | **635350.387** | **766565.639** | **523599.563** |
| **Incidence** | **Uzbekistan** | **Female** | **55+ years** | **Low back pain** | **Number** | **2021** | **248493.427** | **296028.608** | **205392.404** |
| **DALYs (Disability-Adjusted Life Years)** | **Vanuatu** | **Female** | **55+ years** | **Low back pain** | **Number** | **2021** | **342.87048** | **475.545296** | **235.986173** |
| **Prevalence** | **Vanuatu** | **Female** | **55+ years** | **Low back pain** | **Number** | **2021** | **3134.71144** | **3735.95102** | **2556.1403** |
| **Incidence** | **Vanuatu** | **Female** | **55+ years** | **Low back pain** | **Number** | **2021** | **1232.22827** | **1487.71443** | **997.961211** |
| **DALYs (Disability-Adjusted Life Years)** | **Venezuela (Bolivarian Republic of)** | **Female** | **55+ years** | **Low back pain** | **Number** | **2021** | **54551.6405** | **74462.6189** | **37367.1152** |
| **Prevalence** | **Venezuela (Bolivarian Republic of)** | **Female** | **55+ years** | **Low back pain** | **Number** | **2021** | **500393.277** | **598273.585** | **412794.642** |
| **Incidence** | **Venezuela (Bolivarian Republic of)** | **Female** | **55+ years** | **Low back pain** | **Number** | **2021** | **204426.933** | **244976.666** | **167686.865** |
| **DALYs (Disability-Adjusted Life Years)** | **Viet Nam** | **Female** | **55+ years** | **Low back pain** | **Number** | **2021** | **199131.465** | **273376.888** | **137092.075** |
| **Prevalence** | **Viet Nam** | **Female** | **55+ years** | **Low back pain** | **Number** | **2021** | **1810287.11** | **2164973.26** | **1500518.18** |
| **Incidence** | **Viet Nam** | **Female** | **55+ years** | **Low back pain** | **Number** | **2021** | **725991.035** | **861505.57** | **600683.527** |
| **DALYs (Disability-Adjusted Life Years)** | **Yemen** | **Female** | **55+ years** | **Low back pain** | **Number** | **2021** | **25437.9887** | **34877.666** | **17325.9076** |
| **Prevalence** | **Yemen** | **Female** | **55+ years** | **Low back pain** | **Number** | **2021** | **236230.844** | **279615.355** | **195175.334** |
| **Incidence** | **Yemen** | **Female** | **55+ years** | **Low back pain** | **Number** | **2021** | **95899.3005** | **114742.959** | **79061.4659** |
| **DALYs (Disability-Adjusted Life Years)** | **Zambia** | **Female** | **55+ years** | **Low back pain** | **Number** | **2021** | **13020.0313** | **17757.6326** | **8892.79018** |
| **Prevalence** | **Zambia** | **Female** | **55+ years** | **Low back pain** | **Number** | **2021** | **119945.847** | **143732.055** | **97853.0659** |
| **Incidence** | **Zambia** | **Female** | **55+ years** | **Low back pain** | **Number** | **2021** | **49180.5608** | **59123.3387** | **39614.6789** |
| **DALYs (Disability-Adjusted Life Years)** | **Zimbabwe** | **Female** | **55+ years** | **Low back pain** | **Number** | **2021** | **15950.8502** | **22406.6494** | **10709.2073** |
| **Prevalence** | **Zimbabwe** | **Female** | **55+ years** | **Low back pain** | **Number** | **2021** | **146201.556** | **177603.496** | **118714.515** |
| **Incidence** | **Zimbabwe** | **Female** | **55+ years** | **Low back pain** | **Number** | **2021** | **57523.8553** | **69738.5189** | **46304.928** |

Appendix6: National analysis of age-standardized incidence rate/age-standardized prevalence rate/age-standardized DALY rate of low back pain in postmenopausal women in 2021

| measure | location | sex | age | cause | metric | year | val | upper | lower |
| --- | --- | --- | --- | --- | --- | --- | --- | --- | --- |
| DALYs (Disability-Adjusted Life Years) | Afghanistan | Female | Age-standardized | Low back pain | Rate | 2021 | 358.587702 | 489.542284 | 249.104684 |
| Prevalence | Afghanistan | Female | Age-standardized | Low back pain | Rate | 2021 | 3407.1125 | 4088.32902 | 2796.76128 |
| Incidence | Afghanistan | Female | Age-standardized | Low back pain | Rate | 2021 | 1379.04293 | 1647.67019 | 1125.411 |
| DALYs (Disability-Adjusted Life Years) | Albania | Female | Age-standardized | Low back pain | Rate | 2021 | 592.401242 | 818.136315 | 407.504641 |
| Prevalence | Albania | Female | Age-standardized | Low back pain | Rate | 2021 | 5416.76447 | 6292.25424 | 4572.96505 |
| Incidence | Albania | Female | Age-standardized | Low back pain | Rate | 2021 | 1909.1009 | 2251.73203 | 1577.98708 |
| DALYs (Disability-Adjusted Life Years) | Algeria | Female | Age-standardized | Low back pain | Rate | 2021 | 360.9509 | 495.162673 | 244.683296 |
| Prevalence | Algeria | Female | Age-standardized | Low back pain | Rate | 2021 | 3347.65033 | 4001.03892 | 2724.29678 |
| Incidence | Algeria | Female | Age-standardized | Low back pain | Rate | 2021 | 1359.49112 | 1631.15508 | 1109.31964 |
| DALYs (Disability-Adjusted Life Years) | American Samoa | Female | Age-standardized | Low back pain | Rate | 2021 | 353.125713 | 485.409876 | 242.58872 |
| Prevalence | American Samoa | Female | Age-standardized | Low back pain | Rate | 2021 | 3293.28746 | 3947.13258 | 2698.71975 |
| Incidence | American Samoa | Female | Age-standardized | Low back pain | Rate | 2021 | 1330.23902 | 1604.87091 | 1092.24208 |
| DALYs (Disability-Adjusted Life Years) | Andorra | Female | Age-standardized | Low back pain | Rate | 2021 | 435.17458 | 594.277814 | 298.127018 |
| Prevalence | Andorra | Female | Age-standardized | Low back pain | Rate | 2021 | 4012.64496 | 4686.0627 | 3349.93217 |
| Incidence | Andorra | Female | Age-standardized | Low back pain | Rate | 2021 | 1639.09076 | 1930.08935 | 1384.92941 |
| DALYs (Disability-Adjusted Life Years) | Angola | Female | Age-standardized | Low back pain | Rate | 2021 | 375.058959 | 517.162779 | 258.30342 |
| Prevalence | Angola | Female | Age-standardized | Low back pain | Rate | 2021 | 3434.71751 | 4119.07637 | 2801.42636 |
| Incidence | Angola | Female | Age-standardized | Low back pain | Rate | 2021 | 1390.91704 | 1666.33377 | 1127.76118 |
| DALYs (Disability-Adjusted Life Years) | Antigua and Barbuda | Female | Age-standardized | Low back pain | Rate | 2021 | 293.680211 | 401.476051 | 201.879839 |
| Prevalence | Antigua and Barbuda | Female | Age-standardized | Low back pain | Rate | 2021 | 2694.67057 | 3219.14708 | 2190.48234 |
| Incidence | Antigua and Barbuda | Female | Age-standardized | Low back pain | Rate | 2021 | 1107.32143 | 1333.28093 | 907.367263 |
| DALYs (Disability-Adjusted Life Years) | Argentina | Female | Age-standardized | Low back pain | Rate | 2021 | 479.747568 | 650.673769 | 331.240438 |
| Prevalence | Argentina | Female | Age-standardized | Low back pain | Rate | 2021 | 4421.72261 | 5127.82871 | 3712.17005 |
| Incidence | Argentina | Female | Age-standardized | Low back pain | Rate | 2021 | 1744.89656 | 2062.63046 | 1446.79507 |
| DALYs (Disability-Adjusted Life Years) | Armenia | Female | Age-standardized | Low back pain | Rate | 2021 | 497.769093 | 683.660781 | 345.80424 |
| Prevalence | Armenia | Female | Age-standardized | Low back pain | Rate | 2021 | 4588.79969 | 5347.9959 | 3877.88944 |
| Incidence | Armenia | Female | Age-standardized | Low back pain | Rate | 2021 | 1722.99557 | 2047.05542 | 1416.31218 |
| DALYs (Disability-Adjusted Life Years) | Australia | Female | Age-standardized | Low back pain | Rate | 2021 | 530.330036 | 708.195471 | 363.448897 |
| Prevalence | Australia | Female | Age-standardized | Low back pain | Rate | 2021 | 4900.67034 | 5668.13773 | 4135.45664 |
| Incidence | Australia | Female | Age-standardized | Low back pain | Rate | 2021 | 1898.70293 | 2216.26677 | 1576.01195 |
| DALYs (Disability-Adjusted Life Years) | Austria | Female | Age-standardized | Low back pain | Rate | 2021 | 410.352928 | 555.508661 | 285.070897 |
| Prevalence | Austria | Female | Age-standardized | Low back pain | Rate | 2021 | 3797.1437 | 4378.87743 | 3226.79933 |
| Incidence | Austria | Female | Age-standardized | Low back pain | Rate | 2021 | 1586.73139 | 1862.57959 | 1325.34375 |
| DALYs (Disability-Adjusted Life Years) | Azerbaijan | Female | Age-standardized | Low back pain | Rate | 2021 | 454.408572 | 641.897435 | 311.817868 |
| Prevalence | Azerbaijan | Female | Age-standardized | Low back pain | Rate | 2021 | 4135.83164 | 4961.64477 | 3418.71611 |
| Incidence | Azerbaijan | Female | Age-standardized | Low back pain | Rate | 2021 | 1606.56512 | 1914.50312 | 1316.26373 |
| DALYs (Disability-Adjusted Life Years) | Bahamas | Female | Age-standardized | Low back pain | Rate | 2021 | 293.80568 | 407.753184 | 198.89026 |
| Prevalence | Bahamas | Female | Age-standardized | Low back pain | Rate | 2021 | 2679.33382 | 3234.25651 | 2183.17279 |
| Incidence | Bahamas | Female | Age-standardized | Low back pain | Rate | 2021 | 1101.92128 | 1333.57094 | 901.498044 |
| DALYs (Disability-Adjusted Life Years) | Bahrain | Female | Age-standardized | Low back pain | Rate | 2021 | 340.795629 | 465.944764 | 229.662583 |
| Prevalence | Bahrain | Female | Age-standardized | Low back pain | Rate | 2021 | 3189.57424 | 3854.65276 | 2565.50016 |
| Incidence | Bahrain | Female | Age-standardized | Low back pain | Rate | 2021 | 1311.48058 | 1584.50107 | 1054.1496 |
| DALYs (Disability-Adjusted Life Years) | Bangladesh | Female | Age-standardized | Low back pain | Rate | 2021 | 490.657492 | 681.834584 | 340.848011 |
| Prevalence | Bangladesh | Female | Age-standardized | Low back pain | Rate | 2021 | 4552.15428 | 5301.77772 | 3790.17672 |
| Incidence | Bangladesh | Female | Age-standardized | Low back pain | Rate | 2021 | 1661.67024 | 2003.45795 | 1350.11114 |
| DALYs (Disability-Adjusted Life Years) | Barbados | Female | Age-standardized | Low back pain | Rate | 2021 | 300.77336 | 406.231101 | 204.153941 |
| Prevalence | Barbados | Female | Age-standardized | Low back pain | Rate | 2021 | 2765.69434 | 3250.54515 | 2280.61554 |
| Incidence | Barbados | Female | Age-standardized | Low back pain | Rate | 2021 | 1129.20698 | 1338.83969 | 931.937574 |
| DALYs (Disability-Adjusted Life Years) | Belarus | Female | Age-standardized | Low back pain | Rate | 2021 | 565.527569 | 755.896505 | 401.643306 |
| Prevalence | Belarus | Female | Age-standardized | Low back pain | Rate | 2021 | 5244.05131 | 6045.63052 | 4481.20745 |
| Incidence | Belarus | Female | Age-standardized | Low back pain | Rate | 2021 | 1863.88021 | 2182.84118 | 1526.13352 |
| DALYs (Disability-Adjusted Life Years) | Belgium | Female | Age-standardized | Low back pain | Rate | 2021 | 450.769045 | 605.926888 | 315.627354 |
| Prevalence | Belgium | Female | Age-standardized | Low back pain | Rate | 2021 | 4205.79749 | 4882.71832 | 3515.92947 |
| Incidence | Belgium | Female | Age-standardized | Low back pain | Rate | 2021 | 1692.15589 | 1964.0522 | 1429.9417 |
| DALYs (Disability-Adjusted Life Years) | Belize | Female | Age-standardized | Low back pain | Rate | 2021 | 304.322498 | 419.420107 | 203.301911 |
| Prevalence | Belize | Female | Age-standardized | Low back pain | Rate | 2021 | 2773.49502 | 3358.28506 | 2251.77019 |
| Incidence | Belize | Female | Age-standardized | Low back pain | Rate | 2021 | 1128.45784 | 1355.54251 | 916.618408 |
| DALYs (Disability-Adjusted Life Years) | Benin | Female | Age-standardized | Low back pain | Rate | 2021 | 360.314613 | 493.434356 | 244.872515 |
| Prevalence | Benin | Female | Age-standardized | Low back pain | Rate | 2021 | 3321.66052 | 3977.98255 | 2747.23273 |
| Incidence | Benin | Female | Age-standardized | Low back pain | Rate | 2021 | 1340.78939 | 1633.7603 | 1092.80708 |
| DALYs (Disability-Adjusted Life Years) | Bermuda | Female | Age-standardized | Low back pain | Rate | 2021 | 309.754133 | 420.356845 | 212.654054 |
| Prevalence | Bermuda | Female | Age-standardized | Low back pain | Rate | 2021 | 2836.96818 | 3335.93872 | 2374.38602 |
| Incidence | Bermuda | Female | Age-standardized | Low back pain | Rate | 2021 | 1149.84027 | 1365.91668 | 949.404639 |
| DALYs (Disability-Adjusted Life Years) | Bhutan | Female | Age-standardized | Low back pain | Rate | 2021 | 486.696137 | 663.199449 | 336.42929 |
| Prevalence | Bhutan | Female | Age-standardized | Low back pain | Rate | 2021 | 4497.06802 | 5311.00479 | 3763.14617 |
| Incidence | Bhutan | Female | Age-standardized | Low back pain | Rate | 2021 | 1656.68798 | 1989.02069 | 1355.12589 |
| DALYs (Disability-Adjusted Life Years) | Bolivia (Plurinational State of) | Female | Age-standardized | Low back pain | Rate | 2021 | 294.917091 | 397.40753 | 202.294861 |
| Prevalence | Bolivia (Plurinational State of) | Female | Age-standardized | Low back pain | Rate | 2021 | 2721.54068 | 3244.57266 | 2223.20754 |
| Incidence | Bolivia (Plurinational State of) | Female | Age-standardized | Low back pain | Rate | 2021 | 1114.63318 | 1331.71516 | 916.50653 |
| DALYs (Disability-Adjusted Life Years) | Bosnia and Herzegovina | Female | Age-standardized | Low back pain | Rate | 2021 | 551.800452 | 750.06904 | 380.714735 |
| Prevalence | Bosnia and Herzegovina | Female | Age-standardized | Low back pain | Rate | 2021 | 5142.58951 | 5988.529 | 4365.97822 |
| Incidence | Bosnia and Herzegovina | Female | Age-standardized | Low back pain | Rate | 2021 | 1871.48087 | 2199.10475 | 1554.34221 |
| DALYs (Disability-Adjusted Life Years) | Botswana | Female | Age-standardized | Low back pain | Rate | 2021 | 360.011462 | 494.336138 | 245.243796 |
| Prevalence | Botswana | Female | Age-standardized | Low back pain | Rate | 2021 | 3331.31498 | 3970.00999 | 2736.56302 |
| Incidence | Botswana | Female | Age-standardized | Low back pain | Rate | 2021 | 1346.65589 | 1619.33717 | 1088.72398 |
| DALYs (Disability-Adjusted Life Years) | Brazil | Female | Age-standardized | Low back pain | Rate | 2021 | 435.633239 | 592.123796 | 304.977857 |
| Prevalence | Brazil | Female | Age-standardized | Low back pain | Rate | 2021 | 4067.60821 | 4792.14706 | 3407.90523 |
| Incidence | Brazil | Female | Age-standardized | Low back pain | Rate | 2021 | 1553.06926 | 1819.73202 | 1281.43918 |
| DALYs (Disability-Adjusted Life Years) | Brunei Darussalam | Female | Age-standardized | Low back pain | Rate | 2021 | 378.790673 | 521.061385 | 260.314323 |
| Prevalence | Brunei Darussalam | Female | Age-standardized | Low back pain | Rate | 2021 | 3438.64267 | 4136.24067 | 2810.13282 |
| Incidence | Brunei Darussalam | Female | Age-standardized | Low back pain | Rate | 2021 | 1425.24296 | 1710.69586 | 1149.87504 |
| DALYs (Disability-Adjusted Life Years) | Bulgaria | Female | Age-standardized | Low back pain | Rate | 2021 | 569.302217 | 772.15715 | 394.382387 |
| Prevalence | Bulgaria | Female | Age-standardized | Low back pain | Rate | 2021 | 5297.9136 | 6135.41752 | 4537.05588 |
| Incidence | Bulgaria | Female | Age-standardized | Low back pain | Rate | 2021 | 1911.70333 | 2268.29731 | 1586.46391 |
| DALYs (Disability-Adjusted Life Years) | Burkina Faso | Female | Age-standardized | Low back pain | Rate | 2021 | 368.818099 | 515.740007 | 253.925334 |
| Prevalence | Burkina Faso | Female | Age-standardized | Low back pain | Rate | 2021 | 3364.49357 | 3982.37184 | 2743.1114 |
| Incidence | Burkina Faso | Female | Age-standardized | Low back pain | Rate | 2021 | 1358.14343 | 1625.00535 | 1091.45135 |
| DALYs (Disability-Adjusted Life Years) | Burundi | Female | Age-standardized | Low back pain | Rate | 2021 | 413.49074 | 572.64491 | 282.114798 |
| Prevalence | Burundi | Female | Age-standardized | Low back pain | Rate | 2021 | 3760.56027 | 4523.35069 | 3106.77327 |
| Incidence | Burundi | Female | Age-standardized | Low back pain | Rate | 2021 | 1493.44695 | 1803.62232 | 1225.41941 |
| DALYs (Disability-Adjusted Life Years) | Cabo Verde | Female | Age-standardized | Low back pain | Rate | 2021 | 334.993752 | 453.097729 | 230.113487 |
| Prevalence | Cabo Verde | Female | Age-standardized | Low back pain | Rate | 2021 | 3080.41805 | 3677.099 | 2509.89685 |
| Incidence | Cabo Verde | Female | Age-standardized | Low back pain | Rate | 2021 | 1280.33788 | 1532.66064 | 1041.58801 |
| DALYs (Disability-Adjusted Life Years) | Cambodia | Female | Age-standardized | Low back pain | Rate | 2021 | 333.376855 | 463.674997 | 226.970707 |
| Prevalence | Cambodia | Female | Age-standardized | Low back pain | Rate | 2021 | 3061.37364 | 3652.35309 | 2514.16963 |
| Incidence | Cambodia | Female | Age-standardized | Low back pain | Rate | 2021 | 1224.71093 | 1469.43137 | 992.944421 |
| DALYs (Disability-Adjusted Life Years) | Cameroon | Female | Age-standardized | Low back pain | Rate | 2021 | 369.335469 | 508.956461 | 247.44501 |
| Prevalence | Cameroon | Female | Age-standardized | Low back pain | Rate | 2021 | 3391.16643 | 4023.57406 | 2750.66456 |
| Incidence | Cameroon | Female | Age-standardized | Low back pain | Rate | 2021 | 1363.87863 | 1644.1285 | 1114.04366 |
| DALYs (Disability-Adjusted Life Years) | Canada | Female | Age-standardized | Low back pain | Rate | 2021 | 364.369614 | 496.08072 | 249.162416 |
| Prevalence | Canada | Female | Age-standardized | Low back pain | Rate | 2021 | 3354.58411 | 3924.10755 | 2843.20862 |
| Incidence | Canada | Female | Age-standardized | Low back pain | Rate | 2021 | 1389.23803 | 1649.42805 | 1158.47591 |
| DALYs (Disability-Adjusted Life Years) | Central African Republic | Female | Age-standardized | Low back pain | Rate | 2021 | 376.052349 | 528.776253 | 254.806618 |
| Prevalence | Central African Republic | Female | Age-standardized | Low back pain | Rate | 2021 | 3468.75952 | 4209.91354 | 2797.58623 |
| Incidence | Central African Republic | Female | Age-standardized | Low back pain | Rate | 2021 | 1403.36426 | 1710.86117 | 1122.39125 |
| DALYs (Disability-Adjusted Life Years) | Chad | Female | Age-standardized | Low back pain | Rate | 2021 | 392.022184 | 553.051477 | 265.697376 |
| Prevalence | Chad | Female | Age-standardized | Low back pain | Rate | 2021 | 3608.31822 | 4347.14245 | 2918.04199 |
| Incidence | Chad | Female | Age-standardized | Low back pain | Rate | 2021 | 1420.18544 | 1701.42717 | 1162.59217 |
| DALYs (Disability-Adjusted Life Years) | Chile | Female | Age-standardized | Low back pain | Rate | 2021 | 479.10875 | 647.037914 | 330.499156 |
| Prevalence | Chile | Female | Age-standardized | Low back pain | Rate | 2021 | 4437.88986 | 5219.78008 | 3748.96287 |
| Incidence | Chile | Female | Age-standardized | Low back pain | Rate | 2021 | 1753.30806 | 2065.35698 | 1451.47643 |
| DALYs (Disability-Adjusted Life Years) | China | Female | Age-standardized | Low back pain | Rate | 2021 | 317.027354 | 429.767915 | 214.551106 |
| Prevalence | China | Female | Age-standardized | Low back pain | Rate | 2021 | 2900.72516 | 3402.94927 | 2422.91475 |
| Incidence | China | Female | Age-standardized | Low back pain | Rate | 2021 | 1203.26012 | 1419.3356 | 995.850775 |
| DALYs (Disability-Adjusted Life Years) | Colombia | Female | Age-standardized | Low back pain | Rate | 2021 | 360.916368 | 495.43781 | 245.226363 |
| Prevalence | Colombia | Female | Age-standardized | Low back pain | Rate | 2021 | 3304.62668 | 3930.19518 | 2774.19359 |
| Incidence | Colombia | Female | Age-standardized | Low back pain | Rate | 2021 | 1292.93561 | 1520.33803 | 1072.29129 |
| DALYs (Disability-Adjusted Life Years) | Comoros | Female | Age-standardized | Low back pain | Rate | 2021 | 400.475295 | 551.749244 | 273.856556 |
| Prevalence | Comoros | Female | Age-standardized | Low back pain | Rate | 2021 | 3676.03515 | 4375.73001 | 3055.07954 |
| Incidence | Comoros | Female | Age-standardized | Low back pain | Rate | 2021 | 1476.9552 | 1789.16947 | 1205.12669 |
| DALYs (Disability-Adjusted Life Years) | Congo | Female | Age-standardized | Low back pain | Rate | 2021 | 360.800668 | 499.159595 | 242.643974 |
| Prevalence | Congo | Female | Age-standardized | Low back pain | Rate | 2021 | 3320.1274 | 3968.77445 | 2675.95744 |
| Incidence | Congo | Female | Age-standardized | Low back pain | Rate | 2021 | 1361.59978 | 1644.74491 | 1120.0902 |
| DALYs (Disability-Adjusted Life Years) | Cook Islands | Female | Age-standardized | Low back pain | Rate | 2021 | 364.224074 | 490.934515 | 250.652359 |
| Prevalence | Cook Islands | Female | Age-standardized | Low back pain | Rate | 2021 | 3404.42111 | 3976.95678 | 2832.02249 |
| Incidence | Cook Islands | Female | Age-standardized | Low back pain | Rate | 2021 | 1359.43397 | 1616.85837 | 1119.75296 |
| DALYs (Disability-Adjusted Life Years) | Costa Rica | Female | Age-standardized | Low back pain | Rate | 2021 | 340.339004 | 465.812859 | 234.510145 |
| Prevalence | Costa Rica | Female | Age-standardized | Low back pain | Rate | 2021 | 3134.7134 | 3711.96755 | 2613.54957 |
| Incidence | Costa Rica | Female | Age-standardized | Low back pain | Rate | 2021 | 1250.67768 | 1493.23514 | 1030.21042 |
| DALYs (Disability-Adjusted Life Years) | Croatia | Female | Age-standardized | Low back pain | Rate | 2021 | 551.32586 | 741.407718 | 387.544218 |
| Prevalence | Croatia | Female | Age-standardized | Low back pain | Rate | 2021 | 5152.04407 | 5975.12161 | 4336.76178 |
| Incidence | Croatia | Female | Age-standardized | Low back pain | Rate | 2021 | 1879.94034 | 2203.29385 | 1543.92757 |
| DALYs (Disability-Adjusted Life Years) | Cuba | Female | Age-standardized | Low back pain | Rate | 2021 | 294.163944 | 389.461951 | 208.26892 |
| Prevalence | Cuba | Female | Age-standardized | Low back pain | Rate | 2021 | 2703.57068 | 3062.61136 | 2369.91212 |
| Incidence | Cuba | Female | Age-standardized | Low back pain | Rate | 2021 | 1112.31293 | 1274.59657 | 942.827936 |
| DALYs (Disability-Adjusted Life Years) | Cyprus | Female | Age-standardized | Low back pain | Rate | 2021 | 448.024709 | 601.025452 | 304.269457 |
| Prevalence | Cyprus | Female | Age-standardized | Low back pain | Rate | 2021 | 4108.55941 | 4813.64432 | 3455.21833 |
| Incidence | Cyprus | Female | Age-standardized | Low back pain | Rate | 2021 | 1678.45327 | 1978.22206 | 1397.1322 |
| DALYs (Disability-Adjusted Life Years) | Czechia | Female | Age-standardized | Low back pain | Rate | 2021 | 606.121062 | 826.323825 | 422.582957 |
| Prevalence | Czechia | Female | Age-standardized | Low back pain | Rate | 2021 | 5689.89923 | 6566.09745 | 4839.3691 |
| Incidence | Czechia | Female | Age-standardized | Low back pain | Rate | 2021 | 1962.63028 | 2297.84379 | 1630.25159 |
| DALYs (Disability-Adjusted Life Years) | C么te d'Ivoire | Female | Age-standardized | Low back pain | Rate | 2021 | 369.618765 | 517.931766 | 254.767814 |
| Prevalence | C么te d'Ivoire | Female | Age-standardized | Low back pain | Rate | 2021 | 3390.13943 | 4070.72039 | 2756.52646 |
| Incidence | C么te d'Ivoire | Female | Age-standardized | Low back pain | Rate | 2021 | 1364.39184 | 1624.48432 | 1086.59345 |
| DALYs (Disability-Adjusted Life Years) | Democratic People's Republic of Korea | Female | Age-standardized | Low back pain | Rate | 2021 | 389.938699 | 530.367169 | 267.849136 |
| Prevalence | Democratic People's Republic of Korea | Female | Age-standardized | Low back pain | Rate | 2021 | 3553.08869 | 4240.18977 | 2963.93426 |
| Incidence | Democratic People's Republic of Korea | Female | Age-standardized | Low back pain | Rate | 2021 | 1404.98484 | 1651.74083 | 1158.95924 |
| DALYs (Disability-Adjusted Life Years) | Democratic Republic of the Congo | Female | Age-standardized | Low back pain | Rate | 2021 | 379.791311 | 523.673093 | 254.450092 |
| Prevalence | Democratic Republic of the Congo | Female | Age-standardized | Low back pain | Rate | 2021 | 3493.02837 | 4181.95451 | 2843.82108 |
| Incidence | Democratic Republic of the Congo | Female | Age-standardized | Low back pain | Rate | 2021 | 1408.5411 | 1690.73851 | 1149.11451 |
| DALYs (Disability-Adjusted Life Years) | Denmark | Female | Age-standardized | Low back pain | Rate | 2021 | 519.738519 | 723.325131 | 353.968439 |
| Prevalence | Denmark | Female | Age-standardized | Low back pain | Rate | 2021 | 4772.65832 | 5605.38557 | 3899.73855 |
| Incidence | Denmark | Female | Age-standardized | Low back pain | Rate | 2021 | 1684.59506 | 2090.25329 | 1294.82595 |
| DALYs (Disability-Adjusted Life Years) | Djibouti | Female | Age-standardized | Low back pain | Rate | 2021 | 375.323284 | 523.94956 | 253.676029 |
| Prevalence | Djibouti | Female | Age-standardized | Low back pain | Rate | 2021 | 3409.41757 | 4077.65548 | 2758.1573 |
| Incidence | Djibouti | Female | Age-standardized | Low back pain | Rate | 2021 | 1403.18167 | 1692.08565 | 1118.40208 |
| DALYs (Disability-Adjusted Life Years) | Dominica | Female | Age-standardized | Low back pain | Rate | 2021 | 303.404001 | 416.547032 | 207.969959 |
| Prevalence | Dominica | Female | Age-standardized | Low back pain | Rate | 2021 | 2793.41106 | 3298.05211 | 2294.2601 |
| Incidence | Dominica | Female | Age-standardized | Low back pain | Rate | 2021 | 1137.53481 | 1363.60221 | 943.512812 |
| DALYs (Disability-Adjusted Life Years) | Dominican Republic | Female | Age-standardized | Low back pain | Rate | 2021 | 307.647834 | 417.463044 | 211.110319 |
| Prevalence | Dominican Republic | Female | Age-standardized | Low back pain | Rate | 2021 | 2824.78038 | 3340.12101 | 2352.76042 |
| Incidence | Dominican Republic | Female | Age-standardized | Low back pain | Rate | 2021 | 1141.11606 | 1354.45075 | 927.64755 |
| DALYs (Disability-Adjusted Life Years) | Ecuador | Female | Age-standardized | Low back pain | Rate | 2021 | 270.441637 | 359.098899 | 188.346685 |
| Prevalence | Ecuador | Female | Age-standardized | Low back pain | Rate | 2021 | 2493.55466 | 2895.45237 | 2097.75594 |
| Incidence | Ecuador | Female | Age-standardized | Low back pain | Rate | 2021 | 1050.12919 | 1235.29365 | 870.353431 |
| DALYs (Disability-Adjusted Life Years) | Egypt | Female | Age-standardized | Low back pain | Rate | 2021 | 363.276235 | 511.501041 | 244.365375 |
| Prevalence | Egypt | Female | Age-standardized | Low back pain | Rate | 2021 | 3342.533 | 4051.05929 | 2722.64374 |
| Incidence | Egypt | Female | Age-standardized | Low back pain | Rate | 2021 | 1355.36537 | 1638.1305 | 1076.99137 |
| DALYs (Disability-Adjusted Life Years) | El Salvador | Female | Age-standardized | Low back pain | Rate | 2021 | 345.793395 | 465.845143 | 236.428043 |
| Prevalence | El Salvador | Female | Age-standardized | Low back pain | Rate | 2021 | 3187.41555 | 3765.6394 | 2663.37704 |
| Incidence | El Salvador | Female | Age-standardized | Low back pain | Rate | 2021 | 1266.96648 | 1496.96864 | 1055.5671 |
| DALYs (Disability-Adjusted Life Years) | Equatorial Guinea | Female | Age-standardized | Low back pain | Rate | 2021 | 364.50146 | 502.29721 | 248.831566 |
| Prevalence | Equatorial Guinea | Female | Age-standardized | Low back pain | Rate | 2021 | 3368.91806 | 4046.93456 | 2752.62547 |
| Incidence | Equatorial Guinea | Female | Age-standardized | Low back pain | Rate | 2021 | 1374.96062 | 1653.7177 | 1107.34375 |
| DALYs (Disability-Adjusted Life Years) | Eritrea | Female | Age-standardized | Low back pain | Rate | 2021 | 366.73005 | 502.412355 | 247.272659 |
| Prevalence | Eritrea | Female | Age-standardized | Low back pain | Rate | 2021 | 3358.53898 | 4038.43624 | 2722.94271 |
| Incidence | Eritrea | Female | Age-standardized | Low back pain | Rate | 2021 | 1386.07106 | 1676.35314 | 1110.02517 |
| DALYs (Disability-Adjusted Life Years) | Estonia | Female | Age-standardized | Low back pain | Rate | 2021 | 577.131564 | 767.82964 | 403.221688 |
| Prevalence | Estonia | Female | Age-standardized | Low back pain | Rate | 2021 | 5392.09348 | 6207.62109 | 4619.46363 |
| Incidence | Estonia | Female | Age-standardized | Low back pain | Rate | 2021 | 1900.86009 | 2215.86875 | 1576.36894 |
| DALYs (Disability-Adjusted Life Years) | Eswatini | Female | Age-standardized | Low back pain | Rate | 2021 | 330.802334 | 456.780466 | 224.673065 |
| Prevalence | Eswatini | Female | Age-standardized | Low back pain | Rate | 2021 | 3071.1536 | 3681.34625 | 2514.40183 |
| Incidence | Eswatini | Female | Age-standardized | Low back pain | Rate | 2021 | 1277.46637 | 1517.22372 | 1042.83733 |
| DALYs (Disability-Adjusted Life Years) | Ethiopia | Female | Age-standardized | Low back pain | Rate | 2021 | 404.053791 | 550.264102 | 278.896286 |
| Prevalence | Ethiopia | Female | Age-standardized | Low back pain | Rate | 2021 | 3734.39852 | 4412.01279 | 3112.73395 |
| Incidence | Ethiopia | Female | Age-standardized | Low back pain | Rate | 2021 | 1513.75662 | 1788.38891 | 1246.87457 |
| DALYs (Disability-Adjusted Life Years) | Fiji | Female | Age-standardized | Low back pain | Rate | 2021 | 342.337908 | 474.087182 | 232.380241 |
| Prevalence | Fiji | Female | Age-standardized | Low back pain | Rate | 2021 | 3176.43217 | 3828.819 | 2584.73937 |
| Incidence | Fiji | Female | Age-standardized | Low back pain | Rate | 2021 | 1297.24137 | 1561.16827 | 1051.55183 |
| DALYs (Disability-Adjusted Life Years) | Finland | Female | Age-standardized | Low back pain | Rate | 2021 | 405.959501 | 549.176703 | 279.570412 |
| Prevalence | Finland | Female | Age-standardized | Low back pain | Rate | 2021 | 3774.75804 | 4403.1835 | 3166.92631 |
| Incidence | Finland | Female | Age-standardized | Low back pain | Rate | 2021 | 1583.36128 | 1894.79212 | 1327.44054 |
| DALYs (Disability-Adjusted Life Years) | France | Female | Age-standardized | Low back pain | Rate | 2021 | 467.28424 | 621.651831 | 326.619361 |
| Prevalence | France | Female | Age-standardized | Low back pain | Rate | 2021 | 4350.54679 | 5013.85007 | 3710.0634 |
| Incidence | France | Female | Age-standardized | Low back pain | Rate | 2021 | 1728.84755 | 2017.41701 | 1458.46402 |
| DALYs (Disability-Adjusted Life Years) | Gabon | Female | Age-standardized | Low back pain | Rate | 2021 | 356.90921 | 490.35431 | 244.264477 |
| Prevalence | Gabon | Female | Age-standardized | Low back pain | Rate | 2021 | 3298.98186 | 3923.74115 | 2754.95594 |
| Incidence | Gabon | Female | Age-standardized | Low back pain | Rate | 2021 | 1354.98447 | 1631.55699 | 1113.55569 |
| DALYs (Disability-Adjusted Life Years) | Gambia | Female | Age-standardized | Low back pain | Rate | 2021 | 335.981935 | 464.156153 | 230.913734 |
| Prevalence | Gambia | Female | Age-standardized | Low back pain | Rate | 2021 | 3125.31101 | 3741.69945 | 2579.11214 |
| Incidence | Gambia | Female | Age-standardized | Low back pain | Rate | 2021 | 1290.24672 | 1550.98771 | 1049.27071 |
| DALYs (Disability-Adjusted Life Years) | Georgia | Female | Age-standardized | Low back pain | Rate | 2021 | 437.966628 | 586.928866 | 304.076493 |
| Prevalence | Georgia | Female | Age-standardized | Low back pain | Rate | 2021 | 4071.37196 | 4745.05613 | 3427.79837 |
| Incidence | Georgia | Female | Age-standardized | Low back pain | Rate | 2021 | 1608.72486 | 1897.54689 | 1352.94277 |
| DALYs (Disability-Adjusted Life Years) | Germany | Female | Age-standardized | Low back pain | Rate | 2021 | 514.882901 | 690.683976 | 360.594185 |
| Prevalence | Germany | Female | Age-standardized | Low back pain | Rate | 2021 | 4796.87047 | 5549.39507 | 4114.09663 |
| Incidence | Germany | Female | Age-standardized | Low back pain | Rate | 2021 | 1821.22618 | 2113.27391 | 1537.17475 |
| DALYs (Disability-Adjusted Life Years) | Ghana | Female | Age-standardized | Low back pain | Rate | 2021 | 309.98627 | 412.683574 | 217.935539 |
| Prevalence | Ghana | Female | Age-standardized | Low back pain | Rate | 2021 | 2825.69749 | 3330.13573 | 2377.85196 |
| Incidence | Ghana | Female | Age-standardized | Low back pain | Rate | 2021 | 1200.80421 | 1416.76085 | 1001.42029 |
| DALYs (Disability-Adjusted Life Years) | Greece | Female | Age-standardized | Low back pain | Rate | 2021 | 437.929073 | 585.652582 | 307.094751 |
| Prevalence | Greece | Female | Age-standardized | Low back pain | Rate | 2021 | 4059.53819 | 4692.4729 | 3433.69982 |
| Incidence | Greece | Female | Age-standardized | Low back pain | Rate | 2021 | 1656.47999 | 1921.47246 | 1390.86583 |
| DALYs (Disability-Adjusted Life Years) | Greenland | Female | Age-standardized | Low back pain | Rate | 2021 | 354.676768 | 493.54867 | 236.69267 |
| Prevalence | Greenland | Female | Age-standardized | Low back pain | Rate | 2021 | 3219.88214 | 3866.89864 | 2596.6052 |
| Incidence | Greenland | Female | Age-standardized | Low back pain | Rate | 2021 | 1344.85574 | 1651.38097 | 1078.40431 |
| DALYs (Disability-Adjusted Life Years) | Grenada | Female | Age-standardized | Low back pain | Rate | 2021 | 301.571194 | 411.350055 | 205.419448 |
| Prevalence | Grenada | Female | Age-standardized | Low back pain | Rate | 2021 | 2776.71198 | 3301.55698 | 2287.14073 |
| Incidence | Grenada | Female | Age-standardized | Low back pain | Rate | 2021 | 1132.03582 | 1352.63857 | 933.067472 |
| DALYs (Disability-Adjusted Life Years) | Guam | Female | Age-standardized | Low back pain | Rate | 2021 | 357.389933 | 484.328573 | 245.296987 |
| Prevalence | Guam | Female | Age-standardized | Low back pain | Rate | 2021 | 3279.7887 | 3862.48623 | 2739.28142 |
| Incidence | Guam | Female | Age-standardized | Low back pain | Rate | 2021 | 1325.51815 | 1577.35691 | 1094.92566 |
| DALYs (Disability-Adjusted Life Years) | Guatemala | Female | Age-standardized | Low back pain | Rate | 2021 | 377.690691 | 517.012717 | 259.442904 |
| Prevalence | Guatemala | Female | Age-standardized | Low back pain | Rate | 2021 | 3502.3697 | 4150.35414 | 2933.31856 |
| Incidence | Guatemala | Female | Age-standardized | Low back pain | Rate | 2021 | 1347.34154 | 1600.79752 | 1098.53252 |
| DALYs (Disability-Adjusted Life Years) | Guinea | Female | Age-standardized | Low back pain | Rate | 2021 | 368.410932 | 512.02253 | 251.505707 |
| Prevalence | Guinea | Female | Age-standardized | Low back pain | Rate | 2021 | 3383.95453 | 4072.79177 | 2772.2764 |
| Incidence | Guinea | Female | Age-standardized | Low back pain | Rate | 2021 | 1367.74564 | 1627.30968 | 1126.66054 |
| DALYs (Disability-Adjusted Life Years) | Guinea-Bissau | Female | Age-standardized | Low back pain | Rate | 2021 | 348.14782 | 492.267638 | 236.281315 |
| Prevalence | Guinea-Bissau | Female | Age-standardized | Low back pain | Rate | 2021 | 3190.60952 | 3879.54671 | 2602.5486 |
| Incidence | Guinea-Bissau | Female | Age-standardized | Low back pain | Rate | 2021 | 1315.95085 | 1581.00457 | 1060.92461 |
| DALYs (Disability-Adjusted Life Years) | Guyana | Female | Age-standardized | Low back pain | Rate | 2021 | 291.191727 | 406.491021 | 201.694943 |
| Prevalence | Guyana | Female | Age-standardized | Low back pain | Rate | 2021 | 2710.18585 | 3264.2993 | 2193.45317 |
| Incidence | Guyana | Female | Age-standardized | Low back pain | Rate | 2021 | 1113.85756 | 1343.41919 | 913.230509 |
| DALYs (Disability-Adjusted Life Years) | Haiti | Female | Age-standardized | Low back pain | Rate | 2021 | 291.581698 | 402.264909 | 198.64009 |
| Prevalence | Haiti | Female | Age-standardized | Low back pain | Rate | 2021 | 2694.82498 | 3260.99442 | 2189.41083 |
| Incidence | Haiti | Female | Age-standardized | Low back pain | Rate | 2021 | 1107.50655 | 1341.56851 | 887.584504 |
| DALYs (Disability-Adjusted Life Years) | Honduras | Female | Age-standardized | Low back pain | Rate | 2021 | 345.858258 | 468.213575 | 239.803229 |
| Prevalence | Honduras | Female | Age-standardized | Low back pain | Rate | 2021 | 3184.19839 | 3777.4549 | 2607.91497 |
| Incidence | Honduras | Female | Age-standardized | Low back pain | Rate | 2021 | 1268.18937 | 1539.90942 | 1035.47949 |
| DALYs (Disability-Adjusted Life Years) | Hungary | Female | Age-standardized | Low back pain | Rate | 2021 | 627.995995 | 853.817502 | 436.218442 |
| Prevalence | Hungary | Female | Age-standardized | Low back pain | Rate | 2021 | 5872.17666 | 6731.37011 | 5030.74229 |
| Incidence | Hungary | Female | Age-standardized | Low back pain | Rate | 2021 | 1984.32809 | 2315.86886 | 1654.94462 |
| DALYs (Disability-Adjusted Life Years) | Iceland | Female | Age-standardized | Low back pain | Rate | 2021 | 458.440984 | 620.473968 | 317.513682 |
| Prevalence | Iceland | Female | Age-standardized | Low back pain | Rate | 2021 | 4197.54768 | 4887.17684 | 3535.81881 |
| Incidence | Iceland | Female | Age-standardized | Low back pain | Rate | 2021 | 1697.68663 | 2001.6182 | 1414.33819 |
| DALYs (Disability-Adjusted Life Years) | India | Female | Age-standardized | Low back pain | Rate | 2021 | 383.979949 | 524.615166 | 260.65978 |
| Prevalence | India | Female | Age-standardized | Low back pain | Rate | 2021 | 3610.11249 | 4297.50285 | 2982.93068 |
| Incidence | India | Female | Age-standardized | Low back pain | Rate | 2021 | 1440.63119 | 1710.63743 | 1183.88656 |
| DALYs (Disability-Adjusted Life Years) | Indonesia | Female | Age-standardized | Low back pain | Rate | 2021 | 363.969695 | 503.513058 | 245.579304 |
| Prevalence | Indonesia | Female | Age-standardized | Low back pain | Rate | 2021 | 3318.37152 | 3958.01605 | 2729.2508 |
| Incidence | Indonesia | Female | Age-standardized | Low back pain | Rate | 2021 | 1323.69687 | 1575.55758 | 1076.23411 |
| DALYs (Disability-Adjusted Life Years) | Iran (Islamic Republic of) | Female | Age-standardized | Low back pain | Rate | 2021 | 412.007686 | 563.211511 | 282.869247 |
| Prevalence | Iran (Islamic Republic of) | Female | Age-standardized | Low back pain | Rate | 2021 | 3841.20265 | 4544.49288 | 3196.25169 |
| Incidence | Iran (Islamic Republic of) | Female | Age-standardized | Low back pain | Rate | 2021 | 1512.78156 | 1777.2478 | 1246.22555 |
| DALYs (Disability-Adjusted Life Years) | Iraq | Female | Age-standardized | Low back pain | Rate | 2021 | 354.744381 | 486.247861 | 242.4686 |
| Prevalence | Iraq | Female | Age-standardized | Low back pain | Rate | 2021 | 3321.28829 | 3945.57041 | 2751.78739 |
| Incidence | Iraq | Female | Age-standardized | Low back pain | Rate | 2021 | 1349.54312 | 1603.88048 | 1096.50838 |
| DALYs (Disability-Adjusted Life Years) | Ireland | Female | Age-standardized | Low back pain | Rate | 2021 | 454.883222 | 615.79179 | 319.248555 |
| Prevalence | Ireland | Female | Age-standardized | Low back pain | Rate | 2021 | 4179.87792 | 4805.70682 | 3540.60953 |
| Incidence | Ireland | Female | Age-standardized | Low back pain | Rate | 2021 | 1697.27453 | 2005.17971 | 1411.61974 |
| DALYs (Disability-Adjusted Life Years) | Israel | Female | Age-standardized | Low back pain | Rate | 2021 | 464.137187 | 624.166026 | 321.620753 |
| Prevalence | Israel | Female | Age-standardized | Low back pain | Rate | 2021 | 4272.30858 | 5000.4338 | 3599.38991 |
| Incidence | Israel | Female | Age-standardized | Low back pain | Rate | 2021 | 1718.4165 | 2010.10117 | 1424.25349 |
| DALYs (Disability-Adjusted Life Years) | Italy | Female | Age-standardized | Low back pain | Rate | 2021 | 457.578725 | 607.022664 | 320.917047 |
| Prevalence | Italy | Female | Age-standardized | Low back pain | Rate | 2021 | 4256.48782 | 4892.28009 | 3597.1815 |
| Incidence | Italy | Female | Age-standardized | Low back pain | Rate | 2021 | 1714.28874 | 1985.10092 | 1433.61539 |
| DALYs (Disability-Adjusted Life Years) | Jamaica | Female | Age-standardized | Low back pain | Rate | 2021 | 310.213218 | 424.260312 | 211.2251 |
| Prevalence | Jamaica | Female | Age-standardized | Low back pain | Rate | 2021 | 2848.04269 | 3403.31916 | 2358.61985 |
| Incidence | Jamaica | Female | Age-standardized | Low back pain | Rate | 2021 | 1146.34802 | 1355.2808 | 944.375626 |
| DALYs (Disability-Adjusted Life Years) | Japan | Female | Age-standardized | Low back pain | Rate | 2021 | 480.144209 | 639.520356 | 336.04138 |
| Prevalence | Japan | Female | Age-standardized | Low back pain | Rate | 2021 | 4431.62485 | 5065.94073 | 3786.34742 |
| Incidence | Japan | Female | Age-standardized | Low back pain | Rate | 2021 | 1716.3741 | 1996.83243 | 1445.04962 |
| DALYs (Disability-Adjusted Life Years) | Jordan | Female | Age-standardized | Low back pain | Rate | 2021 | 359.446206 | 491.992005 | 244.705124 |
| Prevalence | Jordan | Female | Age-standardized | Low back pain | Rate | 2021 | 3354.84532 | 4023.80177 | 2708.69378 |
| Incidence | Jordan | Female | Age-standardized | Low back pain | Rate | 2021 | 1361.60523 | 1650.50811 | 1090.58938 |
| DALYs (Disability-Adjusted Life Years) | Kazakhstan | Female | Age-standardized | Low back pain | Rate | 2021 | 474.83121 | 664.527234 | 325.315134 |
| Prevalence | Kazakhstan | Female | Age-standardized | Low back pain | Rate | 2021 | 4381.29821 | 5168.09145 | 3637.22148 |
| Incidence | Kazakhstan | Female | Age-standardized | Low back pain | Rate | 2021 | 1676.39694 | 1980.54931 | 1376.28687 |
| DALYs (Disability-Adjusted Life Years) | Kenya | Female | Age-standardized | Low back pain | Rate | 2021 | 459.443184 | 629.586001 | 315.852303 |
| Prevalence | Kenya | Female | Age-standardized | Low back pain | Rate | 2021 | 4237.89656 | 5021.08282 | 3509.33026 |
| Incidence | Kenya | Female | Age-standardized | Low back pain | Rate | 2021 | 1672.26439 | 1986.43197 | 1382.18011 |
| DALYs (Disability-Adjusted Life Years) | Kiribati | Female | Age-standardized | Low back pain | Rate | 2021 | 358.30198 | 495.643215 | 239.702322 |
| Prevalence | Kiribati | Female | Age-standardized | Low back pain | Rate | 2021 | 3308.98792 | 3993.53524 | 2669.95351 |
| Incidence | Kiribati | Female | Age-standardized | Low back pain | Rate | 2021 | 1331.54735 | 1585.32103 | 1072.92117 |
| DALYs (Disability-Adjusted Life Years) | Kuwait | Female | Age-standardized | Low back pain | Rate | 2021 | 348.46243 | 483.701272 | 232.663172 |
| Prevalence | Kuwait | Female | Age-standardized | Low back pain | Rate | 2021 | 3233.56187 | 3908.32615 | 2627.04808 |
| Incidence | Kuwait | Female | Age-standardized | Low back pain | Rate | 2021 | 1307.10152 | 1584.89494 | 1059.99135 |
| DALYs (Disability-Adjusted Life Years) | Kyrgyzstan | Female | Age-standardized | Low back pain | Rate | 2021 | 454.573486 | 628.731849 | 309.660795 |
| Prevalence | Kyrgyzstan | Female | Age-standardized | Low back pain | Rate | 2021 | 4140.84063 | 4890.58128 | 3410.43335 |
| Incidence | Kyrgyzstan | Female | Age-standardized | Low back pain | Rate | 2021 | 1614.35759 | 1917.61267 | 1315.18778 |
| DALYs (Disability-Adjusted Life Years) | Lao People's Democratic Republic | Female | Age-standardized | Low back pain | Rate | 2021 | 315.519191 | 431.499037 | 216.734401 |
| Prevalence | Lao People's Democratic Republic | Female | Age-standardized | Low back pain | Rate | 2021 | 2875.79884 | 3425.67881 | 2358.41421 |
| Incidence | Lao People's Democratic Republic | Female | Age-standardized | Low back pain | Rate | 2021 | 1174.54745 | 1405.87728 | 946.913023 |
| DALYs (Disability-Adjusted Life Years) | Latvia | Female | Age-standardized | Low back pain | Rate | 2021 | 566.947524 | 767.951588 | 399.409037 |
| Prevalence | Latvia | Female | Age-standardized | Low back pain | Rate | 2021 | 5308.8193 | 6091.3377 | 4548.5523 |
| Incidence | Latvia | Female | Age-standardized | Low back pain | Rate | 2021 | 1888.04359 | 2230.08655 | 1576.10653 |
| DALYs (Disability-Adjusted Life Years) | Lebanon | Female | Age-standardized | Low back pain | Rate | 2021 | 365.970526 | 492.879948 | 255.489203 |
| Prevalence | Lebanon | Female | Age-standardized | Low back pain | Rate | 2021 | 3457.63224 | 4094.92494 | 2923.77701 |
| Incidence | Lebanon | Female | Age-standardized | Low back pain | Rate | 2021 | 1384.71774 | 1629.75626 | 1160.51923 |
| DALYs (Disability-Adjusted Life Years) | Lesotho | Female | Age-standardized | Low back pain | Rate | 2021 | 348.740659 | 480.450761 | 239.266718 |
| Prevalence | Lesotho | Female | Age-standardized | Low back pain | Rate | 2021 | 3257.22996 | 3883.24303 | 2689.43193 |
| Incidence | Lesotho | Female | Age-standardized | Low back pain | Rate | 2021 | 1337.11681 | 1597.62416 | 1081.16977 |
| DALYs (Disability-Adjusted Life Years) | Liberia | Female | Age-standardized | Low back pain | Rate | 2021 | 342.486367 | 472.784871 | 234.263476 |
| Prevalence | Liberia | Female | Age-standardized | Low back pain | Rate | 2021 | 3210.4244 | 3869.82124 | 2650.20898 |
| Incidence | Liberia | Female | Age-standardized | Low back pain | Rate | 2021 | 1313.36422 | 1576.07955 | 1067.30803 |
| DALYs (Disability-Adjusted Life Years) | Libya | Female | Age-standardized | Low back pain | Rate | 2021 | 353.424715 | 487.307819 | 242.714822 |
| Prevalence | Libya | Female | Age-standardized | Low back pain | Rate | 2021 | 3291.20589 | 3919.10096 | 2720.2885 |
| Incidence | Libya | Female | Age-standardized | Low back pain | Rate | 2021 | 1338.91659 | 1615.13891 | 1082.89022 |
| DALYs (Disability-Adjusted Life Years) | Lithuania | Female | Age-standardized | Low back pain | Rate | 2021 | 558.455704 | 752.741198 | 387.828388 |
| Prevalence | Lithuania | Female | Age-standardized | Low back pain | Rate | 2021 | 5230.2808 | 6095.125 | 4448.02702 |
| Incidence | Lithuania | Female | Age-standardized | Low back pain | Rate | 2021 | 1875.21366 | 2173.41758 | 1576.39719 |
| DALYs (Disability-Adjusted Life Years) | Luxembourg | Female | Age-standardized | Low back pain | Rate | 2021 | 474.170565 | 634.004122 | 332.887737 |
| Prevalence | Luxembourg | Female | Age-standardized | Low back pain | Rate | 2021 | 4364.87708 | 5037.17101 | 3729.91648 |
| Incidence | Luxembourg | Female | Age-standardized | Low back pain | Rate | 2021 | 1730.77773 | 2021.74044 | 1455.12879 |
| DALYs (Disability-Adjusted Life Years) | Madagascar | Female | Age-standardized | Low back pain | Rate | 2021 | 403.52835 | 564.844363 | 272.845336 |
| Prevalence | Madagascar | Female | Age-standardized | Low back pain | Rate | 2021 | 3672.10512 | 4433.56268 | 3000.01325 |
| Incidence | Madagascar | Female | Age-standardized | Low back pain | Rate | 2021 | 1466.26191 | 1773.78159 | 1175.12042 |
| DALYs (Disability-Adjusted Life Years) | Malawi | Female | Age-standardized | Low back pain | Rate | 2021 | 407.284588 | 561.294837 | 277.851234 |
| Prevalence | Malawi | Female | Age-standardized | Low back pain | Rate | 2021 | 3744.96315 | 4477.06877 | 3082.11838 |
| Incidence | Malawi | Female | Age-standardized | Low back pain | Rate | 2021 | 1496.94591 | 1804.09394 | 1228.15986 |
| DALYs (Disability-Adjusted Life Years) | Malaysia | Female | Age-standardized | Low back pain | Rate | 2021 | 311.427172 | 425.12025 | 212.449018 |
| Prevalence | Malaysia | Female | Age-standardized | Low back pain | Rate | 2021 | 2872.31399 | 3394.85117 | 2367.83815 |
| Incidence | Malaysia | Female | Age-standardized | Low back pain | Rate | 2021 | 1178.38488 | 1402.51894 | 973.725898 |
| DALYs (Disability-Adjusted Life Years) | Maldives | Female | Age-standardized | Low back pain | Rate | 2021 | 295.205599 | 400.108496 | 201.039677 |
| Prevalence | Maldives | Female | Age-standardized | Low back pain | Rate | 2021 | 2700.96911 | 3249.54841 | 2221.70913 |
| Incidence | Maldives | Female | Age-standardized | Low back pain | Rate | 2021 | 1119.04622 | 1345.30848 | 908.283722 |
| DALYs (Disability-Adjusted Life Years) | Mali | Female | Age-standardized | Low back pain | Rate | 2021 | 330.342248 | 450.861666 | 224.448457 |
| Prevalence | Mali | Female | Age-standardized | Low back pain | Rate | 2021 | 3047.63805 | 3670.6404 | 2450.85546 |
| Incidence | Mali | Female | Age-standardized | Low back pain | Rate | 2021 | 1271.4496 | 1536.32416 | 1021.40198 |
| DALYs (Disability-Adjusted Life Years) | Malta | Female | Age-standardized | Low back pain | Rate | 2021 | 500.812287 | 665.779509 | 353.562734 |
| Prevalence | Malta | Female | Age-standardized | Low back pain | Rate | 2021 | 4608.53477 | 5240.13138 | 3916.44108 |
| Incidence | Malta | Female | Age-standardized | Low back pain | Rate | 2021 | 1798.60664 | 2080.54985 | 1524.42061 |
| DALYs (Disability-Adjusted Life Years) | Marshall Islands | Female | Age-standardized | Low back pain | Rate | 2021 | 329.972331 | 467.813794 | 225.057247 |
| Prevalence | Marshall Islands | Female | Age-standardized | Low back pain | Rate | 2021 | 3045.81373 | 3675.44536 | 2464.87002 |
| Incidence | Marshall Islands | Female | Age-standardized | Low back pain | Rate | 2021 | 1255.72485 | 1527.6786 | 998.26582 |
| DALYs (Disability-Adjusted Life Years) | Mauritania | Female | Age-standardized | Low back pain | Rate | 2021 | 344.630026 | 477.370781 | 234.138702 |
| Prevalence | Mauritania | Female | Age-standardized | Low back pain | Rate | 2021 | 3144.47105 | 3812.52725 | 2566.50413 |
| Incidence | Mauritania | Female | Age-standardized | Low back pain | Rate | 2021 | 1295.47946 | 1548.82542 | 1047.46576 |
| DALYs (Disability-Adjusted Life Years) | Mauritius | Female | Age-standardized | Low back pain | Rate | 2021 | 319.340678 | 426.490096 | 215.418357 |
| Prevalence | Mauritius | Female | Age-standardized | Low back pain | Rate | 2021 | 2977.08377 | 3529.76662 | 2478.11671 |
| Incidence | Mauritius | Female | Age-standardized | Low back pain | Rate | 2021 | 1211.59965 | 1449.51809 | 992.316245 |
| DALYs (Disability-Adjusted Life Years) | Mexico | Female | Age-standardized | Low back pain | Rate | 2021 | 326.645399 | 449.023946 | 221.087847 |
| Prevalence | Mexico | Female | Age-standardized | Low back pain | Rate | 2021 | 3022.99896 | 3609.43908 | 2476.01073 |
| Incidence | Mexico | Female | Age-standardized | Low back pain | Rate | 2021 | 1221.61801 | 1455.81343 | 998.941016 |
| DALYs (Disability-Adjusted Life Years) | Micronesia (Federated States of) | Female | Age-standardized | Low back pain | Rate | 2021 | 356.479579 | 487.556885 | 240.050816 |
| Prevalence | Micronesia (Federated States of) | Female | Age-standardized | Low back pain | Rate | 2021 | 3281.21229 | 4001.20527 | 2673.79725 |
| Incidence | Micronesia (Federated States of) | Female | Age-standardized | Low back pain | Rate | 2021 | 1323.99948 | 1576.68499 | 1062.6054 |
| DALYs (Disability-Adjusted Life Years) | Monaco | Female | Age-standardized | Low back pain | Rate | 2021 | 448.456477 | 604.824409 | 308.884947 |
| Prevalence | Monaco | Female | Age-standardized | Low back pain | Rate | 2021 | 4126.58628 | 4754.92857 | 3492.44112 |
| Incidence | Monaco | Female | Age-standardized | Low back pain | Rate | 2021 | 1677.50447 | 1962.79098 | 1409.51224 |
| DALYs (Disability-Adjusted Life Years) | Mongolia | Female | Age-standardized | Low back pain | Rate | 2021 | 444.322206 | 608.134128 | 303.055903 |
| Prevalence | Mongolia | Female | Age-standardized | Low back pain | Rate | 2021 | 4039.6303 | 4846.83743 | 3327.72446 |
| Incidence | Mongolia | Female | Age-standardized | Low back pain | Rate | 2021 | 1588.56792 | 1900.76393 | 1297.61035 |
| DALYs (Disability-Adjusted Life Years) | Montenegro | Female | Age-standardized | Low back pain | Rate | 2021 | 575.156735 | 789.988033 | 394.134759 |
| Prevalence | Montenegro | Female | Age-standardized | Low back pain | Rate | 2021 | 5314.41649 | 6196.80429 | 4396.89282 |
| Incidence | Montenegro | Female | Age-standardized | Low back pain | Rate | 2021 | 1905.40854 | 2233.31156 | 1590.87776 |
| DALYs (Disability-Adjusted Life Years) | Morocco | Female | Age-standardized | Low back pain | Rate | 2021 | 403.782486 | 555.99922 | 280.605341 |
| Prevalence | Morocco | Female | Age-standardized | Low back pain | Rate | 2021 | 3790.73527 | 4484.72167 | 3113.29036 |
| Incidence | Morocco | Female | Age-standardized | Low back pain | Rate | 2021 | 1465.11064 | 1742.49232 | 1198.04165 |
| DALYs (Disability-Adjusted Life Years) | Mozambique | Female | Age-standardized | Low back pain | Rate | 2021 | 423.68142 | 579.151378 | 292.258165 |
| Prevalence | Mozambique | Female | Age-standardized | Low back pain | Rate | 2021 | 3941.50038 | 4714.48376 | 3220.52832 |
| Incidence | Mozambique | Female | Age-standardized | Low back pain | Rate | 2021 | 1535.22855 | 1844.49086 | 1248.93636 |
| DALYs (Disability-Adjusted Life Years) | Myanmar | Female | Age-standardized | Low back pain | Rate | 2021 | 276.565266 | 373.177124 | 189.329122 |
| Prevalence | Myanmar | Female | Age-standardized | Low back pain | Rate | 2021 | 2546.08339 | 3026.44204 | 2097.25814 |
| Incidence | Myanmar | Female | Age-standardized | Low back pain | Rate | 2021 | 1075.72598 | 1284.51205 | 883.318484 |
| DALYs (Disability-Adjusted Life Years) | Namibia | Female | Age-standardized | Low back pain | Rate | 2021 | 376.104444 | 512.56996 | 258.288367 |
| Prevalence | Namibia | Female | Age-standardized | Low back pain | Rate | 2021 | 3448.46693 | 4116.32005 | 2802.45702 |
| Incidence | Namibia | Female | Age-standardized | Low back pain | Rate | 2021 | 1379.50916 | 1661.02343 | 1123.94059 |
| DALYs (Disability-Adjusted Life Years) | Nauru | Female | Age-standardized | Low back pain | Rate | 2021 | 361.481364 | 501.304384 | 245.964857 |
| Prevalence | Nauru | Female | Age-standardized | Low back pain | Rate | 2021 | 3348.87063 | 4022.90793 | 2708.77267 |
| Incidence | Nauru | Female | Age-standardized | Low back pain | Rate | 2021 | 1349.88162 | 1647.54339 | 1090.97268 |
| DALYs (Disability-Adjusted Life Years) | Nepal | Female | Age-standardized | Low back pain | Rate | 2021 | 532.502226 | 738.971185 | 372.238717 |
| Prevalence | Nepal | Female | Age-standardized | Low back pain | Rate | 2021 | 4972.29127 | 5892.70367 | 4182.57655 |
| Incidence | Nepal | Female | Age-standardized | Low back pain | Rate | 2021 | 1732.70619 | 2076.23335 | 1420.76699 |
| DALYs (Disability-Adjusted Life Years) | Netherlands | Female | Age-standardized | Low back pain | Rate | 2021 | 404.519771 | 543.787245 | 275.008104 |
| Prevalence | Netherlands | Female | Age-standardized | Low back pain | Rate | 2021 | 3730.94554 | 4327.33066 | 3164.99205 |
| Incidence | Netherlands | Female | Age-standardized | Low back pain | Rate | 2021 | 1570.03022 | 1855.02474 | 1325.85296 |
| DALYs (Disability-Adjusted Life Years) | New Zealand | Female | Age-standardized | Low back pain | Rate | 2021 | 574.593016 | 779.386103 | 407.468867 |
| Prevalence | New Zealand | Female | Age-standardized | Low back pain | Rate | 2021 | 5293.56719 | 6080.69238 | 4504.76409 |
| Incidence | New Zealand | Female | Age-standardized | Low back pain | Rate | 2021 | 2062.67213 | 2405.11534 | 1721.13741 |
| DALYs (Disability-Adjusted Life Years) | Nicaragua | Female | Age-standardized | Low back pain | Rate | 2021 | 347.574938 | 477.270693 | 240.471607 |
| Prevalence | Nicaragua | Female | Age-standardized | Low back pain | Rate | 2021 | 3202.18051 | 3797.43571 | 2655.41315 |
| Incidence | Nicaragua | Female | Age-standardized | Low back pain | Rate | 2021 | 1269.06912 | 1519.24226 | 1050.66297 |
| DALYs (Disability-Adjusted Life Years) | Niger | Female | Age-standardized | Low back pain | Rate | 2021 | 365.812499 | 505.837583 | 250.730905 |
| Prevalence | Niger | Female | Age-standardized | Low back pain | Rate | 2021 | 3343.71182 | 4010.72686 | 2725.0675 |
| Incidence | Niger | Female | Age-standardized | Low back pain | Rate | 2021 | 1357.54531 | 1635.14528 | 1092.10598 |
| DALYs (Disability-Adjusted Life Years) | Nigeria | Female | Age-standardized | Low back pain | Rate | 2021 | 366.409674 | 506.357889 | 250.167016 |
| Prevalence | Nigeria | Female | Age-standardized | Low back pain | Rate | 2021 | 3363.3503 | 4028.70725 | 2756.46191 |
| Incidence | Nigeria | Female | Age-standardized | Low back pain | Rate | 2021 | 1374.68859 | 1654.23669 | 1115.60395 |
| DALYs (Disability-Adjusted Life Years) | Niue | Female | Age-standardized | Low back pain | Rate | 2021 | 355.906178 | 478.83313 | 248.248777 |
| Prevalence | Niue | Female | Age-standardized | Low back pain | Rate | 2021 | 3324.77633 | 3926.68737 | 2754.98659 |
| Incidence | Niue | Female | Age-standardized | Low back pain | Rate | 2021 | 1336.51439 | 1596.08617 | 1097.18065 |
| DALYs (Disability-Adjusted Life Years) | North Macedonia | Female | Age-standardized | Low back pain | Rate | 2021 | 527.686054 | 719.677207 | 368.718432 |
| Prevalence | North Macedonia | Female | Age-standardized | Low back pain | Rate | 2021 | 4895.65626 | 5704.43957 | 4145.41368 |
| Incidence | North Macedonia | Female | Age-standardized | Low back pain | Rate | 2021 | 1835.00425 | 2168.66345 | 1508.3884 |
| DALYs (Disability-Adjusted Life Years) | Northern Mariana Islands | Female | Age-standardized | Low back pain | Rate | 2021 | 349.513487 | 495.398354 | 236.253387 |
| Prevalence | Northern Mariana Islands | Female | Age-standardized | Low back pain | Rate | 2021 | 3167.3819 | 3832.6147 | 2591.84629 |
| Incidence | Northern Mariana Islands | Female | Age-standardized | Low back pain | Rate | 2021 | 1278.22289 | 1550.88973 | 1032.30712 |
| DALYs (Disability-Adjusted Life Years) | Norway | Female | Age-standardized | Low back pain | Rate | 2021 | 436.927271 | 582.835682 | 304.557725 |
| Prevalence | Norway | Female | Age-standardized | Low back pain | Rate | 2021 | 4036.42307 | 4663.04408 | 3424.12935 |
| Incidence | Norway | Female | Age-standardized | Low back pain | Rate | 2021 | 1653.15419 | 1931.68 | 1396.25801 |
| DALYs (Disability-Adjusted Life Years) | Oman | Female | Age-standardized | Low back pain | Rate | 2021 | 352.699663 | 487.091603 | 240.793424 |
| Prevalence | Oman | Female | Age-standardized | Low back pain | Rate | 2021 | 3266.04558 | 3903.1516 | 2673.07079 |
| Incidence | Oman | Female | Age-standardized | Low back pain | Rate | 2021 | 1335.79273 | 1607.28137 | 1071.14989 |
| DALYs (Disability-Adjusted Life Years) | Pakistan | Female | Age-standardized | Low back pain | Rate | 2021 | 453.002465 | 628.354041 | 305.467274 |
| Prevalence | Pakistan | Female | Age-standardized | Low back pain | Rate | 2021 | 4216.06893 | 5054.31624 | 3373.48548 |
| Incidence | Pakistan | Female | Age-standardized | Low back pain | Rate | 2021 | 1657.88648 | 2014.35345 | 1341.58641 |
| DALYs (Disability-Adjusted Life Years) | Palau | Female | Age-standardized | Low back pain | Rate | 2021 | 345.453507 | 478.934964 | 231.555538 |
| Prevalence | Palau | Female | Age-standardized | Low back pain | Rate | 2021 | 3187.3156 | 3912.15444 | 2565.10442 |
| Incidence | Palau | Female | Age-standardized | Low back pain | Rate | 2021 | 1297.94586 | 1565.92711 | 1046.65883 |
| DALYs (Disability-Adjusted Life Years) | Palestine | Female | Age-standardized | Low back pain | Rate | 2021 | 353.72328 | 491.298225 | 244.029114 |
| Prevalence | Palestine | Female | Age-standardized | Low back pain | Rate | 2021 | 3292.86326 | 3886.16967 | 2724.83743 |
| Incidence | Palestine | Female | Age-standardized | Low back pain | Rate | 2021 | 1340.19081 | 1598.95272 | 1088.31777 |
| DALYs (Disability-Adjusted Life Years) | Panama | Female | Age-standardized | Low back pain | Rate | 2021 | 344.231509 | 468.873206 | 238.745941 |
| Prevalence | Panama | Female | Age-standardized | Low back pain | Rate | 2021 | 3171.53497 | 3792.26043 | 2636.94895 |
| Incidence | Panama | Female | Age-standardized | Low back pain | Rate | 2021 | 1261.43197 | 1509.77374 | 1025.94029 |
| DALYs (Disability-Adjusted Life Years) | Papua New Guinea | Female | Age-standardized | Low back pain | Rate | 2021 | 344.908616 | 478.608316 | 232.223678 |
| Prevalence | Papua New Guinea | Female | Age-standardized | Low back pain | Rate | 2021 | 3153.69292 | 3829.94858 | 2544.1059 |
| Incidence | Papua New Guinea | Female | Age-standardized | Low back pain | Rate | 2021 | 1286.47571 | 1581.25065 | 1036.11613 |
| DALYs (Disability-Adjusted Life Years) | Paraguay | Female | Age-standardized | Low back pain | Rate | 2021 | 355.75713 | 487.66043 | 246.160378 |
| Prevalence | Paraguay | Female | Age-standardized | Low back pain | Rate | 2021 | 3287.18045 | 3938.48592 | 2722.70813 |
| Incidence | Paraguay | Female | Age-standardized | Low back pain | Rate | 2021 | 1296.32577 | 1562.05104 | 1069.28387 |
| DALYs (Disability-Adjusted Life Years) | Peru | Female | Age-standardized | Low back pain | Rate | 2021 | 283.217424 | 382.807842 | 192.720674 |
| Prevalence | Peru | Female | Age-standardized | Low back pain | Rate | 2021 | 2594.21582 | 3081.04634 | 2149.49746 |
| Incidence | Peru | Female | Age-standardized | Low back pain | Rate | 2021 | 1073.24736 | 1275.09756 | 884.459837 |
| DALYs (Disability-Adjusted Life Years) | Philippines | Female | Age-standardized | Low back pain | Rate | 2021 | 358.607888 | 490.912241 | 244.680423 |
| Prevalence | Philippines | Female | Age-standardized | Low back pain | Rate | 2021 | 3300.01649 | 3928.13278 | 2752.88552 |
| Incidence | Philippines | Female | Age-standardized | Low back pain | Rate | 2021 | 1318.96174 | 1565.80489 | 1083.04818 |
| DALYs (Disability-Adjusted Life Years) | Poland | Female | Age-standardized | Low back pain | Rate | 2021 | 584.902837 | 789.264339 | 411.477165 |
| Prevalence | Poland | Female | Age-standardized | Low back pain | Rate | 2021 | 5449.2414 | 6299.86423 | 4669.01308 |
| Incidence | Poland | Female | Age-standardized | Low back pain | Rate | 2021 | 2035.18753 | 2382.42892 | 1688.20017 |
| DALYs (Disability-Adjusted Life Years) | Portugal | Female | Age-standardized | Low back pain | Rate | 2021 | 500.206692 | 672.534698 | 351.430714 |
| Prevalence | Portugal | Female | Age-standardized | Low back pain | Rate | 2021 | 4643.36671 | 5297.84772 | 4025.00617 |
| Incidence | Portugal | Female | Age-standardized | Low back pain | Rate | 2021 | 1803.82021 | 2098.91681 | 1519.40199 |
| DALYs (Disability-Adjusted Life Years) | Puerto Rico | Female | Age-standardized | Low back pain | Rate | 2021 | 300.896345 | 403.380903 | 207.177111 |
| Prevalence | Puerto Rico | Female | Age-standardized | Low back pain | Rate | 2021 | 2792.91043 | 3325.05246 | 2336.55203 |
| Incidence | Puerto Rico | Female | Age-standardized | Low back pain | Rate | 2021 | 1134.68731 | 1345.84979 | 957.524234 |
| DALYs (Disability-Adjusted Life Years) | Qatar | Female | Age-standardized | Low back pain | Rate | 2021 | 343.047042 | 476.020111 | 234.614698 |
| Prevalence | Qatar | Female | Age-standardized | Low back pain | Rate | 2021 | 3209.82656 | 3920.45455 | 2589.27145 |
| Incidence | Qatar | Female | Age-standardized | Low back pain | Rate | 2021 | 1302.03879 | 1585.37573 | 1040.20618 |
| DALYs (Disability-Adjusted Life Years) | Republic of Korea | Female | Age-standardized | Low back pain | Rate | 2021 | 401.359467 | 543.42321 | 274.83281 |
| Prevalence | Republic of Korea | Female | Age-standardized | Low back pain | Rate | 2021 | 3667.49818 | 4314.59055 | 3067.33026 |
| Incidence | Republic of Korea | Female | Age-standardized | Low back pain | Rate | 2021 | 1490.39918 | 1757.58736 | 1225.35907 |
| DALYs (Disability-Adjusted Life Years) | Republic of Moldova | Female | Age-standardized | Low back pain | Rate | 2021 | 552.386358 | 761.659571 | 380.832156 |
| Prevalence | Republic of Moldova | Female | Age-standardized | Low back pain | Rate | 2021 | 5117.89922 | 5946.84903 | 4361.81152 |
| Incidence | Republic of Moldova | Female | Age-standardized | Low back pain | Rate | 2021 | 1845.09423 | 2192.02026 | 1537.02527 |
| DALYs (Disability-Adjusted Life Years) | Romania | Female | Age-standardized | Low back pain | Rate | 2021 | 596.760832 | 809.171241 | 417.762165 |
| Prevalence | Romania | Female | Age-standardized | Low back pain | Rate | 2021 | 5510.23116 | 6347.02215 | 4701.41682 |
| Incidence | Romania | Female | Age-standardized | Low back pain | Rate | 2021 | 1940.77585 | 2263.721 | 1607.44873 |
| DALYs (Disability-Adjusted Life Years) | Russian Federation | Female | Age-standardized | Low back pain | Rate | 2021 | 584.710277 | 792.791844 | 410.349293 |
| Prevalence | Russian Federation | Female | Age-standardized | Low back pain | Rate | 2021 | 5465.49776 | 6325.96606 | 4685.32292 |
| Incidence | Russian Federation | Female | Age-standardized | Low back pain | Rate | 2021 | 2033.91299 | 2373.15983 | 1690.93548 |
| DALYs (Disability-Adjusted Life Years) | Rwanda | Female | Age-standardized | Low back pain | Rate | 2021 | 435.648825 | 596.804273 | 297.091006 |
| Prevalence | Rwanda | Female | Age-standardized | Low back pain | Rate | 2021 | 3972.65536 | 4742.83344 | 3281.00552 |
| Incidence | Rwanda | Female | Age-standardized | Low back pain | Rate | 2021 | 1535.04965 | 1837.72207 | 1248.15332 |
| DALYs (Disability-Adjusted Life Years) | Saint Kitts and Nevis | Female | Age-standardized | Low back pain | Rate | 2021 | 292.689832 | 402.661695 | 193.442571 |
| Prevalence | Saint Kitts and Nevis | Female | Age-standardized | Low back pain | Rate | 2021 | 2671.96254 | 3206.90151 | 2153.81956 |
| Incidence | Saint Kitts and Nevis | Female | Age-standardized | Low back pain | Rate | 2021 | 1101.17232 | 1323.11498 | 887.533549 |
| DALYs (Disability-Adjusted Life Years) | Saint Lucia | Female | Age-standardized | Low back pain | Rate | 2021 | 303.62173 | 407.784819 | 209.863796 |
| Prevalence | Saint Lucia | Female | Age-standardized | Low back pain | Rate | 2021 | 2800.43344 | 3348.87169 | 2291.25739 |
| Incidence | Saint Lucia | Female | Age-standardized | Low back pain | Rate | 2021 | 1136.47846 | 1359.81828 | 933.404296 |
| DALYs (Disability-Adjusted Life Years) | Saint Vincent and the Grenadines | Female | Age-standardized | Low back pain | Rate | 2021 | 297.679478 | 402.695499 | 206.090396 |
| Prevalence | Saint Vincent and the Grenadines | Female | Age-standardized | Low back pain | Rate | 2021 | 2740.25032 | 3290.79779 | 2238.31111 |
| Incidence | Saint Vincent and the Grenadines | Female | Age-standardized | Low back pain | Rate | 2021 | 1121.0613 | 1334.86673 | 920.265611 |
| DALYs (Disability-Adjusted Life Years) | Samoa | Female | Age-standardized | Low back pain | Rate | 2021 | 362.732663 | 497.771073 | 248.41313 |
| Prevalence | Samoa | Female | Age-standardized | Low back pain | Rate | 2021 | 3360.97502 | 3976.13669 | 2758.37759 |
| Incidence | Samoa | Female | Age-standardized | Low back pain | Rate | 2021 | 1349.06988 | 1611.37079 | 1110.78567 |
| DALYs (Disability-Adjusted Life Years) | San Marino | Female | Age-standardized | Low back pain | Rate | 2021 | 441.824977 | 592.873767 | 306.192146 |
| Prevalence | San Marino | Female | Age-standardized | Low back pain | Rate | 2021 | 4078.49961 | 4724.46405 | 3496.69074 |
| Incidence | San Marino | Female | Age-standardized | Low back pain | Rate | 2021 | 1656.6662 | 1941.61411 | 1400.62863 |
| DALYs (Disability-Adjusted Life Years) | Sao Tome and Principe | Female | Age-standardized | Low back pain | Rate | 2021 | 316.05562 | 439.423878 | 214.052878 |
| Prevalence | Sao Tome and Principe | Female | Age-standardized | Low back pain | Rate | 2021 | 2899.55338 | 3487.15576 | 2358.51424 |
| Incidence | Sao Tome and Principe | Female | Age-standardized | Low back pain | Rate | 2021 | 1224.59476 | 1484.89881 | 1001.10276 |
| DALYs (Disability-Adjusted Life Years) | Saudi Arabia | Female | Age-standardized | Low back pain | Rate | 2021 | 343.12509 | 478.027858 | 229.507518 |
| Prevalence | Saudi Arabia | Female | Age-standardized | Low back pain | Rate | 2021 | 3165.45097 | 3827.14897 | 2515.48848 |
| Incidence | Saudi Arabia | Female | Age-standardized | Low back pain | Rate | 2021 | 1290.06506 | 1594.9395 | 1024.38156 |
| DALYs (Disability-Adjusted Life Years) | Senegal | Female | Age-standardized | Low back pain | Rate | 2021 | 325.707962 | 445.579138 | 220.93053 |
| Prevalence | Senegal | Female | Age-standardized | Low back pain | Rate | 2021 | 3008.20074 | 3636.89095 | 2459.39203 |
| Incidence | Senegal | Female | Age-standardized | Low back pain | Rate | 2021 | 1255.80625 | 1492.10545 | 1026.54066 |
| DALYs (Disability-Adjusted Life Years) | Serbia | Female | Age-standardized | Low back pain | Rate | 2021 | 588.350962 | 795.090061 | 406.804286 |
| Prevalence | Serbia | Female | Age-standardized | Low back pain | Rate | 2021 | 5464.36606 | 6282.99305 | 4664.15523 |
| Incidence | Serbia | Female | Age-standardized | Low back pain | Rate | 2021 | 1922.30715 | 2277.75525 | 1619.27855 |
| DALYs (Disability-Adjusted Life Years) | Seychelles | Female | Age-standardized | Low back pain | Rate | 2021 | 300.486436 | 411.183274 | 203.587046 |
| Prevalence | Seychelles | Female | Age-standardized | Low back pain | Rate | 2021 | 2786.13211 | 3275.66696 | 2309.63427 |
| Incidence | Seychelles | Female | Age-standardized | Low back pain | Rate | 2021 | 1148.65291 | 1378.33712 | 944.930331 |
| DALYs (Disability-Adjusted Life Years) | Sierra Leone | Female | Age-standardized | Low back pain | Rate | 2021 | 352.998269 | 486.57085 | 243.28116 |
| Prevalence | Sierra Leone | Female | Age-standardized | Low back pain | Rate | 2021 | 3256.12246 | 3960.75796 | 2674.42835 |
| Incidence | Sierra Leone | Female | Age-standardized | Low back pain | Rate | 2021 | 1326.98979 | 1580.08947 | 1082.59361 |
| DALYs (Disability-Adjusted Life Years) | Singapore | Female | Age-standardized | Low back pain | Rate | 2021 | 341.561918 | 464.175503 | 230.275238 |
| Prevalence | Singapore | Female | Age-standardized | Low back pain | Rate | 2021 | 3068.1699 | 3636.24901 | 2555.41121 |
| Incidence | Singapore | Female | Age-standardized | Low back pain | Rate | 2021 | 1312.78094 | 1539.3428 | 1088.4719 |
| DALYs (Disability-Adjusted Life Years) | Slovakia | Female | Age-standardized | Low back pain | Rate | 2021 | 592.531581 | 799.280798 | 412.826505 |
| Prevalence | Slovakia | Female | Age-standardized | Low back pain | Rate | 2021 | 5499.59773 | 6435.50915 | 4649.69236 |
| Incidence | Slovakia | Female | Age-standardized | Low back pain | Rate | 2021 | 1925.99491 | 2265.77742 | 1598.53593 |
| DALYs (Disability-Adjusted Life Years) | Slovenia | Female | Age-standardized | Low back pain | Rate | 2021 | 540.85349 | 717.438769 | 375.476897 |
| Prevalence | Slovenia | Female | Age-standardized | Low back pain | Rate | 2021 | 5066.93405 | 5856.03641 | 4366.62177 |
| Incidence | Slovenia | Female | Age-standardized | Low back pain | Rate | 2021 | 1849.24091 | 2171.46868 | 1536.32739 |
| DALYs (Disability-Adjusted Life Years) | Solomon Islands | Female | Age-standardized | Low back pain | Rate | 2021 | 366.597108 | 500.013867 | 250.361219 |
| Prevalence | Solomon Islands | Female | Age-standardized | Low back pain | Rate | 2021 | 3372.76522 | 4062.7359 | 2776.59131 |
| Incidence | Solomon Islands | Female | Age-standardized | Low back pain | Rate | 2021 | 1349.72704 | 1616.07183 | 1091.71943 |
| DALYs (Disability-Adjusted Life Years) | Somalia | Female | Age-standardized | Low back pain | Rate | 2021 | 411.901235 | 582.940023 | 274.386839 |
| Prevalence | Somalia | Female | Age-standardized | Low back pain | Rate | 2021 | 3764.98171 | 4537.72086 | 3048.85068 |
| Incidence | Somalia | Female | Age-standardized | Low back pain | Rate | 2021 | 1492.7935 | 1809.14594 | 1199.39639 |
| DALYs (Disability-Adjusted Life Years) | South Africa | Female | Age-standardized | Low back pain | Rate | 2021 | 353.219375 | 475.394291 | 240.442714 |
| Prevalence | South Africa | Female | Age-standardized | Low back pain | Rate | 2021 | 3303.81793 | 3916.95045 | 2712.27001 |
| Incidence | South Africa | Female | Age-standardized | Low back pain | Rate | 2021 | 1350.86682 | 1607.11948 | 1111.27098 |
| DALYs (Disability-Adjusted Life Years) | South Sudan | Female | Age-standardized | Low back pain | Rate | 2021 | 389.738681 | 537.519252 | 260.086857 |
| Prevalence | South Sudan | Female | Age-standardized | Low back pain | Rate | 2021 | 3633.70019 | 4410.09573 | 2963.64677 |
| Incidence | South Sudan | Female | Age-standardized | Low back pain | Rate | 2021 | 1457.91751 | 1757.11532 | 1189.88632 |
| DALYs (Disability-Adjusted Life Years) | Spain | Female | Age-standardized | Low back pain | Rate | 2021 | 387.72604 | 527.153794 | 267.728104 |
| Prevalence | Spain | Female | Age-standardized | Low back pain | Rate | 2021 | 3599.72413 | 4143.40225 | 3008.74455 |
| Incidence | Spain | Female | Age-standardized | Low back pain | Rate | 2021 | 1599.94974 | 1888.51114 | 1302.71186 |
| DALYs (Disability-Adjusted Life Years) | Sri Lanka | Female | Age-standardized | Low back pain | Rate | 2021 | 311.154527 | 424.973201 | 213.061328 |
| Prevalence | Sri Lanka | Female | Age-standardized | Low back pain | Rate | 2021 | 2899.43131 | 3456.44518 | 2422.78771 |
| Incidence | Sri Lanka | Female | Age-standardized | Low back pain | Rate | 2021 | 1195.73425 | 1425.42988 | 976.701732 |
| DALYs (Disability-Adjusted Life Years) | Sudan | Female | Age-standardized | Low back pain | Rate | 2021 | 365.348634 | 499.414565 | 251.369624 |
| Prevalence | Sudan | Female | Age-standardized | Low back pain | Rate | 2021 | 3368.61317 | 3965.36267 | 2763.41878 |
| Incidence | Sudan | Female | Age-standardized | Low back pain | Rate | 2021 | 1357.94667 | 1614.34112 | 1117.57442 |
| DALYs (Disability-Adjusted Life Years) | Suriname | Female | Age-standardized | Low back pain | Rate | 2021 | 301.311305 | 413.019723 | 204.367089 |
| Prevalence | Suriname | Female | Age-standardized | Low back pain | Rate | 2021 | 2780.59629 | 3341.81047 | 2284.55511 |
| Incidence | Suriname | Female | Age-standardized | Low back pain | Rate | 2021 | 1129.50321 | 1354.09677 | 924.314773 |
| DALYs (Disability-Adjusted Life Years) | Sweden | Female | Age-standardized | Low back pain | Rate | 2021 | 374.569231 | 506.08743 | 254.708667 |
| Prevalence | Sweden | Female | Age-standardized | Low back pain | Rate | 2021 | 3475.02804 | 4114.54653 | 2883.42672 |
| Incidence | Sweden | Female | Age-standardized | Low back pain | Rate | 2021 | 1478.45069 | 1738.13553 | 1223.65664 |
| DALYs (Disability-Adjusted Life Years) | Switzerland | Female | Age-standardized | Low back pain | Rate | 2021 | 473.507761 | 636.544164 | 331.761136 |
| Prevalence | Switzerland | Female | Age-standardized | Low back pain | Rate | 2021 | 4398.98885 | 5042.82208 | 3709.9617 |
| Incidence | Switzerland | Female | Age-standardized | Low back pain | Rate | 2021 | 1731.07807 | 2021.57557 | 1458.96156 |
| DALYs (Disability-Adjusted Life Years) | Syrian Arab Republic | Female | Age-standardized | Low back pain | Rate | 2021 | 362.129504 | 499.521647 | 249.626172 |
| Prevalence | Syrian Arab Republic | Female | Age-standardized | Low back pain | Rate | 2021 | 3343.22231 | 3952.80817 | 2763.08157 |
| Incidence | Syrian Arab Republic | Female | Age-standardized | Low back pain | Rate | 2021 | 1358.50746 | 1635.38952 | 1118.02842 |
| DALYs (Disability-Adjusted Life Years) | Taiwan (Province of China) | Female | Age-standardized | Low back pain | Rate | 2021 | 443.885988 | 575.286639 | 326.991883 |
| Prevalence | Taiwan (Province of China) | Female | Age-standardized | Low back pain | Rate | 2021 | 4066.18452 | 4170.15369 | 3959.82623 |
| Incidence | Taiwan (Province of China) | Female | Age-standardized | Low back pain | Rate | 2021 | 1530.16606 | 1772.47387 | 1315.83057 |
| DALYs (Disability-Adjusted Life Years) | Tajikistan | Female | Age-standardized | Low back pain | Rate | 2021 | 424.213334 | 603.481772 | 284.079345 |
| Prevalence | Tajikistan | Female | Age-standardized | Low back pain | Rate | 2021 | 3842.22772 | 4573.01284 | 3131.93529 |
| Incidence | Tajikistan | Female | Age-standardized | Low back pain | Rate | 2021 | 1534.37424 | 1866.82985 | 1224.5332 |
| DALYs (Disability-Adjusted Life Years) | Thailand | Female | Age-standardized | Low back pain | Rate | 2021 | 293.350758 | 405.468426 | 200.195021 |
| Prevalence | Thailand | Female | Age-standardized | Low back pain | Rate | 2021 | 2697.35699 | 3221.68961 | 2239.12702 |
| Incidence | Thailand | Female | Age-standardized | Low back pain | Rate | 2021 | 1125.4508 | 1329.81549 | 925.156863 |
| DALYs (Disability-Adjusted Life Years) | Timor-Leste | Female | Age-standardized | Low back pain | Rate | 2021 | 305.139547 | 415.131583 | 209.862823 |
| Prevalence | Timor-Leste | Female | Age-standardized | Low back pain | Rate | 2021 | 2816.50027 | 3292.55902 | 2339.33875 |
| Incidence | Timor-Leste | Female | Age-standardized | Low back pain | Rate | 2021 | 1175.60686 | 1396.38928 | 966.126795 |
| DALYs (Disability-Adjusted Life Years) | Togo | Female | Age-standardized | Low back pain | Rate | 2021 | 353.640963 | 487.017863 | 242.846697 |
| Prevalence | Togo | Female | Age-standardized | Low back pain | Rate | 2021 | 3230.51476 | 3874.17691 | 2635.23471 |
| Incidence | Togo | Female | Age-standardized | Low back pain | Rate | 2021 | 1322.94918 | 1592.35906 | 1075.24079 |
| DALYs (Disability-Adjusted Life Years) | Tokelau | Female | Age-standardized | Low back pain | Rate | 2021 | 362.295498 | 486.725154 | 252.335752 |
| Prevalence | Tokelau | Female | Age-standardized | Low back pain | Rate | 2021 | 3386.92928 | 3997.67805 | 2811.98581 |
| Incidence | Tokelau | Female | Age-standardized | Low back pain | Rate | 2021 | 1364.41761 | 1625.80502 | 1132.30036 |
| DALYs (Disability-Adjusted Life Years) | Tonga | Female | Age-standardized | Low back pain | Rate | 2021 | 366.41843 | 499.565096 | 249.89204 |
| Prevalence | Tonga | Female | Age-standardized | Low back pain | Rate | 2021 | 3404.0495 | 4027.61336 | 2814.37305 |
| Incidence | Tonga | Female | Age-standardized | Low back pain | Rate | 2021 | 1357.79232 | 1592.54621 | 1117.07437 |
| DALYs (Disability-Adjusted Life Years) | Trinidad and Tobago | Female | Age-standardized | Low back pain | Rate | 2021 | 303.36942 | 412.291738 | 209.434738 |
| Prevalence | Trinidad and Tobago | Female | Age-standardized | Low back pain | Rate | 2021 | 2804.69319 | 3348.31104 | 2318.58994 |
| Incidence | Trinidad and Tobago | Female | Age-standardized | Low back pain | Rate | 2021 | 1141.12814 | 1351.89612 | 953.072107 |
| DALYs (Disability-Adjusted Life Years) | Tunisia | Female | Age-standardized | Low back pain | Rate | 2021 | 384.067868 | 518.941515 | 269.925386 |
| Prevalence | Tunisia | Female | Age-standardized | Low back pain | Rate | 2021 | 3585.32218 | 4224.28254 | 2983.25 |
| Incidence | Tunisia | Female | Age-standardized | Low back pain | Rate | 2021 | 1421.12939 | 1690.53474 | 1163.67182 |
| DALYs (Disability-Adjusted Life Years) | Turkmenistan | Female | Age-standardized | Low back pain | Rate | 2021 | 443.810652 | 611.994929 | 305.441214 |
| Prevalence | Turkmenistan | Female | Age-standardized | Low back pain | Rate | 2021 | 4034.0551 | 4781.79594 | 3331.5681 |
| Incidence | Turkmenistan | Female | Age-standardized | Low back pain | Rate | 2021 | 1584.56836 | 1882.7828 | 1296.35159 |
| DALYs (Disability-Adjusted Life Years) | Tuvalu | Female | Age-standardized | Low back pain | Rate | 2021 | 360.69537 | 491.785311 | 243.056682 |
| Prevalence | Tuvalu | Female | Age-standardized | Low back pain | Rate | 2021 | 3328.7003 | 3995.54715 | 2742.66614 |
| Incidence | Tuvalu | Female | Age-standardized | Low back pain | Rate | 2021 | 1349.12303 | 1612.89364 | 1100.20388 |
| DALYs (Disability-Adjusted Life Years) | T眉rkiye | Female | Age-standardized | Low back pain | Rate | 2021 | 365.947006 | 497.33257 | 253.719284 |
| Prevalence | T眉rkiye | Female | Age-standardized | Low back pain | Rate | 2021 | 3395.05317 | 4020.57816 | 2815.93184 |
| Incidence | T眉rkiye | Female | Age-standardized | Low back pain | Rate | 2021 | 1375.74012 | 1630.42875 | 1130.31548 |
| DALYs (Disability-Adjusted Life Years) | Uganda | Female | Age-standardized | Low back pain | Rate | 2021 | 417.479062 | 582.746401 | 284.194117 |
| Prevalence | Uganda | Female | Age-standardized | Low back pain | Rate | 2021 | 3822.97504 | 4570.26462 | 3136.6957 |
| Incidence | Uganda | Female | Age-standardized | Low back pain | Rate | 2021 | 1499.08294 | 1815.69191 | 1219.61894 |
| DALYs (Disability-Adjusted Life Years) | Ukraine | Female | Age-standardized | Low back pain | Rate | 2021 | 654.701042 | 904.609424 | 462.564683 |
| Prevalence | Ukraine | Female | Age-standardized | Low back pain | Rate | 2021 | 6094.50994 | 7004.99226 | 5243.75591 |
| Incidence | Ukraine | Female | Age-standardized | Low back pain | Rate | 2021 | 2140.09173 | 2514.23702 | 1777.69155 |
| DALYs (Disability-Adjusted Life Years) | United Arab Emirates | Female | Age-standardized | Low back pain | Rate | 2021 | 314.249977 | 447.826577 | 210.082698 |
| Prevalence | United Arab Emirates | Female | Age-standardized | Low back pain | Rate | 2021 | 2876.87105 | 3656.75173 | 2263.5254 |
| Incidence | United Arab Emirates | Female | Age-standardized | Low back pain | Rate | 2021 | 1203.84493 | 1493.9203 | 936.518321 |
| DALYs (Disability-Adjusted Life Years) | United Kingdom | Female | Age-standardized | Low back pain | Rate | 2021 | 464.710312 | 616.366553 | 324.015302 |
| Prevalence | United Kingdom | Female | Age-standardized | Low back pain | Rate | 2021 | 4310.05699 | 4976.20175 | 3660.41214 |
| Incidence | United Kingdom | Female | Age-standardized | Low back pain | Rate | 2021 | 1738.17636 | 2017.62987 | 1455.2755 |
| DALYs (Disability-Adjusted Life Years) | United Republic of Tanzania | Female | Age-standardized | Low back pain | Rate | 2021 | 408.714758 | 561.476756 | 278.772925 |
| Prevalence | United Republic of Tanzania | Female | Age-standardized | Low back pain | Rate | 2021 | 3761.72408 | 4488.60668 | 3118.33892 |
| Incidence | United Republic of Tanzania | Female | Age-standardized | Low back pain | Rate | 2021 | 1494.86061 | 1791.19864 | 1222.49838 |
| DALYs (Disability-Adjusted Life Years) | United States of America | Female | Age-standardized | Low back pain | Rate | 2021 | 419.662521 | 544.958059 | 299.825139 |
| Prevalence | United States of America | Female | Age-standardized | Low back pain | Rate | 2021 | 3965.76854 | 4334.42891 | 3625.60506 |
| Incidence | United States of America | Female | Age-standardized | Low back pain | Rate | 2021 | 1603.57576 | 1797.97868 | 1410.54694 |
| DALYs (Disability-Adjusted Life Years) | United States Virgin Islands | Female | Age-standardized | Low back pain | Rate | 2021 | 304.687552 | 411.294504 | 209.129854 |
| Prevalence | United States Virgin Islands | Female | Age-standardized | Low back pain | Rate | 2021 | 2820.9819 | 3327.33037 | 2348.35595 |
| Incidence | United States Virgin Islands | Female | Age-standardized | Low back pain | Rate | 2021 | 1144.65507 | 1366.66684 | 944.856534 |
| DALYs (Disability-Adjusted Life Years) | Uruguay | Female | Age-standardized | Low back pain | Rate | 2021 | 484.611562 | 652.466198 | 335.194432 |
| Prevalence | Uruguay | Female | Age-standardized | Low back pain | Rate | 2021 | 4492.00969 | 5245.90321 | 3834.08139 |
| Incidence | Uruguay | Female | Age-standardized | Low back pain | Rate | 2021 | 1768.11237 | 2075.7177 | 1486.45061 |
| DALYs (Disability-Adjusted Life Years) | Uzbekistan | Female | Age-standardized | Low back pain | Rate | 2021 | 449.79354 | 628.925546 | 307.566908 |
| Prevalence | Uzbekistan | Female | Age-standardized | Low back pain | Rate | 2021 | 4109.15443 | 4957.7944 | 3386.40143 |
| Incidence | Uzbekistan | Female | Age-standardized | Low back pain | Rate | 2021 | 1607.14133 | 1914.57704 | 1328.38371 |
| DALYs (Disability-Adjusted Life Years) | Vanuatu | Female | Age-standardized | Low back pain | Rate | 2021 | 384.89546 | 533.831974 | 264.910547 |
| Prevalence | Vanuatu | Female | Age-standardized | Low back pain | Rate | 2021 | 3518.92703 | 4193.8594 | 2869.44153 |
| Incidence | Vanuatu | Female | Age-standardized | Low back pain | Rate | 2021 | 1383.26013 | 1670.06075 | 1120.27941 |
| DALYs (Disability-Adjusted Life Years) | Venezuela (Bolivarian Republic of) | Female | Age-standardized | Low back pain | Rate | 2021 | 316.115627 | 431.495685 | 216.534809 |
| Prevalence | Venezuela (Bolivarian Republic of) | Female | Age-standardized | Low back pain | Rate | 2021 | 2899.67695 | 3466.87337 | 2392.06073 |
| Incidence | Venezuela (Bolivarian Republic of) | Female | Age-standardized | Low back pain | Rate | 2021 | 1184.61237 | 1419.5898 | 971.711171 |
| DALYs (Disability-Adjusted Life Years) | Viet Nam | Female | Age-standardized | Low back pain | Rate | 2021 | 331.472298 | 455.060507 | 228.202134 |
| Prevalence | Viet Nam | Female | Age-standardized | Low back pain | Rate | 2021 | 3013.3863 | 3603.79343 | 2497.74796 |
| Incidence | Viet Nam | Female | Age-standardized | Low back pain | Rate | 2021 | 1208.47761 | 1434.05379 | 999.891954 |
| DALYs (Disability-Adjusted Life Years) | Yemen | Female | Age-standardized | Low back pain | Rate | 2021 | 361.319063 | 495.399448 | 246.095741 |
| Prevalence | Yemen | Female | Age-standardized | Low back pain | Rate | 2021 | 3355.40314 | 3971.6331 | 2772.25411 |
| Incidence | Yemen | Female | Age-standardized | Low back pain | Rate | 2021 | 1362.14564 | 1629.79938 | 1122.98244 |
| DALYs (Disability-Adjusted Life Years) | Zambia | Female | Age-standardized | Low back pain | Rate | 2021 | 376.60181 | 513.635985 | 257.222184 |
| Prevalence | Zambia | Female | Age-standardized | Low back pain | Rate | 2021 | 3469.40971 | 4157.42106 | 2830.38043 |
| Incidence | Zambia | Female | Age-standardized | Low back pain | Rate | 2021 | 1422.53792 | 1710.13079 | 1145.84669 |
| DALYs (Disability-Adjusted Life Years) | Zimbabwe | Female | Age-standardized | Low back pain | Rate | 2021 | 396.352428 | 556.768432 | 266.10621 |
| Prevalence | Zimbabwe | Female | Age-standardized | Low back pain | Rate | 2021 | 3632.86852 | 4413.15516 | 2949.86071 |
| Incidence | Zimbabwe | Female | Age-standardized | Low back pain | Rate | 2021 | 1429.37332 | 1732.88764 | 1150.6014 |

Appendix7: The EAPC of ASIR for postmenopausal women worldwide from 1990 to 2021

| **location** | **EAPC** | **LCI** | **UCI** | **EAPC_CI** |  |
| --- | --- | --- | --- | --- | --- |
| **China** | **-0.41** | **-0.49** | **-0.33** | **-0.41 (-0.49 to -0.33)** | |
| **Taiwan (Province of China)** | **0.42** | **0.34** | **0.5** | **0.42 (0.34 to 0.50)** | |
| **Malaysia** | **-0.02** | **-0.06** | **0.02** | **-0.02 (-0.06 to 0.02)** | |
| **Myanmar** | **-0.06** | **-0.09** | **-0.02** | **-0.06 (-0.09 to -0.02)** | |
| **Tonga** | **-0.01** | **-0.03** | **0.02** | **-0.01 (-0.03 to 0.02)** | |
| **Viet Nam** | **-0.11** | **-0.2** | **-0.03** | **-0.11 (-0.20 to -0.03)** | |
| **Fiji** | **-0.03** | **-0.05** | **-0.01** | **-0.03 (-0.05 to -0.01)** | |
| **Marshall Islands** | **-0.23** | **-0.26** | **-0.19** | **-0.23 (-0.26 to -0.19)** | |
| **Cambodia** | **-0.17** | **-0.19** | **-0.16** | **-0.17 (-0.19 to -0.16)** | |
| **Democratic People's Republic of Korea** | **-0.03** | **-0.07** | **0.01** | **-0.03 (-0.07 to 0.01)** | |
| **Timor-Leste** | **0.06** | **0.02** | **0.1** | **0.06 (0.02 to 0.10)** | |
| **Lao People's Democratic Republic** | **-0.13** | **-0.15** | **-0.12** | **-0.13 (-0.15 to -0.12)** | |
| **Sri Lanka** | **-0.05** | **-0.06** | **-0.04** | **-0.05 (-0.06 to -0.04)** | |
| **Maldives** | **0.09** | **0.03** | **0.16** | **0.09 (0.03 to 0.16)** | |
| **Philippines** | **-0.08** | **-0.11** | **-0.05** | **-0.08 (-0.11 to -0.05)** | |
| **Papua New Guinea** | **-0.05** | **-0.08** | **-0.02** | **-0.05 (-0.08 to -0.02)** | |
| **Kazakhstan** | **-0.07** | **-0.1** | **-0.04** | **-0.07 (-0.10 to -0.04)** | |
| **Solomon Islands** | **0.21** | **0.18** | **0.24** | **0.21 (0.18 to 0.24)** | |
| **Vanuatu** | **-0.01** | **-0.02** | **0.01** | **-0.01 (-0.02 to 0.01)** | |
| **Kiribati** | **0.02** | **0** | **0.05** | **0.02 (-0.00 to 0.05)** | |
| **Armenia** | **0.08** | **0.02** | **0.14** | **0.08 (0.02 to 0.14)** | |
| **Thailand** | **0.19** | **0.15** | **0.22** | **0.19 (0.15 to 0.22)** | |
| **Mongolia** | **-0.22** | **-0.24** | **-0.2** | **-0.22 (-0.24 to -0.20)** | |
| **Kyrgyzstan** | **-0.18** | **-0.23** | **-0.12** | **-0.18 (-0.23 to -0.12)** | |
| **Georgia** | **-0.07** | **-0.12** | **-0.02** | **-0.07 (-0.12 to -0.02)** | |
| **Albania** | **0.02** | **0.01** | **0.02** | **0.02 (0.01 to 0.02)** | |
| **Slovakia** | **-0.1** | **-0.12** | **-0.08** | **-0.10 (-0.12 to -0.08)** | |
| **North Macedonia** | **0.07** | **0.06** | **0.08** | **0.07 (0.06 to 0.08)** | |
| **Azerbaijan** | **-0.02** | **-0.08** | **0.03** | **-0.02 (-0.08 to 0.03)** | |
| **Belarus** | **0** | **-0.04** | **0.05** | **0.00 (-0.04 to 0.05)** | |
| **Tajikistan** | **-0.24** | **-0.3** | **-0.17** | **-0.24 (-0.30 to -0.17)** | |
| **Uzbekistan** | **-0.13** | **-0.17** | **-0.08** | **-0.13 (-0.17 to -0.08)** | |
| **Slovenia** | **-0.01** | **-0.03** | **0** | **-0.01 (-0.03 to 0.00)** | |
| **Turkmenistan** | **-0.15** | **-0.19** | **-0.11** | **-0.15 (-0.19 to -0.11)** | |
| **Bulgaria** | **0.03** | **0.01** | **0.05** | **0.03 (0.01 to 0.05)** | |
| **Micronesia (Federated States of)** | **-0.14** | **-0.15** | **-0.13** | **-0.14 (-0.15 to -0.13)** | |
| **Estonia** | **0.07** | **0.05** | **0.09** | **0.07 (0.05 to 0.09)** | |
| **Indonesia** | **-0.03** | **-0.07** | **0.01** | **-0.03 (-0.07 to 0.01)** | |
| **Singapore** | **-0.09** | **-0.14** | **-0.03** | **-0.09 (-0.14 to -0.03)** | |
| **Czechia** | **-0.04** | **-0.06** | **-0.01** | **-0.04 (-0.06 to -0.01)** | |
| **New Zealand** | **-0.06** | **-0.1** | **-0.02** | **-0.06 (-0.10 to -0.02)** | |
| **Samoa** | **-0.13** | **-0.16** | **-0.1** | **-0.13 (-0.16 to -0.10)** | |
| **Hungary** | **-0.02** | **-0.03** | **0** | **-0.02 (-0.03 to 0.00)** | |
| **Romania** | **-0.02** | **-0.03** | **0** | **-0.02 (-0.03 to -0.00)** | |
| **Australia** | **-0.04** | **-0.09** | **0.01** | **-0.04 (-0.09 to 0.01)** | |
| **Bosnia and Herzegovina** | **0.09** | **0.06** | **0.12** | **0.09 (0.06 to 0.12)** | |
| **Russian Federation** | **-0.03** | **-0.07** | **0.01** | **-0.03 (-0.07 to 0.01)** | |
| **Montenegro** | **0.07** | **0.05** | **0.08** | **0.07 (0.05 to 0.08)** | |
| **Republic of Moldova** | **-0.04** | **-0.07** | **0** | **-0.04 (-0.07 to -0.00)** | |
| **Croatia** | **0.26** | **0.15** | **0.37** | **0.26 (0.15 to 0.37)** | |
| **Republic of Korea** | **-0.06** | **-0.1** | **-0.01** | **-0.06 (-0.10 to -0.01)** | |
| **Cyprus** | **-0.05** | **-0.07** | **-0.02** | **-0.05 (-0.07 to -0.02)** | |
| **Finland** | **-0.06** | **-0.1** | **-0.02** | **-0.06 (-0.10 to -0.02)** | |
| **France** | **0.01** | **-0.04** | **0.06** | **0.01 (-0.04 to 0.06)** | |
| **Israel** | **-0.08** | **-0.12** | **-0.05** | **-0.08 (-0.12 to -0.05)** | |
| **Malta** | **0.05** | **0.01** | **0.09** | **0.05 (0.01 to 0.09)** | |
| **Poland** | **-0.12** | **-0.15** | **-0.1** | **-0.12 (-0.15 to -0.10)** | |
| **Ukraine** | **-0.05** | **-0.09** | **-0.01** | **-0.05 (-0.09 to -0.01)** | |
| **Bahamas** | **-0.03** | **-0.05** | **-0.02** | **-0.03 (-0.05 to -0.02)** | |
| **Serbia** | **0.07** | **0.05** | **0.09** | **0.07 (0.05 to 0.09)** | |
| **Cuba** | **0** | **-0.03** | **0.03** | **-0.00 (-0.03 to 0.03)** | |
| **Sweden** | **0.4** | **0.29** | **0.51** | **0.40 (0.29 to 0.51)** | |
| **Belize** | **-0.05** | **-0.06** | **-0.04** | **-0.05 (-0.06 to -0.04)** | |
| **Iceland** | **-0.16** | **-0.2** | **-0.12** | **-0.16 (-0.20 to -0.12)** | |
| **Guyana** | **-0.05** | **-0.07** | **-0.03** | **-0.05 (-0.07 to -0.03)** | |
| **Suriname** | **0.07** | **0.04** | **0.1** | **0.07 (0.04 to 0.10)** | |
| **Saint Lucia** | **-0.13** | **-0.14** | **-0.12** | **-0.13 (-0.14 to -0.12)** | |
| **Dominican Republic** | **0.07** | **0.06** | **0.08** | **0.07 (0.06 to 0.08)** | |
| **Colombia** | **-0.03** | **-0.04** | **-0.02** | **-0.03 (-0.04 to -0.02)** | |
| **Belgium** | **-0.05** | **-0.09** | **-0.02** | **-0.05 (-0.09 to -0.02)** | |
| **Lithuania** | **0.06** | **0.03** | **0.09** | **0.06 (0.03 to 0.09)** | |
| **Saint Vincent and the Grenadines** | **-0.07** | **-0.08** | **-0.05** | **-0.07 (-0.08 to -0.05)** | |
| **Jamaica** | **-0.08** | **-0.11** | **-0.06** | **-0.08 (-0.11 to -0.06)** | |
| **Antigua and Barbuda** | **-0.06** | **-0.07** | **-0.05** | **-0.06 (-0.07 to -0.05)** | |
| **Dominica** | **-0.07** | **-0.1** | **-0.04** | **-0.07 (-0.10 to -0.04)** | |
| **Grenada** | **-0.04** | **-0.06** | **-0.01** | **-0.04 (-0.06 to -0.01)** | |
| **Italy** | **-0.07** | **-0.1** | **-0.05** | **-0.07 (-0.10 to -0.05)** | |
| **Ecuador** | **-0.06** | **-0.12** | **0.01** | **-0.06 (-0.12 to 0.01)** | |
| **Brunei Darussalam** | **-0.15** | **-0.19** | **-0.11** | **-0.15 (-0.19 to -0.11)** | |
| **Norway** | **-0.2** | **-0.27** | **-0.13** | **-0.20 (-0.27 to -0.13)** | |
| **El Salvador** | **0.02** | **0.01** | **0.03** | **0.02 (0.01 to 0.03)** | |
| **Panama** | **-0.03** | **-0.04** | **-0.02** | **-0.03 (-0.04 to -0.02)** | |
| **Japan** | **-0.05** | **-0.11** | **0.01** | **-0.05 (-0.11 to 0.01)** | |
| **Ireland** | **-0.11** | **-0.14** | **-0.08** | **-0.11 (-0.14 to -0.08)** | |
| **Bolivia (Plurinational State of)** | **0.05** | **0.02** | **0.09** | **0.05 (0.02 to 0.09)** | |
| **Haiti** | **-0.06** | **-0.08** | **-0.04** | **-0.06 (-0.08 to -0.04)** | |
| **Latvia** | **0.06** | **0.05** | **0.07** | **0.06 (0.05 to 0.07)** | |
| **Germany** | **-0.04** | **-0.08** | **0** | **-0.04 (-0.08 to 0.00)** | |
| **Barbados** | **-0.08** | **-0.08** | **-0.07** | **-0.08 (-0.08 to -0.07)** | |
| **Paraguay** | **0.12** | **0.08** | **0.16** | **0.12 (0.08 to 0.16)** | |
| **Nicaragua** | **-0.04** | **-0.05** | **-0.04** | **-0.04 (-0.05 to -0.04)** | |
| **United States of America** | **0.03** | **-0.01** | **0.08** | **0.03 (-0.01 to 0.08)** | |
| **Trinidad and Tobago** | **-0.04** | **-0.05** | **-0.03** | **-0.04 (-0.05 to -0.03)** | |
| **Peru** | **0.15** | **0.12** | **0.18** | **0.15 (0.12 to 0.18)** | |
| **Honduras** | **-0.01** | **-0.02** | **0** | **-0.01 (-0.02 to 0.00)** | |
| **Guatemala** | **-0.04** | **-0.07** | **-0.02** | **-0.04 (-0.07 to -0.02)** | |
| **Costa Rica** | **-0.13** | **-0.14** | **-0.12** | **-0.13 (-0.14 to -0.12)** | |
| **United Kingdom** | **0.88** | **0.74** | **1.02** | **0.88 (0.74 to 1.02)** | |
| **Andorra** | **-0.1** | **-0.13** | **-0.07** | **-0.10 (-0.13 to -0.07)** | |
| **Venezuela (Bolivarian Republic of)** | **-0.16** | **-0.17** | **-0.16** | **-0.16 (-0.17 to -0.16)** | |
| **Denmark** | **-0.44** | **-0.56** | **-0.33** | **-0.44 (-0.56 to -0.33)** | |
| **Netherlands** | **0.15** | **0.02** | **0.28** | **0.15 (0.02 to 0.28)** | |
| **Switzerland** | **0.15** | **0.11** | **0.19** | **0.15 (0.11 to 0.19)** | |
| **Uruguay** | **0.18** | **0.12** | **0.23** | **0.18 (0.12 to 0.23)** | |
| **Austria** | **-0.04** | **-0.07** | **-0.01** | **-0.04 (-0.07 to -0.01)** | |
| **Mexico** | **-0.06** | **-0.16** | **0.03** | **-0.06 (-0.16 to 0.03)** | |
| **Brazil** | **0.13** | **0.09** | **0.18** | **0.13 (0.09 to 0.18)** | |
| **Canada** | **-0.19** | **-0.24** | **-0.14** | **-0.19 (-0.24 to -0.14)** | |
| **Yemen** | **-0.14** | **-0.16** | **-0.12** | **-0.14 (-0.16 to -0.12)** | |
| **Algeria** | **-0.03** | **-0.07** | **0.01** | **-0.03 (-0.07 to 0.01)** | |
| **Greece** | **0.07** | **0.03** | **0.11** | **0.07 (0.03 to 0.11)** | |
| **Luxembourg** | **-0.07** | **-0.12** | **-0.02** | **-0.07 (-0.12 to -0.02)** | |
| **Afghanistan** | **0.19** | **0.16** | **0.22** | **0.19 (0.16 to 0.22)** | |
| **Kuwait** | **-0.13** | **-0.21** | **-0.06** | **-0.13 (-0.21 to -0.06)** | |
| **Pakistan** | **0.12** | **0.1** | **0.15** | **0.12 (0.10 to 0.15)** | |
| **Argentina** | **0.04** | **-0.01** | **0.09** | **0.04 (-0.01 to 0.09)** | |
| **Chile** | **0.03** | **-0.02** | **0.08** | **0.03 (-0.02 to 0.08)** | |
| **Spain** | **0.19** | **0.11** | **0.27** | **0.19 (0.11 to 0.27)** | |
| **Portugal** | **0** | **-0.04** | **0.05** | **0.00 (-0.04 to 0.05)** | |
| **Equatorial Guinea** | **-0.13** | **-0.15** | **-0.11** | **-0.13 (-0.15 to -0.11)** | |
| **Zambia** | **0.11** | **0.05** | **0.16** | **0.11 (0.05 to 0.16)** | |
| **Botswana** | **-0.06** | **-0.08** | **-0.03** | **-0.06 (-0.08 to -0.03)** | |
| **United Arab Emirates** | **-0.36** | **-0.43** | **-0.29** | **-0.36 (-0.43 to -0.29)** | |
| **Malawi** | **-0.06** | **-0.09** | **-0.03** | **-0.06 (-0.09 to -0.03)** | |
| **Benin** | **-0.14** | **-0.18** | **-0.1** | **-0.14 (-0.18 to -0.10)** | |
| **Zimbabwe** | **0.07** | **0.06** | **0.08** | **0.07 (0.06 to 0.08)** | |
| **Madagascar** | **-0.25** | **-0.28** | **-0.21** | **-0.25 (-0.28 to -0.21)** | |
| **Somalia** | **0.01** | **-0.01** | **0.02** | **0.01 (-0.01 to 0.02)** | |
| **Uganda** | **-0.13** | **-0.14** | **-0.11** | **-0.13 (-0.14 to -0.11)** | |
| **T眉rkiye** | **0.13** | **0.09** | **0.16** | **0.13 (0.09 to 0.16)** | |
| **Bahrain** | **-0.1** | **-0.16** | **-0.04** | **-0.10 (-0.16 to -0.04)** | |
| **Egypt** | **0.05** | **0.02** | **0.08** | **0.05 (0.02 to 0.08)** | |
| **Burkina Faso** | **-0.15** | **-0.18** | **-0.12** | **-0.15 (-0.18 to -0.12)** | |
| **Saudi Arabia** | **-0.15** | **-0.18** | **-0.12** | **-0.15 (-0.18 to -0.12)** | |
| **South Africa** | **-0.26** | **-0.28** | **-0.24** | **-0.26 (-0.28 to -0.24)** | |
| **Iran (Islamic Republic of)** | **-0.12** | **-0.16** | **-0.09** | **-0.12 (-0.16 to -0.09)** | |
| **Tunisia** | **0** | **-0.05** | **0.04** | **-0.00 (-0.05 to 0.04)** | |
| **Central African Republic** | **-0.08** | **-0.1** | **-0.05** | **-0.08 (-0.10 to -0.05)** | |
| **Burundi** | **-0.18** | **-0.21** | **-0.16** | **-0.18 (-0.21 to -0.16)** | |
| **Jordan** | **0.06** | **0.04** | **0.08** | **0.06 (0.04 to 0.08)** | |
| **Morocco** | **-0.02** | **-0.09** | **0.05** | **-0.02 (-0.09 to 0.05)** | |
| **Nepal** | **-0.11** | **-0.14** | **-0.08** | **-0.11 (-0.14 to -0.08)** | |
| **Iraq** | **-0.08** | **-0.09** | **-0.07** | **-0.08 (-0.09 to -0.07)** | |
| **Oman** | **-0.09** | **-0.11** | **-0.07** | **-0.09 (-0.11 to -0.07)** | |
| **Bangladesh** | **-0.11** | **-0.15** | **-0.07** | **-0.11 (-0.15 to -0.07)** | |
| **Syrian Arab Republic** | **-0.03** | **-0.06** | **0** | **-0.03 (-0.06 to 0.00)** | |
| **American Samoa** | **-0.08** | **-0.1** | **-0.07** | **-0.08 (-0.10 to -0.07)** | |
| **Kenya** | **-0.15** | **-0.19** | **-0.1** | **-0.15 (-0.19 to -0.10)** | |
| **Palestine** | **-0.09** | **-0.13** | **-0.05** | **-0.09 (-0.13 to -0.05)** | |
| **United Republic of Tanzania** | **-0.06** | **-0.09** | **-0.04** | **-0.06 (-0.09 to -0.04)** | |
| **Mauritania** | **-0.09** | **-0.13** | **-0.05** | **-0.09 (-0.13 to -0.05)** | |
| **Seychelles** | **-0.23** | **-0.27** | **-0.19** | **-0.23 (-0.27 to -0.19)** | |
| **Gabon** | **-0.09** | **-0.12** | **-0.05** | **-0.09 (-0.12 to -0.05)** | |
| **Bermuda** | **-0.02** | **-0.03** | **-0.01** | **-0.02 (-0.03 to -0.01)** | |
| **Libya** | **-0.07** | **-0.11** | **-0.04** | **-0.07 (-0.11 to -0.04)** | |
| **Palau** | **-0.15** | **-0.17** | **-0.14** | **-0.15 (-0.17 to -0.14)** | |
| **India** | **-0.31** | **-0.4** | **-0.22** | **-0.31 (-0.40 to -0.22)** | |
| **Lebanon** | **0.17** | **0.14** | **0.2** | **0.17 (0.14 to 0.20)** | |
| **Qatar** | **-0.01** | **-0.03** | **0** | **-0.01 (-0.03 to 0.00)** | |
| **Angola** | **-0.16** | **-0.19** | **-0.13** | **-0.16 (-0.19 to -0.13)** | |
| **Congo** | **-0.07** | **-0.1** | **-0.04** | **-0.07 (-0.10 to -0.04)** | |
| **Democratic Republic of the Congo** | **-0.11** | **-0.15** | **-0.07** | **-0.11 (-0.15 to -0.07)** | |
| **Djibouti** | **-0.14** | **-0.15** | **-0.12** | **-0.14 (-0.15 to -0.12)** | |
| **Cameroon** | **-0.16** | **-0.21** | **-0.11** | **-0.16 (-0.21 to -0.11)** | |
| **Saint Kitts and Nevis** | **-0.24** | **-0.27** | **-0.21** | **-0.24 (-0.27 to -0.21)** | |
| **Gambia** | **-0.13** | **-0.19** | **-0.07** | **-0.13 (-0.19 to -0.07)** | |
| **Ghana** | **-0.19** | **-0.23** | **-0.15** | **-0.19 (-0.23 to -0.15)** | |
| **Bhutan** | **0.01** | **-0.01** | **0.03** | **0.01 (-0.01 to 0.03)** | |
| **Guinea** | **-0.14** | **-0.18** | **-0.09** | **-0.14 (-0.18 to -0.09)** | |
| **United States Virgin Islands** | **0.09** | **0.07** | **0.11** | **0.09 (0.07 to 0.11)** | |
| **Puerto Rico** | **0.01** | **0** | **0.02** | **0.01 (0.00 to 0.02)** | |
| **Cabo Verde** | **-0.2** | **-0.27** | **-0.12** | **-0.20 (-0.27 to -0.12)** | |
| **Nauru** | **-0.02** | **-0.03** | **0** | **-0.02 (-0.03 to -0.00)** | |
| **Eritrea** | **0.06** | **0.05** | **0.08** | **0.06 (0.05 to 0.08)** | |
| **Ethiopia** | **-0.22** | **-0.23** | **-0.21** | **-0.22 (-0.23 to -0.21)** | |
| **Mozambique** | **-0.04** | **-0.05** | **-0.03** | **-0.04 (-0.05 to -0.03)** | |
| **San Marino** | **-0.09** | **-0.11** | **-0.06** | **-0.09 (-0.11 to -0.06)** | |
| **Comoros** | **-0.01** | **-0.03** | **0.01** | **-0.01 (-0.03 to 0.01)** | |
| **Rwanda** | **-0.13** | **-0.16** | **-0.1** | **-0.13 (-0.16 to -0.10)** | |
| **Niger** | **0.03** | **0** | **0.06** | **0.03 (-0.00 to 0.06)** | |
| **Mauritius** | **-0.13** | **-0.16** | **-0.11** | **-0.13 (-0.16 to -0.11)** | |
| **Eswatini** | **-0.1** | **-0.12** | **-0.07** | **-0.10 (-0.12 to -0.07)** | |
| **Lesotho** | **-0.13** | **-0.15** | **-0.11** | **-0.13 (-0.15 to -0.11)** | |
| **Namibia** | **-0.03** | **-0.04** | **-0.01** | **-0.03 (-0.04 to -0.01)** | |
| **Guinea-Bissau** | **-0.1** | **-0.14** | **-0.07** | **-0.10 (-0.14 to -0.07)** | |
| **Sao Tome and Principe** | **-0.14** | **-0.18** | **-0.09** | **-0.14 (-0.18 to -0.09)** | |
| **Guam** | **0** | **-0.01** | **0.01** | **0.00 (-0.01 to 0.01)** | |
| **Niue** | **-0.01** | **-0.04** | **0.02** | **-0.01 (-0.04 to 0.02)** | |
| **C么te d'Ivoire** | **-0.13** | **-0.17** | **-0.08** | **-0.13 (-0.17 to -0.08)** | |
| **Liberia** | **-0.18** | **-0.21** | **-0.15** | **-0.18 (-0.21 to -0.15)** | |
| **Mali** | **0.05** | **0.02** | **0.08** | **0.05 (0.02 to 0.08)** | |
| **Chad** | **-0.14** | **-0.19** | **-0.09** | **-0.14 (-0.19 to -0.09)** | |
| **Northern Mariana Islands** | **-0.16** | **-0.19** | **-0.13** | **-0.16 (-0.19 to -0.13)** | |
| **Sierra Leone** | **-0.17** | **-0.2** | **-0.14** | **-0.17 (-0.20 to -0.14)** | |
| **South Sudan** | **-0.19** | **-0.21** | **-0.17** | **-0.19 (-0.21 to -0.17)** | |
| **Nigeria** | **-0.24** | **-0.28** | **-0.2** | **-0.24 (-0.28 to -0.20)** | |
| **Cook Islands** | **0.11** | **0.08** | **0.14** | **0.11 (0.08 to 0.14)** | |
| **Greenland** | **-0.11** | **-0.15** | **-0.07** | **-0.11 (-0.15 to -0.07)** | |
| **Senegal** | **-0.2** | **-0.23** | **-0.17** | **-0.20 (-0.23 to -0.17)** | |
| **Togo** | **-0.17** | **-0.22** | **-0.13** | **-0.17 (-0.22 to -0.13)** | |
| **Monaco** | **-0.07** | **-0.09** | **-0.04** | **-0.07 (-0.09 to -0.04)** | |
| **Tuvalu** | **0** | **-0.01** | **0.01** | **-0.00 (-0.01 to 0.01)** | |
| **Tokelau** | **0.03** | **0.01** | **0.06** | **0.03 (0.01 to 0.06)** | |
| **Sudan** | **-0.1** | **-0.11** | **-0.09** | **-0.10 (-0.11 to -0.09)** | |

Appendix8: The EAPC of ASDR for postmenopausal women worldwide from 1990 to 2021

| location | EAPC | LCI | UCI | EAPC_CI |
| --- | --- | --- | --- | --- |
| Venezuela (Bolivarian Republic of) | -0.21 | -0.22 | -0.2 | -0.21 (-0.22 to -0.20) |
| Puerto Rico | -0.03 | -0.04 | -0.02 | -0.03 (-0.04 to -0.02) |
| Brazil | 0.16 | 0.11 | 0.21 | 0.16 (0.11 to 0.21) |
| American Samoa | -0.12 | -0.14 | -0.11 | -0.12 (-0.14 to -0.11) |
| Yemen | -0.22 | -0.24 | -0.2 | -0.22 (-0.24 to -0.20) |
| Kuwait | -0.15 | -0.22 | -0.08 | -0.15 (-0.22 to -0.08) |
| Myanmar | -0.03 | -0.06 | 0.01 | -0.03 (-0.06 to 0.01) |
| Brunei Darussalam | -0.22 | -0.25 | -0.18 | -0.22 (-0.25 to -0.18) |
| Turkmenistan | -0.15 | -0.18 | -0.11 | -0.15 (-0.18 to -0.11) |
| Malta | 0.03 | -0.02 | 0.08 | 0.03 (-0.02 to 0.08) |
| Denmark | -0.77 | -0.94 | -0.59 | -0.77 (-0.94 to -0.59) |
| Tajikistan | -0.28 | -0.34 | -0.22 | -0.28 (-0.34 to -0.22) |
| Belgium | -0.09 | -0.12 | -0.07 | -0.09 (-0.12 to -0.07) |
| Slovakia | -0.19 | -0.24 | -0.13 | -0.19 (-0.24 to -0.13) |
| Samoa | -0.24 | -0.28 | -0.2 | -0.24 (-0.28 to -0.20) |
| C么te d'Ivoire | -0.07 | -0.13 | -0.01 | -0.07 (-0.13 to -0.01) |
| Central African Republic | -0.07 | -0.1 | -0.04 | -0.07 (-0.10 to -0.04) |
| Norway | -0.2 | -0.27 | -0.13 | -0.20 (-0.27 to -0.13) |
| Montenegro | 0.08 | 0.06 | 0.09 | 0.08 (0.06 to 0.09) |
| Ghana | -0.19 | -0.24 | -0.14 | -0.19 (-0.24 to -0.14) |
| Lesotho | -0.24 | -0.27 | -0.22 | -0.24 (-0.27 to -0.22) |
| Sierra Leone | -0.19 | -0.22 | -0.16 | -0.19 (-0.22 to -0.16) |
| Uruguay | 0.2 | 0.14 | 0.26 | 0.20 (0.14 to 0.26) |
| Togo | -0.19 | -0.24 | -0.15 | -0.19 (-0.24 to -0.15) |
| Bermuda | -0.04 | -0.05 | -0.04 | -0.04 (-0.05 to -0.04) |
| Costa Rica | -0.18 | -0.19 | -0.18 | -0.18 (-0.19 to -0.18) |
| United States Virgin Islands | 0.05 | 0.03 | 0.06 | 0.05 (0.03 to 0.06) |
| Mauritius | -0.17 | -0.21 | -0.13 | -0.17 (-0.21 to -0.13) |
| Greenland | -0.06 | -0.11 | -0.02 | -0.06 (-0.11 to -0.02) |
| South Africa | -0.36 | -0.38 | -0.34 | -0.36 (-0.38 to -0.34) |
| Rwanda | -0.13 | -0.15 | -0.11 | -0.13 (-0.15 to -0.11) |
| Russian Federation | -0.02 | -0.05 | 0.02 | -0.02 (-0.05 to 0.02) |
| Haiti | -0.09 | -0.12 | -0.06 | -0.09 (-0.12 to -0.06) |
| Tokelau | 0.02 | 0 | 0.04 | 0.02 (-0.00 to 0.04) |
| Romania | -0.11 | -0.13 | -0.09 | -0.11 (-0.13 to -0.09) |
| Mongolia | -0.26 | -0.28 | -0.25 | -0.26 (-0.28 to -0.25) |
| United States of America | -0.06 | -0.1 | -0.02 | -0.06 (-0.10 to -0.02) |
| Guyana | -0.07 | -0.09 | -0.06 | -0.07 (-0.09 to -0.06) |
| Papua New Guinea | -0.03 | -0.06 | -0.01 | -0.03 (-0.06 to -0.01) |
| Libya | -0.14 | -0.18 | -0.11 | -0.14 (-0.18 to -0.11) |
| Spain | 0.19 | 0.11 | 0.28 | 0.19 (0.11 to 0.28) |
| Congo | -0.07 | -0.1 | -0.04 | -0.07 (-0.10 to -0.04) |
| Guinea | -0.15 | -0.2 | -0.09 | -0.15 (-0.20 to -0.09) |
| Peru | 0.17 | 0.14 | 0.21 | 0.17 (0.14 to 0.21) |
| Saint Lucia | -0.16 | -0.17 | -0.15 | -0.16 (-0.17 to -0.15) |
| Dominican Republic | 0.08 | 0.06 | 0.11 | 0.08 (0.06 to 0.11) |
| China | -0.49 | -0.59 | -0.39 | -0.49 (-0.59 to -0.39) |
| Vanuatu | -0.01 | -0.03 | 0.01 | -0.01 (-0.03 to 0.01) |
| Portugal | -0.02 | -0.07 | 0.04 | -0.02 (-0.07 to 0.04) |
| Republic of Korea | -0.16 | -0.2 | -0.12 | -0.16 (-0.20 to -0.12) |
| El Salvador | 0.03 | 0.02 | 0.04 | 0.03 (0.02 to 0.04) |
| Guatemala | -0.11 | -0.15 | -0.08 | -0.11 (-0.15 to -0.08) |
| Eswatini | -0.16 | -0.19 | -0.13 | -0.16 (-0.19 to -0.13) |
| Guinea-Bissau | -0.11 | -0.15 | -0.08 | -0.11 (-0.15 to -0.08) |
| Armenia | 0.1 | 0.06 | 0.15 | 0.10 (0.06 to 0.15) |
| Madagascar | -0.32 | -0.36 | -0.28 | -0.32 (-0.36 to -0.28) |
| Grenada | -0.04 | -0.06 | -0.03 | -0.04 (-0.06 to -0.03) |
| Bangladesh | -0.25 | -0.33 | -0.18 | -0.25 (-0.33 to -0.18) |
| Andorra | -0.16 | -0.19 | -0.14 | -0.16 (-0.19 to -0.14) |
| Algeria | -0.09 | -0.12 | -0.05 | -0.09 (-0.12 to -0.05) |
| Palestine | -0.14 | -0.18 | -0.1 | -0.14 (-0.18 to -0.10) |
| Gambia | -0.16 | -0.22 | -0.1 | -0.16 (-0.22 to -0.10) |
| Gabon | -0.08 | -0.11 | -0.05 | -0.08 (-0.11 to -0.05) |
| Saudi Arabia | -0.11 | -0.13 | -0.09 | -0.11 (-0.13 to -0.09) |
| Austria | -0.06 | -0.09 | -0.02 | -0.06 (-0.09 to -0.02) |
| Guam | -0.02 | -0.03 | -0.02 | -0.02 (-0.03 to -0.02) |
| Bahrain | -0.15 | -0.19 | -0.1 | -0.15 (-0.19 to -0.10) |
| San Marino | -0.14 | -0.16 | -0.12 | -0.14 (-0.16 to -0.12) |
| Democratic Republic of the Congo | -0.1 | -0.15 | -0.06 | -0.10 (-0.15 to -0.06) |
| Italy | -0.09 | -0.12 | -0.06 | -0.09 (-0.12 to -0.06) |
| Sri Lanka | -0.09 | -0.1 | -0.07 | -0.09 (-0.10 to -0.07) |
| Bulgaria | 0.01 | 0 | 0.02 | 0.01 (0.00 to 0.02) |
| France | 0.04 | -0.01 | 0.08 | 0.04 (-0.01 to 0.08) |
| Poland | -0.16 | -0.18 | -0.14 | -0.16 (-0.18 to -0.14) |
| Burkina Faso | -0.12 | -0.16 | -0.09 | -0.12 (-0.16 to -0.09) |
| Equatorial Guinea | -0.15 | -0.17 | -0.12 | -0.15 (-0.17 to -0.12) |
| Seychelles | -0.27 | -0.31 | -0.24 | -0.27 (-0.31 to -0.24) |
| Colombia | -0.01 | -0.04 | 0.02 | -0.01 (-0.04 to 0.02) |
| Mali | 0.11 | 0.07 | 0.14 | 0.11 (0.07 to 0.14) |
| Czechia | -0.05 | -0.07 | -0.04 | -0.05 (-0.07 to -0.04) |
| Australia | -0.09 | -0.12 | -0.05 | -0.09 (-0.12 to -0.05) |
| Georgia | -0.13 | -0.17 | -0.08 | -0.13 (-0.17 to -0.08) |
| Zimbabwe | 0.14 | 0.13 | 0.15 | 0.14 (0.13 to 0.15) |
| Lebanon | 0.16 | 0.14 | 0.19 | 0.16 (0.14 to 0.19) |
| Tuvalu | 0.01 | 0 | 0.02 | 0.01 (-0.00 to 0.02) |
| Solomon Islands | 0.26 | 0.22 | 0.29 | 0.26 (0.22 to 0.29) |
| Belarus | 0.04 | 0 | 0.08 | 0.04 (0.00 to 0.08) |
| Iceland | -0.22 | -0.25 | -0.19 | -0.22 (-0.25 to -0.19) |
| Thailand | 0.27 | 0.21 | 0.33 | 0.27 (0.21 to 0.33) |
| Dominica | -0.1 | -0.13 | -0.08 | -0.10 (-0.13 to -0.08) |
| Albania | 0.05 | 0.04 | 0.05 | 0.05 (0.04 to 0.05) |
| Saint Kitts and Nevis | -0.24 | -0.27 | -0.22 | -0.24 (-0.27 to -0.22) |
| Japan | -0.13 | -0.19 | -0.07 | -0.13 (-0.19 to -0.07) |
| Liberia | -0.18 | -0.2 | -0.15 | -0.18 (-0.20 to -0.15) |
| Ukraine | -0.09 | -0.16 | -0.02 | -0.09 (-0.16 to -0.02) |
| Afghanistan | 0.12 | 0.09 | 0.15 | 0.12 (0.09 to 0.15) |
| Antigua and Barbuda | -0.09 | -0.1 | -0.09 | -0.09 (-0.10 to -0.09) |
| Bhutan | 0.01 | -0.01 | 0.03 | 0.01 (-0.01 to 0.03) |
| Slovenia | -0.03 | -0.04 | -0.02 | -0.03 (-0.04 to -0.02) |
| Latvia | 0.08 | 0.06 | 0.09 | 0.08 (0.06 to 0.09) |
| Namibia | -0.03 | -0.04 | -0.01 | -0.03 (-0.04 to -0.01) |
| Tonga | -0.08 | -0.1 | -0.06 | -0.08 (-0.10 to -0.06) |
| South Sudan | -0.19 | -0.21 | -0.18 | -0.19 (-0.21 to -0.18) |
| Cook Islands | 0.08 | 0.05 | 0.11 | 0.08 (0.05 to 0.11) |
| Oman | -0.13 | -0.15 | -0.11 | -0.13 (-0.15 to -0.11) |
| Bosnia and Herzegovina | 0.1 | 0.07 | 0.13 | 0.10 (0.07 to 0.13) |
| United Kingdom | 0.95 | 0.79 | 1.11 | 0.95 (0.79 to 1.11) |
| Viet Nam | -0.03 | -0.12 | 0.07 | -0.03 (-0.12 to 0.07) |
| Luxembourg | -0.09 | -0.13 | -0.04 | -0.09 (-0.13 to -0.04) |
| Uzbekistan | -0.15 | -0.18 | -0.11 | -0.15 (-0.18 to -0.11) |
| Qatar | -0.04 | -0.05 | -0.03 | -0.04 (-0.05 to -0.03) |
| Barbados | -0.1 | -0.1 | -0.09 | -0.10 (-0.10 to -0.09) |
| Taiwan (Province of China) | 0.71 | 0.58 | 0.84 | 0.71 (0.58 to 0.84) |
| Nauru | -0.03 | -0.04 | -0.02 | -0.03 (-0.04 to -0.02) |
| Paraguay | 0.17 | 0.12 | 0.21 | 0.17 (0.12 to 0.21) |
| Canada | -0.24 | -0.3 | -0.18 | -0.24 (-0.30 to -0.18) |
| Suriname | 0.05 | 0.02 | 0.08 | 0.05 (0.02 to 0.08) |
| Cyprus | -0.06 | -0.08 | -0.05 | -0.06 (-0.08 to -0.05) |
| Malawi | -0.05 | -0.08 | -0.03 | -0.05 (-0.08 to -0.03) |
| Chile | -0.02 | -0.06 | 0.03 | -0.02 (-0.06 to 0.03) |
| Mexico | -0.09 | -0.2 | 0.02 | -0.09 (-0.20 to 0.02) |
| United Republic of Tanzania | -0.06 | -0.08 | -0.03 | -0.06 (-0.08 to -0.03) |
| Philippines | -0.12 | -0.15 | -0.08 | -0.12 (-0.15 to -0.08) |
| Indonesia | -0.02 | -0.05 | 0.02 | -0.02 (-0.05 to 0.02) |
| Iraq | -0.15 | -0.16 | -0.14 | -0.15 (-0.16 to -0.14) |
| Kiribati | 0.08 | 0.05 | 0.11 | 0.08 (0.05 to 0.11) |
| Ecuador | -0.16 | -0.25 | -0.06 | -0.16 (-0.25 to -0.06) |
| Mozambique | -0.03 | -0.04 | -0.01 | -0.03 (-0.04 to -0.01) |
| Serbia | 0.08 | 0.07 | 0.1 | 0.08 (0.07 to 0.10) |
| Iran (Islamic Republic of) | -0.18 | -0.21 | -0.15 | -0.18 (-0.21 to -0.15) |
| Comoros | -0.01 | -0.03 | 0.01 | -0.01 (-0.03 to 0.01) |
| Netherlands | 0.16 | 0.02 | 0.3 | 0.16 (0.02 to 0.30) |
| Saint Vincent and the Grenadines | -0.07 | -0.09 | -0.06 | -0.07 (-0.09 to -0.06) |
| T眉rkiye | 0.17 | 0.12 | 0.21 | 0.17 (0.12 to 0.21) |
| Trinidad and Tobago | -0.05 | -0.06 | -0.04 | -0.05 (-0.06 to -0.04) |
| Tunisia | -0.06 | -0.11 | -0.01 | -0.06 (-0.11 to -0.01) |
| Sudan | -0.1 | -0.12 | -0.09 | -0.10 (-0.12 to -0.09) |
| Democratic People's Republic of Korea | -0.05 | -0.08 | -0.02 | -0.05 (-0.08 to -0.02) |
| Bahamas | -0.07 | -0.08 | -0.06 | -0.07 (-0.08 to -0.06) |
| Timor-Leste | 0 | -0.04 | 0.04 | 0.00 (-0.04 to 0.04) |
| Nicaragua | -0.07 | -0.08 | -0.06 | -0.07 (-0.08 to -0.06) |
| Malaysia | 0.02 | -0.03 | 0.07 | 0.02 (-0.03 to 0.07) |
| Niger | 0.11 | 0.07 | 0.14 | 0.11 (0.07 to 0.14) |
| Nepal | -0.29 | -0.38 | -0.19 | -0.29 (-0.38 to -0.19) |
| Finland | -0.09 | -0.13 | -0.06 | -0.09 (-0.13 to -0.06) |
| Fiji | -0.08 | -0.1 | -0.06 | -0.08 (-0.10 to -0.06) |
| Singapore | -0.11 | -0.17 | -0.05 | -0.11 (-0.17 to -0.05) |
| Cuba | -0.04 | -0.08 | 0 | -0.04 (-0.08 to -0.00) |
| Jamaica | -0.1 | -0.13 | -0.07 | -0.10 (-0.13 to -0.07) |
| Argentina | -0.01 | -0.05 | 0.04 | -0.01 (-0.05 to 0.04) |
| Morocco | -0.11 | -0.23 | 0 | -0.11 (-0.23 to 0.00) |
| Cambodia | -0.21 | -0.23 | -0.19 | -0.21 (-0.23 to -0.19) |
| Bolivia (Plurinational State of) | 0.02 | -0.02 | 0.06 | 0.02 (-0.02 to 0.06) |
| Germany | -0.09 | -0.14 | -0.05 | -0.09 (-0.14 to -0.05) |
| India | -0.31 | -0.4 | -0.22 | -0.31 (-0.40 to -0.22) |
| Honduras | -0.03 | -0.04 | -0.01 | -0.03 (-0.04 to -0.01) |
| Burundi | -0.2 | -0.22 | -0.18 | -0.20 (-0.22 to -0.18) |
| Croatia | 0.38 | 0.22 | 0.54 | 0.38 (0.22 to 0.54) |
| Estonia | 0.13 | 0.1 | 0.16 | 0.13 (0.10 to 0.16) |
| Azerbaijan | 0 | -0.05 | 0.04 | -0.00 (-0.05 to 0.04) |
| Zambia | 0.18 | 0.12 | 0.24 | 0.18 (0.12 to 0.24) |
| Sweden | 0.45 | 0.34 | 0.55 | 0.45 (0.34 to 0.55) |
| Eritrea | 0.1 | 0.08 | 0.11 | 0.10 (0.08 to 0.11) |
| Syrian Arab Republic | -0.08 | -0.1 | -0.05 | -0.08 (-0.10 to -0.05) |
| Egypt | 0.04 | -0.01 | 0.08 | 0.04 (-0.01 to 0.08) |
| Greece | 0.06 | 0.04 | 0.09 | 0.06 (0.04 to 0.09) |
| Benin | -0.15 | -0.19 | -0.11 | -0.15 (-0.19 to -0.11) |
| Micronesia (Federated States of) | -0.14 | -0.15 | -0.13 | -0.14 (-0.15 to -0.13) |
| Lao People's Democratic Republic | -0.12 | -0.14 | -0.11 | -0.12 (-0.14 to -0.11) |
| Somalia | 0 | -0.01 | 0.02 | 0.00 (-0.01 to 0.02) |
| Monaco | -0.12 | -0.14 | -0.1 | -0.12 (-0.14 to -0.10) |
| Israel | -0.11 | -0.14 | -0.09 | -0.11 (-0.14 to -0.09) |
| North Macedonia | 0.05 | 0.03 | 0.06 | 0.05 (0.03 to 0.06) |
| Marshall Islands | -0.28 | -0.31 | -0.25 | -0.28 (-0.31 to -0.25) |
| Cabo Verde | -0.25 | -0.33 | -0.16 | -0.25 (-0.33 to -0.16) |
| Switzerland | 0.26 | 0.2 | 0.32 | 0.26 (0.20 to 0.32) |
| Kyrgyzstan | -0.2 | -0.25 | -0.15 | -0.20 (-0.25 to -0.15) |
| Mauritania | -0.06 | -0.12 | -0.01 | -0.06 (-0.12 to -0.01) |
| Republic of Moldova | -0.05 | -0.09 | -0.01 | -0.05 (-0.09 to -0.01) |
| New Zealand | -0.09 | -0.12 | -0.07 | -0.09 (-0.12 to -0.07) |
| Northern Mariana Islands | -0.19 | -0.21 | -0.16 | -0.19 (-0.21 to -0.16) |
| Sao Tome and Principe | -0.15 | -0.19 | -0.11 | -0.15 (-0.19 to -0.11) |
| Djibouti | -0.17 | -0.18 | -0.15 | -0.17 (-0.18 to -0.15) |
| Uganda | -0.14 | -0.15 | -0.12 | -0.14 (-0.15 to -0.12) |
| Hungary | 0.04 | 0.03 | 0.05 | 0.04 (0.03 to 0.05) |
| Maldives | 0.06 | 0.01 | 0.11 | 0.06 (0.01 to 0.11) |
| Lithuania | 0.04 | 0.02 | 0.06 | 0.04 (0.02 to 0.06) |
| Cameroon | -0.18 | -0.23 | -0.12 | -0.18 (-0.23 to -0.12) |
| Niue | -0.06 | -0.09 | -0.03 | -0.06 (-0.09 to -0.03) |
| Kazakhstan | -0.09 | -0.12 | -0.07 | -0.09 (-0.12 to -0.07) |
| Pakistan | 0.21 | 0.15 | 0.26 | 0.21 (0.15 to 0.26) |
| Belize | -0.06 | -0.07 | -0.05 | -0.06 (-0.07 to -0.05) |
| Nigeria | -0.24 | -0.28 | -0.2 | -0.24 (-0.28 to -0.20) |
| Ireland | -0.16 | -0.19 | -0.14 | -0.16 (-0.19 to -0.14) |
| Angola | -0.21 | -0.24 | -0.17 | -0.21 (-0.24 to -0.17) |
| Palau | -0.18 | -0.19 | -0.16 | -0.18 (-0.19 to -0.16) |
| Panama | -0.04 | -0.06 | -0.03 | -0.04 (-0.06 to -0.03) |
| Jordan | 0.01 | 0 | 0.03 | 0.01 (-0.00 to 0.03) |
| Kenya | -0.18 | -0.22 | -0.13 | -0.18 (-0.22 to -0.13) |
| Ethiopia | -0.26 | -0.27 | -0.24 | -0.26 (-0.27 to -0.24) |
| Chad | -0.15 | -0.21 | -0.09 | -0.15 (-0.21 to -0.09) |
| United Arab Emirates | -0.3 | -0.37 | -0.22 | -0.30 (-0.37 to -0.22) |
| Botswana | -0.09 | -0.11 | -0.07 | -0.09 (-0.11 to -0.07) |
| Senegal | -0.24 | -0.27 | -0.2 | -0.24 (-0.27 to -0.20) |

Appendix9: In 2021, the correlation between DALYs attributed to postmenopausal low back pain and the Socio-Demographic Index (SDI) across global regions

| measure | location | sex | age | cause | rei | metric | year | val | upper | lower |
| --- | --- | --- | --- | --- | --- | --- | --- | --- | --- | --- |
| DALYs (Disability-Adjusted Life Years) | Andean Latin America | Female | 55+ years | Low back pain | Smoking | Number | 2021 | 2921.5433 | 4829.99773 | 1602.72358 |
| DALYs (Disability-Adjusted Life Years) | Andean Latin America | Female | 55+ years | Low back pain | High body-mass index | Number | 2021 | 16311.7855 | 33757.3418 | 1771.64851 |
| DALYs (Disability-Adjusted Life Years) | Andean Latin America | Female | 55+ years | Low back pain | Environmental/occupational risks | Number | 2021 | 15460.3196 | 21571.3961 | 10223.0997 |
| DALYs (Disability-Adjusted Life Years) | Australasia | Female | 55+ years | Low back pain | High body-mass index | Number | 2021 | 31384.6423 | 65164.9823 | 3363.35755 |
| DALYs (Disability-Adjusted Life Years) | Australasia | Female | 55+ years | Low back pain | Smoking | Number | 2021 | 18401.1908 | 28520.8324 | 10187.5349 |
| DALYs (Disability-Adjusted Life Years) | Australasia | Female | 55+ years | Low back pain | Environmental/occupational risks | Number | 2021 | 10626.5505 | 15553.4638 | 6793.09267 |
| DALYs (Disability-Adjusted Life Years) | Caribbean | Female | 55+ years | Low back pain | Smoking | Number | 2021 | 8393.58574 | 13610.1702 | 4716.46572 |
| DALYs (Disability-Adjusted Life Years) | Caribbean | Female | 55+ years | Low back pain | High body-mass index | Number | 2021 | 15334.1459 | 31173.4736 | 1633.81287 |
| DALYs (Disability-Adjusted Life Years) | Caribbean | Female | 55+ years | Low back pain | Environmental/occupational risks | Number | 2021 | 6454.75864 | 9363.07992 | 4170.8094 |
| DALYs (Disability-Adjusted Life Years) | Central Asia | Female | 55+ years | Low back pain | Smoking | Number | 2021 | 5411.11362 | 9337.9419 | 2784.18299 |
| DALYs (Disability-Adjusted Life Years) | Central Asia | Female | 55+ years | Low back pain | High body-mass index | Number | 2021 | 45868.8433 | 94149.5687 | 4811.72817 |
| DALYs (Disability-Adjusted Life Years) | Central Asia | Female | 55+ years | Low back pain | Environmental/occupational risks | Number | 2021 | 38213.2656 | 54964.1811 | 24865.9648 |
| DALYs (Disability-Adjusted Life Years) | Central Europe | Female | 55+ years | Low back pain | Smoking | Number | 2021 | 95985.5492 | 150134.527 | 54752.2293 |
| DALYs (Disability-Adjusted Life Years) | Central Europe | Female | 55+ years | Low back pain | High body-mass index | Number | 2021 | 149875.8 | 306747.283 | 15700.7764 |
| DALYs (Disability-Adjusted Life Years) | Central Europe | Female | 55+ years | Low back pain | Environmental/occupational risks | Number | 2021 | 52096.351 | 75737.252 | 34462.5806 |
| DALYs (Disability-Adjusted Life Years) | Central Latin America | Female | 55+ years | Low back pain | Smoking | Number | 2021 | 24992.8807 | 40240.3672 | 13916.6075 |
| DALYs (Disability-Adjusted Life Years) | Central Latin America | Female | 55+ years | Low back pain | High body-mass index | Number | 2021 | 97745.7895 | 199571.988 | 10740.3532 |
| DALYs (Disability-Adjusted Life Years) | Central Latin America | Female | 55+ years | Low back pain | Environmental/occupational risks | Number | 2021 | 40598.7599 | 58662.2533 | 27196.9581 |
| DALYs (Disability-Adjusted Life Years) | Central Sub-Saharan Africa | Female | 55+ years | Low back pain | Smoking | Number | 2021 | 1795.26502 | 3108.83625 | 913.545694 |
| DALYs (Disability-Adjusted Life Years) | Central Sub-Saharan Africa | Female | 55+ years | Low back pain | High body-mass index | Number | 2021 | 11868.3646 | 26060.3384 | 1244.8607 |
| DALYs (Disability-Adjusted Life Years) | Central Sub-Saharan Africa | Female | 55+ years | Low back pain | Environmental/occupational risks | Number | 2021 | 35686.3651 | 52336.2177 | 23122.2811 |
| DALYs (Disability-Adjusted Life Years) | East Asia | Female | 55+ years | Low back pain | High body-mass index | Number | 2021 | 439604.917 | 945870.457 | 48284.389 |
| DALYs (Disability-Adjusted Life Years) | East Asia | Female | 55+ years | Low back pain | Environmental/occupational risks | Number | 2021 | 796289.808 | 1180678.52 | 492457.08 |
| DALYs (Disability-Adjusted Life Years) | East Asia | Female | 55+ years | Low back pain | Smoking | Number | 2021 | 207138.327 | 329039.88 | 108200.842 |
| DALYs (Disability-Adjusted Life Years) | Eastern Europe | Female | 55+ years | Low back pain | Smoking | Number | 2021 | 63743.2092 | 106180.833 | 35368.6785 |
| DALYs (Disability-Adjusted Life Years) | Eastern Europe | Female | 55+ years | Low back pain | High body-mass index | Number | 2021 | 314160.726 | 632799.152 | 33885.2461 |
| DALYs (Disability-Adjusted Life Years) | Eastern Europe | Female | 55+ years | Low back pain | Environmental/occupational risks | Number | 2021 | 76101.7648 | 111768.609 | 48367.0531 |
| DALYs (Disability-Adjusted Life Years) | Eastern Sub-Saharan Africa | Female | 55+ years | Low back pain | Smoking | Number | 2021 | 11583.1691 | 18844.3442 | 6357.12901 |
| DALYs (Disability-Adjusted Life Years) | Eastern Sub-Saharan Africa | Female | 55+ years | Low back pain | High body-mass index | Number | 2021 | 31560.0023 | 67722.9379 | 3231.35039 |
| DALYs (Disability-Adjusted Life Years) | Eastern Sub-Saharan Africa | Female | 55+ years | Low back pain | Environmental/occupational risks | Number | 2021 | 131036.881 | 185973.038 | 86903.3494 |
| DALYs (Disability-Adjusted Life Years) | Global | Female | 55+ years | Low back pain | Smoking | Number | 2021 | 1392955.5 | 2163179.51 | 772161.019 |
| DALYs (Disability-Adjusted Life Years) | Global | Female | 55+ years | Low back pain | High body-mass index | Number | 2021 | 2708943.98 | 5543616.58 | 292321.708 |
| DALYs (Disability-Adjusted Life Years) | Global | Female | 55+ years | Low back pain | Environmental/occupational risks | Number | 2021 | 2400873.81 | 3425391.54 | 1604144.38 |
| DALYs (Disability-Adjusted Life Years) | High SDI | Female | 55+ years | Low back pain | Environmental/occupational risks | Number | 2021 | 309366.22 | 425658.112 | 207497.579 |
| DALYs (Disability-Adjusted Life Years) | High SDI | Female | 55+ years | Low back pain | Smoking | Number | 2021 | 611672.278 | 933711.313 | 348756.301 |
| DALYs (Disability-Adjusted Life Years) | High SDI | Female | 55+ years | Low back pain | High body-mass index | Number | 2021 | 825871.143 | 1666899.55 | 87584.6654 |
| DALYs (Disability-Adjusted Life Years) | High-income Asia Pacific | Female | 55+ years | Low back pain | High body-mass index | Number | 2021 | 75481.3165 | 150459.311 | 7890.84682 |
| DALYs (Disability-Adjusted Life Years) | High-income Asia Pacific | Female | 55+ years | Low back pain | Smoking | Number | 2021 | 64773.9695 | 105593.567 | 33728.0898 |
| DALYs (Disability-Adjusted Life Years) | High-income Asia Pacific | Female | 55+ years | Low back pain | Environmental/occupational risks | Number | 2021 | 80848.9957 | 115813.053 | 51938.5459 |
| DALYs (Disability-Adjusted Life Years) | High-income North America | Female | 55+ years | Low back pain | High body-mass index | Number | 2021 | 335531.609 | 658310.156 | 35586.2496 |
| DALYs (Disability-Adjusted Life Years) | High-income North America | Female | 55+ years | Low back pain | Smoking | Number | 2021 | 236129.217 | 358943.985 | 134504.959 |
| DALYs (Disability-Adjusted Life Years) | High-income North America | Female | 55+ years | Low back pain | Environmental/occupational risks | Number | 2021 | 92088.5672 | 127846.253 | 61425.6498 |
| DALYs (Disability-Adjusted Life Years) | High-middle SDI | Female | 55+ years | Low back pain | Smoking | Number | 2021 | 345995.141 | 557034.413 | 192039.599 |
| DALYs (Disability-Adjusted Life Years) | High-middle SDI | Female | 55+ years | Low back pain | High body-mass index | Number | 2021 | 832541.097 | 1693283.36 | 90285.8011 |
| DALYs (Disability-Adjusted Life Years) | High-middle SDI | Female | 55+ years | Low back pain | Environmental/occupational risks | Number | 2021 | 472898.193 | 694968.909 | 310218.402 |
| DALYs (Disability-Adjusted Life Years) | Low SDI | Female | 55+ years | Low back pain | Smoking | Number | 2021 | 44594.7586 | 72017.6505 | 23340.2945 |
| DALYs (Disability-Adjusted Life Years) | Low SDI | Female | 55+ years | Low back pain | High body-mass index | Number | 2021 | 73166.8904 | 158889.1 | 7641.47862 |
| DALYs (Disability-Adjusted Life Years) | Low SDI | Female | 55+ years | Low back pain | Environmental/occupational risks | Number | 2021 | 260796.611 | 380210.606 | 175654.466 |
| DALYs (Disability-Adjusted Life Years) | Low-middle SDI | Female | 55+ years | Low back pain | Smoking | Number | 2021 | 142204.229 | 228987.286 | 75799.7667 |
| DALYs (Disability-Adjusted Life Years) | Low-middle SDI | Female | 55+ years | Low back pain | High body-mass index | Number | 2021 | 304503.729 | 640655.878 | 33363.755 |
| DALYs (Disability-Adjusted Life Years) | Low-middle SDI | Female | 55+ years | Low back pain | Environmental/occupational risks | Number | 2021 | 514513.689 | 743436.984 | 339227.022 |
| DALYs (Disability-Adjusted Life Years) | Middle SDI | Female | 55+ years | Low back pain | Smoking | Number | 2021 | 246359.236 | 397781.792 | 132772.009 |
| DALYs (Disability-Adjusted Life Years) | Middle SDI | Female | 55+ years | Low back pain | High body-mass index | Number | 2021 | 669380.899 | 1404231.43 | 72040.748 |
| DALYs (Disability-Adjusted Life Years) | Middle SDI | Female | 55+ years | Low back pain | Environmental/occupational risks | Number | 2021 | 841626.744 | 1206315.39 | 553257.724 |
| DALYs (Disability-Adjusted Life Years) | North Africa and Middle East | Female | 55+ years | Low back pain | Smoking | Number | 2021 | 35073.2911 | 58323.9699 | 18841.2503 |
| DALYs (Disability-Adjusted Life Years) | North Africa and Middle East | Female | 55+ years | Low back pain | High body-mass index | Number | 2021 | 207051.028 | 407253.581 | 23811.5304 |
| DALYs (Disability-Adjusted Life Years) | North Africa and Middle East | Female | 55+ years | Low back pain | Environmental/occupational risks | Number | 2021 | 46697.389 | 69232.4794 | 29258.5905 |
| DALYs (Disability-Adjusted Life Years) | Oceania | Female | 55+ years | Low back pain | Smoking | Number | 2021 | 1487.87227 | 2417.82577 | 788.39745 |
| DALYs (Disability-Adjusted Life Years) | Oceania | Female | 55+ years | Low back pain | High body-mass index | Number | 2021 | 1850.41371 | 3834.81673 | 207.195847 |
| DALYs (Disability-Adjusted Life Years) | Oceania | Female | 55+ years | Low back pain | Environmental/occupational risks | Number | 2021 | 1806.99328 | 2901.52051 | 1123.67264 |
| DALYs (Disability-Adjusted Life Years) | South Asia | Female | 55+ years | Low back pain | Smoking | Number | 2021 | 136427.012 | 226606.573 | 70828.6034 |
| DALYs (Disability-Adjusted Life Years) | South Asia | Female | 55+ years | Low back pain | High body-mass index | Number | 2021 | 217178.286 | 460499.6 | 22941.6436 |
| DALYs (Disability-Adjusted Life Years) | South Asia | Female | 55+ years | Low back pain | Environmental/occupational risks | Number | 2021 | 401246.277 | 593410.592 | 254202.165 |
| DALYs (Disability-Adjusted Life Years) | Southeast Asia | Female | 55+ years | Low back pain | Smoking | Number | 2021 | 50941.9402 | 83342.7053 | 27746.9346 |
| DALYs (Disability-Adjusted Life Years) | Southeast Asia | Female | 55+ years | Low back pain | High body-mass index | Number | 2021 | 101772.538 | 222270.103 | 10552.5461 |
| DALYs (Disability-Adjusted Life Years) | Southeast Asia | Female | 55+ years | Low back pain | Environmental/occupational risks | Number | 2021 | 249521.742 | 362534.434 | 163792.683 |
| DALYs (Disability-Adjusted Life Years) | Southern Latin America | Female | 55+ years | Low back pain | High body-mass index | Number | 2021 | 50970.2838 | 104848.767 | 5641.26479 |
| DALYs (Disability-Adjusted Life Years) | Southern Latin America | Female | 55+ years | Low back pain | Smoking | Number | 2021 | 33160.8622 | 53963.0816 | 18037.8387 |
| DALYs (Disability-Adjusted Life Years) | Southern Latin America | Female | 55+ years | Low back pain | Environmental/occupational risks | Number | 2021 | 16733.2617 | 23539.7413 | 10772.253 |
| DALYs (Disability-Adjusted Life Years) | Southern Sub-Saharan Africa | Female | 55+ years | Low back pain | High body-mass index | Number | 2021 | 29894.4462 | 60270.9232 | 3267.41725 |
| DALYs (Disability-Adjusted Life Years) | Southern Sub-Saharan Africa | Female | 55+ years | Low back pain | Smoking | Number | 2021 | 7526.93826 | 12601.7188 | 3991.17761 |
| DALYs (Disability-Adjusted Life Years) | Southern Sub-Saharan Africa | Female | 55+ years | Low back pain | Environmental/occupational risks | Number | 2021 | 15220.4784 | 21847.2243 | 10043.7735 |
| DALYs (Disability-Adjusted Life Years) | Tropical Latin America | Female | 55+ years | Low back pain | Smoking | Number | 2021 | 83508.7271 | 134349.511 | 45489.1016 |
| DALYs (Disability-Adjusted Life Years) | Tropical Latin America | Female | 55+ years | Low back pain | High body-mass index | Number | 2021 | 116692.857 | 243389.859 | 12317.3096 |
| DALYs (Disability-Adjusted Life Years) | Tropical Latin America | Female | 55+ years | Low back pain | Environmental/occupational risks | Number | 2021 | 58215.6661 | 88765.4131 | 35310.6298 |
| DALYs (Disability-Adjusted Life Years) | Western Europe | Female | 55+ years | Low back pain | Smoking | Number | 2021 | 298088.64 | 455818.879 | 170397.672 |
| DALYs (Disability-Adjusted Life Years) | Western Europe | Female | 55+ years | Low back pain | High body-mass index | Number | 2021 | 369729.627 | 764311.766 | 38187.7281 |
| DALYs (Disability-Adjusted Life Years) | Western Europe | Female | 55+ years | Low back pain | Environmental/occupational risks | Number | 2021 | 119389.82 | 172676.513 | 78690.4679 |
| DALYs (Disability-Adjusted Life Years) | Western Sub-Saharan Africa | Female | 55+ years | Low back pain | High body-mass index | Number | 2021 | 49076.5561 | 104843.664 | 5176.48977 |
| DALYs (Disability-Adjusted Life Years) | Western Sub-Saharan Africa | Female | 55+ years | Low back pain | Smoking | Number | 2021 | 5471.19556 | 9064.3778 | 2910.55897 |
| DALYs (Disability-Adjusted Life Years) | Western Sub-Saharan Africa | Female | 55+ years | Low back pain | Environmental/occupational risks | Number | 2021 | 116539.798 | 167096.566 | 77888.504 |

Appendix10: Global analysis of incidence/prevalence/DALYs of low back pain in postmenopausal women across different age groups and regions from 1990 to 2021

| **measure** | **location** | **sex** | **age** | **cause** | **metric** | **year** | **val** | **upper** | **lower** |
| --- | --- | --- | --- | --- | --- | --- | --- | --- | --- |
| **DALYs (Disability-Adjusted Life Years)** | **Global** | **Female** | **55-59 years** | **Low back pain** | **Number** | **1990** | **2190077.54** | **3227720.98** | **1378957.64** |
| **DALYs (Disability-Adjusted Life Years)** | **Global** | **Female** | **55-59 years** | **Low back pain** | **Rate** | **1990** | **2372.47524** | **3496.53744** | **1493.80229** |
| **DALYs (Disability-Adjusted Life Years)** | **Global** | **Female** | **55-59 years** | **Low back pain** | **Number** | **1992** | **2209702.24** | **3244608.37** | **1392912.13** |
| **DALYs (Disability-Adjusted Life Years)** | **Global** | **Female** | **55-59 years** | **Low back pain** | **Rate** | **1992** | **2315.2131** | **3399.53485** | **1459.42215** |
| **DALYs (Disability-Adjusted Life Years)** | **Global** | **Female** | **55-59 years** | **Low back pain** | **Number** | **1991** | **2192946.06** | **3225969.69** | **1380951.76** |
| **DALYs (Disability-Adjusted Life Years)** | **Global** | **Female** | **55-59 years** | **Low back pain** | **Rate** | **1991** | **2340.6713** | **3443.28335** | **1473.97795** |
| **DALYs (Disability-Adjusted Life Years)** | **Global** | **Female** | **55-59 years** | **Low back pain** | **Number** | **1993** | **2230166.31** | **3269927.37** | **1403361.39** |
| **DALYs (Disability-Adjusted Life Years)** | **Global** | **Female** | **55-59 years** | **Low back pain** | **Rate** | **1993** | **2296.4282** | **3367.08227** | **1445.05756** |
| **DALYs (Disability-Adjusted Life Years)** | **Global** | **Female** | **55-59 years** | **Low back pain** | **Number** | **1994** | **2253401.78** | **3303988.16** | **1420230.67** |
| **DALYs (Disability-Adjusted Life Years)** | **Global** | **Female** | **55-59 years** | **Low back pain** | **Rate** | **1994** | **2285.73587** | **3351.39713** | **1440.60958** |
| **DALYs (Disability-Adjusted Life Years)** | **Global** | **Female** | **55-59 years** | **Low back pain** | **Number** | **1995** | **2280334.92** | **3343624.58** | **1438619.21** |
| **DALYs (Disability-Adjusted Life Years)** | **Global** | **Female** | **55-59 years** | **Low back pain** | **Rate** | **1995** | **2280.51216** | **3343.88446** | **1438.73102** |
| **DALYs (Disability-Adjusted Life Years)** | **Global** | **Female** | **55-59 years** | **Low back pain** | **Number** | **1996** | **2297175.77** | **3356538.93** | **1451307.07** |
| **DALYs (Disability-Adjusted Life Years)** | **Global** | **Female** | **55-59 years** | **Low back pain** | **Rate** | **1996** | **2275.44589** | **3324.78811** | **1437.57859** |
| **DALYs (Disability-Adjusted Life Years)** | **Global** | **Female** | **55-59 years** | **Low back pain** | **Number** | **1997** | **2299672.83** | **3361012.46** | **1449559.76** |
| **DALYs (Disability-Adjusted Life Years)** | **Global** | **Female** | **55-59 years** | **Low back pain** | **Rate** | **1997** | **2265.44498** | **3310.98785** | **1427.98481** |
| **DALYs (Disability-Adjusted Life Years)** | **Global** | **Female** | **55-59 years** | **Low back pain** | **Number** | **1998** | **2290476.75** | **3347664.23** | **1446533.79** |
| **DALYs (Disability-Adjusted Life Years)** | **Global** | **Female** | **55-59 years** | **Low back pain** | **Rate** | **1998** | **2249.9388** | **3288.41567** | **1420.93234** |
| **DALYs (Disability-Adjusted Life Years)** | **Global** | **Female** | **55-59 years** | **Low back pain** | **Number** | **1999** | **2283959.65** | **3343173.4** | **1444483.17** |
| **DALYs (Disability-Adjusted Life Years)** | **Global** | **Female** | **55-59 years** | **Low back pain** | **Rate** | **1999** | **2233.33961** | **3269.07771** | **1412.46868** |
| **DALYs (Disability-Adjusted Life Years)** | **Global** | **Female** | **55-59 years** | **Low back pain** | **Number** | **2000** | **2275550.65** | **3320207.16** | **1439134.11** |
| **DALYs (Disability-Adjusted Life Years)** | **Global** | **Female** | **55-59 years** | **Low back pain** | **Rate** | **2000** | **2217.67124** | **3235.75654** | **1402.52923** |
| **DALYs (Disability-Adjusted Life Years)** | **Global** | **Female** | **55-59 years** | **Low back pain** | **Number** | **2001** | **2297731.27** | **3353150.19** | **1452437.16** |
| **DALYs (Disability-Adjusted Life Years)** | **Global** | **Female** | **55-59 years** | **Low back pain** | **Rate** | **2001** | **2205.98575** | **3219.26312** | **1394.44317** |
| **DALYs (Disability-Adjusted Life Years)** | **Global** | **Female** | **55-59 years** | **Low back pain** | **Number** | **2002** | **2386692.81** | **3490169.74** | **1512894.72** |
| **DALYs (Disability-Adjusted Life Years)** | **Global** | **Female** | **55-59 years** | **Low back pain** | **Rate** | **2002** | **2204.18894** | **3223.286** | **1397.2078** |
| **DALYs (Disability-Adjusted Life Years)** | **Global** | **Female** | **55-59 years** | **Low back pain** | **Number** | **2003** | **2490724.8** | **3636275.33** | **1575371.4** |
| **DALYs (Disability-Adjusted Life Years)** | **Global** | **Female** | **55-59 years** | **Low back pain** | **Rate** | **2003** | **2206.92847** | **3221.95354** | **1395.87159** |
| **DALYs (Disability-Adjusted Life Years)** | **Global** | **Female** | **55-59 years** | **Low back pain** | **Number** | **2004** | **2630051.73** | **3845335.64** | **1661709.53** |
| **DALYs (Disability-Adjusted Life Years)** | **Global** | **Female** | **55-59 years** | **Low back pain** | **Rate** | **2004** | **2206.03758** | **3225.39471** | **1393.81048** |
| **DALYs (Disability-Adjusted Life Years)** | **Global** | **Female** | **55-59 years** | **Low back pain** | **Number** | **2005** | **2756950.31** | **4037329.25** | **1739482.95** |
| **DALYs (Disability-Adjusted Life Years)** | **Global** | **Female** | **55-59 years** | **Low back pain** | **Rate** | **2005** | **2210.45376** | **3237.02955** | **1394.67389** |
| **DALYs (Disability-Adjusted Life Years)** | **Global** | **Female** | **55-59 years** | **Low back pain** | **Number** | **2006** | **2901664.43** | **4254878.34** | **1831779.21** |
| **DALYs (Disability-Adjusted Life Years)** | **Global** | **Female** | **55-59 years** | **Low back pain** | **Rate** | **2006** | **2207.01045** | **3236.26704** | **1393.2541** |
| **DALYs (Disability-Adjusted Life Years)** | **Global** | **Female** | **55-59 years** | **Low back pain** | **Number** | **2007** | **2996698.16** | **4388854.67** | **1893842.64** |
| **DALYs (Disability-Adjusted Life Years)** | **Global** | **Female** | **55-59 years** | **Low back pain** | **Rate** | **2007** | **2196.62768** | **3217.10066** | **1388.21694** |
| **DALYs (Disability-Adjusted Life Years)** | **Global** | **Female** | **55-59 years** | **Low back pain** | **Number** | **2008** | **3099691.43** | **4532076.12** | **1959324.23** |
| **DALYs (Disability-Adjusted Life Years)** | **Global** | **Female** | **55-59 years** | **Low back pain** | **Rate** | **2008** | **2183.57432** | **3192.61618** | **1380.24386** |
| **DALYs (Disability-Adjusted Life Years)** | **Global** | **Female** | **55-59 years** | **Low back pain** | **Number** | **2009** | **3184823.72** | **4656788.12** | **2012447.81** |
| **DALYs (Disability-Adjusted Life Years)** | **Global** | **Female** | **55-59 years** | **Low back pain** | **Rate** | **2009** | **2173.09363** | **3177.45579** | **1373.149** |
| **DALYs (Disability-Adjusted Life Years)** | **Global** | **Female** | **55-59 years** | **Low back pain** | **Number** | **2010** | **3301575.03** | **4824607.54** | **2087028.51** |
| **DALYs (Disability-Adjusted Life Years)** | **Global** | **Female** | **55-59 years** | **Low back pain** | **Rate** | **2010** | **2161.00012** | **3157.87991** | **1366.03555** |
| **DALYs (Disability-Adjusted Life Years)** | **Global** | **Female** | **55-59 years** | **Low back pain** | **Number** | **2011** | **3382518.01** | **4954666.63** | **2134013.94** |
| **DALYs (Disability-Adjusted Life Years)** | **Global** | **Female** | **55-59 years** | **Low back pain** | **Rate** | **2011** | **2156.94963** | **3159.47065** | **1360.8089** |
| **DALYs (Disability-Adjusted Life Years)** | **Global** | **Female** | **55-59 years** | **Low back pain** | **Number** | **2012** | **3454800.29** | **5051761.38** | **2178481.69** |
| **DALYs (Disability-Adjusted Life Years)** | **Global** | **Female** | **55-59 years** | **Low back pain** | **Rate** | **2012** | **2155.96024** | **3152.54017** | **1359.47654** |
| **DALYs (Disability-Adjusted Life Years)** | **Global** | **Female** | **55-59 years** | **Low back pain** | **Number** | **2013** | **3515198.91** | **5146430.88** | **2214667.41** |
| **DALYs (Disability-Adjusted Life Years)** | **Global** | **Female** | **55-59 years** | **Low back pain** | **Rate** | **2013** | **2159.95741** | **3162.28805** | **1360.82976** |
| **DALYs (Disability-Adjusted Life Years)** | **Global** | **Female** | **55-59 years** | **Low back pain** | **Number** | **2014** | **3566083.6** | **5228160.83** | **2246306.67** |
| **DALYs (Disability-Adjusted Life Years)** | **Global** | **Female** | **55-59 years** | **Low back pain** | **Rate** | **2014** | **2165.88593** | **3175.36021** | **1364.31014** |
| **DALYs (Disability-Adjusted Life Years)** | **Global** | **Female** | **55-59 years** | **Low back pain** | **Number** | **2015** | **3615211.19** | **5299226.31** | **2279602.98** |
| **DALYs (Disability-Adjusted Life Years)** | **Global** | **Female** | **55-59 years** | **Low back pain** | **Rate** | **2015** | **2169.92265** | **3180.70249** | **1368.26367** |
| **DALYs (Disability-Adjusted Life Years)** | **Global** | **Female** | **55-59 years** | **Low back pain** | **Number** | **2016** | **3677481.31** | **5377256.32** | **2321743.58** |
| **DALYs (Disability-Adjusted Life Years)** | **Global** | **Female** | **55-59 years** | **Low back pain** | **Rate** | **2016** | **2168.64579** | **3171.01932** | **1369.15432** |
| **DALYs (Disability-Adjusted Life Years)** | **Global** | **Female** | **55-59 years** | **Low back pain** | **Number** | **2017** | **3761869.03** | **5507461** | **2363755.74** |
| **DALYs (Disability-Adjusted Life Years)** | **Global** | **Female** | **55-59 years** | **Low back pain** | **Rate** | **2017** | **2161.00847** | **3163.76508** | **1357.86125** |
| **DALYs (Disability-Adjusted Life Years)** | **Global** | **Female** | **55-59 years** | **Low back pain** | **Number** | **2018** | **3875152.8** | **5660441.1** | **2435207.2** |
| **DALYs (Disability-Adjusted Life Years)** | **Global** | **Female** | **55-59 years** | **Low back pain** | **Rate** | **2018** | **2146.94095** | **3136.03964** | **1349.17159** |
| **DALYs (Disability-Adjusted Life Years)** | **Global** | **Female** | **55-59 years** | **Low back pain** | **Number** | **2019** | **4002700.13** | **5856370.28** | **2507923.55** |
| **DALYs (Disability-Adjusted Life Years)** | **Global** | **Female** | **55-59 years** | **Low back pain** | **Rate** | **2019** | **2131.4684** | **3118.56192** | **1335.48845** |
| **DALYs (Disability-Adjusted Life Years)** | **Global** | **Female** | **55-59 years** | **Low back pain** | **Number** | **2020** | **4113470.43** | **6032450.46** | **2586232.13** |
| **DALYs (Disability-Adjusted Life Years)** | **Global** | **Female** | **55-59 years** | **Low back pain** | **Rate** | **2020** | **2110.28957** | **3094.76329** | **1326.7869** |
| **DALYs (Disability-Adjusted Life Years)** | **Global** | **Female** | **55-59 years** | **Low back pain** | **Number** | **2021** | **4215179.67** | **6184007.64** | **2646254.37** |
| **DALYs (Disability-Adjusted Life Years)** | **Global** | **Female** | **55-59 years** | **Low back pain** | **Rate** | **2021** | **2097.06755** | **3076.56678** | **1316.52138** |
| **Prevalence** | **Global** | **Female** | **55-59 years** | **Low back pain** | **Number** | **1990** | **19412403** | **25811026.8** | **14192961.2** |
| **Prevalence** | **Global** | **Female** | **55-59 years** | **Low back pain** | **Rate** | **1990** | **21029.1392** | **27960.6639** | **15375.0032** |
| **Prevalence** | **Global** | **Female** | **55-59 years** | **Low back pain** | **Number** | **1991** | **19432468.8** | **25814885.5** | **14230088.5** |
| **Prevalence** | **Global** | **Female** | **55-59 years** | **Low back pain** | **Rate** | **1991** | **20741.5143** | **27553.875** | **15188.6817** |
| **Prevalence** | **Global** | **Female** | **55-59 years** | **Low back pain** | **Number** | **1992** | **19580016.4** | **25984248.6** | **14363092.3** |
| **Prevalence** | **Global** | **Female** | **55-59 years** | **Low back pain** | **Rate** | **1992** | **20514.9407** | **27224.9679** | **15048.9141** |
| **Prevalence** | **Global** | **Female** | **55-59 years** | **Low back pain** | **Number** | **1993** | **19761816.5** | **26196649.8** | **14521496.2** |
| **Prevalence** | **Global** | **Female** | **55-59 years** | **Low back pain** | **Rate** | **1993** | **20348.9724** | **26974.9951** | **14952.9536** |
| **Prevalence** | **Global** | **Female** | **55-59 years** | **Low back pain** | **Number** | **1994** | **19970309.8** | **26442386.7** | **14699952.5** |
| **Prevalence** | **Global** | **Female** | **55-59 years** | **Low back pain** | **Rate** | **1994** | **20256.864** | **26821.8088** | **14910.8824** |
| **Prevalence** | **Global** | **Female** | **55-59 years** | **Low back pain** | **Number** | **1995** | **20211081.3** | **26731715.1** | **14900318.4** |
| **Prevalence** | **Global** | **Female** | **55-59 years** | **Low back pain** | **Rate** | **1995** | **20212.6522** | **26733.7928** | **14901.4765** |
| **Prevalence** | **Global** | **Female** | **55-59 years** | **Low back pain** | **Number** | **1996** | **20362436.7** | **26904670.3** | **15020119.3** |
| **Prevalence** | **Global** | **Female** | **55-59 years** | **Low back pain** | **Rate** | **1996** | **20169.8205** | **26650.1685** | **14878.0381** |
| **Prevalence** | **Global** | **Female** | **55-59 years** | **Low back pain** | **Number** | **1997** | **20384842.8** | **26918559.9** | **15046068.2** |
| **Prevalence** | **Global** | **Female** | **55-59 years** | **Low back pain** | **Rate** | **1997** | **20081.439** | **26517.9096** | **14822.1256** |
| **Prevalence** | **Global** | **Female** | **55-59 years** | **Low back pain** | **Number** | **1998** | **20305784.2** | **26803071.5** | **15007342** |
| **Prevalence** | **Global** | **Female** | **55-59 years** | **Low back pain** | **Rate** | **1998** | **19946.4028** | **26328.6979** | **14741.7349** |
| **Prevalence** | **Global** | **Female** | **55-59 years** | **Low back pain** | **Number** | **1999** | **20247465.6** | **26720345.7** | **14996804.9** |
| **Prevalence** | **Global** | **Female** | **55-59 years** | **Low back pain** | **Rate** | **1999** | **19798.7154** | **26128.1352** | **14664.4265** |
| **Prevalence** | **Global** | **Female** | **55-59 years** | **Low back pain** | **Number** | **2000** | **20168421.7** | **26615850** | **14970323.3** |
| **Prevalence** | **Global** | **Female** | **55-59 years** | **Low back pain** | **Rate** | **2000** | **19655.431** | **25938.8667** | **14589.5479** |
| **Prevalence** | **Global** | **Female** | **55-59 years** | **Low back pain** | **Number** | **2001** | **20362692.6** | **26883132.6** | **15131017.3** |
| **Prevalence** | **Global** | **Female** | **55-59 years** | **Low back pain** | **Rate** | **2001** | **19549.6359** | **25809.7229** | **14526.8548** |
| **Prevalence** | **Global** | **Female** | **55-59 years** | **Low back pain** | **Number** | **2002** | **21144698.8** | **27912535.2** | **15733803.3** |
| **Prevalence** | **Global** | **Female** | **55-59 years** | **Low back pain** | **Rate** | **2002** | **19527.8215** | **25778.1399** | **14530.6823** |
| **Prevalence** | **Global** | **Female** | **55-59 years** | **Low back pain** | **Number** | **2003** | **22064000.9** | **29117610.6** | **16434168** |
| **Prevalence** | **Global** | **Female** | **55-59 years** | **Low back pain** | **Rate** | **2003** | **19550.0009** | **25799.9134** | **14561.6382** |
| **Prevalence** | **Global** | **Female** | **55-59 years** | **Low back pain** | **Number** | **2004** | **23296981.3** | **30740573.4** | **17347698.2** |
| **Prevalence** | **Global** | **Female** | **55-59 years** | **Low back pain** | **Rate** | **2004** | **19541.0667** | **25784.6108** | **14550.9207** |
| **Prevalence** | **Global** | **Female** | **55-59 years** | **Low back pain** | **Number** | **2005** | **24418086.9** | **32211436.4** | **18181279.1** |
| **Prevalence** | **Global** | **Female** | **55-59 years** | **Low back pain** | **Rate** | **2005** | **19577.8109** | **25826.3235** | **14577.2945** |
| **Prevalence** | **Global** | **Female** | **55-59 years** | **Low back pain** | **Number** | **2006** | **25697595.6** | **33916092.1** | **19141663** |
| **Prevalence** | **Global** | **Female** | **55-59 years** | **Low back pain** | **Rate** | **2006** | **19545.6309** | **25796.6321** | **14559.1784** |
| **Prevalence** | **Global** | **Female** | **55-59 years** | **Low back pain** | **Number** | **2007** | **26535792.3** | **35046424.3** | **19765563.8** |
| **Prevalence** | **Global** | **Female** | **55-59 years** | **Low back pain** | **Rate** | **2007** | **19451.1601** | **25689.5895** | **14488.4743** |
| **Prevalence** | **Global** | **Female** | **55-59 years** | **Low back pain** | **Number** | **2008** | **27440089.8** | **36266434** | **20431587.1** |
| **Prevalence** | **Global** | **Female** | **55-59 years** | **Low back pain** | **Rate** | **2008** | **19330.142** | **25547.8507** | **14393.0097** |
| **Prevalence** | **Global** | **Female** | **55-59 years** | **Low back pain** | **Number** | **2009** | **28193882.8** | **37281934.9** | **20987330.4** |
| **Prevalence** | **Global** | **Female** | **55-59 years** | **Low back pain** | **Rate** | **2009** | **19237.4688** | **25438.499** | **14320.2381** |
| **Prevalence** | **Global** | **Female** | **55-59 years** | **Low back pain** | **Number** | **2010** | **29226742.9** | **38675790.3** | **21748649.4** |
| **Prevalence** | **Global** | **Female** | **55-59 years** | **Low back pain** | **Rate** | **2010** | **19129.959** | **25314.7018** | **14235.2766** |
| **Prevalence** | **Global** | **Female** | **55-59 years** | **Low back pain** | **Number** | **2011** | **29935455.3** | **39587170** | **22263596.8** |
| **Prevalence** | **Global** | **Female** | **55-59 years** | **Low back pain** | **Rate** | **2011** | **19089.1132** | **25243.7775** | **14196.9553** |
| **Prevalence** | **Global** | **Female** | **55-59 years** | **Low back pain** | **Number** | **2012** | **30583134.6** | **40411716.4** | **22729966.9** |
| **Prevalence** | **Global** | **Female** | **55-59 years** | **Low back pain** | **Rate** | **2012** | **19085.3355** | **25218.8395** | **14184.584** |
| **Prevalence** | **Global** | **Female** | **55-59 years** | **Low back pain** | **Number** | **2013** | **31125112.7** | **41087960.6** | **23125597.5** |
| **Prevalence** | **Global** | **Female** | **55-59 years** | **Low back pain** | **Rate** | **2013** | **19125.2101** | **25247.0052** | **14209.8092** |
| **Prevalence** | **Global** | **Female** | **55-59 years** | **Low back pain** | **Number** | **2014** | **31583476.6** | **41650431.7** | **23449865.8** |
| **Prevalence** | **Global** | **Female** | **55-59 years** | **Low back pain** | **Rate** | **2014** | **19182.4464** | **25296.6823** | **14242.4408** |
| **Prevalence** | **Global** | **Female** | **55-59 years** | **Low back pain** | **Number** | **2015** | **32026906.6** | **42211109.5** | **23763696.2** |
| **Prevalence** | **Global** | **Female** | **55-59 years** | **Low back pain** | **Rate** | **2015** | **19223.1952** | **25335.9591** | **14263.4496** |
| **Prevalence** | **Global** | **Female** | **55-59 years** | **Low back pain** | **Number** | **2016** | **32584011.6** | **42989862.2** | **24154828.1** |
| **Prevalence** | **Global** | **Female** | **55-59 years** | **Low back pain** | **Rate** | **2016** | **19215.1023** | **25351.5316** | **14244.3324** |
| **Prevalence** | **Global** | **Female** | **55-59 years** | **Low back pain** | **Number** | **2017** | **33328917.9** | **44054202.5** | **24654724.5** |
| **Prevalence** | **Global** | **Female** | **55-59 years** | **Low back pain** | **Rate** | **2017** | **19145.8217** | **25306.9695** | **14162.9249** |
| **Prevalence** | **Global** | **Female** | **55-59 years** | **Low back pain** | **Number** | **2018** | **34330855.2** | **45471462.7** | **25328909.1** |
| **Prevalence** | **Global** | **Female** | **55-59 years** | **Low back pain** | **Rate** | **2018** | **19020.2355** | **25192.4376** | **14032.9104** |
| **Prevalence** | **Global** | **Female** | **55-59 years** | **Low back pain** | **Number** | **2019** | **35460118.8** | **47010569.3** | **26095240.2** |
| **Prevalence** | **Global** | **Female** | **55-59 years** | **Low back pain** | **Rate** | **2019** | **18882.7842** | **25033.4873** | **13895.9148** |
| **Prevalence** | **Global** | **Female** | **55-59 years** | **Low back pain** | **Number** | **2020** | **36499622** | **48532126.3** | **26975119.1** |
| **Prevalence** | **Global** | **Female** | **55-59 years** | **Low back pain** | **Rate** | **2020** | **18725.0092** | **24897.9157** | **13838.7557** |
| **Prevalence** | **Global** | **Female** | **55-59 years** | **Low back pain** | **Number** | **2021** | **37448900.7** | **49708719.7** | **27533466.2** |
| **Prevalence** | **Global** | **Female** | **55-59 years** | **Low back pain** | **Rate** | **2021** | **18630.9672** | **24730.2727** | **13698.0018** |
| **Incidence** | **Global** | **Female** | **55-59 years** | **Low back pain** | **Number** | **1990** | **7697430.3** | **10349743.2** | **5502739.96** |
| **Incidence** | **Global** | **Female** | **55-59 years** | **Low back pain** | **Rate** | **1990** | **8338.50055** | **11211.7078** | **5961.02834** |
| **Incidence** | **Global** | **Female** | **55-59 years** | **Low back pain** | **Number** | **1991** | **7727656.63** | **10389496.6** | **5535878.5** |
| **Incidence** | **Global** | **Female** | **55-59 years** | **Low back pain** | **Rate** | **1991** | **8248.22114** | **11089.3729** | **5908.79645** |
| **Incidence** | **Global** | **Female** | **55-59 years** | **Low back pain** | **Number** | **1992** | **7805349.54** | **10492326.5** | **5603155.92** |
| **Incidence** | **Global** | **Female** | **55-59 years** | **Low back pain** | **Rate** | **1992** | **8178.0464** | **10993.3235** | **5870.70045** |
| **Incidence** | **Global** | **Female** | **55-59 years** | **Low back pain** | **Number** | **1993** | **7892166.4** | **10608014** | **5670728.85** |
| **Incidence** | **Global** | **Female** | **55-59 years** | **Low back pain** | **Rate** | **1993** | **8126.65558** | **10923.1954** | **5839.21548** |
| **Incidence** | **Global** | **Female** | **55-59 years** | **Low back pain** | **Number** | **1994** | **7983794.73** | **10729698.8** | **5738383.96** |
| **Incidence** | **Global** | **Female** | **55-59 years** | **Low back pain** | **Rate** | **1994** | **8098.35431** | **10883.6594** | **5820.72411** |
| **Incidence** | **Global** | **Female** | **55-59 years** | **Low back pain** | **Number** | **1995** | **8082414.73** | **10860420.2** | **5810058.81** |
| **Incidence** | **Global** | **Female** | **55-59 years** | **Low back pain** | **Rate** | **1995** | **8083.04292** | **10861.2643** | **5810.51039** |
| **Incidence** | **Global** | **Female** | **55-59 years** | **Low back pain** | **Number** | **1996** | **8143194.78** | **10933771.8** | **5858244.64** |
| **Incidence** | **Global** | **Female** | **55-59 years** | **Low back pain** | **Rate** | **1996** | **8066.16511** | **10830.345** | **5802.82921** |
| **Incidence** | **Global** | **Female** | **55-59 years** | **Low back pain** | **Number** | **1997** | **8154970.64** | **10942063.1** | **5870193.99** |
| **Incidence** | **Global** | **Female** | **55-59 years** | **Low back pain** | **Rate** | **1997** | **8033.59374** | **10779.2037** | **5782.82323** |
| **Incidence** | **Global** | **Female** | **55-59 years** | **Low back pain** | **Number** | **1998** | **8128802.91** | **10899968.5** | **5854652.85** |
| **Incidence** | **Global** | **Female** | **55-59 years** | **Low back pain** | **Rate** | **1998** | **7984.93547** | **10707.0556** | **5751.03441** |
| **Incidence** | **Global** | **Female** | **55-59 years** | **Low back pain** | **Number** | **1999** | **8111993.41** | **10872300.7** | **5843548.91** |
| **Incidence** | **Global** | **Female** | **55-59 years** | **Low back pain** | **Rate** | **1999** | **7932.20503** | **10631.3348** | **5714.0367** |
| **Incidence** | **Global** | **Female** | **55-59 years** | **Low back pain** | **Number** | **2000** | **8088357.04** | **10837933.4** | **5824698.44** |
| **Incidence** | **Global** | **Female** | **55-59 years** | **Low back pain** | **Rate** | **2000** | **7882.62689** | **10562.2668** | **5676.54522** |
| **Incidence** | **Global** | **Female** | **55-59 years** | **Low back pain** | **Number** | **2001** | **8173471.34** | **10940080.3** | **5881251.54** |
| **Incidence** | **Global** | **Female** | **55-59 years** | **Low back pain** | **Rate** | **2001** | **7847.11491** | **10503.2566** | **5646.42056** |
| **Incidence** | **Global** | **Female** | **55-59 years** | **Low back pain** | **Number** | **2002** | **8492446.3** | **11351168** | **6107996.23** |
| **Incidence** | **Global** | **Female** | **55-59 years** | **Low back pain** | **Rate** | **2002** | **7843.05215** | **10483.1752** | **5640.93446** |
| **Incidence** | **Global** | **Female** | **55-59 years** | **Low back pain** | **Number** | **2003** | **8863846.06** | **11835463.8** | **6374014.99** |
| **Incidence** | **Global** | **Female** | **55-59 years** | **Low back pain** | **Rate** | **2003** | **7853.88827** | **10486.9161** | **5647.75169** |
| **Incidence** | **Global** | **Female** | **55-59 years** | **Low back pain** | **Number** | **2004** | **9363511.12** | **12500786.3** | **6732260.61** |
| **Incidence** | **Global** | **Female** | **55-59 years** | **Low back pain** | **Rate** | **2004** | **7853.9358** | **10485.4228** | **5646.89271** |
| **Incidence** | **Global** | **Female** | **55-59 years** | **Low back pain** | **Number** | **2005** | **9813442.87** | **13097981.9** | **7055313.71** |
| **Incidence** | **Global** | **Female** | **55-59 years** | **Low back pain** | **Rate** | **2005** | **7868.17288** | **10501.6341** | **5656.77395** |
| **Incidence** | **Global** | **Female** | **55-59 years** | **Low back pain** | **Number** | **2006** | **10329043.7** | **13792168.8** | **7424423.14** |
| **Incidence** | **Global** | **Female** | **55-59 years** | **Low back pain** | **Rate** | **2006** | **7856.28661** | **10490.3449** | **5647.02771** |
| **Incidence** | **Global** | **Female** | **55-59 years** | **Low back pain** | **Number** | **2007** | **10667343.4** | **14253448.1** | **7663171.5** |
| **Incidence** | **Global** | **Female** | **55-59 years** | **Low back pain** | **Rate** | **2007** | **7819.33335** | **10448.0054** | **5617.22727** |
| **Incidence** | **Global** | **Female** | **55-59 years** | **Low back pain** | **Number** | **2008** | **11034688.6** | **14763256.2** | **7921219.26** |
| **Incidence** | **Global** | **Female** | **55-59 years** | **Low back pain** | **Rate** | **2008** | **7773.37457** | **10399.96** | **5580.09445** |
| **Incidence** | **Global** | **Female** | **55-59 years** | **Low back pain** | **Number** | **2009** | **11340652.3** | **15190656.9** | **8136251.34** |
| **Incidence** | **Global** | **Female** | **55-59 years** | **Low back pain** | **Rate** | **2009** | **7738.04186** | **10365.0069** | **5551.59012** |
| **Incidence** | **Global** | **Female** | **55-59 years** | **Low back pain** | **Number** | **2010** | **11764896.2** | **15774564.5** | **8436885.13** |
| **Incidence** | **Global** | **Female** | **55-59 years** | **Low back pain** | **Rate** | **2010** | **7700.54953** | **10325.0223** | **5522.24608** |
| **Incidence** | **Global** | **Female** | **55-59 years** | **Low back pain** | **Number** | **2011** | **12057461.6** | **16159807.5** | **8649996.12** |
| **Incidence** | **Global** | **Female** | **55-59 years** | **Low back pain** | **Rate** | **2011** | **7688.75059** | **10304.717** | **5515.89258** |
| **Incidence** | **Global** | **Female** | **55-59 years** | **Low back pain** | **Number** | **2012** | **12324933.8** | **16500317.5** | **8846894.58** |
| **Incidence** | **Global** | **Female** | **55-59 years** | **Low back pain** | **Rate** | **2012** | **7691.34687** | **10296.9855** | **5520.8844** |
| **Incidence** | **Global** | **Female** | **55-59 years** | **Low back pain** | **Number** | **2013** | **12546640.2** | **16778518.7** | **9013306.39** |
| **Incidence** | **Global** | **Female** | **55-59 years** | **Low back pain** | **Rate** | **2013** | **7709.43812** | **10309.7681** | **5538.3375** |
| **Incidence** | **Global** | **Female** | **55-59 years** | **Low back pain** | **Number** | **2014** | **12732231.5** | **17009879.8** | **9154447.19** |
| **Incidence** | **Global** | **Female** | **55-59 years** | **Low back pain** | **Rate** | **2014** | **7733.01026** | **10331.07** | **5560.01782** |
| **Incidence** | **Global** | **Female** | **55-59 years** | **Low back pain** | **Number** | **2015** | **12911455** | **17233974** | **9289724.85** |
| **Incidence** | **Global** | **Female** | **55-59 years** | **Low back pain** | **Rate** | **2015** | **7749.71567** | **10344.1787** | **5575.88018** |
| **Incidence** | **Global** | **Female** | **55-59 years** | **Low back pain** | **Number** | **2016** | **13137766.7** | **17545153.8** | **9449468.15** |
| **Incidence** | **Global** | **Female** | **55-59 years** | **Low back pain** | **Rate** | **2016** | **7747.46628** | **10346.5445** | **5572.44146** |
| **Incidence** | **Global** | **Female** | **55-59 years** | **Low back pain** | **Number** | **2017** | **13443497.9** | **17970617.9** | **9659333.78** |
| **Incidence** | **Global** | **Female** | **55-59 years** | **Low back pain** | **Rate** | **2017** | **7722.62742** | **10323.2349** | **5548.81149** |
| **Incidence** | **Global** | **Female** | **55-59 years** | **Low back pain** | **Number** | **2018** | **13859136.5** | **18550690.5** | **9943426.27** |
| **Incidence** | **Global** | **Female** | **55-59 years** | **Low back pain** | **Rate** | **2018** | **7678.34178** | **10277.5914** | **5508.93091** |
| **Incidence** | **Global** | **Female** | **55-59 years** | **Low back pain** | **Number** | **2019** | **14330355.9** | **19205597.1** | **10268561.1** |
| **Incidence** | **Global** | **Female** | **55-59 years** | **Low back pain** | **Rate** | **2019** | **7631.02399** | **10227.1272** | **5468.08724** |
| **Incidence** | **Global** | **Female** | **55-59 years** | **Low back pain** | **Number** | **2020** | **14772963** | **19801982.9** | **10574062.5** |
| **Incidence** | **Global** | **Female** | **55-59 years** | **Low back pain** | **Rate** | **2020** | **7578.81458** | **10158.7987** | **5424.69776** |
| **Incidence** | **Global** | **Female** | **55-59 years** | **Low back pain** | **Number** | **2021** | **15158767.3** | **20279983.5** | **10836956.9** |
| **Incidence** | **Global** | **Female** | **55-59 years** | **Low back pain** | **Rate** | **2021** | **7541.54308** | **10089.3671** | **5391.42634** |
| **DALYs (Disability-Adjusted Life Years)** | **Global** | **Female** | **60-64 years** | **Low back pain** | **Number** | **1990** | **2106893.3** | **3130968.91** | **1314760.17** |
| **DALYs (Disability-Adjusted Life Years)** | **Global** | **Female** | **60-64 years** | **Low back pain** | **Rate** | **1990** | **2567.40555** | **3815.31753** | **1602.13266** |
| **DALYs (Disability-Adjusted Life Years)** | **Global** | **Female** | **60-64 years** | **Low back pain** | **Number** | **1992** | **2115369.73** | **3154456.59** | **1326912.73** |
| **DALYs (Disability-Adjusted Life Years)** | **Global** | **Female** | **60-64 years** | **Low back pain** | **Rate** | **1992** | **2503.85202** | **3733.76455** | **1570.5969** |
| **DALYs (Disability-Adjusted Life Years)** | **Global** | **Female** | **60-64 years** | **Low back pain** | **Number** | **1991** | **2116830.56** | **3144909.88** | **1323593.74** |
| **DALYs (Disability-Adjusted Life Years)** | **Global** | **Female** | **60-64 years** | **Low back pain** | **Rate** | **1991** | **2536.54921** | **3768.47293** | **1586.03184** |
| **DALYs (Disability-Adjusted Life Years)** | **Global** | **Female** | **60-64 years** | **Low back pain** | **Number** | **1993** | **2114782.76** | **3142384.44** | **1328703.52** |
| **DALYs (Disability-Adjusted Life Years)** | **Global** | **Female** | **60-64 years** | **Low back pain** | **Rate** | **1993** | **2474.51449** | **3676.9147** | **1554.7205** |
| **DALYs (Disability-Adjusted Life Years)** | **Global** | **Female** | **60-64 years** | **Low back pain** | **Number** | **1994** | **2113271.41** | **3140294.36** | **1335376.69** |
| **DALYs (Disability-Adjusted Life Years)** | **Global** | **Female** | **60-64 years** | **Low back pain** | **Rate** | **1994** | **2448.68974** | **3638.72172** | **1547.32761** |
| **DALYs (Disability-Adjusted Life Years)** | **Global** | **Female** | **60-64 years** | **Low back pain** | **Number** | **1995** | **2125970.33** | **3156643.57** | **1343420.62** |
| **DALYs (Disability-Adjusted Life Years)** | **Global** | **Female** | **60-64 years** | **Low back pain** | **Rate** | **1995** | **2434.0766** | **3614.12017** | **1538.11587** |
| **DALYs (Disability-Adjusted Life Years)** | **Global** | **Female** | **60-64 years** | **Low back pain** | **Number** | **1996** | **2152448.23** | **3190750.11** | **1360480.91** |
| **DALYs (Disability-Adjusted Life Years)** | **Global** | **Female** | **60-64 years** | **Low back pain** | **Rate** | **1996** | **2425.55608** | **3595.60022** | **1533.10202** |
| **DALYs (Disability-Adjusted Life Years)** | **Global** | **Female** | **60-64 years** | **Low back pain** | **Number** | **1997** | **2192167.92** | **3257860.68** | **1384813.42** |
| **DALYs (Disability-Adjusted Life Years)** | **Global** | **Female** | **60-64 years** | **Low back pain** | **Rate** | **1997** | **2421.10777** | **3598.09653** | **1529.4369** |
| **DALYs (Disability-Adjusted Life Years)** | **Global** | **Female** | **60-64 years** | **Low back pain** | **Number** | **1998** | **2232645.32** | **3309953.23** | **1409232.06** |
| **DALYs (Disability-Adjusted Life Years)** | **Global** | **Female** | **60-64 years** | **Low back pain** | **Rate** | **1998** | **2419.16676** | **3586.47599** | **1526.96325** |
| **DALYs (Disability-Adjusted Life Years)** | **Global** | **Female** | **60-64 years** | **Low back pain** | **Number** | **1999** | **2271239** | **3362358.88** | **1437758.53** |
| **DALYs (Disability-Adjusted Life Years)** | **Global** | **Female** | **60-64 years** | **Low back pain** | **Rate** | **1999** | **2419.85959** | **3582.37788** | **1531.83957** |
| **DALYs (Disability-Adjusted Life Years)** | **Global** | **Female** | **60-64 years** | **Low back pain** | **Number** | **2000** | **2308009.24** | **3419330.91** | **1457647.41** |
| **DALYs (Disability-Adjusted Life Years)** | **Global** | **Female** | **60-64 years** | **Low back pain** | **Rate** | **2000** | **2420.13534** | **3585.44647** | **1528.46182** |
| **DALYs (Disability-Adjusted Life Years)** | **Global** | **Female** | **60-64 years** | **Low back pain** | **Number** | **2001** | **2331830.1** | **3454280.31** | **1472995.89** |
| **DALYs (Disability-Adjusted Life Years)** | **Global** | **Female** | **60-64 years** | **Low back pain** | **Rate** | **2001** | **2417.70897** | **3581.49785** | **1527.24479** |
| **DALYs (Disability-Adjusted Life Years)** | **Global** | **Female** | **60-64 years** | **Low back pain** | **Number** | **2002** | **2341814.26** | **3455149.19** | **1486417.02** |
| **DALYs (Disability-Adjusted Life Years)** | **Global** | **Female** | **60-64 years** | **Low back pain** | **Rate** | **2002** | **2411.20196** | **3557.52487** | **1530.45939** |
| **DALYs (Disability-Adjusted Life Years)** | **Global** | **Female** | **60-64 years** | **Low back pain** | **Number** | **2003** | **2341289.55** | **3455621.81** | **1484701.71** |
| **DALYs (Disability-Adjusted Life Years)** | **Global** | **Female** | **60-64 years** | **Low back pain** | **Rate** | **2003** | **2399.28279** | **3541.21683** | **1521.47746** |
| **DALYs (Disability-Adjusted Life Years)** | **Global** | **Female** | **60-64 years** | **Low back pain** | **Number** | **2004** | **2343970.21** | **3448993.86** | **1489587.28** |
| **DALYs (Disability-Adjusted Life Years)** | **Global** | **Female** | **60-64 years** | **Low back pain** | **Rate** | **2004** | **2385.37608** | **3509.9198** | **1515.90061** |
| **DALYs (Disability-Adjusted Life Years)** | **Global** | **Female** | **60-64 years** | **Low back pain** | **Number** | **2005** | **2342934.8** | **3442934.69** | **1493280.02** |
| **DALYs (Disability-Adjusted Life Years)** | **Global** | **Female** | **60-64 years** | **Low back pain** | **Rate** | **2005** | **2369.6053** | **3482.1269** | **1510.27858** |
| **DALYs (Disability-Adjusted Life Years)** | **Global** | **Female** | **60-64 years** | **Low back pain** | **Number** | **2006** | **2370224.74** | **3490597.87** | **1511926.29** |
| **DALYs (Disability-Adjusted Life Years)** | **Global** | **Female** | **60-64 years** | **Low back pain** | **Rate** | **2006** | **2355.22565** | **3468.50892** | **1502.35863** |
| **DALYs (Disability-Adjusted Life Years)** | **Global** | **Female** | **60-64 years** | **Low back pain** | **Number** | **2007** | **2465296.77** | **3624411.89** | **1570736.5** |
| **DALYs (Disability-Adjusted Life Years)** | **Global** | **Female** | **60-64 years** | **Low back pain** | **Rate** | **2007** | **2348.8963** | **3453.2831** | **1496.57323** |
| **DALYs (Disability-Adjusted Life Years)** | **Global** | **Female** | **60-64 years** | **Low back pain** | **Number** | **2008** | **2573588.69** | **3796944.33** | **1634652.22** |
| **DALYs (Disability-Adjusted Life Years)** | **Global** | **Female** | **60-64 years** | **Low back pain** | **Rate** | **2008** | **2346.47251** | **3461.86844** | **1490.39608** |
| **DALYs (Disability-Adjusted Life Years)** | **Global** | **Female** | **60-64 years** | **Low back pain** | **Number** | **2009** | **2719418.44** | **4012069.89** | **1725846.76** |
| **DALYs (Disability-Adjusted Life Years)** | **Global** | **Female** | **60-64 years** | **Low back pain** | **Rate** | **2009** | **2342.37444** | **3455.80136** | **1486.56024** |
| **DALYs (Disability-Adjusted Life Years)** | **Global** | **Female** | **60-64 years** | **Low back pain** | **Number** | **2010** | **2852857.61** | **4209176.13** | **1811545.16** |
| **DALYs (Disability-Adjusted Life Years)** | **Global** | **Female** | **60-64 years** | **Low back pain** | **Rate** | **2010** | **2345.87905** | **3461.16752** | **1489.61723** |
| **DALYs (Disability-Adjusted Life Years)** | **Global** | **Female** | **60-64 years** | **Low back pain** | **Number** | **2011** | **3010481.94** | **4449757.08** | **1907682.54** |
| **DALYs (Disability-Adjusted Life Years)** | **Global** | **Female** | **60-64 years** | **Low back pain** | **Rate** | **2011** | **2345.705** | **3467.15828** | **1486.42661** |
| **DALYs (Disability-Adjusted Life Years)** | **Global** | **Female** | **60-64 years** | **Low back pain** | **Number** | **2012** | **3121222.09** | **4616136.06** | **1978950.09** |
| **DALYs (Disability-Adjusted Life Years)** | **Global** | **Female** | **60-64 years** | **Low back pain** | **Rate** | **2012** | **2342.55552** | **3464.52597** | **1485.25171** |
| **DALYs (Disability-Adjusted Life Years)** | **Global** | **Female** | **60-64 years** | **Low back pain** | **Number** | **2013** | **3240536.93** | **4783004.35** | **2057683.17** |
| **DALYs (Disability-Adjusted Life Years)** | **Global** | **Female** | **60-64 years** | **Low back pain** | **Rate** | **2013** | **2336.94371** | **3449.30861** | **1483.91759** |
| **DALYs (Disability-Adjusted Life Years)** | **Global** | **Female** | **60-64 years** | **Low back pain** | **Number** | **2014** | **3339941.34** | **4933429.92** | **2116447.52** |
| **DALYs (Disability-Adjusted Life Years)** | **Global** | **Female** | **60-64 years** | **Low back pain** | **Rate** | **2014** | **2333.16817** | **3446.3245** | **1478.47746** |
| **DALYs (Disability-Adjusted Life Years)** | **Global** | **Female** | **60-64 years** | **Low back pain** | **Number** | **2015** | **3467984.91** | **5115504.67** | **2194292.28** |
| **DALYs (Disability-Adjusted Life Years)** | **Global** | **Female** | **60-64 years** | **Low back pain** | **Rate** | **2015** | **2324.98725** | **3429.50834** | **1471.08529** |
| **DALYs (Disability-Adjusted Life Years)** | **Global** | **Female** | **60-64 years** | **Low back pain** | **Number** | **2016** | **3550001.16** | **5242339.46** | **2241960.68** |
| **DALYs (Disability-Adjusted Life Years)** | **Global** | **Female** | **60-64 years** | **Low back pain** | **Rate** | **2016** | **2320.18534** | **3426.25217** | **1465.28524** |
| **DALYs (Disability-Adjusted Life Years)** | **Global** | **Female** | **60-64 years** | **Low back pain** | **Number** | **2017** | **3618891.16** | **5339992.2** | **2285904.31** |
| **DALYs (Disability-Adjusted Life Years)** | **Global** | **Female** | **60-64 years** | **Low back pain** | **Rate** | **2017** | **2316.41465** | **3418.07355** | **1463.18361** |
| **DALYs (Disability-Adjusted Life Years)** | **Global** | **Female** | **60-64 years** | **Low back pain** | **Number** | **2018** | **3672705.61** | **5421076.17** | **2321185.02** |
| **DALYs (Disability-Adjusted Life Years)** | **Global** | **Female** | **60-64 years** | **Low back pain** | **Rate** | **2018** | **2316.75751** | **3419.63671** | **1464.21287** |
| **DALYs (Disability-Adjusted Life Years)** | **Global** | **Female** | **60-64 years** | **Low back pain** | **Number** | **2019** | **3716355.63** | **5470046.97** | **2342818.76** |
| **DALYs (Disability-Adjusted Life Years)** | **Global** | **Female** | **60-64 years** | **Low back pain** | **Rate** | **2019** | **2319.21384** | **3413.61535** | **1462.04998** |
| **DALYs (Disability-Adjusted Life Years)** | **Global** | **Female** | **60-64 years** | **Low back pain** | **Number** | **2020** | **3745139.63** | **5561675.58** | **2362659.7** |
| **DALYs (Disability-Adjusted Life Years)** | **Global** | **Female** | **60-64 years** | **Low back pain** | **Rate** | **2020** | **2312.60351** | **3434.30465** | **1458.92961** |
| **DALYs (Disability-Adjusted Life Years)** | **Global** | **Female** | **60-64 years** | **Low back pain** | **Number** | **2021** | **3803171.37** | **5601422.76** | **2393055.74** |
| **DALYs (Disability-Adjusted Life Years)** | **Global** | **Female** | **60-64 years** | **Low back pain** | **Rate** | **2021** | **2311.80463** | **3404.89392** | **1454.64845** |
| **Prevalence** | **Global** | **Female** | **60-64 years** | **Low back pain** | **Number** | **1990** | **18900618.8** | **24903798.3** | **13752711.1** |
| **Prevalence** | **Global** | **Female** | **60-64 years** | **Low back pain** | **Rate** | **1990** | **23031.804** | **30347.1228** | **16758.6972** |
| **Prevalence** | **Global** | **Female** | **60-64 years** | **Low back pain** | **Number** | **1991** | **18983916.6** | **24986136.8** | **13839914** |
| **Prevalence** | **Global** | **Female** | **60-64 years** | **Low back pain** | **Rate** | **1991** | **22747.9892** | **29940.3111** | **16584.0496** |
| **Prevalence** | **Global** | **Female** | **60-64 years** | **Low back pain** | **Number** | **1992** | **18971023.1** | **24946571.4** | **13857819.4** |
| **Prevalence** | **Global** | **Female** | **60-64 years** | **Low back pain** | **Rate** | **1992** | **22455.0034** | **29527.9461** | **16402.7728** |
| **Prevalence** | **Global** | **Female** | **60-64 years** | **Low back pain** | **Number** | **1993** | **18964179.4** | **24922346.1** | **13874296.9** |
[truncated: 241,147 more chars]
