# Supplementary material for: Global, regional, and national burden of low back pain in postmenopausal women from 1990 to 2021: a comprehensive analysis using data from the Global Burden of Disease Study 2021
Source: Front Endocrinol (Lausanne). 2025 Sep 26;16:1683183. doi: 10.3389/fendo.2025.1683183 (PMC12510828; doi:10.3389/fendo.2025.1683183)
Supplement: Supplementary file 2 [file DataSheet2.pdf]

Table 1 Regional Analysis of the Burden of Low Back Pain in Postmenopausal Women from 1990 to 2021

Location	Sex	Incidence(95%UI)		Incidence(95%UI)			DALY(95%UI)		DALY(95%UI)		
		Case,1990	ASIR(per100000),	Case,2021	ASIR(per100000),	EAPC of ASIR(95%CI),	Case,1990	ASDR(per100000),	Case,2021	ASDR(per100000),	EAPC of ASDR(95%CI),
			1990		2021	1990-2021		1990		2021	1990-2021
Global	Both	54793907.9 (45557317-64649618.5)	1335.4 (1110.3-1575.5)	109855843.5 (91292145.1-128356823.8)	1209.7 (1005.3-1413.4)	-0.27 (-0.29--0.26)	14956691.4 (10228044.8-20401083.9)	364.5 (249.3-497.2)	29470958.8 (20299620.1-39656202.3)	324.5 (223.5-436.7)	-0.31 (-0.33--0.3)
	Male	19631913 (16122483.9-23366912.6)	1031.3 (847-1227.5)	39581955.3 (32674724.6-46718109)	925.9 (764.3-1092.8)	-0.31 (-0.32--0.3)	5193579.2 (3526648.8-7133546.3)	272.8 (185.3-374.7)	10392850.6 (7120333.6-14102954.8)	243.1 (166.6-329.9)	-0.32 (-0.33--0.3)
	Female	35161994.9 (29292850.2-41189119.9)	1598.5 (1331.7-1872.5)	70273888.3 (58553412.9-81718501.4)	1462.1 (1218.2-1700.2)	-0.24 (-0.26--0.22)	9763112.1 (6744495.3-13185106.8)	443.8 (306.6-599.4)	19078108.2 (13229420.8-25575222.4)	396.9 (275.2-532.1)	-0.29 (-0.32--0.27)

**High SDI**	Both	16810521.8 (14058004.8-19684318.5)	1475.2 (1233.7-1727.4)	30064934.2 (25454373.5-34657041.3)	1425.9 (1207.2-1643.7)	-0.06 (-0.08--0.04)	4651438.5 (3218875.1-6262043.1)	408.2 (282.5-549.5)	8172142.2 (5772313.9-10848923.3)	387.6 (273.8-514.5)	-0.11 (-0.12--0.09)
	Male	5995650.9 (4979407.5-7070198.6)	1212.9 (1007.3-1430.3)	11568362.8 (9765035-13311340.3)	1178.6 (994.9-1356.2)	-0.03 (-0.06--0.01)	1652596.8 (1134818.2-2253793.7)	334.3 (229.6-455.9)	3151405.1 (2228479.8-4194002.8)	321.1 (227-427.3)	-0.06 (-0.08--0.03)
	Female	10814870.9 (9106520.8-12599975.9)	1676.2 (1411.4-1952.8)	18496571.5 (15758611-21225253.8)	1641.2 (1398.3-1883.3)	-0.02 (-0.04-0)	2998841.8 (2090452.4-4012211.8)	464.8 (324-621.8)	5020737.1 (3570804.2-6638984.5)	445.5 (316.8-589.1)	-0.08 (-0.1--0.06)
**High-middle SDI**	Both	15500277.6 (12965242.6-18181724.7)	1470.1 (1229.7-1724.4)	26580280.8 (21992910.7-31078414.8)	1254.6 (1038.1-1466.9)	-0.47 (-0.5--0.45)	4280725.1 (2932716-5831199.6)	406 (278.2-553.1)	7175199.4 (4930972.5-9628619.5)	338.7 (232.7-454.5)	-0.53 (-0.55--0.5)
	Male	5247620.3 (4316514.3-6222687)	1141.3 (938.8-1353.4)	9269338.6 (7636054.6-10975065.3)	955.3 (787-1131.1)	-0.55 (-0.58--0.52)	1376075.2 (933750-1893617.5)	299.3 (203.1-411.8)	2421906.5 (1645547.2-3307594.6)	249.6 (169.6-340.9)	-0.54 (-0.57--0.52)
	Fema	10252657.3 (8574381.6-	1724.4 (1442.1	17310942.1 (14343474.7-	1507.5 (1249.1	-0.39 (-0.4	2904649.9 (2009966.4-	488.5 (338.1	4753293 (3300839.7-	413.9 (287.4	-0.47 (-0.49

	le	11945069.1)	-2009)	20146559.4)	-1754.4)	2--0.37)	3935025.7)	-661.8)	6379353.1)	-555.5)	--0.44)
**Middle SDI**	Both	12076824.5 (9852017.1-14409737.4)	1138.6 (928.8-1358.5)	29827967.7 (24517588.8-35288900.3)	1038.8 (853.8-1228.9)	-0.22 (-0.25--0.19)	3242801.5 (2195843.2-4456004.7)	305.7 (207-420.1)	7874295.5 (5328741.1-10691410.4)	274.2 (185.6-372.3)	-0.25 (-0.29--0.21)
	Male	4312772.6 (3486934.8-5201583.5)	834.5 (674.7-1006.4)	10299863.4 (8376941.5-12369203.1)	753.3 (612.6-904.6)	-0.28 (-0.3--0.26)	1117940.8 (751167.8-1550449.5)	216.3 (145.3-300)	2644649.4 (1790299.5-3621809)	193.4 (130.9-264.9)	-0.29 (-0.31--0.27)
	Female	7764051.9 (6377313.1-9245306.8)	1427.6 (1172.6-1700)	19528104.2 (16133207.1-23044299.8)	1298.3 (1072.6-1532.1)	-0.21 (-0.26--0.17)	2124860.6 (1439838.3-2908342.5)	390.7 (264.7-534.8)	5229646.1 (3565664.8-7106807.6)	347.7 (237.1-472.5)	-0.26 (-0.31--0.2)
**Low-middle SDI**	Both	7445138.8 (6107343.1-8864452.6)	1208.6 (991.4-1439)	17211155.8 (14146501.5-20425047.7)	1168.2 (960.2-1386.3)	-0.11 (-0.15--0.07)	1987999.5 (1351096.9-2726372.3)	322.7 (219.3-442.6)	4597572 (3110225.6-6307742.2)	312.1 (211.1-428.1)	-0.09 (-0.13--0.06)
	Male	2859960.7 (2335047.3-3432475.6)	912.4 (745-1095.1)	6077694.4 (4946331.5-7298301.9)	860.4 (700.2-1033.2)	-0.19 (-0.23--0.15)	732643.4 (495021.7-1007901.9)	233.7 (157.9-321.6)	1560537.5 (1048592.8-2147122.8)	220.9 (148.4-304)	-0.17 (-0.21--0.12)

	Female	4585178.1 (3769042.9-5427544.1)	1515.3 (1245.6-1793.7)	11133461.4 (9170940.8-13149306.8)	1451.7 (1195.8-1714.5)	-0.14 (-0.17--0.1)	1255356 (859439.3-1722098.8)	414.9 (284-569.1)	3037034.5 (2074275.9-4156587.1)	396 (270.5-542)	-0.14 (-0.17--0.1)
**Low SDI**	Both	2890625.1 (2361205.2-3439246.7)	1267.8 (1035.6-1508.4)	6061268.9 (4958646.7-7234327.2)	1208.7 (988.8-1442.6)	-0.16 (-0.18--0.15)	773678 (529987.9-1063192.1)	339.3 (232.4-466.3)	1621060.7 (1104232.7-2225334.7)	323.3 (220.2-443.7)	-0.15 (-0.17--0.13)
	Male	1189023.6 (969222.3-1425049.4)	1015.6 (827.8-1217.2)	2324574.4 (1890410.1-2786369.8)	945.7 (769.1-1133.6)	-0.24 (-0.26--0.22)	307070.5 (207778.1-423958.1)	262.3 (177.5-362.1)	603057.8 (408858.4-830363.6)	245.4 (166.3-337.8)	-0.21 (-0.24--0.19)
	Female	1701601.5 (1394367.2-2024559.1)	1534 (1257-1825.2)	3736694.5 (3061680.6-4444059.3)	1461.4 (1197.4-1738.1)	-0.16 (-0.18--0.15)	466607.5 (320184.3-638610.6)	420.7 (288.6-575.7)	1018002.9 (699654-1401691.2)	398.1 (273.6-548.2)	-0.17 (-0.19--0.15)
**Andean Latin America**	Both	185536.8 (151625.6-222105.9)	904.7 (739.3-1083)	559462.3 (463821.9-664278.7)	924.1 (766.1-1097.2)	0.09 (0.06-0.11)	48860.5 (33195-67513.7)	238.2 (161.9-329.2)	145825.8 (99602.1-197086.8)	240.9 (164.5-325.5)	0.07 (0.04-0.09)
	Male	74518.2 (60022.2-89796.	746.4 (601.2-899.	220174 (182085-259357.6)	761.1 (629.4-896.	0.08 (0.06-0.1)	19418.4 (13061.6-26724.	194.5 (130.8-	56845.3 (38935.1-78202)	196.5 (134.6-	0.06 (0.04-0.09)

		2)	4)		5)		3)	267.7)		270.3)	
	Female	111018.7 (91729.1-133079.2)	1054.8 (871.6-1264.5)	339288.2 (280313.1-400792.5)	1073.3 (886.7-1267.8)	0.08 (0.06-0.1)	29442.1 (20173.8-40642.4)	279.7 (191.7-386.2)	88980.5 (60760.6-119444.6)	281.5 (192.2-377.8)	0.06 (0.03-0.08)
**Australasia**	Both	381925.9 (321708.4-444854.1)	1586.4 (1336.2-1847.7)	815568.5 (676005.6-952939.5)	1510.6 (1252.1-1765.1)	-0.15 (-0.18--0.12)	106063 (74301.7-143417.2)	440.5 (308.6-595.7)	224686.2 (155763.9-301330)	416.2 (288.5-558.1)	-0.19 (-0.21--0.17)
	Male	128180.4 (106346.7-151498.8)	1160.6 (962.9-1371.8)	270486 (223228.3-320169.1)	1053.5 (869.4-1247)	-0.25 (-0.3--0.21)	34648.4 (23888.2-46892.5)	313.7 (216.3-424.6)	72508.5 (50207.1-98325.6)	282.4 (195.5-383)	-0.29 (-0.33--0.25)
	Female	253745.5 (212799.7-294429.8)	1947.1 (1632.9-2259.3)	545082.5 (452759.5-634938.4)	1925.2 (1599.1-2242.5)	-0.05 (-0.09-0)	71414.6 (50648-97263.7)	548 (388.7-746.4)	152177.8 (104950.5-202916.6)	537.5 (370.7-716.7)	-0.09 (-0.12--0.05)
**Caribbean**	Both	247570.9 (203455-293535.5)	940 (772.5-1114.5)	526582.1 (440810.8-615831)	930.7 (779.1-1088.4)	-0.02 (-0.03--0.01)	65704.7 (44587.9-89458.3)	249.5 (169.3-339.7)	138144.8 (96325.9-186396.8)	244.2 (170.2-329.4)	-0.04 (-0.05--0.03)
	M	93403.	733	189366	712.	-	24173	18	48485.	18	-0.1

	ale	4 (76650-111642.8)	(601.5-876.1)	.5 (157473.2-225163.7)	5 (592.5-847.2)	0.08 (-0.09--0.08)	(16415.5-32848.6)	9.7 (128.8-257.8)	9 (33307.9-65469.5)	2.4 (125.3-246.3)	(-0.11--0.09)
	Female	154167.5 (126944.1-183371.7)	1134 (933.7-1348.8)	337215.6 (281712.5-392977.2)	1123.9 (938.9-1309.7)	-0.01 (-0.02-0)	41531.7 (28077-56792.5)	305.5 (206.5-417.7)	89658.9 (62685.9-120160)	298.8 (208.9-400.5)	-0.03 (-0.04--0.02)
**Central Asia**	Both	738759.4 (617146.9-873657)	1511.4 (1262.6-1787.4)	1301698.3 (1072508.2-1543234.6)	1463.9 (1206.2-1735.6)	-0.11 (-0.16--0.05)	203973.7 (142724.3-276890.1)	417.3 (292-566.5)	355560.4 (244543.3-494356.7)	399.9 (275-556)	-0.12 (-0.16--0.07)
	Male	251900.9 (207428.1-302981.1)	1272.1 (1047.5-1530.1)	491802.1 (398612.8-590436.2)	1260 (1021.2-1512.7)	-0.06 (-0.15-0.02)	65523.1 (44783.7-89747.6)	330.9 (226.2-453.2)	128062.8 (86831.6-178481.5)	328.1 (222.5-457.3)	-0.04 (-0.12-0.04)
	Female	486858.6 (406488.2-572478.5)	1674.4 (1398-1968.8)	809896.1 (668401.3-957323.4)	1623.5 (1339.9-1919)	-0.1 (-0.14--0.05)	138450.6 (97861.2-188997.4)	476.2 (336.6-650)	227497.7 (156835-315904.8)	456 (314.4-633.3)	-0.11 (-0.14--0.08)
**Central Europe**	Both	2957573.9 (2466349.5-3467574.5)	1824.8 (1521.7-213	4147628.5 (3443045.7-4847843.6)	1832.9 (1521.5-214	-0.02 (-0.05-0)	865441.4 (598958.1-1180086.6)	534 (369.6-72	1199163.3 (834446.5-1619291.7)	529.9 (368.7-71	-0.04 (-0.06--0.02

			9.5)		2.3)			8.1)		5.6)	)
	Male	1153243.8 (955160.9-1362252.5)	1647.3 (1364.3-1945.8)	1643368.3 (1362286.4-1947159.6)	1661.7 (1377.5-1968.9)	-0.01 (-0.04-0.01)	314853.8 (214448.3-429429.1)	449.7 (306.3-613.4)	449568.1 (307399.7-604475.2)	454.6 (310.8-611.2)	0.01 (-0.02-0.03)
	Female	1804330.1 (1496334.4-2110093.2)	1959.8 (1625.3-2291.9)	2504260.2 (2081775.6-2922791.8)	1965.7 (1634.1-2294.3)	-0.02 (-0.04--0.01)	550587.6 (385634.4-752690.8)	598 (418.9-817.6)	749595.1 (526791.6-1017647.8)	588.4 (413.5-798.8)	-0.05 (-0.07--0.04)
**Central Latin America**	Both	849948.6 (697608.2-1013286.3)	1024.9 (841.2-1221.8)	2698010.3 (2214968.4-3193477.5)	1032.3 (847.5-1221.9)	0.04 (0.01-0.08)	226964.1 (152607.9-309905.4)	273.7 (184-373.7)	719812.6 (490965.1-988149.1)	275.4 (187.9-378.1)	0.05 (0.01-0.09)
	Male	300317.3 (245218.9-360912.7)	751.7 (613.8-903.4)	942311 (768079.9-1134414.8)	784.4 (639.4-944.3)	0.14 (0.11-0.17)	76400.1 (51465.8-104852.5)	191.2 (128.8-262.4)	243478.6 (165282.6-334370)	202.7 (137.6-278.3)	0.2 (0.17-0.22)
	Female	549631.3 (452163.4-658478.8)	1278.8 (1052-1532.1)	1755699.2 (1449558.3-2083076)	1243.2 (1026.4-1475)	-0.06 (-0.1--0.02)	150564 (101520-206016.3)	350.3 (236.2-479.3)	476334 (328946.3-654709.4)	337.3 (232.9-463.6)	-0.07 (-0.12--0.02)
**Central**	Bo	302628.2	1316.9	701742.6	1272.5	-0.1	80280.8	349.3	186725.4	338.6	-0.13

**Sub-Saharan Africa**	th	(244730.5-365446.4)	(1064.9-1590.2)	(568932.5-844330.9)	(1031.7-1531.1)	5 (-0.19--0.1)	(54922.1-111769.8)	(239-486.4)	(126347.8-257936.8)	(229.1-467.7)	(-0.18--0.08)
	Male	125846.1 (101379.6-150949.7)	1176.2 (947.5-1410.8)	282090 (225413.3-343454.3)	1119.7 (894.7-1363.3)	-0.19 (-0.23--0.15)	32731.6 (22142.7-45078.8)	305.9 (207-421.3)	73750 (49613.7-100853)	292.7 (196.9-400.3)	-0.15 (-0.19--0.12)
	Female	176782.1 (141181.6-213622.8)	1439.5 (1149.6-1739.4)	419652.6 (342459.4-503898.8)	1401 (1143.3-1682.3)	-0.12 (-0.16--0.08)	47549.2 (32358.8-66220.7)	387.2 (263.5-539.2)	112975.3 (76317.5-156461.5)	377.2 (254.8-522.4)	-0.12 (-0.16--0.07)
**East Asia**	Both	10419681.3 (8465584.7-12469538.3)	1144.6 (930-1369.8)	22983051.5 (18915371.7-27187454.6)	959.1 (789.3-1134.5)	-0.4 (-0.46--0.33)	2810444.4 (1883605.6-3885465.5)	308.7 (206.9-426.8)	6040797.7 (4102131.6-8198947.3)	252.1 (171.2-342.1)	-0.45 (-0.53--0.37)
	Male	3696709.1 (2963004.5-4466741)	826.9 (662.7-999.1)	7977215.1 (6504329.8-9524447.8)	688.1 (561.1-821.6)	-0.47 (-0.51--0.42)	970941.8 (649843.3-1354938.7)	217.2 (145.4-303.1)	2073259.9 (1417731.2-2849577.2)	178.8 (122.3-245.8)	-0.48 (-0.53--0.44)
	Female	6722972.2 (5491857.4-8053251.8)	1451.3 (1185.6-173	15005836.4 (12429376.7-17630330.8)	1213 (1004.7-142	-0.39 (-0.46--0.3	1839502.7 (1233239.6-2533309.8)	397.1 (266.2-54	3967537.8 (2695352-5359538.4)	320.7 (217.9-43	-0.46 (-0.55--0.36

			8.5)		5.1)	1)		6.9)		3.2)	)
**Eastern Europe**	Both	5589139.1 (4713281.9-6540636)	1870.5 (1577.4-2189)	6956041.1 (5783589.2-8125691.1)	1833.5 (1524.5-2141.8)	-0.06 (-0.11--0.01)	1601541.3 (1112686.1-2172460.2)	536 (372.4-727.1)	1958304.9 (1365433.4-2665272.5)	516.2 (359.9-702.5)	-0.07 (-0.12--0.02)
	Male	1601138.2 (1310618.4-1903458.6)	1511.6 (1237.3-1797)	2195687.2 (1812866.2-2626296)	1502.1 (1240.2-1796.6)	-0.05 (-0.12-0.02)	410736.7 (280431.9-567643.7)	387.8 (264.7-535.9)	563760 (383651.2-764594.8)	385.7 (262.5-523.1)	-0.01 (-0.08-0.05)
	Female	3988000.9 (3361846.1-4677994.2)	2067.6 (1743-2425.4)	4760354 (3938299.6-5551248.5)	2041.2 (1688.7-2380.4)	-0.04 (-0.07-0)	1190804.7 (832771.6-1620286)	617.4 (431.8-840.1)	1394544.9 (980538-1900698.2)	598 (420.5-815)	-0.05 (-0.09--0.01)
**Eastern Sub-Saharan Africa**	Both	998926.3 (822541.1-1190951.5)	1343.6 (1106.3-1601.9)	2149247.8 (1764756.9-2562158.8)	1300.7 (1068-1550.6)	-0.1 (-0.12--0.09)	268413.6 (183219.1-366227.5)	361 (246.4-492.6)	576849.8 (392696.8-794934.8)	349.1 (237.7-481.1)	-0.09 (-0.11--0.08)
	Male	414784.7 (340170.3-497259.6)	1110.6 (910.8-1331.4)	832883.4 (676428.5-998499)	1058.2 (859.4-1268.6)	-0.15 (-0.17--0.14)	107602.7 (72886-147514.3)	288.1 (195.1-395)	217729.6 (147623.5-300620.7)	276.6 (187.6-381.9)	-0.12 (-0.13--0.1)
	Fe	584141.6	1578.8	1316364.4	1521.3	-0.1	160810.9	434.6	359120.2	415	-0.13

	male	(480386.7-695169.2)	(1298.4-1878.9)	(1082532.7-1568289.1)	(1251.1-1812.5)	2 (-0.14--0.1)	(110208.4-219843.9)	(297.9-594.2)	(246483.8-494293.8)	(284.9-571.3)	(-0.15--0.12)
**High-income Asia Pacific**	Both	3194231.4 (2642893.1-3779385.3)	1494.7 (1236.7-1768.5)	6027762 (5038522.2-7039949.5)	1399 (1169.4-1633.9)	-0.16 (-0.19--0.13)	890733.5 (605411.6-1216709)	416.8 (283.3-569.3)	1651181.6 (1150931.5-2207027.9)	383.2 (267.1-512.2)	-0.23 (-0.26--0.2)
	Male	1141082.8 (930065.9-1361883.1)	1212.6 (988.3-1447.2)	2168847.4 (1806944.4-2547719.6)	1096.8 (913.8-1288.4)	-0.22 (-0.29--0.15)	310112.1 (209351.9-430210.8)	329.5 (222.5-457.2)	581382.5 (399196.3-784803.3)	294 (201.9-396.9)	-0.25 (-0.34--0.16)
	Female	2053148.6 (1700341.3-2403809)	1716.7 (1421.7-2009.9)	3858914.5 (3238396.1-4484380.5)	1655.3 (1389.1-1923.6)	-0.09 (-0.14--0.05)	580621.3 (399953.2-788543.3)	485.5 (334.4-659.3)	1069799.1 (747100.8-1428075.8)	458.9 (320.5-612.6)	-0.19 (-0.23--0.14)
**High-income North America**	Both	5171132.8 (4351419.9-6049819.4)	1460.7 (1229.1-1708.9)	9959782.8 (8723113.8-11213780.2)	1448.2 (1268.4-1630.5)	0.03 (-0.01-0.06)	1418275 (983037.5-1897750.4)	400.6 (277.7-536.1)	2638415.7 (1881403.3-3438538.5)	383.6 (273.6-500)	-0.05 (-0.09--0.01)
	Male	1945051.1 (1625398.7-2285999.3)	1268.9 (1060.4-149	4140741.1 (3617349.8-4685994.5)	1295.7 (1132-1466.4)	0.12 (0.08-0.16)	539959.3 (372841.4-730796.1)	352.3 (243.2-47	1115230.5 (798939.1-1448372.8)	349 (250-453.2	0.05 (0-0.09)

			1.4)					6.8)		)	
	Female	3226081.6 (2723263.1-3750438.1)	1607.1 (1356.6-1868.3)	5819041.8 (5096334.8-6527553.6)	1580.5 (1384.2-1773)	0.01 (-0.03-0.05)	878315.7 (613328.9-1166697.9)	437.5 (305.5-581.2)	1523185.2 (1083936.8-1979779.2)	413.7 (294.4-537.7)	-0.08 (-0.12--0.04)
**North Africa and Middle East**	Both	2188954.9 (1801268.2-2590327.2)	1267.3 (1042.8-1499.6)	5824251.3 (4792989.7-6887575.6)	1250.1 (1028.8-1478.4)	-0.04 (-0.07--0.02)	581713.6 (397626.4-793803.3)	336.8 (230.2-459.6)	1525157.2 (1039951.8-2087117.9)	327.4 (223.2-448)	-0.07 (-0.1--0.04)
	Male	1011263 (836720.1-1198309.2)	1149.8 (951.3-1362.5)	2611614.5 (2148247.9-3109185.5)	1107.5 (911-1318.5)	-0.11 (-0.14--0.08)	261780.2 (178858.5-358515.5)	297.6 (203.4-407.6)	663898.9 (451918.6-905605.9)	281.5 (191.6-384)	-0.13 (-0.17--0.09)
	Female	1177691.9 (970757.1-1392762.5)	1389.2 (1145.1-1642.8)	3212636.8 (2639678-3782428)	1396.3 (1147.3-1643.9)	0.01 (-0.02-0.03)	319933.4 (219761.1-436717.8)	377.4 (259.2-515.1)	861258.3 (589440.8-1176909.4)	374.3 (256.2-511.5)	-0.02 (-0.04-0)
**Oceania**	Both	30579.8 (24435.8-37135.9)	1040.1 (831.1-1263.1)	76968.8 (62541.3-93687)	1020.5 (829.2-1242.1)	-0.08 (-0.1--0.06)	8113.7 (5521.1-11386.3)	276 (187.8-387.3)	20361.1 (13834.1-27900.4)	270 (183.4-369.9)	-0.07 (-0.09--0.05)
	Ma	12032.7	787.7	30313.7	767.4	-0.1	3108.1 (2120.	203.5	7851.5 (5281-	198.8	-0.08

	le	(9560-14629.6)	(625.8-957.7)	(24057.2-37133.2)	(609-940.1)	(-0.12--0.07)	3-4388.7)	(138.8-287.3)	10865.5)	(133.7-275.1)	(-0.1--0.05)
	Female	18547.1 (14794-22493.8)	1313.1 (1047.4-1592.5)	46655.2 (38039.3-56700)	1298.7 (1058.9-1578.3)	-0.04 (-0.06--0.02)	5005.7 (3393.5-6979.1)	354.4 (240.3-494.1)	12509.6 (8478.6-17218.7)	348.2 (236-479.3)	-0.04 (-0.06--0.02)
**South Asia**	Both	7088815.6 (5797594.6-8461934)	1221.7 (999.2-1458.4)	17305586.2 (14200486.8-20601162.4)	1140.5 (935.8-1357.6)	-0.23 (-0.3--0.15)	1882473.4 (1283364.5-2599704.3)	324.4 (221.2-448.1)	4588383.9 (3101132.6-6283787.8)	302.4 (204.4-414.1)	-0.22 (-0.29--0.14)
	M	26927	883	584477	786	-	68517	22	14853	19	-
	Female	4396039.7 (3597441.7-5218506)	1597 (1306.9-1895.8)	11460812.6 (9403886.3-13590111.9)	1481.2 (1215.3-1756.4)	-0.25 (-0.32--0.17)	1197298.6 (819515.8-1649107.7)	435 (297.7-599.1)	3103073.9 (2115862.7-4237387.1)	401 (273.5-547.6)	-0.25 (-0.33--0.17)
**Southeast Asia**	Both	2521702 (2074317.6-3008988.7)	974.5 (801.6-1162.8)	6701640.8 (5479338.6-7996958.1)	957.3 (782.7-1142.3)	-0.06 (-0.08--0.03)	665862.6 (456040.6-910909.5)	257.3 (176.2-352)	1778283.3 (1204249.9-2434022.4)	254 (172-347.7)	-0.03 (-0.05-0)
	Male	793358.4 (645101.8-961546.6)	655.2 (532.8-794.1)	2069760.7 (1663869.9-2509856.9)	636.2 (511.4-771.5)	-0.08 (-0.1--0.0	200838.8 (134929.9-277183.1)	165.9 (111.4-22	528564 (357670.6-734858.6)	162.5 (109.9-22	-0.04 (-0.05--0.02

						7)		8.9)		5.9)	)
	Female	1728343.6 (1420598.4-2059513.1)	1255.3 (1031.8-1495.9)	4631880.1 (3816652.6-5491702.3)	1236 (1018.4-1465.4)	-0.04 (-0.07--0.02)	465023.8 (320249.1-634997.3)	337.8 (232.6-461.2)	1249719.3 (851100.3-1714201.5)	333.5 (227.1-457.4)	-0.02 (-0.04-0)
**Southern Latin America**	Both	663195.3 (548308.8-778118.3)	1369.9 (1132.6-1607.3)	1242357.7 (1029986.2-1461657.4)	1381.4 (1145.3-1625.2)	0.01 (-0.01-0.04)	180054.6 (123726.4-245039.5)	371.9 (255.6-506.2)	337120 (231395.2-454528.7)	374.8 (257.3-505.4)	-0.01 (-0.04-0.01)
	Male	204660.5 (167256.1-244472.3)	947 (773.9-1131.2)	372623 (303538.7-444506.3)	926.8 (755-1105.6)	-0.05 (-0.08--0.02)	53973.1 (36417.8-73954.1)	249.7 (168.5-342.2)	98481.5 (66558.1-134819.1)	245 (165.5-335.3)	-0.04 (-0.07--0.02)
	Female	458534.8 (380822-536141.3)	1711 (1421-2000.5)	869734.7 (719821.9-1020604.6)	1748.9 (1447.4-2052.3)	0.05 (0-0.1)	126081.5 (87735.7-171110.7)	470.5 (327.4-638.5)	238638.5 (166464.1-321703.9)	479.9 (334.7-646.9)	0.01 (-0.04-0.06)
**Southern Sub-Saharan Africa**	Both	340878.1 (281900.2-402524)	1260.6 (1042.5-1488.6)	710425.3 (581567.6-844322.3)	1194.1 (977.5-1419.1)	-0.17 (-0.18--0.15)	91173 (62335.1-123936.9)	337.2 (230.5-458.3)	185685.3 (127134.6-252650.7)	312.1 (213.7-424.7)	-0.21 (-0.23--0.2)
	Ma	115374.2	990.7	237302.5	960.9	-0.1	29799.2	255.9	60891.1	246.6	-0.11

	le	(94816.9-136121.3)	(814.2-1168.9)	(193028.8-285174.6)	(781.6-1154.8)	2 (-0.15--0.1)	(20315.7-40677.4)	(174.4-349.3)	(41695-83349.7)	(168.8-337.5)	(-0.12--0.09)
	Female	225503.9 (186759.6-265430.9)	1464.7 (1213.1-1724.1)	473122.7 (388048.2-562497.9)	1359.5 (1115.1-1616.3)	-0.2 (-0.22--0.19)	61373.8 (42209.9-83735.6)	398.6 (274.2-543.9)	124794.2 (85078.9-169622.3)	358.6 (244.5-487.4)	-0.28 (-0.3--0.26)
**Tropical Latin America**	Both	1160909.2 (949278.1-1384821.8)	1254.6 (1025.9-1496.6)	3595129.8 (2971749.5-4236446.7)	1328 (1097.7-1564.9)	0.14 (0.1-0.18)	315112.3 (214602.5-432139.3)	340.5 (231.9-467)	987817.8 (680881-1338032.7)	364.9 (251.5-494.3)	0.18 (0.13-0.22)
	Male	436007.9 (354517.8-522995.7)	1008.4 (819.9-1209.6)	1294084.1 (1066607.4-1539194.3)	1060.4 (874-1261.3)	0.11 (0.07-0.15)	114543.3 (78165.6-156487.3)	264.9 (180.8-361.9)	342626.5 (232719-464982.1)	280.8 (190.7-381)	0.15 (0.11-0.18)
	Female	724901.3 (593863-863468.4)	1470.5 (1204.7-1751.6)	2301045.7 (1898902.6-2695709.4)	1547.6 (1277.2-1813.1)	0.13 (0.09-0.18)	200568.9 (136404.9-275684)	406.9 (276.7-559.2)	645191.3 (451538.6-877221.4)	433.9 (303.7-590)	0.16 (0.11-0.21)
**Western Europe**	Both	8692773.3 (7332780.7-10167469.8)	1464.7 (1235.5-171	13274682.5 (11081293-15513755.8)	1456.5 (1215.8-170	0.04 (0.02-0.06)	2363656.3 (1652428-3168909.9)	398.3 (278.4-53	3604461.8 (2514690.8-4847206.6)	395.5 (275.9-53	0.03 (0.01-0.05)

			3.2)		2.2)			3.9)		1.8)	
	Male	2974883 (2482797.3-3500404)	1171.7 (977.9-1378.6)	4870927.5 (4021735.2-5748972.9)	1155.9 (954.4-1364.3)	0.02 (-0.01-0.05)	816398.7 (562590.5-1107527.6)	321.5 (221.6-436.2)	1346689.5 (921984.5-1840419.4)	319.6 (218.8-436.8)	0.03 (0-0.05)
	Female	5717890.3 (4849645.9-6619589.8)	1683.8 (1428.1-1949.3)	8403755 (7035741.3-9768717.3)	1715 (1435.8-1993.5)	0.11 (0.09-0.14)	1547257.6 (1084824.5-2059646.8)	455.6 (319.5-606.5)	2257772.4 (1581274.1-3007680.7)	460.7 (322.7-613.8)	0.09 (0.07-0.11)
**Western Sub-Saharan Africa**	Both	1069045.1 (878674.6-1267886.8)	1211.8 (996-1437.2)	2298223.5 (1878171-2732132.3)	1169.9 (956.1-1390.8)	-0.13 (-0.16--0.09)	281835.8 (192456-383812)	319.5 (218.2-435.1)	608220.5 (416678-838322.4)	309.6 (212.1-426.8)	-0.11 (-0.14--0.08)
	Male	465381.7 (380182.5-558452.8)	1018.4 (831.9-1222)	895583.6 (732352.7-1071713.9)	973.2 (795.8-1164.6)	-0.17 (-0.22--0.12)	120862 (82084.3-165941.9)	264.5 (179.6-363.1)	234476.2 (159085.9-323131.9)	254.8 (172.9-351.1)	-0.13 (-0.18--0.09)
	Female	603663.4 (498977.2-714337.2)	1419.7 (1173.5-1680)	1402639.9 (1143133.9-1666945.2)	1343.3 (1094.8-1596.5)	-0.19 (-0.23--0.15)	160973.8 (110020.4-217716.2)	378.6 (258.7-512)	373744.3 (257562.3-513861)	357.9 (246.7-492.1)	-0.19 (-0.22--0.15)
